# Identification of Traits Contributing to High and Stable Yields in Different Soybean Varieties Across Three Chinese Latitudes

**DOI:** 10.3389/fpls.2019.01642

**Published:** 2020-01-21

**Authors:** Minmin Li, Ying Liu, Chunsheng Wang, Xue Yang, Dongmei Li, Xiaoming Zhang, Chongjing Xu, Yan Zhang, Wenbin Li, Lin Zhao

**Affiliations:** Key Laboratory of Soybean Biology of Ministry of Education, China (Key Laboratory of Biology and Genetics & Breeding for Soybean in Northeast China), Northeast Agricultural University, Harbin, China

**Keywords:** soybean yield per plant, agronomic traits, correlation analysis, genetic diversity, genotype plus genotype × environment interaction biplot

## Abstract

Soybean yield is a complex quantitative trait, which is greatly affected by environmental conditions. The main objective of this study is not only to identify specific traits contributing to yield in different latitudes, which can be further used in breeding, but also to identify the outperforming varieties, as this can help to select new lines with these traits. One hundred and seventy-three soybean genotypes were tested in three different ecological environments, including Harbin, Changchun, and Shenyang in China during 2015–2016 cropping seasons. The evaluation on the different agronomic and physiological traits indicated that the soybean varieties with higher plant height, more nodes of main stem, branches, pods, grains, and 100-grain weight, or longer growth periods may have higher yield. Pods, grains and 100-grain weight can be used as direct selection criteria for yield increase, and likewise the other traits such as plant height, nodes of main stem, branches, growth periods indirectly affected yield by affecting the three traits above. The effect of genotype × environment (G × E) interaction on different agronomic traits was significant. The representativeness and discriminability for grains yield per plant was the most significant in Harbin, which could be used to screen varieties with high yield and wider adaptability. Genotype “Suinong 1” was considered stable with higher value of grain yield per plant than other genotypes used in this study. As the yield of certain soybean cultivars may be significantly reduced if they are grown in a region as little as 2°N beyond its normal cultivation latitudes, therefore, the identification and analysis on the stable and widely adaptive soybean genotypes would be very important, and it would provide the significant reference accordance of soybean variety selection for the soybean breeders.

## Introduction

Soybean yield is a complex quantitative trait controlled by many genes, and it is determined by multiple interactions between genes and environment (Li et al., 2008), greatly affected by environmental conditions especially day length of different latitude (Singh and Vatsa, 2009; Zhang et al., 2015; Bhartiya and Aditya, 2016; AbdulHamid et al., 2017). Photoperiod is the leading climatic factor in determining soybean adaptation to different eco-regions (Câmara et al., 1997). Due to photoperiodic sensitivity, the cultivation area of each soybean cultivar was restricted to a very narrow range of latitudes to attain its highest yield (Cober and Morrison, 2010). The yield of certain soybean cultivars was significantly reduced when they were cultivated 2°N beyond its typical cultivation latitudes (Gai and Wang, 2001). Nevertheless, soybean is grown worldwide in a broad range of latitudes (50°N–35°S) (Mcblain et al., 1987). Therefore, it is important to identify among the highest and most stable soybean varieties available, which traits are determining the best performance at different latitudes.

Plant breeders often sought to improve the yield by selecting for components of yield (Dao et al., 2017). Correlation studies accompanied with path coefficient analysis provided an effective means of partitioning the genotypic correlation coefficients into direct and indirect effects and made a clear understanding of their associations with grain yield (Debebe et al., 2014). Path analysis had been used to identify the traits that had significant effects on grain yield in soybean (Li et al., 2013; Yahaya and Ankrumah, 2017; AlBallat and Al-Araby, 2019). Genotype × environment (G × E) interaction is a major problem in the study of quantitative traits such as yield and yield component, because it complicated the interpretation of genetic experiments and predictions (Farshadfar and Sutka, 2003; Becker and Leon, 2010).

On a routine basis in crop breeding programs, genotypes are evaluated in multi-environment trials to test their performance across environments and selecting the best genotypes in specific environments. A significant G × E interaction for a quantitative trait such as grain yield could seriously restrict the progress of variety adaptive selection (Reddy et al., 2011; Luo et al., 2012). A large number of studies had been carried out to determine the effects of G × E interaction on crop yield and other agronomic traits by several statistical modeling methods (Gravois and Bernhardt, 2000; Casanoves et al., 2005; Grüneberg et al., 2005; Luo et al., 2009). These methods may use linear models, such as joint regression analysis (Yates and Cochran, 1938; Eberhart and Russell, 1966), multivariate analytical methods such as AMMI (additive main effects and multiplicative interaction) analysis (Zobel et al., 1988; Gauch, 1994), or GGE (genotype plus G × E interaction) biplot analysis (Yan et al., 2000a; Yan et al., 2000b; Yan, 2002).

Of these, GGE biplot visually examined the relationships among genotypes, test environments, and genotype-by-environment interactions, which was an effective method for recommending specific genotypes in specific mega-environments, evaluating the mean performance and stability of genotypes, and analyzing the power of target environments to distinguish genotypes (Yan and Kang, 2003; Tiwari, 2019). The greater use of GGE biplot came into play when genotypes were tested across a wide range of environments where the interaction between genotype and environment played a significant role (as in advanced stages of testing) as well as when a large number of hybrids were evaluated in fewer locations (as in early stages of testing) where the primary objective was to discard inferior genotypes. The stability and adaptability of 121 soybean varieties planted in three test sites were analyzed using GGE biplot, and the local variety “Yapoche” showed high and stable protein content and special adaptability to Harbin (Wu et al., 2015). The high-yield and stable variety AN2 was identified by analyzing 24 soybean germplasm resources planted at eight test locations using GGE biplot (Zhou, 2012). Moreover, the characteristics of stable and high yield of oat germplasms in regional trial were also analyzed using GGE biplot, and the discriminative power and representativeness of different environments for yield traits were determined. Finally, three high-yield and stable-yield oat lines in the national oat regional trial were screened (Zhang et al., 2010). Several sugarcane varieties that could adapt to a wider range of conditions were also identified by GGE biplot (Luo et al., 2015). In conclusion, GGE biplot tool had become increasingly popular in cultivar evaluation and mega-environment investigation for plant breeders and agricultural researchers (Yan et al., 2000b), and many studies on G × E interaction effect on crop growth, yield, and other agronomic traits had been conducted in various plants using GGE biplot.

In this study, we evaluated the effects of G × E interaction on phenotypic variation of different agronomic traits to minimize the environmental effects, and identified the soybean varieties with stable and the best yield performance according to their significant interactions with the environment. Therefore, the performance of soybean cultivars was significantly influenced by G × E interaction, which made it difficult to identify superior cultivars that were stable throughout the different ecological region. The identification of stable and widely adaptive soybean genotypes would provide the soybean selection significant accordance for the soybean breeders.

## Materials and Methods

### Plant Materials and Planting Ecological Regions

**Figure 1:**
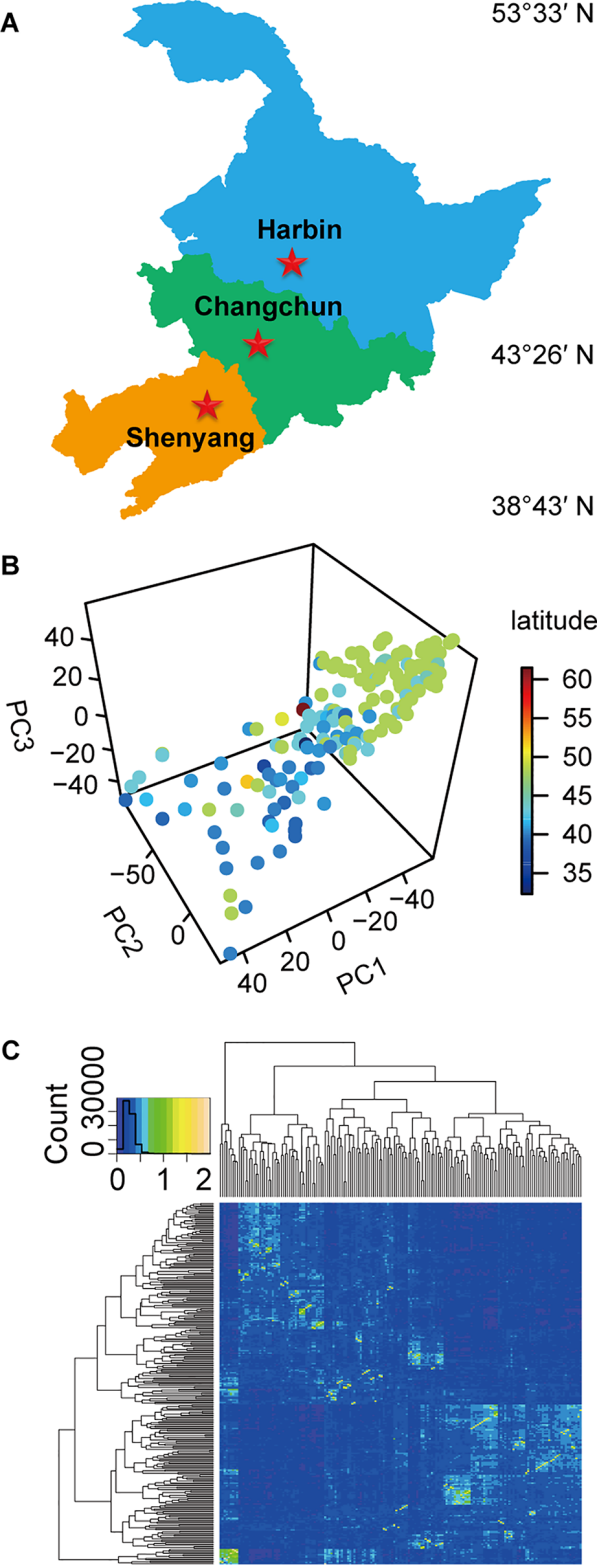
The experimental locations, population structure, and kinship relationship of materials. **(A)** The three experimental testing locations in China. **(B)** The scatterplot of the first three principal components from principal component analysis (PCA) of population structure for 173 soybean varieties. **(C)** The kinship relationship of population structure for 173 soybean varieties.

The experiments were carried out in Harbin, Changchun, and Shenyang in China, representing three different climatic conditions (**Figure 1A**). The longitude, latitude, soil type, precipitation, and environmental parameters of these test ecological locations were listed in **Table S1**. The experimental materials consisting of 173 soybean germplasm resources (**Table S2**) (Han et al., 2015; Zhao et al., 2018) were distributed in 32.3~61.5°N. Among these, 159 soybean germplasms originated from China, and 14 germplasms originated from other countries. Nineteen standard varieties from MGs000-IV (**Table S3**) (Boerma and Specht, 2004) originating from North America were used as reference to divide the maturity groups (MGs) of the evaluated soybeans.

### Experimental Design and Investigation Methods

Soybean was sowed manually at the beginning of May in 2015 and 2016 using completely randomized block experimental designs with two replications in three experimental locations and harvested manually at the beginning of October annually. Each experimental block consisted of 2-m-long rows with 0.6 m row spacing and 0.05 m plant spacing. Fourteen traits of 10 plants randomly selected in each block were recorded, including plant height, number of nodes of main stem, number of branches per plant, number of pods per plant, grain number per plant, grain yield per plant and 100-grain weight, beginning bloom (R1), full bloom (R2), beginning pod (R3), full pod (R4), beginning seed (R5), full seed (R6), and beginning maturity (R7), and the average values were calculated for the statistical analysis. Of these, the seven traits including plant height, number of nodes of main stem, number of branches per plant, number of pods per plant, grain number per plant, grain yield per plant, and 100-grain weight were measured after the harvesting time (Chen et al., 2006). The other seven growth periods such as R1, R2, R3, R4, R5, R6, and R7 were recorded (Fehr et al., 1971) in the process of soybean growth. The MGs of various varieties were also identified using an MG classification system consisting of 13 MGs (MG000–MGVIII) (**Table S3**) (Wang et al., 2006; Abdurakhmonov and Abdukarimov, 2008; Alliprandini et al., 2009). The mid-range values of R7 in 13 standard MGs were calculated, and the averages of mid-range values for two adjacent MGs were used as the threshold. The 173 soybeans were classified into different MGs (Jia et al., 2014).

### Statistical Analysis

#### Descriptive and Correlation Analysis

The descriptively statistical analyses were carried out by SPSS 19.0 software (Field, 2013). The phenotypic data were ranked by individual cases and transformed into variables obeying standard normal distribution for the statistical analysis by SPSS 19.0 software (Field, 2013). The significance of interrelationships between 14 phenotypic variables across three locations was identified using Pearson’s correlation coefficients of “Performance Analytics” package in R software (Micheaux et al., 2013). The correlation coefficients were formulated as follows (Wen et al., 2012):

$$r=\frac{\sum_{i=1}^{n}(x_{i}-\bar{x})\left(y_{i}-\bar{y}\right)}{\sqrt{\sum_{i=1}^{n}{\left(x_{i}-\bar{x}\right)}^{2}}\sqrt{\sum_{i=1}^{n}{\left(y_{i}-\bar{y}\right)}^{2}}}.$$

where $\bar{x}$ and $\bar{y}$ represented the average values of samples *x_i_ y_i_* respectively. The 14 phenotypic variables of 173 soybean varieties with genetic diversity were computed by principal component analysis (PCA) by “FactoMineR” package in R software (Le et al., 2008). Path analysis based on stepwise regression analysis was carried out by DPS v14.1.0 software (Tang and Zhang, 2013) to gradually remove those traits that had no significant effect on yield, and the direct and indirect effects of each trait on yield per plant were further clearly understood. Multiple regression models could generally be expressed as (Britto, 1985):

$$y=B_{0}+B_{1}x_{1}+B_{2}x_{2}+\cdots +B_{k}x_{k}+\varepsilon .$$

where *B*_0_ *B*_1_ *B*_2_..., *B*_k_ were the parameters of the model, and *ε* as the error term. The optimal linear regression equation of yield was established by eliminating the independent variables with the smallest squared sum and less significant levels.

#### Combined Analysis of Variance

Analysis of variance (ANOVA) was performed on the 14 phenotypic variables following the standard procedure of a mixed effect model using DPS v14.1.0 software to determine the level of the significance of genotype differences, locations, cultivation years, and their interactions (Tang and Zhang, 2013). Genotype and location were considered as the fixed effects, while year was considered as a random effect. The phenotypic observation *Z_ijkr_* was modeled as:

$$Z_{ijkr}=\text{μ}+G_{i}+L_{j}+Y_{k}+B_{r}\left(L_{j}Y_{k}\right)+GL_{ij}+GY_{ik}+LY_{jk}+GLY_{ijk}+e_{ijkr}.$$

where *Z_ijkr_* was the response variable; μ was the grand mean; *G_i_* was the genotype effect; *L_j_* as the location effect; *Y_k_* was the year effect; *B_r_* (*L_j_Y_k_*) was the block effect; *GL_ij_* was the genotype-by-location interaction; *GY_ik_* was the genotype-by-year interaction; *LY_jk_* was the location-by-year interaction; *GLY_ijk_* was the genotype-by-location-by-year interaction; and *e_ijkr_* is the residual error. The broad-sense heritability (*h^2^*) at individual environment was estimated based on ANOVA, and the formula used was (Jamoza et al., 2014):

$${\text{h}}^{2}=\frac{\sigma_{g}^{2}}{\sigma_{g}^{2}+\sigma_{\mathrm{gy}}^{2}/y+\sigma_{\mathrm{gl}}^{2}/l+\sigma_{\mathrm{gly}}^{2}/ly+\sigma_{\varepsilon }^{2}/\mathrm{rly}}.$$

where σ_*g*_^2^ was genotype, σ_*gl*_^2^ was genotype-by-location, σ_*gy*_^2^ was genotype-by-crop year, σ_*gly*_^2^ was genotype-by-location-by-year, σ_*ϵ*_^2^ was error, *r* was number of replications, *l* was number of locations, and *y* was crop years respectively.

#### AMMI Model

Magnitude of genotype, environment, and G × E interaction was assessed through ANOVA of AMMI method (Gauch, 1992) with the genotype as fixed and environment as random effects using “Agricolae” package in R software (Mendiburu and Simon, 2015). The AMMI model for genotypes and environments was as:

$$Y_{ij}=\mu +g_{i}+e_{j}+\overset{k}{\underset{n=1}{\sum }}\lambda_{n}\alpha_{in}\gamma_{jn}+\varepsilon_{ij}.$$

where *Y_ij_* was the target trait response of the *i^th^* genotype in the *j^th^* environment; *μ* was the general mean; *g_i_* was the *i^th^* genotypic effect; *e_j_* was the *j^th^* environment effect; *λ_n_* was the singular value of the *n^th^* principal interaction axis; *α_in_* and *γ_jn_* were the *i^th^* element of the singular column vector associated to axis n and the *j^th^* element of the singular row vector associated to axis n; *ε_ij_* was the AMMI residual; and *k* was the number of principal axes (principal components) retained in the model.

#### GGE Biplot Analysis

The graphic method of GGE biplot was used in view of its simple visualization in experiments data analysis conducted in different environments. GGE biplot completed by “GGEBiplotGUI” package in R software (Frutos et al., 2014) was used to analyze the multi-environment trial data, and evaluate the adaptability and stability of the cultivars and the effects of genotype, environment, and G × E interaction. The general model of GGE biplot based on singular value decomposition (SVD) of environment-centered or environment-standardized could be written as:

$$Y_{ij}-\mu -\beta_{j}=\lambda_{1}\xi_{i1}\eta_{j1}+\lambda_{2}\xi_{i2}\eta_{j2}+\varepsilon_{ij}.$$

where *Y_ij_* was the measured mean of *i^th^* genotype in *j^th^* environment; *μ* was the grand mean; *β_j_* was the main effect of *j^th^* environment; *μ*+*β_j_* was the average trait over all genotypes in *j^th^* environment; *λ*_1_ and *λ*_2_ were the singular values for the first and second principal component (PC1 and PC2); *ξ_i_*_1_ and *ξ_i_*_2_ were eigenvectors of *i^th^* genotype for PC1 and PC2; *η_j_*_1_ and *η_j__2_* were eigenvectors of *j^th^* environment for PC1 and PC2; and *ε_ij_* was the residual of the model associated with *i^th^* genotype in *j^th^* environment.

### Genotyping and Quality Control

The natural population was sequenced by specific length amplified fragment sequencing (SLAF-seq) method. The double enzyme group comprising *Mse*I and *Hae*III (Thermo Fisher Scientific Inc., Waltham, MA, USA) was used to digest the soybean genomic DNA that was isolated from the fresh leaves of a single plant (Wu et al., 2010) into more than 50,000 sequencing tags, based on which, the sequencing libraries of each accession were constructed (Sun et al., 2013; Han et al., 2016). The Short Oligonucleotide Alignment Program 2 was used to map raw paired-end reads of the 45 bp sequence read at both ends of the sequencing tags for each library, which was obtained using the barcode approach combined with the Illumina Genome Analyzer II (Illumina Inc., San Diego, CA, USA) onto the reference genome (Li et al., 2009). Approximately 58,000 high-quality SLAF tags were obtained after sequencing reads with the same genomic position of each accession. A total of 34,710 single nucleotide polymorphism (SNP) loci with missing rate less than 10% and minor allele frequency (MAF) greater than 0.04 were used for PCA and kinship analysis by “scatterplot3d” (Glaab et al., 2010) and “gplots” packages in R, respectively.

## Results

### Genetic Variability, Correlation, and PCA

The natural population consisting of 173 soybean genotypes was grown in three experimental locations (Harbin, Changchun, and Shenyang) for 2 years (**Figure 1A**, **Table S1**, **Table S2**), and the phenotypic data were recorded. The population structure for 173 soybean genotypes obtained by SLAF-seq reflected the abundant genetic diversity of experimental materials (**Figure 1B**), and the heat map of the kinship matrix indicated the low level of relationship among the 173 individuals (**Figure 1C**). Different agronomic traits of 173 genotypes displayed high ranges of phenotypic variations (12.0~77.6%) (**Table S4**).

**Figure 2:**
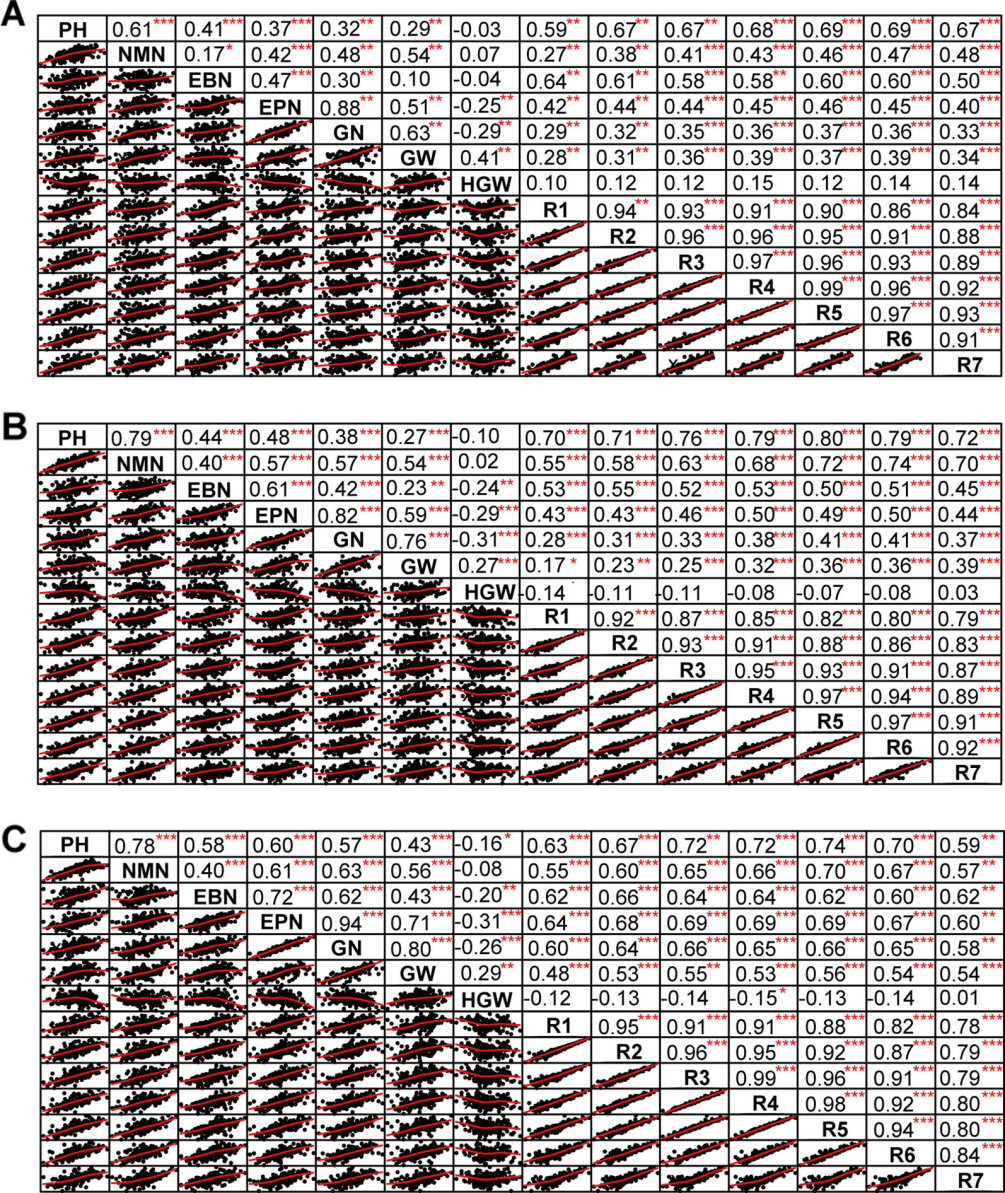
The correlation matrix of different traits. **(A)** Harbin. **(B)** Changchun. **(C)** Shenyang. The name of the trait was displayed on the diagonal line. The top right of the figure showed the correlation coefficient and the significance level, and the bottom left of the figure showed the bivariate scatterplot with the fitted line. PH, plant height; NMN, number of nodes of main stem; EBN, number of branch number per plant; EPN, number of pods per plant; GN, grain number per plant; GW, grain yield per plant; HGW, 100-grain weight; R1, beginning bloom; R2, full bloom; R3, beginning pod; R4, full pod; R5, beginning seed; R6, full seed; R7, beginning maturity. *** *P*<0.001, ** *P*<0.01, * *P*<0.05.

**Figure 3:**
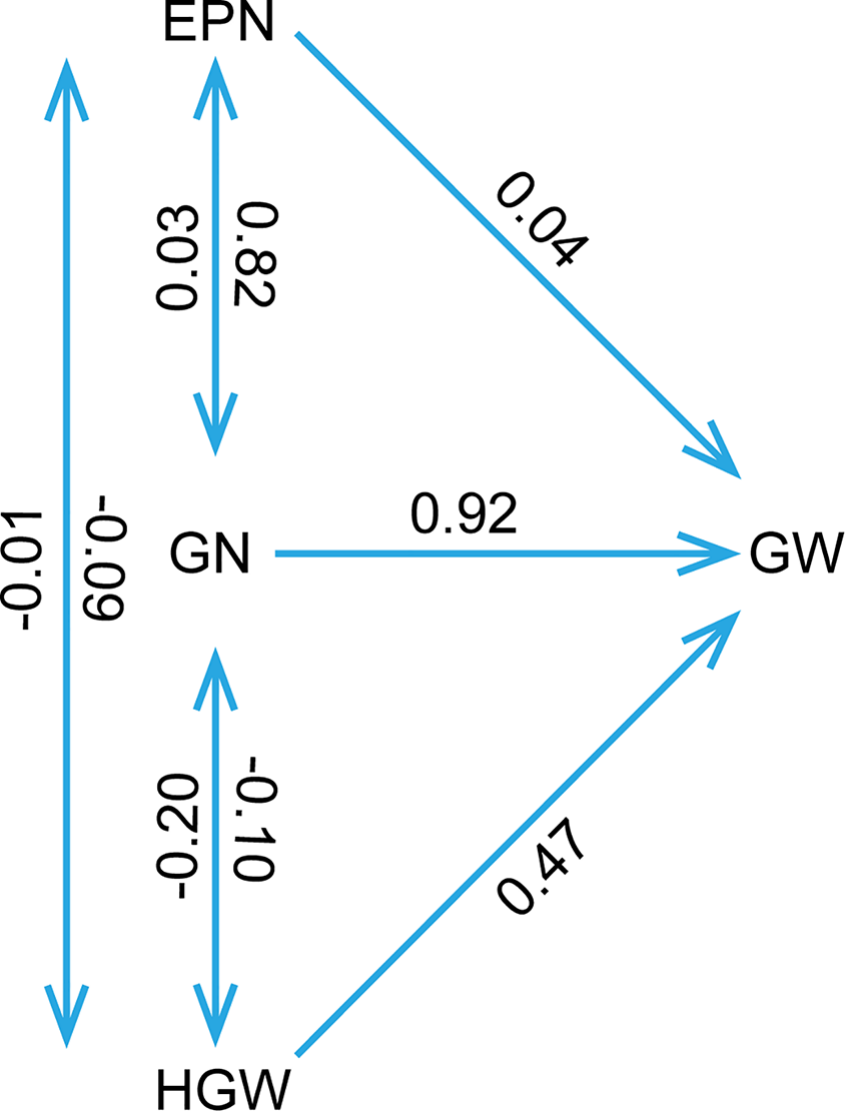
The path coefficient analysis. The double arrow lines represented the correlation among various variables, while the single arrow lines indicated the direct effects of variables to the soybean yield per plant as measured by path coefficient. The determination coefficient was 0.94, and the residual path coefficient was 0.25. EPN, number of pods per plant; GN, grain number per plant; GW, grain yield per plant; HGW, 100-grain weight.

The phenotypic data of different agronomic traits were transformed into standard normal random variable for statistical analysis (**Figure S1**). Pearson’s correlation studies (**Figure 2**) indicated that the yield showed very significant (*P*≤0.001, *P*≤0.01) or significant (*P*≤0.05) positive correlations with plant height, nodes of main stem, branches, pods, grains, 100-grain weight, and growth periods (R1–R7) (0.17<r<0.80) in at least two locations. Therefore, we should focus on selecting the soybean varieties with higher plant height, more nodes of main stem, branches, pods, and more grains, or longer growth periods R1–R7. Hundred-grain weight was negatively correlated (*P*≤0.001, *P*≤0.01) with number of branches per plant, number of pods per plant, and grain number per plant (−0.31<r<−0.24) in at least two locations. Path coefficient analysis showed that grains, 100-grain weight, and pods had positively direct effects on grain yield per plant (**Figure 3**, **Table S5**), implying that plant height, nodes of main stem, branches, and growth periods indirectly affected soybean yield by affecting grains, 100-grain weight, and pods.

**Figure 4:**
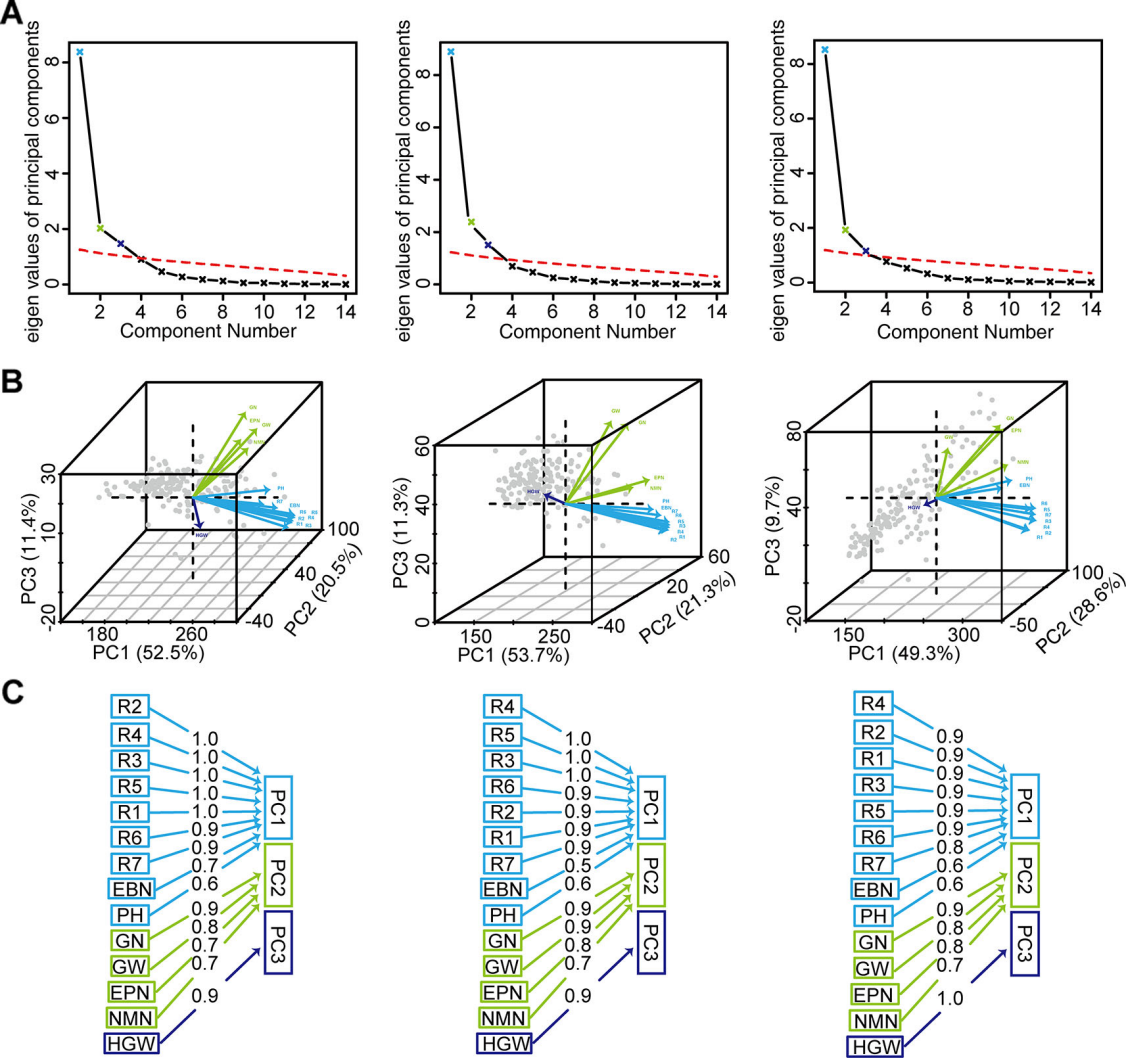
The PCA of grain weight per plant and the other 13 agronomic traits of 173 soybean genotypes. **(A)** Parallel analysis scree plots. **(B)** Variables factor map. **(C)** Components analysis. For **(A)**, **(B)**, and **(C)**, the figures from left to right represented the environment Harbin, Changchun, Shenyang, respectively. PH, plant height; NMN, number of nodes of main stem; EBN, number of branch number per plant; EPN, number of pods per plant; GN, grain number per plant; GW, grain yield per plant; HGW, 100-grain weight; R1, beginning bloom; R2, full bloom; R3, beginning pod; R4, full pod; R5, beginning seed; R6, full seed; R7, beginning maturity.

PCA was used to reduce the dimensions of 14 inter-correlated agronomic traits. The former three principal components with cumulative contribution rates 84.4~87.6% and the eigenvalue higher than 1 indicated that it comprehensively reflected the whole information (**Figures 4A**, **B**). PC1 indicated that plant height, number of branches per plant, and growth periods R1–R7 were important traits for classification. While the number of nodes of main stem per plant, the number of pods, the grains number, and the grain yield were important in PC2. In PC3, 100-grain weight was important (**Figures 4B**, **C**).

### Variance Analysis and Estimation of Broad-Sense Heritability

Combining ANOVA, we found that the factors genotype, genotype-by-year interaction, genotype-by-location interaction, and genotype-by-location-by-year interaction had significant effects (*P*<0.01) on the grain weight per plant and the other 13 agronomic traits (**Table S6**). Genotype had the greatest effect on total phenotypic variation of plant height, number of nodes of main stem, number of branches per plant, number of pods per plant, grain number per plant, grain yield per plant, and 100-grain weight accounting for 36.1~60.7%, followed by genotype-by-location interaction, accounting for 10.5~19.9%. R1–R7 were also mainly affected by genotype (61.0~69.3%), followed by trial location and genotype-by-location interaction (9.7~18.9%, 4.0~9.1%). The variance components of the AMMI model for grain weight per plant and the other 13 agronomic traits showed the same results (**Table S7**), suggesting that the different performance and response of genotypes across environments may be attributed to the location-dependent differences in soybean growth stages and maturing stages besides genotypes. The broad-sense heritability (*h^2^*) was usually used to determine whether the expression of plant traits was mainly influenced by heredity or environment. The *h^2^* values could be divided into three criteria, namely high *h^2^* greater than 50%, medium *h^2^* greater than 20% and less than 50%, and low *h^2^* less than 20% (Sulistyo et al., 2018). Grain weight per plant and the other 13 agronomic traits showed high heritability values (*h^2^*) (59.8~95.1%) (**Table S6**), reinforcing previous studies (Kuswantoro, 2019).

### The Relative Maturity of 173 Soybean Varieties in Various Environments

Because the phenotypic variation of R1–R7 caused by environmental variation were higher than that caused by G × E interaction, we speculated that the traits R1–R7 were more sensitive to the climate factors such as day length and temperature, thus affecting the adaptability of soybean varieties. In order to determine whether the introduced varieties were adapted to the local day length and temperature conditions more accurately and quickly, the mid-range values of R7 in 13 standard MGs were first calculated, and the averages of mid-range values for two adjacent MGs were used as the threshold (**Table S8**), and then the MGs of various varieties were determined (**Table S9**). Of these, 127 soybean varieties from Heilongjiang, Jilin, and Liaoning were mainly classified as MG000-III. Six, 15, and 8 varieties from Inner Mongolia, Beijing, and Hebei were mainly classified as MGs 00–0, MGs II–III, and MGs III–IV, respectively.

### The Identification of Ideal Location, Ecological Zoning, and Stable High-Yield Genotypes by GGE Biplot

**Figure 5:**
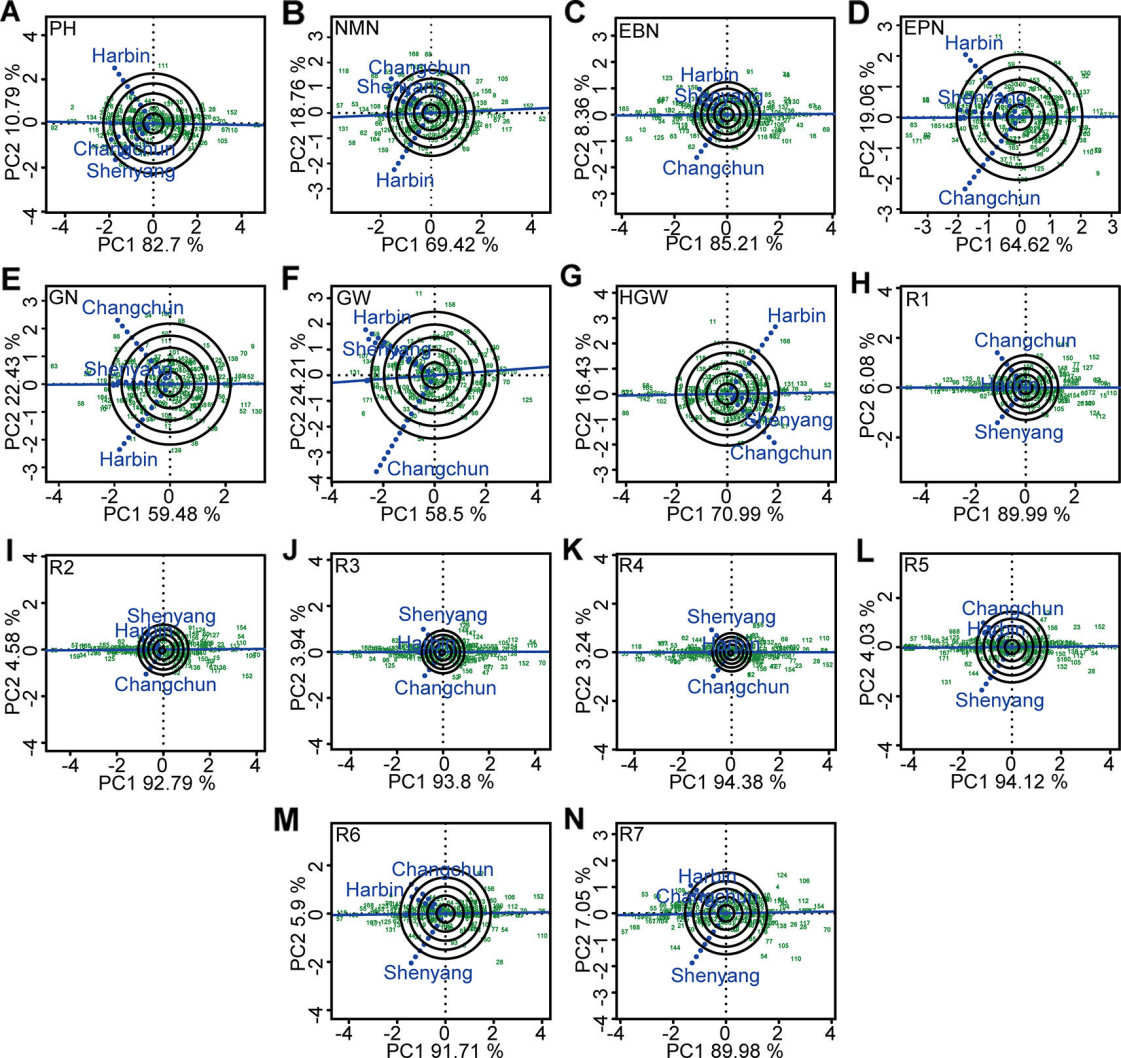
The representativeness and discrimination ability based on genotype plus genotype × environment interaction (GGE) biplot analysis. **(A)** PH, plant height. **(B)** NMN, numberof nodes of main stem. **(C)** EBN, number of branches per plant. **(D)** EPN, number of pods per plant. **(E)** GN, grain number per plant. **(F)** GW, grain yield per plant. **(G)** HGW, 100-grain weight. **(H)** R1, beginning bloom. **(I)** R2, full bloom. **(J)** R3, beginning pod. **(K)** R4, full pod. **(L)** R5, beginning seed. **(M)** R6, full seed. **(N)** R7, beginning maturity. The discrimination power of the experimental location was proportional to the length of the environment vector, which was the line connecting the origin and the testing environment point. The representativeness of target location was expressed by the angle between the testing location vector and the average environment coordination (AEC) horizontal axis (the single-arrowed line passing through biplot origin). The smaller the angle was, the stronger the representativeness of the location was. PH, plant height; NMN, number of nodes of main stem; EBN, number of branch number per plant; EPN, number of pods per plant; GN, grain number per plant; GW, grain yield per plant; HGW, 100-grain weight; R1, beginning bloom; R2, full bloom; R3, beginning pod; R4, full pod; R5, beginning seed; R6, full seed; R7, beginning maturity.

The significant influences of G × E interaction on different agronomic and physiological traits, such as plant height, number of nodes of main stem, number of branches, number of pods, grains number, 100-grain weight, growth periods, and grain yield per plant were the basis of mega-environment investigation. The GGE biplot explained 81.91~98.15% of total variations in the sum of squares for different agronomic traits. This excluded the false-positive from results (Alwala et al., 2010). The representativeness and discriminability of three testing environments on different agronomic traits was evaluated using GGE biplot to screen varieties with excellent and stable agronomic traits (**Figure 5**). Shenyang was the most discriminating and representative of the test environments for five agronomic traits such as plant height and R1–R4 (**Figures 5A**, **H–K**). Changchun was the ideal testing environment for five agronomic traits such as number of nodes of main stem, number of grains, 100-grain weight, R5, and R6 (**Figures 5B**, **E**, **G**, **L**, **M**). Harbin was the ideal testing environment for four agronomic traits such as number of branches, number of pods, grains yield per plant, and R7 (**Figures 5C**, **D**, **F**, **N**). In this sense, all three environments could be considered as specific selection environment for early screening of soybean genotypes that have different traits.

**Figure 6:**
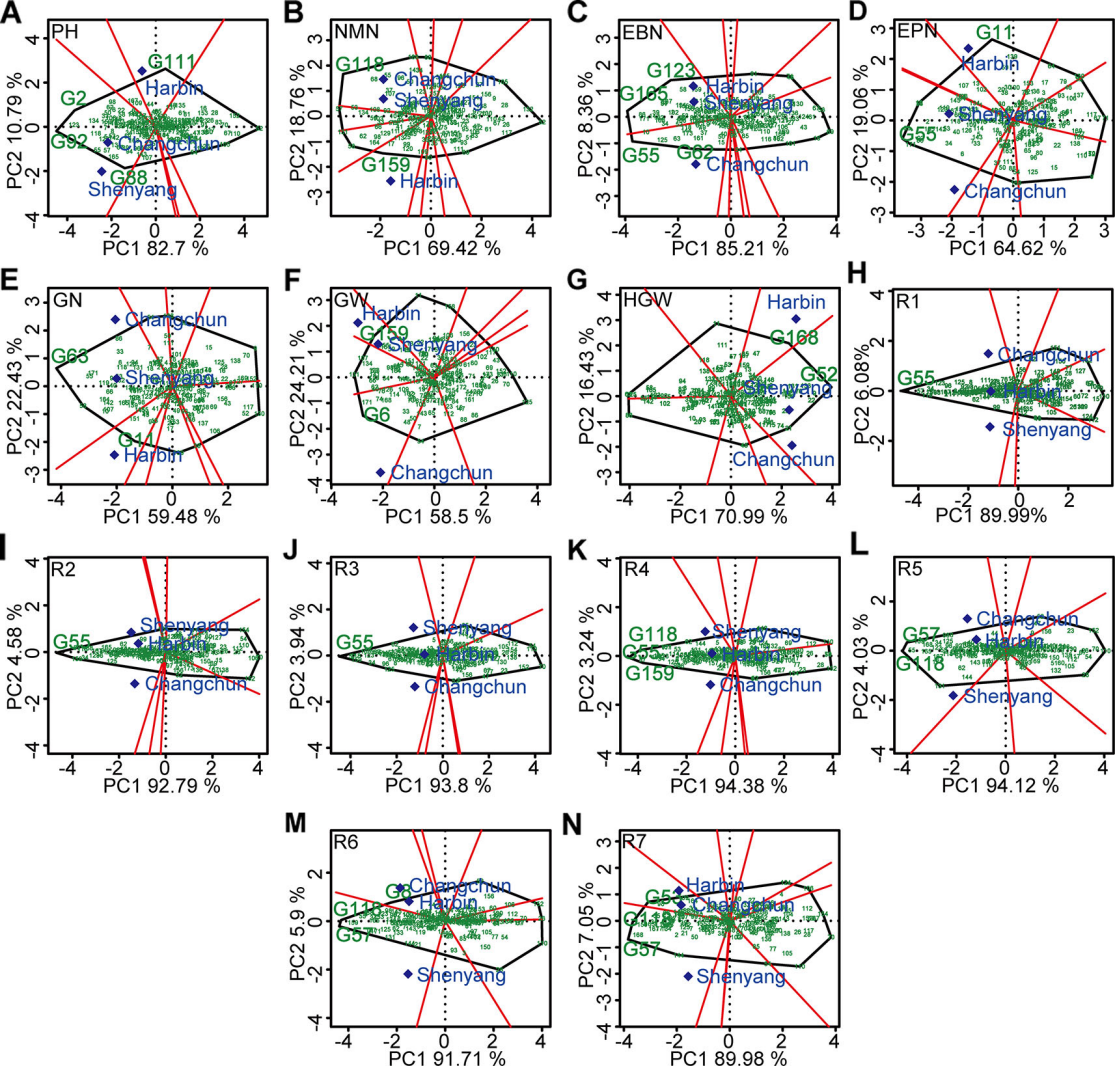
“Which-won-where” pattern based on GGE biplot analysis. **(A)** PH, plant height. **(B)** NMN, number of nodes of main stem. **(C)** EBN, number of branches per plant. **(D)** EPN, number of pods per plant. **(E)** GN, grain number per plant. **(F)** GW, grain yield per plant. **(G)** HGW, 100-grain weight. **(H)** R1, beginning bloom. **(I)** R2, full bloom. **(J)** R3, beginning pod. **(K)** R4, full pod. **(L)** R5, beginning seed. **(M)** R6, full seed. **(N)** R7, beginning maturity. The peripheral varieties were connected to form a polygon, which were divided into several sectors by perpendicular lines from origin to various edges. The varieties located at the vertex of the polygon were the ones with the best average performance in the experimental combinations within each fan-shaped region. G2, “Datunxiaoheidou.” G6, “Tiedou 58.” G8, “Jichanghuangdou 1.” G11, “Gongye 04L-141.” G52, “Liaoxian 1.” G53, “Zhongpin 95-5388.” G55, “L-59 Peking.” G57, “Ji 06B7.” G62, “Zhongzuo 00-683.” G63, “Dongnong 50.” G88, “Qinganheidou.” G92, “Heimoshidou.” G111, “Liaonong 2.” G118, “Qinganheidou.” G123, “Jinshanchamoshidou.” G159, “Hefeng 37.” G165, “Chamoshidou.” G168, “Wuxing 4.”. PH, plant height; NMN, number of nodes of main stem; EBN, number of branch number per plant; EPN, number of pods per plant; GN, grain number per plant; GW, grain yield per plant; HGW, 100-grain weight; R1, beginning bloom; R2, full bloom; R3, beginning pod; R4, full pod; R5, beginning seed; R6, full seed; R7, beginning maturity.

The polygon view (“which-won-where” pattern) of GGE biplot for different agronomic traits (**Figure 6**) indicated ecological zoning and the best genotypes for each mega-environment. By GGE biplot, mega-environment 1 consisting of Changchun and Shenyang was defined for plant height, number of nodes of main stem, number of pods, number of grains, and 100-grain weight. Three genotypes (“Datunxiaoheidou,” “Heimoshidou,” “Qinganheidou”), genotype “Qinganheidou,” genotype “L-59 Peking,” genotype “Dongnong 50,” and genotype “Liaoxian 1” were the best-performing genotype on plant height, number of nodes of main stem, number of pods, number of grains, and 100-grain weight in mega-environment 1, respectively (**Figures 6A**, **B**, **D**, **E**, **G**). Mega-environment 2 consisting of Harbin and Shenyang was defined for number of branches and grain yield, two genotypes (“Chamoshidou,” “Jinshanchamoshidou”) and genotype “Hefeng 37” showed the highest phenotypic value in mega-environment 2, respectively (**Figures 6C**, **F**). Mega-environment 3 consisting of Harbin, Changchun, and Shenyang was defined for R1–R5, genotype “L-59 Peking” was verified to be recognized as the winning genotype for R1–R3, three genotypes (“L-59 Peking,” “Hefeng 37,” and “Qinganheido”) and two genotypes (“Qinganheidou,” “Ji 06B7”) showed the highest phenotypic value for R4 and R5 in mega-environment 3, respectively (**Figure 6H–L**). Mega-environment 4 consisting of Harbin and Changchun was defined for R6 and R7, genotype “Jichanghuangdou 1” and genotype “Zhongpin 95-5388” showed the highest phenotypic value in mega-environment 4, respectively (**Figures 6M**, **N**). The rational region distribution of these cultivars should be that the best adaptive genotypes were planted in the most desirable environments to maximize the positive G × E interaction effects.

**Figure 7:**
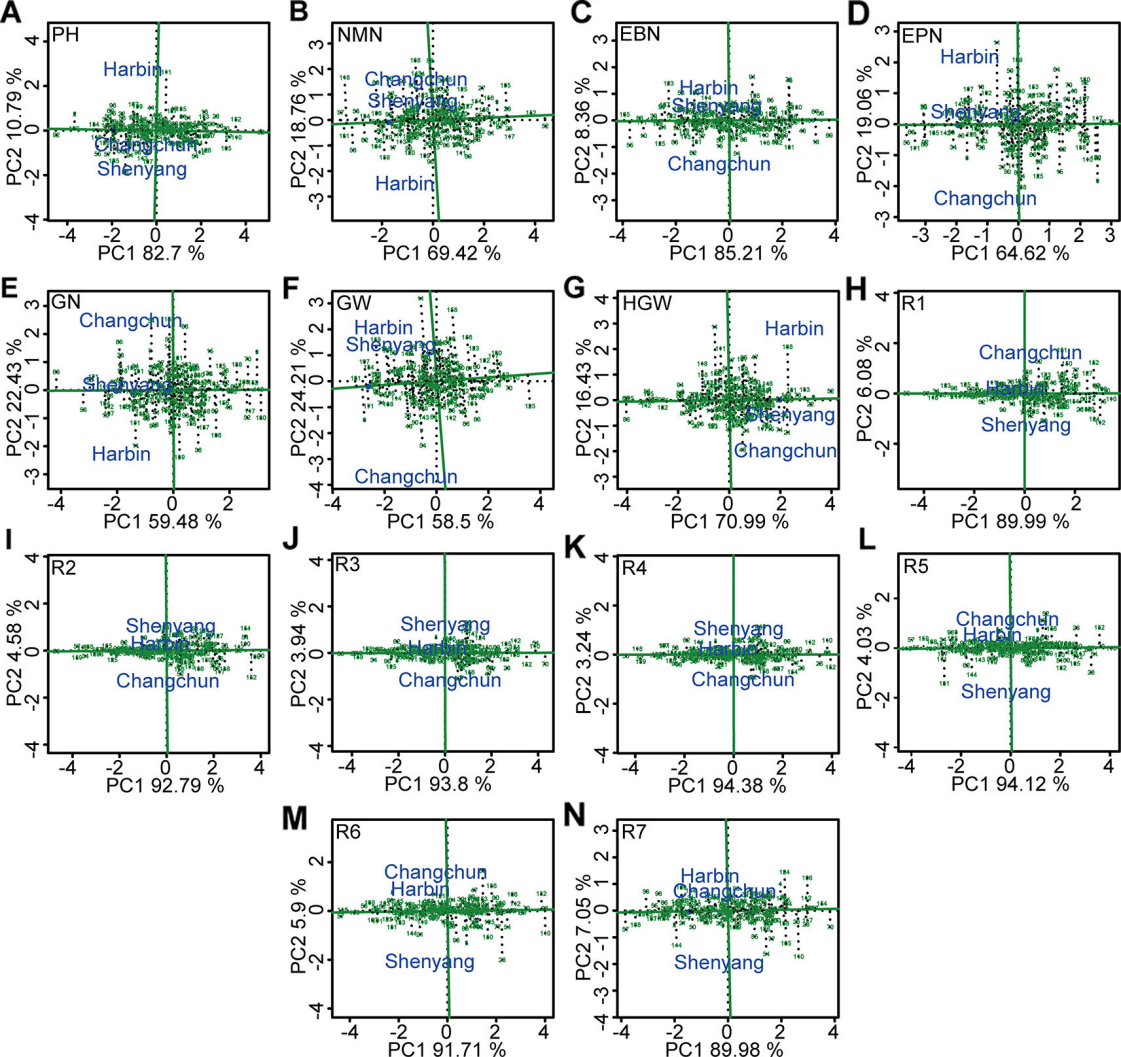
The stability and the mean performance of 173 genotypes for grain weight per plant and the other 13 agronomic traits and the specific genotype-by-environment interactions across environment based on GGE biplot analysis. **(A)** PH, plant height. **(B)** NMN, number of nodes of main stem. **(C)** EBN, number of branches per plant. **(D)** EPN, number of pods per plant. **(E)** GN, grain number per plant. **(F)** GW, grain yield per plant. **(G)** HGW, 100-grain weight. **(H)** R1, beginning bloom. **(I)** R2, full bloom. **(J)** R3, beginning pod. **(K)** R4, full pod. **(L)** R5, beginning seed. **(M)** R6, full seed. **(N )** R7, beginning maturity. The projection of soybean genotypes in the AEC horizontal axis was close to the positive direction, indicating the higher values of corresponding traits. The stability of each genotype was determined by the length of perpendicular line segments from soybean varieties to AEC, and the most stable genotype was almost located on the AEC horizontal axis and had a near-zero projection onto the AEC vertical axis. PH, plant height; NMN, number of nodes of main stem; EBN, number of branch number per plant; EPN, number of pods per plant; GN, grain number per plant; GW, grain yield per plant; HGW, 100-grain weight; R1, beginning bloom; R2, full bloom; R3, beginning pod; R4, full pod; R5, beginning seed; R6, full seed; R7, beginning maturity.

The soybean germplasm populations with stable above- or below-average of different agronomic traits were screened by GGE biplot (**Figure 7**, **Table S10**). Of these, genotypes “Heimoshidou,” “Jihuang 13,” “Zhongpin 03-5334,” “Datunxiaoheidou,” “Jidou 17,” “Suinong 1”, and “Longquandadou” were considered stable with the highest phenotypic value of plant height, number of nodes of main stem per plant, number of branches per plant, number of pods per plant, number of grains per plant, grain yield per plant, and 100-grain weight, respectively (**Figure 7**, **Table S11**). “L-59 Peking” with the highest value of growth periods R1–R4 was relatively stable genotype, “L-9” was relatively stable genotype with performance of the longest growth periods R5–R7 (**Figure 7**, **Table S11**). Conversely, genotypes “Liaoxian 1,” “Heihe 18,” “Beifeng 9,” “Suinong 29,” “Jilinchalihua,” “Bei 1484,” “Hujiao 04-528,” “Hujiao 03-286,” and “Dongnong 49” showed stable and the lowest phenotypic value of plant height, number of nodes of main stem per plant, number of branches per plant, number of pods per plant, 100-grain weight, R2, R3, R6, and R7 (**Figure 7**, **Table S11**). “Dongnong 44” was considered as genotype with both the lowest phenotypic value of number of grains per plant, grain yield per plant, R5, and stability performance (**Figure 7**, **Table S11**). “Huajiang 2” showed stable and the lowest phenotypic value of R1 and R4 (**Figure 7**, **Table S11**).

## Discussion

The improvement of soybean yield had become the problem that received the most attention in soybean production (Boerma and Specht, 2004). This study demonstrated that it was beneficial to cultivate soybeans with high yield by choosing higher plant height, more nodes of main stem, more branches, more pods, more grains, and longer growth periods, which was in accordance with previous research (Yadav et al., 2009; Luo, 2010; Aditya et al., 2011; Ngalamu et al., 2013; Islam and Rai, 2015; AbdulHamid et al., 2017; Nagarajan et al., 2017). However, the undesirable association of these yield-related traits was one of the most difficult challenges for crop breeders (Yan, 2014); for example, in this study we found that 100-grain weight was negatively correlated (*P*≤0.001, *P*≤0.01) with branches, pods, and grains (−0.31<r<−0.24). Thus, a realistic high-yield breeding strategy was to balance multiple traits at an acceptable level to achieve the high yields, instead of choosing a single trait. In addition, the previous reports have suggested that the genetic basis for improving the selection efficiency of soybean yield may be due to the direct selection of pods, grains, and 100-grain weight (Yadav et al., 2009; Wang et al., 2012). Furthermore, this study also inferred that the plant height, number of nodes of main stem, number of branches, and R1–R7 indirectly affected soybean yield by regulating pods, grains, and 100-grain weight. Therefore, when selecting high-yield soybean varieties, we should focus on plants with more pods, grains, and 100-grain weight, and then judged whether they have higher plant height, more nodes of main stem, more branches, and longer R1–R7. The high heritability values of different agronomic traits (59.8~95.1%) indicated that if they were selected by strict criteria in the early stages of breeding, the probability of obtaining offspring with excellent target traits was high (Song, 1999).

The interaction of genotype and environment could make the evaluation of genotypes complicated (Krishnamurthy et al., 2017); therefore, the significant contributions of G × E interaction to total variation of different agronomic traits were identified in this study. The significant effect of genotype-by-environment interaction on soybean yield was also confirmed by Junior et al. (2017). The genetic and environmental interactions for soybean yield per plant and other agronomic traits would be attributed to the predictable factors, such as soil type, pest, and disease management, and unpredictable factors in each environment, such as precipitation, temperature, and humidity (Aditya et al., 2011). The contributions of environment factor on the total phenotypic variations were greater than the G × E interaction effects for R1–R7. The short-day soybean growth period was controlled by photoperiod, which is an obstacle factor to enlarge the range of adaptation (Alliprandini et al., 2009). To better guide the breeding and planting practice of 173 soybean varieties, we classified the soybean varieties into different mature groups (MGs). After the soybeans were disseminated northward, the early MGs 000–0 varieties were developed in the Northeast of China covering a wide range of MGs 000–III (Liu et al., 2017). Likewise, 127 soybean varieties from Heilongjiang, Jilin, and Liaoning were mainly classified as MG000–III, which was consistent with MGs distribution in various soybean eco-regions in China by Gai et al. (Gai and Wang, 2001; Wu et al., 2012; Jia et al., 2014).

Estimates of decomposition of the complex G × E interaction greater than 50% represented a predominance of complex interactions (Sousa et al., 2015), indicating the changes in the magnitude of the differences among genotypes in different environments or from changes in their relative ranking (Sivcev et al., 2011). The major challenge of plant breeders was to find the useful information hidden within the multi-environment data, and then interpret and use it effectively. Harbin, Changchun, and Shenyang were the ideal locations with good discrimination power and representativeness for four traits (number of branches per plant, number of pods per plant, grain yield per plant, and R7), five traits (number of nodes of main stem, grain number per plant, 100-grain weight, R5, and R6), and five agronomic traits such as plant height and R1–R4, respectively. Based on the evaluation on the testing sites for mega-environment differentiation above, we could select the ideal candidate location to improve the efficiency and accuracy of cultivar selection and recommendation. The varieties selected from the ideal test location were most likely the ones with outstanding average performance and wider adaptability (Yan et al., 2000a).

Mega-environment concept was useful for optimum resource allocation in a breeding or cultivar evaluation program (Gauch and Zobel, 1997). Mega-environment was defined as a group of locations that consistently share the same best cultivars (Yan and Rajcan, 2002). The advantage of GGE biplot was evident when testing a large number of genotypes in mega-environment consisting of several environments, as the pattern of genotype-by-environment interaction could make the evaluation of genotypes complicated (Xu et al., 2013; Krishnamurthy et al., 2017). In other words, there was inconsistency in the superiority of the genotype with the environmental variation, which limited the indication of cultivars. The cultivar evaluation should be conducted specifically to each mega-environment prior to the cultivar recommendation due to the large effect of genotype by mega-environment interaction (Yan et al., 2011). The genotypes selected from one ecological region often performed well in the other ecological region of the same mega-environment; for example, genotype “Hefeng 37” showed the highest grain yield per plant in mega-environment 2 consisting of Harbin and Shenyang; this providing a theoretical basis for the introduction of cultivars.

In conclusion, because the region represented by the three locations might be considered as a complex mega-environment, a set of soybean genotypes based on the stability should be clarified. It was the most ideal way to “avoid” G × E interaction by identifying the stable genotypes for various traits (Yan and Kang, 2003). Genotype “Suinong 1” showed the highest and the most stable grain yield per plant across environments. The 173 individuals exhibited the low levels of kinship relatedness, of which, the stable varieties with extreme phenotype may be considered as crossbreeding parents to expand the genetic basis of improved soybean germplasm to produce greater super-parental effects. Simultaneously, soybean populations with the stable above-average or below-average traits were screened for more efficient molecular breeding.

Overall, the yield of soybean is a complex quantitative trait, which in our study was related to various traits such as plant morphology and growth period. Both high yield and stable yield were the main breeding objective, and the excellent soybean varieties should also be adapted to much wider ecological regions. First, the main purpose of our study was to identify and analyze soybean varieties with high-yield potential using GGE biplot, further used in the production of soybeans. Second, we analyzed the performance and stability of yield-related traits of different varieties, such as plant height, growth period, etc.; the stable varieties with extreme phenotype may be considered as crossbreeding parents to expand the genetic basis of improved soybean germplasm to produce greater super-parental effects. Third, soybean yield is controlled by multiple genes. In the early stage, our laboratory studied the 173 varieties at molecular level, such as SLAF-seq and genome-wide association study (GWAS) analysis, constructed high-density genetic maps, and explored genetic loci and candidate genes associated with multiple traits. However, whether the genes that regulate yield also regulate other traits, or whether the genes that regulate yield and those that regulate other traits are in a co-expression network, all need to be verified from different angles. As a result, we identified varieties with stable and excellent traits in different environments, providing us with accurate plant material for constructing genetic networks for different traits using next generation sequencing technology.

## Conclusion

The present investigation showed the significant genetic variability for different agronomic traits of 173 soybean genotypes in multi-location trials. The effects of G × E interaction on the phenotypic variation of all agronomic traits were significant, based on which, the ideal locations for various traits were further evaluated, the closeness of respective environments was depicted, and the stable genotypes with extreme phenotype, such as the genotypes with stable and high yield, were screened to provide a theoretical reference for the utilization and genetic improvement of soybean.

## Data Availability Statement

The raw data supporting the conclusions of this manuscript will be made available by the authors, without undue reservation, to any qualified researcher.

All datasets [generated/analyzed] for this study are included in the manuscript and the supplementary files

## Author Contributions

ML, YL, and CW performed the phenotype observations and measurements. XY, DL, and XZ performed the descriptive and correlation analysis. ML, CX, and XZ performed GGE biplot analysis. ML, WL, and LZ wrote the manuscript. All authors read, corrected, and approved the manuscript.

## Funding

This study was conducted in the Key Laboratory of Soybean Biology of Chinese Education Ministry, Key Laboratory of Biology, Genetics & Breeding for Soybean in Northeast China, Ministry of Agriculture, Soybean Research & Development Center, and financially supported by National Natural Science Foundation of China (31771820), Chinese Key Projects of Soybean Transformation (2016ZX08004-005), Key Special Project National Key Research & Development Program “seven crop breeding” (2016YFD0101005).

## Conflict of Interest

The authors declare that the research was conducted in the absence of any commercial or financial relationships that could be construed as a potential conflict of interest.
