# Supplementary material for: Identification of Traits Contributing to High and Stable Yields in Different Soybean Varieties Across Three Chinese Latitudes
Source: Front Plant Sci. 2020 Jan 21;10:1642. doi: 10.3389/fpls.2019.01642 (PMC6985368; doi:10.3389/fpls.2019.01642)
Supplement: Supplementary file 1 [file DataSheet_1.docx]

Supplementary Material

# Supplementary Figures and Tables

## Supplementary Figures


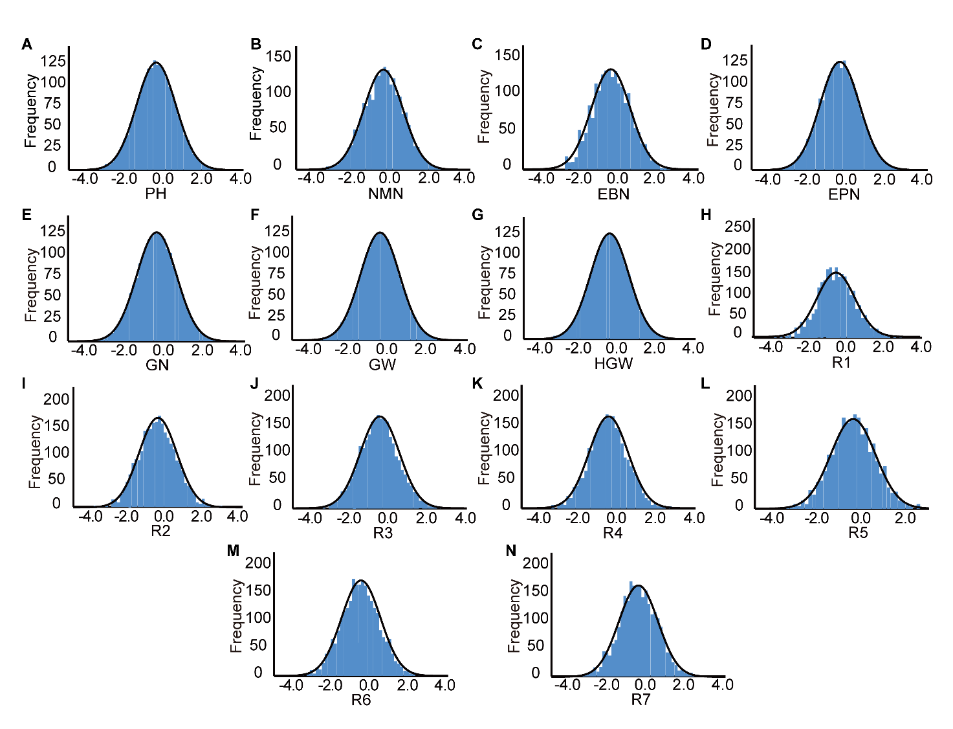


Figure S1. The normal score of standard normal random variable transformed from the phenotypic data of grain weight per plant and the other thirteen agronomic traits for 173 soybean genotypes. The abscissa axis represented the normal score, and the ordinate axis represented the frequency. PH: plant height, NMN: number of nodes of main stem, EBN: number of branch number per plant, EPN: number of pods per plant, GN: grain number per plant, GW: grain yield per plant, HGW: 100-grain weight, R1: beginning bloom, R2: full bloom, R3: beginning pod, R4: full pod, R5: beginning seed, R6: full seed, R7: beginning maturity.

## Supplementary Tables

**Supplementary Table 1.** Ecological information of the three test locations.

| **Location** | **Longitude (E)** | **Latitude (N)** | **Soil type** | **Precipitation (mm)** | **Mean daylength (h) from May 1 to October 1 in 2015 and 2016** | **Mean daily temperature (°C) from May 1 to October 1 in 2015 and 2016** | |
| --- | --- | --- | --- | --- | --- | --- | --- |
|  |  |  |  |  |  | **Highest** | **Lowest** |
| Harbin | 126°63′ | 45°75′ | Black earth | 517 | 14.5 | 24.9 | 15.1 |
| Changchun | 125°35 ′ | 43°88′ | Black earth | 566 | 14.35 | 25.5 | 15.2 |
| Shenyang | 122°25′ | 41°11′ | Brown soil | 663 | 14.18 | 27.1 | 15.8 |

Black soil: The average content of organic substances is 3% to 10%, the C/N ratio is 10-14; the clay content is >35%; the cation exchange capacity is large (25-80% by mole (+) per kilogram of soil), and the exchangeable base ( In particular, the content of Ca2+ and Mg2+) is also high, the salt base saturation is more than 50%, and increases with depth; the pH value is more than 6.0 to 8.5.

Brown soil: It is mainly distributed in the warm temperate Liaodong Peninsula and Shandong Peninsula. It is a neutral to slightly acidic soil developed under the summer green broad-leaved forest or coniferous and broad-leaved mixed forest. It is characterized by a brown depositional viscous layer below the humus layer. The mineral weathering degree is not high, the silica/alumina ratio is about 3.0, the clay mineral is mainly composed of hydromica and vermiculite, and there are a small amount of kaolinite and montmorillonite, and the salt base is close to saturation.

**Supplementary Table 2.** General information and phenotypic data of 173 test soybean accessions.

| **Number** | **Accession** | **Origin** | **Latitude (°N)** | **Longitude (°W)** | **2015 Harbin** | | | | | | | | | | | | | |
| --- | --- | --- | --- | --- | --- | --- | --- | --- | --- | --- | --- | --- | --- | --- | --- | --- | --- | --- |
|  |  |  |  |  | **PH**  **(cm)**  **()** | **NMN** | **EBN** | **EPN** | **GN** | **GW**  **(g)** | **HGW**  **(g)** | **R1**  **(day)** | **R2**  **(day)** | **R3**  **(day)** | **R4**  **(day)** | **R5**  **(day)** | **R6**  **(day)** | **R7**  **(day)** |
| G1 | Tiedou 50 | Liaoning,China | 40.8 | 122 | 89.9 | 18.5 | 3 | 48.2 | 89 | 17.9 | 18.1 | 54.6 | 65.4 | 76.6 | 83.6 | 89.8 | 97 | 120.4 |
| G2 | Datun xiaoheidou | Hebei,China | 39.2 | 116.3 | 117.5 | 16.1 | 5.7 | 93.9 | 100.3 | 11.4 | 11.1 | 69 | 75 | 79.9 | 86.9 | 93.8 | 101 | 123.7 |
| G3 | Ken 04-8579 | Heilongjiang,China | 48.3 | 128.1 | 70.9 | 17.1 | 0.7 | 39.5 | 99.3 | 16.1 | 15.8 | 37.8 | 47.5 | 55.2 | 64.1 | 71.1 | 78.3 | 108.1 |
| G4 | Kangxian2 | Heilongjiang,China | 48.3 | 128.1 | 103.2 | 16.6 | 1.7 | 45.6 | 94.2 | 14 | 14.9 | 37.8 | 53 | 58.8 | 69.6 | 79 | 85.7 | 112.7 |
| G5 | Liushitianhuancang | Liaoning,China | 40.8 | 122 | 90.8 | 15.6 | 2.4 | 50.6 | 92.5 | 15.6 | 14.9 | 33.4 | 53.3 | 60.3 | 79 | 85 | 91 | 118 |
| G6 | Tiedou 58 | Liaoning,China | 40.8 | 122 | 96.7 | 18.9 | 3.3 | 69.8 | 155.7 | 34.4 | 22.5 | 64.3 | 70.3 | 79.4 | 87.5 | 93.5 | 98.9 | 123.4 |
| G7 | Zhonghuang 20 | Beijing,China | 40.1 | 116.3 | 89.6 | 15.7 | 2.9 | 63.4 | 88.8 | 17.5 | 19.8 | 40.8 | 57.9 | 76.7 | 83.7 | 90.6 | 96.7 | 124 |
| G8 | Jichanghuangdou 1 | Xinjiang,China | 41.2 | 81.4 | 96.9 | 17.1 | 3.1 | 53.6 | 90.8 | 15.4 | 16.8 | 60.9 | 71.9 | 80.9 | 87.9 | 93.9 | 101.9 | 124 |
| G9 | Longquandadou | Heilongjiang,China | 48.3 | 128.1 | 73.4 | 14 | 1.6 | 19 | 27.6 | 6.9 | 25 | 42.5 | 47.4 | 59 | 66.6 | 71.6 | 78.6 | 108.6 |
| G10 | Xiaobaiqi | Liaoning,China | 40.8 | 122 | 102.6 | 18.4 | 5.8 | 92.4 | 139.6 | 20.1 | 14.4 | 60.1 | 69.1 | 79 | 86 | 93 | 100 | 124 |
| G11 | Gongye 04L-141 | Jilin,China | 43.4 | 126.3 | 92 | 18 | 1.2 | 51.4 | 121.1 | 22.2 | 28 | 37.5 | 67.1 | 67.9 | 83.9 | 90.3 | 96.3 | 114.2 |
| G12 | Tiedou 52 | Liaoning,China | 40.8 | 122 | 89.4 | 17.4 | 1.3 | 46.7 | 105 | 24 | 22.7 | 32 | 52.8 | 58.9 | 73.6 | 83.8 | 90.8 | 121.8 |
| G13 | L-57 | America | 43.4 | 79.3 | 98.6 | 16.5 | 2.8 | 46 | 87.6 | 17.9 | 20.4 | 39 | 52.8 | 59.9 | 77.4 | 83.4 | 89.4 | 119.4 |
| G14 | He 05-31 | Heilongjiang,China | 48.3 | 128.1 | 77.5 | 14.8 | 0.5 | 38.7 | 62.2 | 8.8 | 14.1 | 37.7 | 52.4 | 57.7 | 67.5 | 76.5 | 82.5 | 112.5 |
| G15 | L-21 | Canada | 43.4 | 79.3 | 81.2 | 18 | 1.3 | 30.9 | 60.3 | 10.3 | 17.1 | 28 | 38.5 | 50.6 | 55.5 | 65.5 | 77.3 | 101.4 |
| G16 | Hongfeng11 | Heilongjiang,China | 48.3 | 128.1 | 76.1 | 17.4 | 0.1 | 41.2 | 70.3 | 13.3 | 18.9 | 34.2 | 52.3 | 56.4 | 67.4 | 72.7 | 78.7 | 107.7 |
| G17 | Heinong 33 | Heilongjiang,China | 48.3 | 128.1 | 103 | 22.3 | 1.4 | 50 | 125.6 | 23.6 | 18.9 | 31.8 | 46.4 | 55.6 | 64.6 | 73.6 | 81.6 | 112.6 |
| G18 | Liao 98072 | Liaoning,China | 40.8 | 122 | 93.7 | 17.6 | 2.3 | 44.1 | 81.5 | 15.3 | 18.7 | 52.1 | 55.3 | 66.3 | 79.3 | 85.3 | 91.3 | 122.3 |
| G19 | Kenfeng 22 | Heilongjiang,China | 48.3 | 128.1 | 73.9 | 16.9 | 0.5 | 33.8 | 66.3 | 11.6 | 17.6 | 32.1 | 44.7 | 56.8 | 64.6 | 71.6 | 81.6 | 112.6 |
| G20 | Jiyu 89 | Jilin,China | 43.4 | 126.3 | 89.8 | 16 | 2.4 | 64.7 | 108.7 | 14 | 12.6 | 37.5 | 47.8 | 52.6 | 62.5 | 77.8 | 83.8 | 114.8 |
| G21 | Tiedou 51 | Liaoning,China | 40.8 | 122 | 85.8 | 16.8 | 2.6 | 36.6 | 71.8 | 15.3 | 21.2 | 38.7 | 50.3 | 60.8 | 71.8 | 80.8 | 88.8 | 119.8 |
| G22 | Tongnong 13 | Jilin,China | 43.4 | 126.3 | 102.6 | 17.2 | 1 | 27.1 | 52.2 | 14.9 | 28.3 | 44.8 | 58.7 | 68.9 | 77.9 | 83.9 | 89.9 | 119.9 |
| G23 | Bei 1873 | Heilongjiang,China | 48.3 | 128.1 | 81.7 | 16.5 | 0.3 | 31.8 | 53.2 | 11.2 | 21.1 | 28.4 | 37.3 | 46.4 | 53.7 | 61.6 | 69.7 | 96.7 |
| G24 | Hefeng 50 | Heilongjiang,China | 48.3 | 128.1 | 72.9 | 15.7 | 0.1 | 37.4 | 68.8 | 12.6 | 18.4 | 30.8 | 45.2 | 51.1 | 59.4 | 68.4 | 79.4 | 109.4 |
| G25 | Sui 02-339 | Heilongjiang,China | 48.3 | 128.1 | 87.3 | 18 | 2.2 | 29.6 | 61.2 | 17.2 | 24 | 38.5 | 53.8 | 59.7 | 69.7 | 78.7 | 84.7 | 115.7 |
| G26 | Hujiao 03-286 | Inner Mongolia,China | 44.5 | 111.7 | 74.7 | 15 | 2.7 | 78.4 | 102.8 | 13.6 | 21.2 | 30.9 | 39.5 | 51.5 | 56.2 | 61.3 | 69.3 | 99.3 |
| G27 | Heinong 44 | Heilongjiang,China | 48.3 | 128.1 | 78.7 | 14.3 | 0 | 44.2 | 102 | 18.6 | 18.3 | 34.2 | 45.4 | 55.4 | 66.1 | 73.4 | 80.4 | 111.4 |
| G28 | Hujiao 04-528 | Inner Mongolia,China | 44.5 | 111.7 | 88.4 | 16 | 2.9 | 51.1 | 53.1 | 9.8 | 18.4 | 29.6 | 37.5 | 49.2 | 53.8 | 60.8 | 69.8 | 97.8 |
| G29 | Fengshou 10 | Heilongjiang,China | 48.3 | 128.1 | 76.9 | 18 | 1.7 | 37.9 | 89.6 | 17.6 | 19.6 | 34.7 | 52.2 | 57.4 | 68 | 76.9 | 82.9 | 107.9 |
| G30 | Hersong 2 | Ukraine | 50.3 | 30.3 | 88.4 | 16 | 2.9 | 51.1 | 105.4 | 21.3 | 20.2 | 56.1 | 59.6 | 70.6 | 78.6 | 83.6 | 88.6 | 118.6 |
| G31 | Jinzhou 4-1 | Liaoning,China | 40.8 | 122 | 82.3 | 18.1 | 1.8 | 42.4 | 90.4 | 16.4 | 18.2 | 60.2 | 70.1 | 78.1 | 83.1 | 88.1 | 93.1 | 124.1 |
| G32 | Suinong 14 | Heilongjiang,China | 48.3 | 128.1 | 53.2 | 12.7 | 0.3 | 40.3 | 82.4 | 16 | 19.4 | 33.2 | 49.9 | 56.9 | 66.3 | 74.2 | 80.2 | 109.2 |
| G33 | Tiejiasilihuang | Jilin,China | 43.4 | 126.3 | 108.2 | 18.6 | 3.2 | 46.7 | 92 | 14.9 | 16.5 | 49.9 | 56.2 | 69.2 | 80.2 | 87.2 | 94.2 | 118.1 |
| G34 | Tiefeng 8 | Liaoning,China | 40.8 | 122 | 80.1 | 14.3 | 3.8 | 61.8 | 99.8 | 18.8 | 18.8 | 73.2 | 79.2 | 85.2 | 92.2 | 99.2 | 105.2 | 125 |
| G35 | Silihuang | Jilin,China | 43.4 | 126.3 | 84.4 | 15.8 | 1.6 | 39.8 | 78.2 | 15 | 20.2 | 39.4 | 55.6 | 60.5 | 69.7 | 78.7 | 84.7 | 115.7 |
| G36 | Bei 1361 | Heilongjiang,China | 48.3 | 128.1 | 77.9 | 19.3 | 0.6 | 37.8 | 82.6 | 16.5 | 20 | 33.5 | 48.7 | 55.9 | 64.9 | 71.9 | 82.6 | 110.6 |
| G37 | L-5 | Canada | 43.4 | 79.3 | 96.5 | 16.8 | 2.1 | 42.4 | 93.7 | 14 | 14.6 | 48 | 54.5 | 64.1 | 78.1 | 86.1 | 93.1 | 117.1 |
| G38 | Maoyandou | Gansu, China | 32.3 | 92.1 | 88.8 | 16.8 | 3.7 | 63.8 | 118.1 | 26.1 | 22.1 | 49.7 | 59.7 | 67.7 | 79.7 | 84.7 | 89.7 | 115.7 |
| G39 | Fengdihuang | Jilin,China | 43.4 | 126.3 | 90 | 13.1 | 5.1 | 39 | 66.1 | 12.3 | 18.5 | 54.8 | 67.8 | 77.8 | 83.8 | 90.8 | 97.8 | 125 |
| G40 | Tiedou 54 | Liaoning,China | 40.8 | 122 | 91.6 | 18 | 1.2 | 63.6 | 141.2 | 28.1 | 19.9 | 62.3 | 69.2 | 79.2 | 86.2 | 93.2 | 100.2 | 124.8 |
| G41 | Jiunong 20 | Jilin,China | 43.4 | 126.3 | 84 | 18.8 | 1.4 | 46.3 | 119.1 | 18.6 | 15.9 | 42.5 | 55.5 | 69.6 | 80.6 | 86.6 | 92.6 | 118.6 |
| G42 | Duludou | Jilin,China | 43.4 | 126.3 | 82.4 | 16.3 | 1.8 | 38.9 | 72.9 | 13.3 | 18.2 | 32.5 | 45.7 | 55.5 | 64.5 | 70.7 | 77.8 | 108.7 |
| G43 | Sui 03-3046 | Heilongjiang,China | 48.3 | 128.1 | 66 | 13.2 | 0 | 44.4 | 84 | 13 | 15.4 | 31.7 | 43.1 | 53.7 | 59.5 | 65.6 | 74.5 | 101.7 |
| G44 | Hefeng 47 | Heilongjiang,China | 48.3 | 128.1 | 90.6 | 15.8 | 0.4 | 47.6 | 93.2 | 20.1 | 21.6 | 50.5 | 61.1 | 73.3 | 79.3 | 85.3 | 91.3 | 118.3 |
| G45 | V111-4 | Jilin,China | 43.4 | 126.3 | 91.8 | 19.4 | 0.6 | 49.7 | 155.7 | 32.1 | 20.6 | 31.1 | 54.5 | 62.8 | 70.5 | 79.5 | 85.5 | 113.5 |
| G46 | Longxuan 1 | Heilongjiang,China | 48.3 | 128.1 | 63.9 | 16 | 0.9 | 44.3 | 96.8 | 17.5 | 18.1 | 31.3 | 47 | 53.3 | 58.3 | 67.8 | 74.8 | 105.8 |
| G47 | Dongnong 8004 | Heilongjiang,China | 48.3 | 128.1 | 78.4 | 15.7 | 0.3 | 44.3 | 93.4 | 17.9 | 28.7 | 39 | 52 | 54.9 | 63.9 | 71.1 | 79.3 | 110.3 |
| G48 | Sui 03-3952 | Heilongjiang,China | 48.3 | 128.1 | 100.6 | 15.4 | 0 | 28.6 | 55.3 | 13.5 | 14.4 | 39.3 | 54 | 60.7 | 70 | 77.1 | 83.1 | 114.1 |
| G49 | Hefeng 25 | Heilongjiang,China | 48.3 | 128.1 | 78 | 16.1 | 2.9 | 46.6 | 93.4 | 16 | 17.1 | 39.8 | 55.7 | 60.8 | 66.4 | 74.5 | 81.5 | 111.5 |
| G50 | Liaodou 3 | Liaoning,China | 40.8 | 122 | 107.2 | 15 | 1.5 | 35.7 | 63.7 | 15.1 | 23.7 | 46.8 | 57.4 | 63.2 | 69.6 | 79.6 | 86.6 | 116.6 |
| G51 | Hujiao 423 | Inner Mongolia,China | 44.5 | 111.7 | 68.1 | 15.5 | 1.7 | 41.9 | 115.1 | 17.8 | 15.5 | 30.2 | 37.7 | 50.6 | 57.7 | 62.7 | 71.4 | 103.4 |
| G52 | Liaoxian 1 | Liaoning,China | 40.8 | 122 | 25.8 | 11.8 | 3.4 | 47.7 | 76.8 | 27.4 | 35.7 | 39.2 | 49.7 | 59.7 | 67.4 | 70.5 | 78.4 | 112.4 |
| G53 | Zhongpin 95-5388 | Beijing,China | 40.1 | 116.3 | 96 | 18.2 | 4.3 | 32.9 | 60.7 | 11.8 | 19.5 | 58.3 | 70 | 79 | 86 | 93 | 100 | 125 |
| G54 | Heihe 45 | Heilongjiang,China | 48.3 | 128.1 | 58.3 | 15.1 | 0.3 | 32.8 | 67.3 | 14.2 | 21.6 | 28 | 37 | 48.5 | 56.7 | 62.6 | 68.5 | 96.5 |
| G55 | L-59 Peking | America | 41.8 | 92.9 | 94.7 | 15.8 | 4.2 | 71.2 | 137 | 11.6 | 8.5 | 78.6 | 81.8 | 87.8 | 93.8 | 99.8 | 105.8 | 125 |
| G56 | Sui 04-5804 | Heilongjiang,China | 48.3 | 128.1 | 83 | 18.6 | 0 | 41 | 95.1 | 17.7 | 18.6 | 31.9 | 48.8 | 57.3 | 63.3 | 68.4 | 73.4 | 106.4 |
| G57 | Ji 06B7 | Hebei,China | 39.2 | 116.3 | 89 | 21.4 | 8.2 | 64.8 | 74 | 16.8 | 22.7 | 73 | 81 | 86 | 93 | 100 | 107 | 125 |
| G58 | Marca. Joe lisa | Heilongjiang,China | 48.3 | 128.1 | 92.8 | 22.9 | 4.9 | 89.2 | 148.9 | 17.8 | 11.9 | 66.9 | 71.1 | 78.1 | 84.1 | 91.1 | 98.1 | 124 |
| G59 | Jihuang 13 | Hebei,China | 39.2 | 116.3 | 96 | 19.9 | 3.4 | 55.5 | 98.8 | 20 | 19.3 | 57.9 | 73.9 | 78.9 | 85.9 | 92.9 | 99.9 | 125 |
| G60 | L-28 | Canada | 43.4 | 79.3 | 92.3 | 16.3 | 1.9 | 52.6 | 64.4 | 11.3 | 17.6 | 27.5 | 45.9 | 50.4 | 56.5 | 65.5 | 73.5 | 106.5 |
| G61 | Beifeng 9 | Heilongjiang,China | 48.3 | 128.1 | 84.5 | 14.3 | 0 | 33.7 | 89.3 | 14.9 | 17.3 | 30.5 | 47.9 | 56.3 | 62.8 | 68.5 | 74.5 | 107.6 |
| G62 | Zhongzuo 00-683 | Beijing,China | 40.1 | 116.3 | 108.1 | 21.4 | 3.6 | 64.9 | 136.1 | 25.5 | 19.1 | 57.3 | 68.1 | 78.1 | 84.1 | 89.1 | 94.1 | 123.1 |
| G63 | Dongnong 50 | Heilongjiang,China | 48.3 | 128.1 | 79.4 | 17.1 | 1.7 | 51.2 | 111.6 | 8 | 7.2 | 35.6 | 56.5 | 61.4 | 68 | 73.2 | 79.2 | 111.2 |
| G64 | Dongnong 93-046 | Heilongjiang,China | 48.3 | 128.1 | 98 | 19.9 | 1.5 | 46.3 | 83.5 | 18.6 | 25 | 34.1 | 52.4 | 59.2 | 69.1 | 75.1 | 82.1 | 115.1 |
| G65 | Suinong 10 | Heilongjiang,China | 48.3 | 128.1 | 98 | 19.9 | 1.5 | 46.3 | 83.5 | 18.6 | 17.3 | 32.8 | 39 | 51.8 | 63.9 | 69 | 77 | 109 |
| G66 | Tiefeng 31 | Liaoning,China | 40.8 | 122 | 88.2 | 19.5 | 3.4 | 64.9 | 142 | 25.6 | 18 | 44.8 | 54.9 | 72.4 | 79.4 | 86.4 | 93.4 | 123.1 |
| G67 | Dongnong 49 | Heilongjiang,China | 48.3 | 128.1 | 53.6 | 13.7 | 0.1 | 24.4 | 55.5 | 8.7 | 15.6 | 28.5 | 37.6 | 53.8 | 57.5 | 63.5 | 71.1 | 93.1 |
| G68 | Huayou 446 | Hebei,China | 39.2 | 116.3 | 92.4 | 18 | 1.7 | 30.2 | 69 | 14.3 | 20.7 | 54.8 | 67.6 | 74.4 | 82.4 | 89.4 | 96.4 | 124 |
| G69 | Heihe 48 | Heilongjiang,China | 48.3 | 128.1 | 71 | 17.6 | 0 | 46.8 | 107 | 17.3 | 16.2 | 30.7 | 49 | 53 | 61.9 | 67 | 75.1 | 101.1 |
| G70 | Huajiang 2 | Heilongjiang,China | 48.3 | 128.1 | 59.4 | 13.8 | 0.2 | 23.8 | 48.5 | 8.1 | 16.8 | 27.2 | 37.6 | 45.3 | 54.9 | 60.8 | 67.8 | 93.8 |
| G71 | Suinong 29 | Heilongjiang,China | 48.3 | 128.1 | 69.9 | 16.1 | 1.4 | 39.3 | 87.8 | 15.9 | 18.2 | 33.9 | 49.2 | 57.5 | 65.1 | 69.9 | 77.9 | 107.9 |
| G72 | Hefeng 35 | Heilongjiang,China | 48.3 | 128.1 | 63.7 | 15.6 | 1 | 46.7 | 95.3 | 18.2 | 19.3 | 34.1 | 49.4 | 56.6 | 62.5 | 68.6 | 74.6 | 108.6 |
| G73 | Suinong 8 | Heilongjiang,China | 48.3 | 128.1 | 98.9 | 19.2 | 1.5 | 39.7 | 87.4 | 22.3 | 22.2 | 30.9 | 49.2 | 57.4 | 63.5 | 74.3 | 76.1 | 108.2 |
| G74 | Jilin 47 | Jilin,China | 43.4 | 126.3 | 84.3 | 18.5 | 1.2 | 71.3 | 141.9 | 26.9 | 18.6 | 48 | 57.5 | 67.7 | 71 | 76.8 | 83 | 115 |
| G75 | Heinong 48 | Heilongjiang,China | 48.3 | 128.1 | 82.6 | 16.3 | 0.1 | 42.3 | 76.1 | 17.8 | 21.4 | 35.7 | 49.6 | 57.5 | 64.3 | 69.7 | 76.7 | 109.7 |
| G76 | Nova | Italy | 41.5 | 12.3 | 105.8 | 19 | 2 | 48.2 | 78.9 | 22.7 | 28.6 | 46.3 | 57.9 | 66.7 | 73.6 | 81 | 89 | 121 |
| G77 | Beidou 14 | Heilongjiang,China | 48.3 | 128.1 | 70.8 | 16.4 | 0.1 | 51.4 | 101.5 | 19.4 | 19.1 | 29.1 | 40 | 50 | 55.2 | 60.1 | 72.1 | 104.1 |
| G78 | Kangxian 3 | Heilongjiang,China | 48.3 | 128.1 | 102.3 | 18.8 | 2.5 | 36.3 | 62.3 | 12.2 | 19.5 | 37.4 | 55.5 | 62.5 | 68.8 | 74.8 | 82.8 | 112.8 |
| G79 | Dongnong 42 | Heilongjiang,China | 48.3 | 128.1 | 92.7 | 15.3 | 0.6 | 30.4 | 62.7 | 15.8 | 25.4 | 30.8 | 48.5 | 58.4 | 67.4 | 72.3 | 79.3 | 110.3 |
| G80 | Kenjian 23 | Heilongjiang,China | 48.3 | 128.1 | 67.2 | 14 | 0 | 27.6 | 63.6 | 11.2 | 17.6 | 33.6 | 49.2 | 56 | 60.6 | 67.4 | 74.1 | 105.1 |
| G81 | Heinong 55 | Heilongjiang,China | 48.3 | 128.1 | 80.1 | 15.5 | 0.5 | 42.9 | 99.7 | 18.2 | 18.2 | 35 | 45.3 | 59.3 | 65.9 | 74.1 | 79.1 | 109.1 |
| G82 | Hefeng 45 | Heilongjiang,China | 48.3 | 128.1 | 89.3 | 17.8 | 0.4 | 33 | 72.9 | 16.3 | 24.5 | 32 | 48.4 | 57 | 66 | 72.1 | 81.2 | 111.2 |
| G83 | Kenfeng 15 | Heilongjiang,China | 48.3 | 128.1 | 66.2 | 16 | 0 | 50.9 | 94.8 | 14.6 | 15.4 | 29.1 | 38.6 | 52.6 | 62.5 | 68.7 | 76.7 | 108.7 |
| G84 | Chasedou | Jilin,China | 43.4 | 126.3 | 92.9 | 16 | 1.3 | 39.9 | 78.8 | 14.9 | 19.1 | 38.4 | 60.5 | 67.3 | 77.3 | 82.3 | 86.3 | 114.4 |
| G85 | He 05-991 | Heilongjiang,China | 48.3 | 128.1 | 98.8 | 17.8 | 1.2 | 27.8 | 59.9 | 15.7 | 26.2 | 27.7 | 48.1 | 52.5 | 61.5 | 66.7 | 74.7 | 100.5 |
| G86 | Xiaolimoshidou | Heilongjiang,China | 39.2 | 116.3 | 71.5 | 16.3 | 5.7 | 41.3 | 24.7 | 0.7 | 2.8 | 50.5 | 56.8 | 61.8 | 69.8 | 79.8 | 84.9 | 110.9 |
| G87 | Jingshanpu | Heilongjiang,China | 48.3 | 128.1 | 78.9 | 18.7 | 4.3 | 50.2 | 108.2 | 18.6 | 17.2 | 37.6 | 58.5 | 64.5 | 70.6 | 77.6 | 84.6 | 114.6 |
| G88 | Qinganheidou | Heilongjiang,China | 48.3 | 128.1 | 70.8 | 13.1 | 2.9 | 40.8 | 42.2 | 5.5 | 13 | 49.8 | 57.4 | 64.8 | 72.1 | 79.1 | 85.1 | 118.1 |
| G89 | Dongnong594 | Heilongjiang,China | 48.3 | 128.1 | 76.7 | 16.5 | 0.1 | 50.2 | 111.7 | 19.1 | 17 | 37.9 | 51.8 | 60.3 | 66.3 | 69.9 | 76.9 | 116.9 |
| G90 | Yapoche | Heilongjiang,China | 48.3 | 128.1 | 93.3 | 20.9 | 2.9 | 49.3 | 90.5 | 16.4 | 18.2 | 47 | 53.2 | 60.5 | 67.6 | 74.6 | 86.6 | 117.6 |
| G91 | Kennong 29 | Heilongjiang,China | 48.3 | 128.1 | 68.7 | 14.9 | 1.3 | 53.4 | 114.8 | 21.7 | 18.9 | 30.9 | 48 | 57.4 | 63.5 | 67 | 75 | 108.1 |
| G92 | Heimoshidou | Jilin,China | 43.4 | 126.3 | 112.4 | 18.2 | 4.8 | 65.8 | 109.6 | 14.7 | 13.4 | 66.4 | 72.5 | 80.5 | 87.5 | 93.5 | 100.5 | 125 |
| G93 | Ha 04-1824 | Heilongjiang,China | 48.3 | 128.1 | 86.7 | 16.3 | 1 | 43.1 | 102.5 | 18.5 | 13 | 32.6 | 47.9 | 50.9 | 61.1 | 68 | 75 | 107 |
| G94 | Fangzhengmoshidou | Heilongjiang,China | 48.3 | 128.1 | 86.7 | 18.2 | 8.4 | 38.8 | 65.1 | 9.1 | 14.3 | 50.4 | 58.3 | 73.3 | 80.3 | 86.3 | 92.3 | 121.3 |
| G95 | Helongyoutai | Jilin,China | 43.4 | 126.3 | 107.8 | 16.8 | 4.4 | 49.1 | 103.2 | 19.3 | 18.9 | 38.6 | 47.5 | 55.4 | 60.6 | 67.6 | 77.6 | 106.6 |
| G96 | Kexin 3 | Beijing,China | 40.1 | 116.3 | 73.3 | 16.6 | 0.2 | 32.1 | 72.7 | 13.6 | 18.7 | 72 | 77.9 | 82.9 | 87.9 | 93.9 | 101 | 124 |
| G97 | Yuanbaojin | Heilongjiang,China | 48.3 | 128.1 | 95.5 | 15 | 4.6 | 49 | 121.6 | 23.2 | 19.6 | 41.5 | 49.3 | 55.6 | 63.6 | 70.6 | 78.6 | 109.6 |
| G98 | Zhongzuo J4133 | Beijing,China | 40.1 | 116.3 | 108.5 | 21.2 | 4.1 | 79.2 | 137.3 | 33.6 | 25.1 | 65.8 | 73.2 | 80.2 | 87.2 | 93.2 | 99.2 | 124 |
| G99 | Jilin 30 | Jilin,China | 43.4 | 126.3 | 93 | 19.5 | 1.5 | 36.8 | 94.8 | 17.3 | 18.2 | 57.7 | 68.4 | 78.7 | 85.7 | 92.7 | 98.7 | 124 |
| G100 | Ji 100 | Jilin,China | 43.4 | 126.3 | 87.3 | 15.4 | 2.3 | 55.3 | 115.4 | 20.1 | 17.4 | 49.4 | 56.9 | 69.6 | 76.9 | 82.9 | 89.9 | 119.9 |
| G101 | Suinong 30 | Heilongjiang,China | 48.3 | 128.1 | 76 | 15.9 | 0 | 38.7 | 70.8 | 13.2 | 18.7 | 36.5 | 48.4 | 52.5 | 59.7 | 68.8 | 78.7 | 108.7 |
| G102 | Boige du | Germany | 52.3 | 13.2 | 93.4 | 16.1 | 4.9 | 59.8 | 97.6 | 12.5 | 12.8 | 44.6 | 50.9 | 60.8 | 71.4 | 78.4 | 84.4 | 114.4 |
| G103 | Beidou 16 | Heilongjiang,China | 48.3 | 128.1 | 102.2 | 19.1 | 3 | 55.1 | 118.9 | 21.4 | 17.9 | 41.7 | 54 | 63 | 73.4 | 80.5 | 87.5 | 121.5 |
| G104 | Kebei 1 | Heilongjiang,China | 48.3 | 128.1 | 76.2 | 15.5 | 0 | 25.5 | 53.2 | 9 | 16.9 | 31.8 | 40.8 | 55 | 60.5 | 65.8 | 77.8 | 99.6 |
| G105 | Mengdou 21 | Inner Mongolia,China | 44.5 | 111.7 | 47.4 | 10.9 | 0.7 | 46.3 | 64.6 | 13.5 | 21.3 | 28.8 | 38.8 | 45 | 57.8 | 61.6 | 70.9 | 93.9 |
| G106 | Heihe 38 | Heilongjiang,China | 48.3 | 128.1 | 81 | 17.7 | 0.5 | 47.6 | 125.5 | 23.3 | 18.6 | 31.5 | 37.8 | 54.7 | 60.2 | 66.2 | 76.2 | 104.2 |
| G107 | Zhongdou 35 | Beijing,China | 40.1 | 116.3 | 71.3 | 16.6 | 4.1 | 84.6 | 158.4 | 29.5 | 18.8 | 39 | 47 | 54 | 66.9 | 76.9 | 94.9 | 121.9 |
| G108 | Zhonghuang 6 | Beijing,China | 40.1 | 116.3 | 109.2 | 21.2 | 4.7 | 44.1 | 90.7 | 17.9 | 19.6 | 64.4 | 72.4 | 80.4 | 85.4 | 90.4 | 97.7 | 124 |
| G109 | Jiyu 94 | Jilin,China | 43.4 | 126.3 | 103.8 | 19.8 | 5.7 | 58.7 | 127 | 22.7 | 17.9 | 65.2 | 70.2 | 78.3 | 85.3 | 90.3 | 97.3 | 125 |
| G110 | Dongnong 44 | Heilongjiang,China | 48.3 | 128.1 | 56 | 13.4 | 0.1 | 10.7 | 19.3 | 5.3 | 27.6 | 29 | 38 | 44 | 55.3 | 60.5 | 64.5 | 97.5 |
| G111 | Liaonong 2 | Liaoning,China | 40.8 | 122 | 108.4 | 17.8 | 1 | 35.8 | 70.4 | 20.3 | 28.8 | 56.4 | 60.9 | 73.4 | 79.4 | 85.4 | 95.4 | 120.4 |
| G112 | Bei 1484 | America | 48.3 | 128.1 | 71.1 | 15.4 | 0.2 | 36.6 | 79.4 | 13.9 | 17.5 | 28.9 | 38 | 51.4 | 57.9 | 64.6 | 73.1 | 94.1 |
| G113 | Williams82 | America | 41.8 | 92.9 | 100.8 | 19.5 | 3.2 | 67.5 | 122.1 | 20.9 | 16.9 | 46.5 | 56.4 | 74.4 | 80.4 | 86.4 | 93.4 | 124.4 |
| G114 | Jindou 33 | Liaoning,China | 40.8 | 122 | 102.8 | 17.2 | 3 | 59.6 | 101.6 | 23.5 | 23.1 | 66.6 | 74.8 | 79.8 | 85.8 | 92.8 | 98.8 | 125 |
| G115 | Kenfeng 18 | Heilongjiang,China | 48.3 | 128.1 | 73 | 16 | 0.1 | 28 | 74.4 | 11.9 | 15.9 | 31.7 | 45.6 | 58.5 | 62.8 | 71.8 | 82.9 | 109.9 |
| G116 | Suinong 20 | Heilongjiang,China | 48.3 | 128.1 | 75.9 | 16.3 | 0.1 | 39.8 | 90.3 | 18.1 | 20.1 | 29.8 | 42 | 57.1 | 62.5 | 67.6 | 76.6 | 108 |
| G117 | Mengdou 9 | Inner Mongolia,China | 44.5 | 111.7 | 60.3 | 16.8 | 0.1 | 35.3 | 85.9 | 13.7 | 16 | 30 | 42.6 | 55.4 | 59.6 | 63.4 | 70.5 | 95.5 |
| G118 | L-9 | America | 41.8 | 92.9 | 101 | 23.1 | 3.4 | 58.6 | 102 | 20.2 | 19.8 | 74.5 | 81.5 | 88.5 | 95.5 | 105.6 | 111.6 | 124 |
| G119 | Suinong 1 | Heilongjiang,China | 48.3 | 128.1 | 80.3 | 19.3 | 3.3 | 57.9 | 101.5 | 15.1 | 14.9 | 29 | 46.7 | 56 | 63 | 71 | 78 | 109.2 |
| G120 | Kenfeng 14 | Heilongjiang,China | 48.3 | 128.1 | 92.9 | 20.9 | 0.4 | 40.3 | 94.5 | 18.5 | 19.7 | 32.9 | 45.4 | 58.5 | 63.9 | 73.9 | 80.9 | 111.9 |
| G121 | Tiejiazi | Liaoning,China | 40.8 | 122 | 89.4 | 16.9 | 0.1 | 34.7 | 76.3 | 16.1 | 21.1 | 34.9 | 45.9 | 55.9 | 62 | 66.9 | 78.9 | 109.9 |
| G122 | Suinong 4 | Heilongjiang,China | 48.3 | 128.1 | 80.2 | 19.6 | 2 | 32.5 | 66.9 | 15.1 | 22.7 | 29 | 46 | 58 | 63 | 74 | 80 | 112 |
| G123 | Jinshanchamoshidou | Jilin,China | 43.4 | 126.3 | 119.6 | 16.4 | 6.7 | 79.7 | 100.3 | 12.5 | 12.4 | 73 | 78.9 | 83.9 | 88.9 | 95.9 | 102.9 | 125 |
| G124 | Longpin 03-311 | Heilongjiang,China | 48.3 | 128.1 | 98.7 | 17.9 | 0 | 43.7 | 90.2 | 23.1 | 27.2 | 27 | 49.4 | 55.6 | 63.6 | 70.6 | 78.6 | 109.7 |
| G125 | Jilinchalihua | Jilin,China | 43.4 | 126.3 | 82.3 | 12.9 | 3.8 | 33.9 | 59.6 | 4.5 | 7.1 | 66.8 | 74.2 | 80.2 | 85.2 | 90.2 | 95.2 | 122.2 |
| G126 | Heinong 37 | Heilongjiang,China | 48.3 | 128.1 | 54 | 15.6 | 2.2 | 58.7 | 101 | 22 | 21.7 | 45.7 | 53.7 | 58.7 | 71.7 | 76.7 | 83.7 | 90.7 |
| G127 | Huajiang 4403 | Heilongjiang,China | 48.3 | 128.1 | 69 | 15.3 | 0.1 | 36 | 71.9 | 15.4 | 21.4 | 29 | 46 | 53 | 60 | 66 | 77 | 107 |
| G128 | Huangbaozhu | Liaoning,China | 40.8 | 122 | 85.8 | 12.9 | 1.4 | 32.3 | 59.5 | 10.1 | 17 | 58.1 | 66.1 | 73.1 | 80.1 | 87.1 | 93.1 | 123.1 |
| G129 | Suinong 25 | Heilongjiang,China | 48.3 | 128.1 | 91.1 | 20.5 | 1.8 | 45.6 | 83.4 | 15.2 | 18.3 | 37.8 | 51.8 | 59.8 | 66.8 | 76.8 | 82.8 | 112.8 |
| G130 | Sui 05-7304 | Heilongjiang,China | 48.3 | 128.1 | 65.6 | 14.9 | 1 | 59.9 | 88.7 | 17.7 | 20 | 31 | 48 | 52 | 59 | 63 | 70 | 103 |
| G131 | Jidou 9 | Hebei,China | 39.2 | 116.3 | 99.4 | 21.8 | 1.6 | 45 | 94.2 | 24.9 | 26.4 | 50.9 | 67.9 | 73.9 | 80.9 | 86.9 | 93.9 | 123.5 |
| G132 | Mengdou 14 | Inner Mongolia,China | 44.5 | 111.7 | 78.3 | 16.6 | 0.9 | 37.8 | 98.1 | 19.6 | 20 | 28.7 | 45.7 | 51.7 | 56.7 | 62.7 | 78.7 | 107.7 |
| G133 | Handou 5 | Hebei,China | 39.2 | 116.3 | 100.7 | 16.4 | 2.8 | 44.3 | 89.8 | 25.7 | 29 | 51 | 66 | 78 | 84 | 92 | 99 | 125 |
| G134 | Jidou 17 | Hebei,China | 39.2 | 116.3 | 109.4 | 19.2 | 4.2 | 57.2 | 113.6 | 26.5 | 23.3 | 58.6 | 68.6 | 78.6 | 85.6 | 92.6 | 99.6 | 125 |
| G135 | Aika 166 | Heilongjiang,China | 44.2 | 26.1 | 106.8 | 19.8 | 2 | 48.5 | 87.1 | 19.2 | 22.1 | 35.7 | 48.7 | 57.7 | 65.7 | 76.7 | 82.7 | 112.7 |
| G136 | Sui 04-6018 | Heilongjiang,China | 48.3 | 128.1 | 68.4 | 17.5 | 0.6 | 58.2 | 118.2 | 22.6 | 19 | 37.5 | 46.5 | 55.5 | 63.5 | 72.5 | 80.5 | 107.5 |
| G137 | Dongnong 48 | Heilongjiang,China | 48.3 | 128.1 | 90.3 | 17.1 | 0.9 | 42.5 | 112.7 | 19.4 | 17.4 | 36 | 55 | 59 | 64 | 72 | 80 | 110 |
| G138 | L-79 | America | 43.4 | 79.3 | 67.1 | 13.4 | 1.4 | 26.4 | 44.4 | 8.9 | 20.1 | 28.2 | 38.2 | 49.2 | 59.2 | 64.2 | 75.2 | 103.2 |
| G139 | Zhonghuang 35 | Beijing,China | 40.1 | 116.3 | 83.5 | 16 | 2.8 | 75.5 | 164.6 | 27.5 | 16.7 | 32.2 | 47.2 | 52.2 | 59.2 | 66.2 | 79.2 | 104.2 |
| G140 | Kennong 30 | Heilongjiang,China | 48.3 | 128.1 | 75.3 | 15.1 | 1.6 | 44.9 | 101.8 | 17.8 | 17.8 | 30.2 | 46.6 | 50.5 | 61.8 | 65.8 | 72.8 | 109.3 |
| G141 | Zhonghuang 30 | Beijing,China | 40.1 | 116.3 | 78.7 | 18.3 | 1.3 | 42.6 | 100.3 | 21.3 | 22.2 | 54.5 | 64.3 | 74.3 | 83 | 90 | 99 | 125 |
| G142 | Baichengmoshidou | Jilin,China | 43.4 | 126.3 | 92.6 | 17.1 | 6.7 | 57.1 | 170.7 | 13 | 6.8 | 50.8 | 61 | 71.5 | 78.5 | 85.5 | 93.5 | 124 |
| G143 | L-10 | America | 43.4 | 79.3 | 67.2 | 13.6 | 3.6 | 55.7 | 86.4 | 15.4 | 17.3 | 51.9 | 58.4 | 70.5 | 77.5 | 84.5 | 90.7 | 124.4 |
| G144 | Zaoshu 18 | Beijing,China | 40.1 | 116.3 | 66.5 | 16.7 | 2.7 | 29 | 70.6 | 16.9 | 24.6 | 47.6 | 60.7 | 70.6 | 75.6 | 82.6 | 89.6 | 121.6 |
| G145 | Zhonghuang 10 | Beijing,China | 40.1 | 116.3 | 102.5 | 15.6 | 4.2 | 51.7 | 79.4 | 25.4 | 18 | 62.3 | 68.3 | 76.3 | 83.3 | 90.3 | 98.3 | 124 |
| G146 | Dongnong L-13 | Heilongjiang,China | 43.4 | 79.3 | 92.8 | 15.6 | 0.2 | 29.1 | 50.5 | 13 | 25.7 | 30.4 | 48.5 | 58.1 | 65.1 | 73.1 | 80.1 | 113.1 |
| G147 | Neifeng 15 | Heilongjiang,China | 48.3 | 128.1 | 88.9 | 15.9 | 2 | 36.1 | 72.8 | 13.3 | 17.4 | 36.3 | 49.4 | 55.3 | 64.6 | 69.6 | 76.6 | 104.6 |
| G148 | Dongnong 47 | Heilongjiang,China | 48.3 | 128.1 | 80.7 | 16.8 | 1.1 | 39.3 | 100.1 | 20.3 | 20.3 | 32.5 | 47.5 | 56.3 | 63.3 | 69.3 | 71.3 | 109.3 |
| G149 | Hefeng 29 | Heilongjiang,China | 48.3 | 128.1 | 85.2 | 17.5 | 1.2 | 39.3 | 73.7 | 13.9 | 18.4 | 47.5 | 55.2 | 65 | 71.5 | 79.2 | 87 | 115 |
| G150 | Dongnong 07-909 | Heilongjiang,China | 48.3 | 128.1 | 69.5 | 16.1 | 0.5 | 35.6 | 78.9 | 12.6 | 15.9 | 34.1 | 48.7 | 54.9 | 59.3 | 66 | 75.7 | 113.7 |
| G151 | Hefeng 52 | Heilongjiang,China | 48.3 | 128.1 | 78.5 | 17.5 | 0.5 | 57.3 | 122.5 | 20.4 | 16.6 | 37.7 | 48.6 | 61.6 | 66.2 | 71.2 | 78.2 | 111.2 |
| G152 | Heihe 18 | Heilongjiang,China | 48.3 | 128.1 | 68.6 | 13.4 | 0.1 | 25 | 48.1 | 10.9 | 20.7 | 33.9 | 36.6 | 47.3 | 54.9 | 63.3 | 68.3 | 100.3 |
| G153 | Neifeng 11 | Heilongjiang,China | 48.3 | 128.1 | 71.6 | 16.6 | 1.1 | 66.3 | 141.8 | 21.3 | 16 | 32.7 | 49.3 | 55.7 | 62.2 | 68.2 | 75.2 | 108.2 |
| G154 | Heihexiaohuangdou | Heilongjiang,China | 48.3 | 128.1 | 52.1 | 12.2 | 1.6 | 19.4 | 27.2 | 5.2 | 19.1 | 34 | 38.6 | 50.3 | 56.4 | 63.1 | 68 | 96 |
| G155 | Zhongdou 27 | Beijing,China | 40.1 | 116.3 | 95.3 | 17.5 | 3.2 | 29.9 | 50.8 | 11.5 | 22.5 | 58.6 | 68.5 | 76.5 | 83.5 | 89.5 | 96.5 | 121.5 |
| G156 | Bei 4834 | Heilongjiang,China | 48.3 | 128.1 | 68.2 | 15 | 5.2 | 67.6 | 72.8 | 16.2 | 22.3 | 47 | 54.7 | 58.6 | 64.7 | 69.7 | 76.7 | 111.7 |
| G157 | Heilongjiang 41 | Heilongjiang,China | 48.3 | 128.1 | 96.1 | 15.1 | 0.4 | 37.9 | 72.8 | 16.8 | 23 | 48.4 | 55.7 | 67.2 | 77.1 | 83.1 | 89.1 | 121.5 |
| G158 | Zhongpin 03-5179 | Beijing,China | 40.1 | 116.3 | 76.7 | 17 | 3.2 | 46.6 | 81.2 | 18 | 22.2 | 38 | 48 | 56 | 64.4 | 72.4 | 87.4 | 123.4 |
| G159 | Hefeng 37 | Heilongjiang,China | 48.3 | 128.1 | 105 | 22.8 | 4.6 | 68 | 140.4 | 33.3 | 23.7 | 74.4 | 82.4 | 90.4 | 97.4 | 105.4 | 111.4 | 125 |
| G160 | Dongnong 46 | Heilongjiang,China | 48.3 | 128.1 | 66.6 | 14.7 | 0.3 | 24.4 | 66.9 | 14.3 | 21.4 | 34 | 49 | 57.2 | 65.5 | 71.1 | 78.1 | 109.1 |
| G161 | Dongnong 43 | Heilongjiang,China | 48.3 | 128.1 | 79.5 | 15.1 | 2.7 | 36.5 | 53.7 | 11.1 | 20.5 | 39 | 48 | 56 | 62.5 | 67.4 | 74.4 | 110.4 |
| G162 | Dongnong 1068 | Heilongjiang,China | 48.3 | 128.1 | 74.8 | 18 | 1.6 | 50.2 | 96.2 | 17.4 | 18 | 34 | 38.7 | 57.6 | 63.4 | 68.4 | 75.4 | 109.4 |
| G163 | Dongnong 56 | Heilongjiang,China | 48.3 | 128.1 | 85 | 15.8 | 0.1 | 35.1 | 53 | 10.8 | 20.3 | 42.5 | 49 | 58.6 | 65.5 | 72.5 | 86.5 | 119.5 |
| G164 | Jiunong 21 | Jilin,China | 43.4 | 126.3 | 100.1 | 21.3 | 1.8 | 57.8 | 135.3 | 25.4 | 19 | 38.4 | 62.3 | 71.1 | 78.1 | 86.1 | 94.1 | 125 |
| G165 | Chamoshidou | Jilin,China | 43.4 | 126.3 | 91.7 | 14.3 | 6.3 | 63 | 130.6 | 10.5 | 18.6 | 58.3 | 69 | 78 | 85 | 92 | 99 | 125 |
| G166 | Daliheidou | Shandong,China | 36.2 | 118.3 | 76.1 | 16.8 | 4.6 | 36.6 | 76.3 | 13.8 | 18.7 | 36.7 | 54.9 | 61.2 | 66.2 | 72.2 | 81.2 | 116.2 |
| G167 | Zhongpin 03-5373 | Beijing,China | 40.1 | 116.3 | 104.8 | 16.4 | 5.8 | 63.6 | 96 | 25.4 | 26.5 | 69.8 | 76.8 | 82.8 | 89.7 | 96.7 | 103.7 | 125 |
| G168 | Wuxing 4 | Hebei,China | 39.2 | 116.3 | 68.5 | 13.2 | 1.9 | 25.6 | 37.7 | 16.7 | 33.7 | 70.6 | 77.6 | 85 | 91 | 98 | 105 | 125 |
| G169 | Hefeng 55 | Heilongjiang,China | 48.3 | 128.1 | 84.2 | 21.1 | 0.3 | 32.2 | 51.6 | 10.4 | 20.6 | 29 | 44 | 53.2 | 63.7 | 70.7 | 77.7 | 110.7 |
| G170 | Suinong 28 | Heilongjiang,China | 48.3 | 128.1 | 71.1 | 17.5 | 0.6 | 43.1 | 85.3 | 14.6 | 16.9 | 33 | 47.4 | 61.6 | 68.3 | 72.3 | 79.3 | 109.3 |
| G171 | Zhongpin 03-5334 | Beijing,China | 40.1 | 116.3 | 55.5 | 15 | 6.8 | 74.3 | 137.3 | 30.5 | 22.2 | 66.4 | 70.4 | 78.4 | 86.4 | 94.4 | 101.4 | 125 |
| G172 | Fengshou 6 | Heilongjiang,China | 48.3 | 128.1 | 79.8 | 16.2 | 1.5 | 32.8 | 82 | 13.4 | 16.3 | 30.9 | 40.4 | 49.7 | 62.6 | 69.6 | 77.6 | 108.6 |
| G173 | Dunajika | Russia | 61.5 | 98 | 80.9 | 16.7 | 3 | 58.6 | 112.1 | 12.6 | 10.9 | 30.3 | 44 | 51 | 63.4 | 69.4 | 77.4 | 103.4 |

| **Number** | **Accession** | **Origin** | **Latitude (°N)** | **Longitude (°W)** | **2016 Harbin** | | | | | | | | | | | | | |
| --- | --- | --- | --- | --- | --- | --- | --- | --- | --- | --- | --- | --- | --- | --- | --- | --- | --- | --- |
|  |  |  |  |  | **PH**  **(cm)** | **NMN** | **EBN** | **EPN** | **GN** | **GW**  **(g)** | **HGW**  **(g)** | **R1**  **(day)** | **R2**  **(day)** | **R3**  **(day)** | **R4**  **(day)** | **R5**  **(day)** | **R6**  **(day)** | **R7**  **(day)** |
| G1 | Tiedou 50 | Liaoning,China | 40.8 | 122 | 87.1 | 19.3 | 4.3 | 69.7 | 139.4 | 22.3 | 17 | 56 | 60 | 71 | 79 | 87 | 97 | 123 |
| G2 | Datun xiaoheidou | Hebei,China | 39.2 | 116.3 | 116.8 | 18.6 | 5.2 | 99.4 | 200.8 | 21.2 | 10.5 | 71.9 | 75.9 | 79.9 | 85 | 92 | 103 | 124 |
| G3 | Ken 04-8579 | Heilongjiang,China | 48.3 | 128.1 | 81.5 | 17.8 | 2.5 | 63.9 | 139.9 | 24.8 | 17.2 | 44.8 | 49.8 | 55 | 63.8 | 71 | 84 | 108 |
| G4 | Kangxian2 | Heilongjiang,China | 48.3 | 128.1 | 103.2 | 16.6 | 1.7 | 45.6 | 94.2 | 14 | 14.9 | 50.8 | 53.8 | 59.8 | 73 | 78 | 91 | 116 |
| G5 | Liushitianhuancang | Liaoning,China | 40.8 | 122 | 105.7 | 17.3 | 3.3 | 58 | 116 | 13.4 | 13.7 | 46 | 55 | 65 | 75 | 85 | 101 | 121 |
| G6 | Tiedou 58 | Liaoning,China | 40.8 | 122 | 104.2 | 18.2 | 1.2 | 39.8 | 79.6 | 14.4 | 22.9 | 71 | 74 | 80 | 87 | 91 | 105 | 124 |
| G7 | Zhonghuang 20 | Beijing,China | 40.1 | 116.3 | 144.7 | 17.8 | 3 | 52.2 | 104.4 | 15.9 | 16.8 | 50.9 | 56.9 | 69 | 80 | 87 | 100 | 122 |
| G8 | Jichanghuangdou 1 | Xinjiang,China | 41.2 | 81.4 | 102.4 | 19.8 | 2.2 | 56.6 | 113.2 | 17.1 | 17.2 | 69.9 | 76.9 | 82 | 89 | 95 | 113 | 124 |
| G9 | Longquandadou | Heilongjiang,China | 48.3 | 128.1 | 42.7 | 13.3 | 3.3 | 29.3 | 58.6 | 8.2 | 31.4 | 44.9 | 48.9 | 52.9 | 64.9 | 70 | 82 | 114 |
| G10 | Xiaobaiqi | Liaoning,China | 40.8 | 122 | 102.6 | 18.4 | 5.8 | 102.4 | 204.8 | 20.1 | 14.4 | 66.9 | 70.9 | 77 | 87 | 92 | 105 | 125 |
| G11 | Gongye 04L-141 | Jilin,China | 43.4 | 126.3 | 100.7 | 20.1 | 3.6 | 104.3 | 208.6 | 43.1 | 32.4 | 49.8 | 65.8 | 74.8 | 83.8 | 90 | 98 | 119 |
| G12 | Tiedou 52 | Liaoning,China | 40.8 | 122 | 79.8 | 14.9 | 1.9 | 36.1 | 84.3 | 15.8 | 18.6 | 50 | 53 | 57 | 67.5 | 81 | 95 | 120.2 |
| G13 | L-57 | America | 43.4 | 79.3 | 87.1 | 19.1 | 4.8 | 94.4 | 188.8 | 33.9 | 24.4 | 49.9 | 58.9 | 67.9 | 78.9 | 86 | 103 | 120 |
| G14 | He 05-31 | Heilongjiang,China | 48.3 | 128.1 | 92.1 | 15.6 | 2 | 57.2 | 114.4 | 14.3 | 15.6 | 47.9 | 54.9 | 61.9 | 69.1 | 78 | 91 | 116 |
| G15 | L-21 | Canada | 43.4 | 79.3 | 79.4 | 17.6 | 2.8 | 40.6 | 81.2 | 15 | 20.2 | 35 | 45.1 | 51.7 | 65.5 | 72.8 | 90.6 | 104.6 |
| G16 | Hongfeng11 | Heilongjiang,China | 48.3 | 128.1 | 76.1 | 17.4 | 0.1 | 41.2 | 82.4 | 13.3 | 18.9 | 37.5 | 48 | 53 | 66 | 73 | 86 | 111 |
| G17 | Heinong 33 | Heilongjiang,China | 48.3 | 128.1 | 92.8 | 17.5 | 2.1 | 55.6 | 135.6 | 21.8 | 16 | 41.8 | 48 | 54 | 67 | 75 | 90 | 112 |
| G18 | Liao 98072 | Liaoning,China | 40.8 | 122 | 99.5 | 17.4 | 2.4 | 63.6 | 127.2 | 22.5 | 23 | 55 | 59 | 71 | 81 | 88 | 103 | 127 |
| G19 | Kenfeng 22 | Heilongjiang,China | 48.3 | 128.1 | 71.2 | 15.8 | 1.6 | 51.3 | 122 | 22.2 | 18.2 | 41.2 | 50 | 53 | 64 | 70 | 86 | 106 |
| G20 | Jiyu 89 | Jilin,China | 43.4 | 126.3 | 89.8 | 16 | 2.4 | 64.7 | 129.4 | 14 | 12.6 | 41.8 | 49.8 | 60.8 | 70.9 | 78 | 91 | 117 |
| G21 | Tiedou 51 | Liaoning,China | 40.8 | 122 | 92.6 | 17.4 | 3.1 | 52.8 | 106.8 | 20 | 18.7 | 50.8 | 54.8 | 60 | 69 | 81 | 97 | 129 |
| G22 | Tongnong 13 | Jilin,China | 43.4 | 126.3 | 105.4 | 18.1 | 1 | 40.2 | 80.4 | 22.9 | 20.4 | 51 | 59.7 | 69 | 79 | 85 | 98 | 124 |
| G23 | Bei 1873 | Heilongjiang,China | 48.3 | 128.1 | 58.3 | 15.1 | 2.9 | 64.2 | 128.4 | 19.7 | 16.1 | 44 | 49 | 56 | 66 | 72.1 | 87 | 111 |
| G24 | Hefeng 50 | Heilongjiang,China | 48.3 | 128.1 | 69.9 | 14.3 | 3.1 | 71.6 | 160.4 | 25.7 | 16 | 45 | 50 | 57 | 67 | 73 | 85 | 108 |
| G25 | Sui 02-339 | Heilongjiang,China | 48.3 | 128.1 | 92.8 | 19.8 | 3.6 | 58.4 | 135.2 | 20.3 | 16.9 | 44.4 | 52 | 58 | 69 | 75 | 90 | 114 |
| G26 | Hujiao 03-286 | Inner Mongolia,China | 44.5 | 111.7 | 60.5 | 13.2 | 0.5 | 37.2 | 77.7 | 14.2 | 18.3 | 37.4 | 44.4 | 51 | 59 | 65 | 75 | 98 |
| G27 | Heinong 44 | Heilongjiang,China | 48.3 | 128.1 | 61.8 | 12.3 | 0.7 | 49.9 | 99.8 | 13 | 15 | 43 | 49 | 53 | 67 | 72 | 84 | 111 |
| G28 | Hujiao 04-528 | Inner Mongolia,China | 44.5 | 111.7 | 50.4 | 10.7 | 0.8 | 41.7 | 83.4 | 14.2 | 17.9 | 37.2 | 45.1 | 50 | 56 | 64 | 75 | 95 |
| G29 | Fengshou 10 | Heilongjiang,China | 48.3 | 128.1 | 90 | 18.2 | 3 | 43.7 | 93.1 | 16.4 | 17.5 | 44.9 | 49.9 | 56 | 67 | 73 | 89 | 107 |
| G30 | Hersong 2 | Ukraine | 50.3 | 30.3 | 106.8 | 15.4 | 4 | 59.8 | 119.6 | 16.8 | 17.7 | 57.9 | 63.9 | 72 | 79 | 84 | 97 | 119 |
| G31 | Jinzhou 4-1 | Liaoning,China | 40.8 | 122 | 84.4 | 16.1 | 2.4 | 65 | 130 | 17.5 | 18 | 63.9 | 67.9 | 76 | 80 | 83 | 95 | 123 |
| G32 | Suinong 14 | Heilongjiang,China | 48.3 | 128.1 | 72 | 16.6 | 1.9 | 70.7 | 159.4 | 27.6 | 17.3 | 42 | 50 | 59 | 70 | 75 | 89 | 112 |
| G33 | Tiejiasilihuang | Jilin,China | 43.4 | 126.3 | 106.5 | 19.6 | 2.2 | 60 | 127.8 | 17.3 | 13.7 | 54.6 | 57.6 | 68 | 76 | 84 | 96 | 120 |
| G34 | Tiefeng 8 | Liaoning,China | 40.8 | 122 | 97.2 | 15.2 | 5.4 | 52.2 | 104.4 | 25.6 | 25.5 | 71.9 | 76.9 | 83.9 | 88.9 | 93 | 108 | NA |
| G35 | Silihuang | Jilin,China | 43.4 | 126.3 | 83.1 | 16.6 | 2.9 | 42 | 90.7 | 19.7 | 21.7 | 49.8 | 52.8 | 58.8 | 72 | 75 | 87 | 108 |
| G36 | Bei 1361 | Heilongjiang,China | 48.3 | 128.1 | 82.6 | 17.4 | 1.8 | 55.6 | 111.6 | 17.8 | 15.9 | 40.9 | 49.4 | 53.5 | 66.5 | 74.5 | 89.5 | 108.5 |
| G37 | L-5 | Canada | 43.4 | 79.3 | 96.5 | 16.8 | 2.1 | 42.4 | 93.7 | 14 | 14.6 | 56 | 59 | 67 | 77 | 85 | 101 | 119 |
| G38 | Maoyandou | Gansu, China | 32.3 | 92.1 | 88.3 | 14.7 | 4 | 61.7 | 123.4 | 23 | 19.7 | 52.9 | 54.9 | 62 | 69 | 78 | 90 | 112 |
| G39 | Fengdihuang | Jilin,China | 43.4 | 126.3 | 83.5 | 14.9 | 4.9 | 36.9 | 73.8 | 12.5 | 20.2 | 58.9 | 64.9 | 73 | 83 | 89 | 103 | 124 |
| G40 | Tiedou 54 | Liaoning,China | 40.8 | 122 | 91.6 | 18 | 1.2 | 63.6 | 141.2 | 28.1 | 19.9 | 62.9 | 70.9 | 77 | 84 | 89 | 99 | 127 |
| G41 | Jiunong 20 | Jilin,China | 43.4 | 126.3 | 84 | 18.8 | 1.4 | 46.3 | 119.1 | 18.6 | 15.9 | 50 | 60 | 75 | 81 | 86 | 98 | 121 |
| G42 | Duludou | Jilin,China | 43.4 | 126.3 | 58.2 | 13.3 | 1.7 | 37 | 74 | 10.3 | 17 | 52 | 56.1 | 61.1 | 67 | 72 | 86.1 | 107.1 |
| G43 | Sui 03-3046 | Heilongjiang,China | 48.3 | 128.1 | 76 | 14.9 | 0.1 | 48 | 96 | 16.6 | 17.8 | 39.7 | 46.7 | 52 | 61 | 69 | 85 | 106 |
| G44 | Hefeng 47 | Heilongjiang,China | 48.3 | 128.1 | 137.1 | 19.2 | 0.5 | 58.3 | 116.6 | 21.9 | 19.7 | 55.9 | 63.9 | 74 | 82 | 88 | 99 | 123 |
| G45 | V111-4 | Jilin,China | 43.4 | 126.3 | 92.8 | 17.8 | 0.8 | 66.1 | 159.5 | 24.6 | 15.8 | 44 | 50.2 | 59.2 | 72.2 | 82.2 | 94.2 | 118.2 |
| G46 | Longxuan 1 | Heilongjiang,China | 48.3 | 128.1 | 76.7 | 15.6 | 1.7 | 47.4 | 102 | 16 | 15.7 | 41.8 | 50 | 58 | 67 | 72 | 87 | 109 |
| G47 | Dongnong 8004 | Heilongjiang,China | 48.3 | 128.1 | 49.3 | 15.7 | 3.9 | 46 | 92 | 16.8 | 30.9 | 45.9 | 50.9 | 54 | 67 | 76 | 88 | 110 |
| G48 | Sui 03-3952 | Heilongjiang,China | 48.3 | 128.1 | 80.3 | 18 | 2.6 | 69 | 138 | 24.3 | 18.5 | 49.9 | 55.9 | 62 | 74 | 79 | 91 | 118 |
| G49 | Hefeng 25 | Heilongjiang,China | 48.3 | 128.1 | 84.6 | 17.8 | 3.7 | 68.1 | 136.2 | 18.3 | 17.5 | 50.9 | 55.9 | 68 | 75 | 79 | 92 | 114 |
| G50 | Liaodou 3 | Liaoning,China | 40.8 | 122 | 137.5 | 15.9 | 1.8 | 47.8 | 95.6 | 19.1 | 21.7 | 49.9 | 55.9 | 65 | 75 | 84 | 98 | 124 |
| G51 | Hujiao 423 | Inner Mongolia,China | 44.5 | 111.7 | 78.9 | 17.3 | 1.9 | 50.1 | 111.8 | 19.7 | 17.3 | 39.3 | 46.2 | 52 | 60 | 68 | 82 | 105 |
| G52 | Liaoxian 1 | Liaoning,China | 40.8 | 122 | 43.3 | 14.5 | 2.7 | 32.1 | 71.3 | 14.9 | 28.2 | 45.9 | 53.9 | 67.9 | 73 | 76 | 90 | 120 |
| G53 | Zhongpin 95-5388 | Beijing,China | 40.1 | 116.3 | 96.2 | 20.4 | 3.6 | 89 | 178 | 32.9 | 24.6 | 65.9 | 71.9 | 81 | 91 | 98 | 113 | 131 |
| G54 | Heihe 45 | Heilongjiang,China | 48.3 | 128.1 | 52 | 15.2 | 1.3 | 42.5 | 85 | 12.2 | 20.2 | 36.9 | 45.6 | 50.6 | 57.6 | 62.6 | 84.6 | 99.6 |
| G55 | L-59 Peking | America | 41.8 | 92.9 | 94.7 | 15.8 | 4.2 | 71.2 | 142.4 | 11.6 | 8.5 | 82.9 | 86.9 | 92 | 98 | 101 | 112 | NA |
| G56 | Sui 04-5804 | Heilongjiang,China | 48.3 | 128.1 | 85.4 | 17.4 | 0.8 | 45.2 | 108 | 16.1 | 14.9 | 44 | 49 | 58.1 | 67.1 | 73.1 | 87.1 | 108.1 |
| G57 | Ji 06B7 | Hebei,China | 39.2 | 116.3 | 89 | 18.4 | 8.2 | 64.8 | 129.6 | 16.8 | 22.7 | 76.9 | 84.9 | 91 | 98 | 102 | 118 | NA |
| G58 | Marca. Joe lisa | Heilongjiang,China | 48.3 | 128.1 | 141.9 | 25.7 | 6.3 | 105.5 | 211 | 38.2 | 13.2 | 67 | 73 | 79 | 85 | 92 | 102 | NA |
| G59 | Jihuang 13 | Hebei,China | 39.2 | 116.3 | 90.2 | 18.2 | 4.2 | 74.8 | 157.2 | 32.6 | 20.7 | 59.3 | 70.6 | 78 | 88 | 94 | 106 | NA |
| G60 | L-28 | Canada | 43.4 | 79.3 | 61 | 17.6 | 6.8 | 67.2 | 134.4 | 18.2 | 14.3 | 36.1 | 45.2 | 51.5 | 59.6 | 68.6 | 89.7 | 105.7 |
| G61 | Beifeng 9 | Heilongjiang,China | 48.3 | 128.1 | 78.9 | 15.9 | 0.3 | 45.4 | 117.8 | 18.3 | 15.4 | 37.3 | 45.3 | 51 | 61 | 69 | 81 | 105 |
| G62 | Zhongzuo 00-683 | Beijing,China | 40.1 | 116.3 | 138.8 | 21 | 1.2 | 68.2 | 136.4 | 33 | 22.4 | 61.9 | 69.9 | 81 | 88 | 93 | 110 | 131 |
| G63 | Dongnong 50 | Heilongjiang,China | 48.3 | 128.1 | 79.7 | 18.4 | 3.6 | 103.1 | 212.9 | 23.7 | 6.7 | 38.4 | 50 | 56 | 69 | 76 | 85 | 107 |
| G64 | Dongnong 93-046 | Heilongjiang,China | 48.3 | 128.1 | 92.9 | 16.9 | 3.6 | 51.1 | 108.5 | 20 | 18.3 | 45.9 | 53.9 | 61 | 75 | 81 | 96 | 120 |
| G65 | Suinong 10 | Heilongjiang,China | 48.3 | 128.1 | 75.1 | 16.6 | 2 | 45.2 | 120.6 | 18.7 | 15.5 | 39.8 | 48 | 57 | 66 | 73 | 88 | 107 |
| G66 | Tiefeng 31 | Liaoning,China | 40.8 | 122 | 88.2 | 19.5 | 3.4 | 64.9 | 142 | 25.6 | 18 | 51 | 55.2 | 68 | 80 | 88 | 103 | NA |
| G67 | Dongnong 49 | Heilongjiang,China | 48.3 | 128.1 | 50.3 | 15 | 0.5 | 40.3 | 87.8 | 13.6 | 15.4 | 35.3 | 44.6 | 50.6 | 60.7 | 66.6 | 80.6 | 97.7 |
| G68 | Huayou 446 | Hebei,China | 39.2 | 116.3 | 83.6 | 15 | 1 | 44.4 | 93.6 | 24.8 | 16.5 | 60.9 | 66.9 | 79 | 87 | 94 | 109 | NA |
| G69 | Heihe 48 | Heilongjiang,China | 48.3 | 128.1 | 71 | 17.6 | 0 | 46.8 | 107 | 17.3 | 16.2 | 37 | 45.5 | 51.2 | 62.2 | 68.2 | 83.2 | 98.2 |
| G70 | Huajiang 2 | Heilongjiang,China | 48.3 | 128.1 | 51.8 | 15 | 0.4 | 25.2 | 53.8 | 8.9 | 16.5 | 36.1 | 41.5 | 46.6 | 55.7 | 61.7 | 80.7 | 94.6 |
| G71 | Suinong 29 | Heilongjiang,China | 48.3 | 128.1 | 69.9 | 16.1 | 1.4 | 39.3 | 87.8 | 15.9 | 18.2 | 41.5 | 50 | 56 | 67 | 79 | 87 | 109 |
| G72 | Hefeng 35 | Heilongjiang,China | 48.3 | 128.1 | 78.4 | 17 | 0.9 | 44.8 | 92.3 | 16.9 | 18.2 | 44.6 | 50 | 59 | 67 | 73 | 86 | 109 |
| G73 | Suinong 8 | Heilongjiang,China | 48.3 | 128.1 | 87.7 | 17.5 | 2.3 | 57.6 | 115.2 | 21.9 | 16 | 42.1 | 51 | 55 | 66 | 71 | 90 | 108 |
| G74 | Jilin 47 | Jilin,China | 43.4 | 126.3 | 84.3 | 18.5 | 1.2 | 71.3 | 142.6 | 26.9 | 18.6 | 49.9 | 58.9 | 71 | 76 | 81 | 94 | 121 |
| G75 | Heinong 48 | Heilongjiang,China | 48.3 | 128.1 | 92.9 | 17.7 | 0.1 | 50.6 | 112.7 | 19.8 | 17.7 | 43.9 | 50.9 | 58.9 | 68.9 | 75 | 87 | 113 |
| G76 | Nova | Italy | 41.5 | 12.3 | 105.8 | 19 | 2 | 48.2 | 96.4 | 22.7 | 20.6 | 55 | 64 | 71 | 80 | 84 | 97 | 124 |
| G77 | Beidou 14 | Heilongjiang,China | 48.3 | 128.1 | 78 | 14.8 | 2 | 51 | 134.2 | 21.7 | 16.2 | 37 | 44.1 | 50.1 | 59.1 | 68.1 | 84.1 | 98.1 |
| G78 | Kangxian 3 | Heilongjiang,China | 48.3 | 128.1 | 102.2 | 16.8 | 2.2 | 59.5 | 133.1 | 20.5 | 15.3 | 41.8 | 50.2 | 55.8 | 68.8 | 80 | 92 | 114 |
| G79 | Dongnong 42 | Heilongjiang,China | 48.3 | 128.1 | 100.8 | 18.8 | 1.4 | 59.4 | 139 | 29.2 | 21 | 38.9 | 48.9 | 54 | 67 | 73 | 87 | 112 |
| G80 | Kenjian 23 | Heilongjiang,China | 48.3 | 128.1 | 62.8 | 15.7 | 1.1 | 54 | 136.8 | 20.7 | 15.4 | 41.9 | 49.9 | 55.9 | 66 | 71 | 84 | 108 |
| G81 | Heinong 55 | Heilongjiang,China | 48.3 | 128.1 | 80.1 | 15.5 | 0.5 | 42.9 | 99.7 | 18.2 | 18.2 | 41.9 | 49.9 | 58.9 | 69 | 75 | 87 | 109 |
| G82 | Hefeng 45 | Heilongjiang,China | 48.3 | 128.1 | 74.1 | 17.2 | 3.9 | 59.7 | 132.7 | 23.5 | 17.7 | 48.9 | 53.9 | 60 | 67 | 74 | 91 | 118 |
| G83 | Kenfeng 15 | Heilongjiang,China | 48.3 | 128.1 | 58.9 | 16.1 | 0.4 | 67.5 | 136.3 | 23.7 | 17.3 | 40.3 | 47 | 58 | 67 | 78 | 87.9 | 112.9 |
| G84 | Chasedou | Jilin,China | 43.4 | 126.3 | 99.4 | 17 | 1.7 | 56.9 | 113.8 | 18 | 16.6 | 54.8 | 65.8 | 74 | 83 | 88 | 101 | 119 |
| G85 | He 05-991 | Heilongjiang,China | 48.3 | 128.1 | 91.4 | 17.6 | 2.2 | 39.5 | 80.2 | 21.5 | 26.1 | 57 | 65 | 74.1 | 82.1 | 88.1 | 104.2 | 121 |
| G86 | Xiaolimoshidou | Heilongjiang,China | 39.2 | 116.3 | 116 | 17.5 | 7 | 76.8 | 153.6 | 5.5 | 3.2 | 57.9 | 64.9 | 70 | 80 | 86 | 96 | 119 |
| G87 | Jingshanpu | Heilongjiang,China | 48.3 | 128.1 | 94.3 | 16 | 4.2 | 67.1 | 134.2 | 15.5 | 12.1 | 49.8 | 58.8 | 67 | 74 | 82 | 92 | 120 |
| G88 | Qinganheidou | Heilongjiang,China | 48.3 | 128.1 | 76.2 | 14 | 3.8 | 31.2 | 62.4 | 7.5 | 17.9 | 56.9 | 62.9 | 70 | 79 | 84 | 96 | 122 |
| G89 | Dongnong594 | Heilongjiang,China | 48.3 | 128.1 | 76.7 | 16.5 | 0.1 | 50.2 | 111.7 | 19.1 | 17 | 42.8 | 53.8 | 63.8 | 73.8 | 80 | 93 | 121 |
| G90 | Yapoche | Heilongjiang,China | 48.3 | 128.1 | 93.6 | 19 | 3.2 | 48.8 | 107.4 | 18.1 | 17 | 50 | 53 | 59 | 73 | 81 | 95 | 119 |
| G91 | Kennong 29 | Heilongjiang,China | 48.3 | 128.1 | 68.7 | 14.9 | 1.3 | 53.4 | 114.8 | 21.7 | 18.9 | 43.4 | 51 | 56 | 68 | 73 | 88 | 107 |
| G92 | Heimoshidou | Jilin,China | 43.4 | 126.3 | 112.4 | 18.2 | 4.8 | 65.8 | 131.6 | 14.7 | 13.4 | 66.9 | 72.9 | 79.9 | 86.9 | 92 | 105 | 127 |
| G93 | Ha 04-1824 | Heilongjiang,China | 48.3 | 128.1 | 76.7 | 17.8 | 1.1 | 48.2 | 123.9 | 21.2 | 17 | 41.2 | 52 | 57 | 69 | 74 | 89 | 112 |
| G94 | Fangzhengmoshidou | Heilongjiang,China | 48.3 | 128.1 | 80 | 17.8 | 5.2 | 101.6 | 203.2 | 38 | 19.9 | 54 | 57 | 69 | 82 | 88 | 98 | 123 |
| G95 | Helongyoutai | Jilin,China | 43.4 | 126.3 | 82.9 | 15.4 | 4.3 | 47.7 | 95.4 | 14.3 | 15.8 | 50.9 | 53.9 | 59 | 66 | 72 | 85 | 105 |
| G96 | Kexin 3 | Beijing,China | 40.1 | 116.3 | 82.8 | 14 | 1.6 | 33.6 | 74.4 | 14.2 | 19.1 | 70.9 | 76.9 | 82 | 85 | 93 | 107 | 130 |
| G97 | Yuanbaojin | Heilongjiang,China | 48.3 | 128.1 | 89.3 | 14 | 4.3 | 34.1 | 72.8 | 10.7 | 14.7 | 51.9 | 54.9 | 59 | 66 | 74 | 84 | 106 |
| G98 | Zhongzuo J4133 | Beijing,China | 40.1 | 116.3 | 138.8 | 18.8 | 1.2 | 61.2 | 122.4 | 20 | 20.5 | 67 | 75 | 85 | 93 | 98 | 111 | 131 |
| G99 | Jilin 30 | Jilin,China | 43.4 | 126.3 | 136.4 | 19.1 | 2.2 | 61.8 | 132.2 | 21.9 | 16.6 | 50.9 | 62.9 | 71 | 80 | 86 | 99 | 124 |
| G100 | Ji 100 | Jilin,China | 43.4 | 126.3 | 87.3 | 15.4 | 2.3 | 55.3 | 115.4 | 20.1 | 17.4 | 52.9 | 55.9 | 69 | 75 | 81 | 94 | 120 |
| G101 | Suinong 30 | Heilongjiang,China | 48.3 | 128.1 | 70.5 | 14.1 | 0.2 | 54.3 | 108.6 | 17.5 | 17.8 | 43.8 | 49.8 | 54 | 64 | 70 | 81 | 105 |
| G102 | Boige du | Germany | 52.3 | 13.2 | 75.5 | 14.3 | 3.8 | 65.5 | 131 | 13.6 | 10.8 | 52 | 55 | 60 | 68 | 75 | 87 | 111 |
| G103 | Beidou 16 | Heilongjiang,China | 48.3 | 128.1 | 102.2 | 19.1 | 3 | 55.1 | 118.9 | 21.4 | 17.9 | 50.9 | 56.9 | 63 | 76 | 86 | 99 | 121 |
| G104 | Kebei 1 | Heilongjiang,China | 48.3 | 128.1 | 75.2 | 14.5 | 2.6 | 47.5 | 102.1 | 18.8 | 18.4 | 42 | 50 | 54.9 | 67.9 | 71.9 | 86.9 | 105.9 |
| G105 | Mengdou 21 | Inner Mongolia,China | 44.5 | 111.7 | 47.8 | 13.6 | 0.9 | 54.5 | 126 | 21.3 | 17.1 | 39.7 | 45.2 | 49.7 | 57.6 | 62.6 | 78.6 | 95.6 |
| G106 | Heihe 38 | Heilongjiang,China | 48.3 | 128.1 | 64.3 | 14.3 | 0.5 | 49.1 | 104.3 | 17 | 18.3 | 37 | 44 | 50 | 60 | 67.1 | 78.1 | 100.1 |
| G107 | Zhongdou 35 | Beijing,China | 40.1 | 116.3 | 71.3 | 16.6 | 4.1 | 84.6 | 169.2 | 29.5 | 18.8 | 48.9 | 51.8 | 57 | 81 | 88 | 104 | 131 |
| G108 | Zhonghuang 6 | Beijing,China | 40.1 | 116.3 | 100 | 16 | 2.4 | 46.4 | 92.8 | 19.9 | 22.8 | 68.9 | 72.9 | 81 | 87 | 92 | 105 | 129 |
| G109 | Jiyu 94 | Jilin,China | 43.4 | 126.3 | 102 | 19.8 | 5.4 | 79.6 | 159.2 | 26.5 | 20.1 | 68.9 | 73.9 | 80 | 85 | 91 | 104 | 129 |
| G110 | Dongnong 44 | Heilongjiang,China | 48.3 | 128.1 | 47.3 | 15 | 1.1 | 48 | 96 | 14.2 | 19.7 | 37.7 | 44.7 | 49.8 | 58.7 | 64.7 | 72.9 | 96.9 |
| G111 | Liaonong 2 | Liaoning,China | 40.8 | 122 | 108.4 | 17.8 | 5 | 35.8 | 71.6 | 20.3 | 21.8 | 67.9 | 70.9 | 77.9 | 82.9 | 87 | 98 | 125 |
| G112 | Bei 1484 | America | 48.3 | 128.1 | 37.2 | 15 | 2.6 | 44.9 | 89.8 | 11.3 | 19.8 | 38 | 44.5 | 50 | 56 | 65.1 | 84.1 | 104 |
| G113 | Williams82 | America | 41.8 | 92.9 | 100.8 | 19.5 | 3.2 | 67.5 | 135 | 20.9 | 16.9 | 54 | 57 | 68 | 80 | 87 | 99 | 112 |
| G114 | Jindou 33 | Liaoning,China | 40.8 | 122 | 102.8 | 17.2 | 3 | 59.6 | 119.2 | 23.5 | 23.1 | 71 | 75 | 83 | 89 | 92 | 106 | NA |
| G115 | Kenfeng 18 | Heilongjiang,China | 48.3 | 128.1 | 84.7 | 17 | 2.9 | 64.4 | 150.9 | 27.2 | 18.1 | 42 | 50.1 | 58.2 | 68.2 | 75.2 | 92.2 | 113.2 |
| G116 | Suinong 20 | Heilongjiang,China | 48.3 | 128.1 | 57.1 | 15.1 | 0.9 | 86.3 | 206.1 | 44.4 | 16.3 | 43.5 | 47 | 58.5 | 68.5 | 75 | 90 | 107 |
| G117 | Mengdou 9 | Inner Mongolia,China | 44.5 | 111.7 | 57.7 | 12.6 | 0.8 | 40.6 | 84.9 | 17.8 | 20.1 | 36 | 45 | 51.9 | 56.9 | 64.9 | 75.9 | 96.9 |
| G118 | L-9 | America | 41.8 | 92.9 | 101 | 12 | 4 | 58.6 | 117.2 | 20.2 | 19.8 | 75.9 | 82.9 | 91 | 97 | 108 | 123 | NA |
| G119 | Suinong 1 | Heilongjiang,China | 48.3 | 128.1 | 87.9 | 18.1 | 3.4 | 89.7 | 179.4 | 34.7 | 22.9 | 55 | 63 | 74.8 | 83.9 | 91 | 109.9 | 128 |
| G120 | Kenfeng 14 | Heilongjiang,China | 48.3 | 128.1 | 88.5 | 18.6 | 1.3 | 44.6 | 115.9 | 22 | 18.8 | 39.2 | 51 | 55 | 68 | 75 | 89 | 116 |
| G121 | Tiejiazi | Liaoning,China | 40.8 | 122 | 136 | 20.3 | 0.4 | 71.4 | 142.8 | 26.7 | 20.8 | 48.9 | 59.9 | 76 | 81 | 86 | 101 | 122 |
| G122 | Suinong 4 | Heilongjiang,China | 48.3 | 128.1 | 66.1 | 16.8 | 3.3 | 82.6 | 165.2 | 24 | 17.8 | 48 | 52.1 | 56.1 | 68.1 | 73.1 | 87.1 | 114.1 |
| G123 | Jinshanchamoshidou | Jilin,China | 43.4 | 126.3 | 101 | 14.6 | 4.6 | 63.4 | 126.8 | 15.5 | 12.3 | 71.9 | 76.9 | 83 | 90 | 95 | 108 | 125 |
| G124 | Longpin 03-311 | Heilongjiang,China | 48.3 | 128.1 | 86.4 | 18.1 | 0.5 | 50.5 | 111.4 | 19.5 | 17.5 | 37.7 | 50 | 55 | 71 | 77 | 91 | 111 |
| G125 | Jilinchalihua | Jilin,China | 43.4 | 126.3 | 139.2 | 14.8 | 4.6 | 31.6 | 63.2 | 4.7 | 7 | 68.9 | 72.9 | 82 | 91 | 98 | 108 | 124 |
| G126 | Heinong 37 | Heilongjiang,China | 48.3 | 128.1 | 54 | 15.6 | 2.2 | 58.7 | 117.4 | 22 | 21.7 | 48.9 | 53.9 | 60.4 | 70 | 77 | 87 | 120 |
| G127 | Huajiang 4403 | Heilongjiang,China | 48.3 | 128.1 | 73.6 | 14.2 | 1 | 42.8 | 103.6 | 16.6 | 16 | 37.7 | 44.4 | 50 | 60.1 | 66.1 | 81.1 | 103.1 |
| G128 | Huangbaozhu | Liaoning,China | 40.8 | 122 | 139.5 | 16.8 | 3.3 | 72.3 | 158.7 | 25.9 | 16.3 | 57.9 | 64.9 | 73 | 81 | 85 | 96 | 123 |
| G129 | Suinong 25 | Heilongjiang,China | 48.3 | 128.1 | 87.2 | 19.1 | 1.8 | 66.1 | 132.2 | 19.6 | 16.2 | 43 | 51 | 57 | 70 | 76 | 92 | 112 |
| G130 | Sui 05-7304 | Heilongjiang,China | 48.3 | 128.1 | 49.9 | 13.5 | 0.9 | 40.1 | 80.2 | 12.1 | 19.4 | 43.4 | 46.6 | 52.2 | 63.2 | 67.2 | 80 | 114 |
| G131 | Jidou 9 | Hebei,China | 39.2 | 116.3 | 99.4 | 21.8 | 1.6 | 45 | 94.2 | 24.9 | 26.4 | 56.9 | 65.9 | 76 | 86 | 95 | 107 | 129 |
| G132 | Mengdou 14 | Inner Mongolia,China | 44.5 | 111.7 | 81.8 | 13.6 | 2.4 | 52.2 | 104.4 | 15.4 | 16.6 | 38 | 45 | 54 | 62.1 | 67.1 | 83.1 | 100.3 |
| G133 | Handou 5 | Hebei,China | 39.2 | 116.3 | 100.7 | 16.4 | 2.8 | 44.3 | 89.8 | 25.7 | 29 | 57.9 | 64.9 | 71 | 84 | 91 | 105 | NA |
| G134 | Jidou 17 | Hebei,China | 39.2 | 116.3 | 137.4 | 19.2 | 4.2 | 57.2 | 114.4 | 26.5 | 23.3 | 63.8 | 70.8 | 78 | 87 | 94 | 109 | NA |
| G135 | Aika 166 | Heilongjiang,China | 44.2 | 26.1 | 75.1 | 16.5 | 4.6 | 61.2 | 122.4 | 17 | 14.9 | 49.9 | 53.9 | 63.9 | 71 | 75 | 87 | 113 |
| G136 | Sui 04-6018 | Heilongjiang,China | 48.3 | 128.1 | 68.4 | 17.5 | 0.6 | 58.2 | 118.2 | 22.6 | 19 | 36 | 45 | 53.2 | 65.2 | 71.2 | 86.2 | 100.2 |
| G137 | Dongnong 48 | Heilongjiang,China | 48.3 | 128.1 | 90.3 | 17.1 | 0.9 | 42.5 | 112.7 | 19.4 | 17.4 | 43.9 | 49.8 | 58.9 | 67 | 74 | 87 | 109 |
| G138 | L-79 | America | 43.4 | 79.3 | 57.2 | 14.5 | 1.6 | 30.2 | 61 | 10.6 | 17.2 | 36 | 43.7 | 49.7 | 58.7 | 65.7 | 82.7 | 97.6 |
| G139 | Zhonghuang 35 | Beijing,China | 40.1 | 116.3 | 83.5 | 16 | 2.8 | 75.5 | 164.6 | 27.5 | 16.7 | 41.8 | 45.8 | 53 | 65 | 72 | 87 | 102 |
| G140 | Kennong 30 | Heilongjiang,China | 48.3 | 128.1 | 75.3 | 15.1 | 1.6 | 44.9 | 101.8 | 17.8 | 17.8 | 36.9 | 47.9 | 54 | 65 | 71 | 86 | 107 |
| G141 | Zhonghuang 30 | Beijing,China | 40.1 | 116.3 | 70.6 | 16.2 | 2 | 61.7 | 133.8 | 31.2 | 23.4 | 55.9 | 63.9 | 79 | 87 | 91 | 104 | 128 |
| G142 | Baichengmoshidou | Jilin,China | 43.4 | 126.3 | 95.7 | 14.8 | 4.7 | 82.5 | 165 | 13.9 | 11.4 | 65 | 71 | 75 | 83 | 87 | 101 | 116 |
| G143 | L-10 | America | 43.4 | 79.3 | 76.4 | 15.5 | 3.8 | 53.8 | 107.6 | 15.4 | 17.3 | 54.9 | 57.9 | 67 | 78 | 85 | 101 | NA |
| G144 | Zaoshu 18 | Beijing,China | 40.1 | 116.3 | 61 | 17 | 4.7 | 59.1 | 128.9 | 29.2 | 22.4 | 60.9 | 65.9 | 73 | 82 | 85 | 97 | 126 |
| G145 | Zhonghuang 10 | Beijing,China | 40.1 | 116.3 | 84.6 | 16.6 | 3.4 | 90.4 | 180.8 | 25.8 | 16 | 65.9 | 73.9 | 80 | 87 | 91 | 106 | 128 |
| G146 | Dongnong L-13 | Heilongjiang,China | 43.4 | 79.3 | 142.4 | 19.1 | 1.4 | 50 | 100 | 18.8 | 22 | 42 | 49 | 56.1 | 69.1 | 75 | 93 | 115 |
| G147 | Neifeng 15 | Heilongjiang,China | 48.3 | 128.1 | 94.1 | 15 | 3.9 | 69.8 | 142.7 | 21.8 | 15.1 | 44.9 | 51 | 56 | 68 | 74 | 85 | 107 |
| G148 | Dongnong 47 | Heilongjiang,China | 48.3 | 128.1 | 72.6 | 17.5 | 2.4 | 53.5 | 129.5 | 21.6 | 16.5 | 38.6 | 50 | 59 | 69 | 74 | 87 | 107 |
| G149 | Hefeng 29 | Heilongjiang,China | 48.3 | 128.1 | 93.4 | 16.9 | 2 | 64.4 | 144.3 | 22.4 | 15.5 | 50 | 54 | 61 | 68 | 78 | 90 | 117 |
| G150 | Dongnong 07-909 | Heilongjiang,China | 48.3 | 128.1 | 88.8 | 19.7 | 1.9 | 53.6 | 107.2 | 15.3 | 14.6 | 37.9 | 44 | 50 | 58 | 67 | 72 | 107 |
| G151 | Hefeng 52 | Heilongjiang,China | 48.3 | 128.1 | 72 | 16.1 | 2.2 | 75 | 182.7 | 26.5 | 14.5 | 41.9 | 49.9 | 58.9 | 68.9 | 74 | 87 | 116 |
| G152 | Heihe 18 | Heilongjiang,China | 48.3 | 128.1 | 43.4 | 11.2 | 0.4 | 31.4 | 65.6 | 11.9 | 18.2 | 35.9 | 43.9 | 51 | 58 | 62 | 72 | 99 |
| G153 | Neifeng 11 | Heilongjiang,China | 48.3 | 128.1 | 70.9 | 15.6 | 2.3 | 53.9 | 120.5 | 20.2 | 16.8 | 42.9 | 48.9 | 56.9 | 68 | 72.9 | 86.9 | 107 |
| G154 | Heihexiaohuangdou | Heilongjiang,China | 48.3 | 128.1 | 64.8 | 15.7 | 2.7 | 63.7 | 156 | 27.6 | 17.6 | 41.1 | 45 | 51 | 60 | 67 | 80 | 99 |
| G155 | Zhongdou 27 | Beijing,China | 40.1 | 116.3 | 85.4 | 16.2 | 6.8 | 83 | 180.8 | 33.9 | 18.8 | 59 | 66 | 75 | 82 | 87 | 105 | 123 |
| G156 | Bei 4834 | Heilongjiang,China | 48.3 | 128.1 | 42.8 | 12.7 | 3.2 | 40.5 | 81 | 11.7 | 22.3 | 45.2 | 50 | 54 | 64 | 70 | 81 | 114 |
| G157 | Heilongjiang 41 | Heilongjiang,China | 48.3 | 128.1 | 96.5 | 15.1 | 1.7 | 48.1 | 96.2 | 18.5 | 16.3 | 47.9 | 58.9 | 63 | 74 | 81 | 92 | 118 |
| G158 | Zhongpin 03-5179 | Beijing,China | 40.1 | 116.3 | 76.7 | 17 | 3.2 | 46.6 | 93.2 | 18 | 22.2 | 50.7 | 52.7 | 61.7 | 79.7 | 86 | 102 | 130 |
| G159 | Hefeng 37 | Heilongjiang,China | 48.3 | 128.1 | 105 | 17.8 | 4.6 | 68 | 140.4 | 33.3 | 20.7 | 76.9 | 84.9 | 91 | 100 | 107 | 120 | NA |
| G160 | Dongnong 46 | Heilongjiang,China | 48.3 | 128.1 | 73.5 | 17.5 | 1.9 | 41.2 | 108.3 | 19.7 | 18.2 | 40.9 | 48.9 | 54.1 | 66.1 | 71.1 | 84.1 | 106.1 |
| G161 | Dongnong 43 | Heilongjiang,China | 48.3 | 128.1 | 71.8 | 16.8 | 2.3 | 65.8 | 131.6 | 19.5 | 16.4 | 52.9 | 56.9 | 62.9 | 73 | 80 | 93 | 112 |
| G162 | Dongnong 1068 | Heilongjiang,China | 48.3 | 128.1 | 85.1 | 14.9 | 1.4 | 34.1 | 86.5 | 15.5 | 18 | 40.2 | 45.3 | 54 | 64 | 71 | 85 | 106 |
| G163 | Dongnong 56 | Heilongjiang,China | 48.3 | 128.1 | 64.4 | 15.6 | 1.9 | 50.2 | 100.4 | 16.8 | 18.6 | 49.9 | 54.9 | 61 | 69 | 76 | 87 | 116 |
| G164 | Jiunong 21 | Jilin,China | 43.4 | 126.3 | 86.5 | 20.7 | 1.4 | 76.6 | 153.2 | 14.7 | 15.9 | 45.9 | 59.9 | 66.9 | 79.9 | 88 | 102 | 129 |
| G165 | Chamoshidou | Jilin,China | 43.4 | 126.3 | 84.7 | 13.3 | 7.6 | 69 | 138 | 5.2 | 9.3 | 68.9 | 80.9 | 84 | 91 | 102 | 112 | 129 |
| G166 | Daliheidou | Shandong,China | 36.2 | 118.3 | 95.4 | 13.3 | 4.1 | 36.8 | 73.6 | 10.8 | 24.9 | 50.8 | 55.8 | 66.8 | 77.8 | 85 | 102 | 123 |
| G167 | Zhongpin 03-5373 | Beijing,China | 40.1 | 116.3 | 104.8 | 16.4 | 5.8 | 63.6 | 127.2 | 25.4 | 26.5 | 71.9 | 76.9 | 83 | 90 | 96 | 108 | 129 |
| G168 | Wuxing 4 | Hebei,China | 39.2 | 116.3 | 97 | 15 | 6.8 | 44 | 88 | 26.1 | 38.4 | 75.9 | 79.9 | 91 | 97 | 102 | 114 | NA |
| G169 | Hefeng 55 | Heilongjiang,China | 48.3 | 128.1 | 81.2 | 16.6 | 0.6 | 42.2 | 90.4 | 15.3 | 16.9 | 39.4 | 48.3 | 56 | 64.9 | 71 | 85.9 | 106.9 |
| G170 | Suinong 28 | Heilongjiang,China | 48.3 | 128.1 | 76 | 17.7 | 0.8 | 56 | 120.2 | 23.9 | 14.9 | 42.9 | 48.9 | 52.9 | 65.9 | 73 | 87.9 | 110.9 |
| G171 | Zhongpin 03-5334 | Beijing,China | 40.1 | 116.3 | 91.6 | 17.6 | 5.4 | 74.8 | 149.6 | 21 | 21 | 70 | 75 | 85 | 90 | 94 | 111 | NA |
| G172 | Fengshou 6 | Heilongjiang,China | 48.3 | 128.1 | 78.5 | 16.1 | 3.1 | 63.6 | 146.2 | 20.2 | 13.9 | 39.9 | 48 | 54 | 65 | 73 | 86 | 105 |
| G173 | Dunajika | Russia | 61.5 | 98 | 84.1 | 14.9 | 3.9 | 66.7 | 133.4 | 12.4 | 10.3 | 46.9 | 49.9 | 56 | 63.9 | 71.9 | 89 | 107 |

| **Number** | **Accession** | **Origin** | **Latitude (°N)** | **Longitude (°W)** | **2015 Changchun** | | | | | | | | | | | | | |
| --- | --- | --- | --- | --- | --- | --- | --- | --- | --- | --- | --- | --- | --- | --- | --- | --- | --- | --- |
|  |  |  |  |  | **PH**  **(cm)** | **NMN** | **EBN** | **EPN** | **GN** | **GW**  **(g)** | **HGW**  **(g)** | **R1**  **(day)** | **R2**  **(day)** | **R3**  **(day)** | **R4**  **(day)** | **R5**  **(day)** | **R6**  **(day)** | **R7**  **(day)** |
| G1 | Tiedou 50 | Liaoning,China | 40.8 | 122 | 83.3 | 14.1 | 2.4 | 30.5 | 53.4 | 11 | 20.6 | 35.4 | 40.4 | 59.4 | 67.4 | 72.4 | 79.4 | 111.4 |
| G2 | Datunxiaoheidou | Hebei,China | 39.2 | 116.3 | 103.1 | 15.6 | 3.2 | 72.4 | 105.3 | 12 | 11.3 | 53.1 | 62.1 | 69.1 | 76.1 | 82.1 | 88.1 | 113.1 |
| G3 | Ken 04-8579 | Heilongjiang,China | 48.3 | 128.1 | 57.4 | 13.2 | 0.8 | 31.5 | 70.8 | 9.7 | 13.8 | 32.9 | 42.9 | 45.9 | 55.9 | 61.9 | 71.9 | 91.9 |
| G4 | Kangxian2 | Heilongjiang,China | 48.3 | 128.1 | 96.8 | 15.4 | 1.7 | 35.6 | 76.5 | 13.1 | 17.4 | 33 | 42.7 | 48.7 | 60.7 | 66.7 | 76.7 | 91.7 |
| G5 | Liushitianhuancang | Liaoning,China | 40.8 | 122 | 107.2 | 15.2 | 1.4 | 54 | 90 | 12.9 | 14 | 34.5 | 43.5 | 47.5 | 57.5 | 63.5 | 73.5 | 111.5 |
| G6 | Tiedou 58 | Liaoning,China | 40.8 | 122 | 96.6 | 19.3 | 3.2 | 79.8 | 127.9 | 24.5 | 19.2 | 52 | 61 | 69 | 77 | 81 | 87 | 114 |
| G7 | Zhonghuang 20 | Beijing,China | 40.1 | 116.3 | 94.2 | 15.4 | 1.7 | 39 | 79.4 | 15.9 | 20.2 | 32.6 | 46.6 | 60.6 | 67.6 | 77.6 | 81.6 | 114.6 |
| G8 | Jichanghuangdou 1 | Xinjiang,China | 41.2 | 81.4 | 100.1 | 14.2 | 1.8 | 36.1 | 73.4 | 12 | 16.6 | 50.3 | 57.3 | 65.3 | 74.3 | 79.3 | 83.3 | 119 |
| G9 | Longquandadou | Heilongjiang,China | 48.3 | 128.1 | 81 | 15.7 | 3.2 | 56.2 | 101.3 | 18.7 | 26.1 | 36.2 | 45.2 | 61.2 | 70.2 | 72.2 | 79.6 | 106.2 |
| G10 | Xiaobaiqi | Liaoning,China | 40.8 | 122 | 108.9 | 18.5 | 6.8 | 83.7 | 143.9 | 25.2 | 16.7 | 47.3 | 55.3 | 61.3 | 68.3 | 74.3 | 81.3 | 103.3 |
| G11 | Gongye 04L-141 | Jilin,China | 43.4 | 126.3 | 94.4 | 15.7 | 1.1 | 29.4 | 60.8 | 6.4 | 10.8 | 37.4 | 49.4 | 60.4 | 67.4 | 71.4 | 77.4 | 105.4 |
| G12 | Tiedou 52 | Liaoning,China | 40.8 | 122 | 76.8 | 15.5 | 0.6 | 34.4 | 75.2 | 18.1 | 24.3 | 33.6 | 42.6 | 50.6 | 61.6 | 76.6 | 80.6 | 119 |
| G13 | L-57 | America | 43.4 | 79.3 | 89.3 | 15.6 | 2.1 | 27.5 | 45.2 | 8.2 | 18.5 | 34.9 | 43.9 | 48.9 | 61.9 | 67.9 | 77.9 | 105.9 |
| G14 | He 05-31 | Heilongjiang,China | 48.3 | 128.1 | 78.4 | 13.9 | 0.7 | 34.3 | 64 | 9.4 | 15 | 34.4 | 41.4 | 47.4 | 58.4 | 65.4 | 74.4 | 94.4 |
| G15 | L-21 | Canada | 43.4 | 79.3 | 78.7 | 16.6 | 4.1 | 55.8 | 104.9 | 17.3 | 29.7 | 32.5 | 42.5 | 47.5 | 54.5 | 61.5 | 68.5 | 95.5 |
| G16 | Hongfeng11 | Heilongjiang,China | 48.3 | 128.1 | 59.2 | 13.6 | 0.3 | 34.4 | 57.1 | 9.9 | 17.3 | 32.7 | 43.7 | 50.7 | 55.7 | 63.7 | 72.7 | 87.7 |
| G17 | Heinong 33 | Heilongjiang,China | 48.3 | 128.1 | 79.2 | 15.3 | 1.1 | 33.5 | 59.2 | 11.8 | 20.3 | 33.1 | 42.1 | 48.1 | 58.1 | 64.1 | 76.1 | 102.1 |
| G18 | Liao 98072 | Liaoning,China | 40.8 | 122 | 82.3 | 14 | 2.5 | 43.6 | 83.9 | 18.1 | 21.6 | 33.6 | 50.6 | 59.6 | 67.6 | 73.6 | 79.6 | 109.6 |
| G19 | Kenfeng 22 | Heilongjiang,China | 48.3 | 128.1 | 63.1 | 14.8 | 1.1 | 46.7 | 89.4 | 15.5 | 18.1 | 32.1 | 42.1 | 48.1 | 57.1 | 63.1 | 72.1 | 93.1 |
| G20 | Jiyu 89 | Jilin,China | 43.4 | 126.3 | 67.7 | 13.8 | 1.8 | 32.8 | 51.5 | 9.3 | 18.4 | 34.3 | 42.3 | 52.3 | 61.3 | 69.3 | 83.3 | 106.3 |
| G21 | Tiedou 51 | Liaoning,China | 40.8 | 122 | 81.6 | 14.1 | 2.9 | 51.6 | 95 | 20.8 | 31 | 35.4 | 41.4 | 50.4 | 61.4 | 66.4 | 71.4 | 109.4 |
| G22 | Tongnong 13 | Jilin,China | 43.4 | 126.3 | 98.9 | 14.5 | 0.3 | 23.8 | 41.3 | 10.3 | 25.5 | 35.6 | 45.6 | 59.6 | 69.6 | 74.6 | 78.6 | 114.6 |
| G23 | Bei 1873 | Heilongjiang,China | 48.3 | 128.1 | 60.7 | 13.9 | 1.8 | 45.7 | 78.3 | 14.5 | 18.7 | 33.8 | 42.8 | 49.8 | 56.8 | 63.8 | 76.8 | 94.8 |
| G24 | Hefeng 50 | Heilongjiang,China | 48.3 | 128.1 | 67.1 | 14.6 | 0.2 | 32.8 | 54.7 | 14.8 | 22.1 | 32.4 | 41.4 | 48.4 | 55.4 | 60.4 | 68.4 | 88.4 |
| G25 | Sui 02-339 | Heilongjiang,China | 48.3 | 128.1 | 74.7 | 15.1 | 3.3 | 47.7 | 96.9 | 22 | 23.4 | 35.9 | 43.9 | 49.9 | 61.9 | 67.9 | 76.9 | 93.9 |
| G26 | Hujiao 03-286 | Inner Mongolia,China | 44.5 | 111.7 | 54 | 11.6 | 1.1 | 40.3 | 78.6 | 11.6 | 14.8 | 32 | 41 | 46 | 55 | 61 | 68 | 86 |
| G27 | Heinong 44 | Heilongjiang,China | 48.3 | 128.1 | 70.3 | 13.6 | 0.7 | 42.4 | 76.6 | 13.1 | 16.9 | 33.1 | 42.1 | 47.2 | 54.1 | 62.1 | 76.1 | 105.1 |
| G28 | Hujiao 04-528 | Inner Mongolia,China | 44.5 | 111.7 | 59 | 13.1 | 1.2 | 45.7 | 73.6 | 11.8 | 16 | 34.4 | 41.4 | 46.4 | 55.4 | 60.4 | 68.4 | 80.4 |
| G29 | Fengshou 10 | Heilongjiang,China | 48.3 | 128.1 | 75.3 | 15.1 | 2.6 | 50.9 | 77.7 | 14 | 18.2 | 33.5 | 43.5 | 48.5 | 55.5 | 61.5 | 76.5 | 86.5 |
| G30 | Hersong 2 | Ukraine | 50.3 | 30.3 | 89.8 | 13.8 | 2.3 | 34.6 | 59.2 | 10.4 | 18.6 | 37.8 | 45.8 | 60.8 | 67.8 | 72.8 | 79.8 | 98.8 |
| G31 | Jinzhou 4-1 | Liaoning,China | 40.8 | 122 | 89.1 | 15.8 | 1.4 | 36 | 48.7 | 11.4 | 23.4 | 46.8 | 57.8 | 63.8 | 73.8 | 77.8 | 82.8 | 119 |
| G32 | Suinong 14 | Heilongjiang,China | 48.3 | 128.1 | 81.2 | 15.4 | 1.1 | 46.2 | 91.1 | 17.7 | 19.3 | 35.7 | 43.7 | 49.7 | 61.7 | 69.7 | 78.7 | 100.7 |
| G33 | Tiejiasilihuang | Jilin,China | 43.4 | 126.3 | 92.7 | 13.7 | 2.1 | 43.3 | 95.1 | 16.7 | 17.7 | 33.7 | 47.7 | 55.7 | 63.7 | 68.7 | 81.7 | 108.7 |
| G34 | Tiefeng 8 | Liaoning,China | 40.8 | 122 | 112.5 | 20.4 | 4.4 | 61.4 | 127.6 | 19.4 | 15.2 | 64.5 | 75.5 | 80.5 | 84.5 | 89.5 | 99.5 | 120.3 |
| G35 | Silihuang | Jilin,China | 43.4 | 126.3 | 79.3 | 11.8 | 0.5 | 21.1 | 36 | 6.7 | 18.9 | 33.6 | 43.6 | 56.6 | 66.6 | 73.6 | 79.6 | 88.6 |
| G36 | Bei 1361 | Heilongjiang,China | 48.3 | 128.1 | 72.7 | 13.7 | 0.5 | 25.8 | 51.4 | 9.9 | 19.3 | 34.1 | 44.1 | 49.1 | 56.1 | 64.1 | 73.1 | 91.1 |
| G37 | L-5 | Canada | 43.4 | 79.3 | 79.3 | 17.4 | 3.8 | 54.3 | 84.2 | 16.5 | 19.8 | 42.3 | 47.3 | 60.3 | 68.3 | 75.3 | 82.3 | 116.3 |
| G38 | Maoyandou | Gansu, China | 32.3 | 92.1 | 75.3 | 12.5 | 2.7 | 34.2 | 45.3 | 10 | 24.6 | 43.7 | 45.8 | 48.8 | 57.8 | 65.8 | 76.8 | 99.8 |
| G39 | Fengdihuang | Jilin,China | 43.4 | 126.3 | 87.3 | 15.5 | 3.6 | 39 | 53.6 | 10.3 | 19.3 | 45.5 | 58.5 | 67.5 | 76.5 | 81.5 | 84.5 | 112.5 |
| G40 | Tiedou 54 | Liaoning,China | 40.8 | 122 | 78.8 | 14.7 | 3.3 | 55.3 | 104.4 | 21.4 | 30.8 | 43.7 | 50.7 | 58.7 | 66.3 | 72.7 | 77.7 | 111.7 |
| G41 | Jiunong 20 | Jilin,China | 43.4 | 126.3 | 86.2 | 16.3 | 0.9 | 37.1 | 62.9 | 12.9 | 20.6 | 32.5 | 42.5 | 48.5 | 63.5 | 68.5 | 75.5 | 98.5 |
| G42 | Duludou | Jilin,China | 43.4 | 126.3 | 70.2 | 13.1 | 1.9 | 43.1 | 77.7 | 13.1 | 16.8 | 33.5 | 43.5 | 50.5 | 59.5 | 63.5 | 75.5 | 88.5 |
| G43 | Sui 03-3046 | Heilongjiang,China | 48.3 | 128.1 | 63.4 | 11.7 | 0.5 | 32.9 | 48.5 | 7.3 | 15.5 | 34.3 | 41.3 | 48.3 | 55.3 | 61.3 | 69.3 | 89.3 |
| G44 | Hefeng 47 | Heilongjiang,China | 48.3 | 128.1 | 65.9 | 13.1 | 0.1 | 33.2 | 55.8 | 10.9 | 19.2 | 35.3 | 43.3 | 47.3 | 57.3 | 64.3 | 78.3 | 104.3 |
| G45 | V111-4 | Jilin,China | 43.4 | 126.3 | 87.5 | 15.7 | 0.5 | 38 | 66.7 | 14 | 20.8 | 34.1 | 44.1 | 60.1 | 67.1 | 71.1 | 77.1 | 108.1 |
| G46 | Longxuan 1 | Heilongjiang,China | 48.3 | 128.1 | 88.9 | 14.4 | 0.1 | 30 | 61.8 | 11.2 | 18.1 | 34.4 | 42.4 | 48.4 | 59.4 | 66.4 | 72.4 | 88.4 |
| G47 | Dongnong 8004 | Heilongjiang,China | 48.3 | 128.1 | 58.8 | 13.4 | 3.6 | 42.2 | 98 | 16 | 15.5 | 30.5 | 42.5 | 48.5 | 56.5 | 64.5 | 74.5 | 96.5 |
| G48 | Sui 03-3952 | Heilongjiang,China | 48.3 | 128.1 | 75.8 | 11.8 | 0.5 | 28.9 | 45 | 17.6 | 29.1 | 33.4 | 41.4 | 47.4 | 54.4 | 61.4 | 69.4 | 89.4 |
| G49 | Hefeng 25 | Heilongjiang,China | 48.3 | 128.1 | 76.2 | 15.7 | 3.6 | 45.5 | 73.6 | 13.1 | 18.3 | 34.7 | 42.7 | 50.7 | 58.7 | 65.7 | 77.7 | 88.7 |
| G50 | Liaodou 3 | Liaoning,China | 40.8 | 122 | 101.7 | 17.8 | 4.2 | 67 | 119.9 | 24.8 | 19.8 | 36.9 | 47.9 | 58.9 | 67.9 | 72.9 | 76.9 | 106.9 |
| G51 | Hujiao 423 | Inner Mongolia,China | 44.5 | 111.7 | 62.6 | 12.9 | 2.1 | 45.4 | 94.4 | 16.4 | 18.5 | 35.1 | 43.1 | 51.1 | 61.1 | 65.1 | 77.1 | 97.1 |
| G52 | Liaoxian 1 | Liaoning,China | 40.8 | 122 | 35.3 | 8.6 | 4 | 32.3 | 44.4 | 15.9 | 30.8 | 33 | 44 | 52 | 59 | 65 | 76 | 104 |
| G53 | Zhongpin 95-5388 | Beijing,China | 40.1 | 116.3 | 109.4 | 21.9 | 3.8 | 66.8 | 128.9 | 22.6 | 17.5 | 50.3 | 61.3 | 69.3 | 75.3 | 87.3 | 98.3 | 119 |
| G54 | Heihe 45 | Heilongjiang,China | 48.3 | 128.1 | 43.6 | 12.8 | 1.7 | 40.1 | 109.9 | 19.9 | 18.2 | 31.5 | 39.5 | 45.5 | 50.5 | 59.5 | 68.5 | 89.5 |
| G55 | L-59 Peking | America | 41.8 | 92.9 | 109 | 18 | 6.8 | 82.2 | 141 | 18.8 | 10.9 | 76.9 | 81.9 | 84.9 | 89.9 | 93.9 | 99.9 | 117.9 |
| G56 | Sui 04-5804 | Heilongjiang,China | 48.3 | 128.1 | 75.8 | 14.3 | 0.2 | 35.2 | 60.4 | 11.3 | 19 | 34.7 | 40.7 | 46.7 | 58.7 | 69.7 | 74.7 | 89.7 |
| G57 | Ji 06B7 | Hebei,China | 39.2 | 116.3 | 118.4 | 22.4 | 4.8 | 67.8 | 135.4 | 20.8 | 15.4 | 69.5 | 77.5 | 84.5 | 92.5 | 97.5 | 109 | 127 |
| G58 | Marca. Joe lisa | Heilongjiang,China | 48.3 | 128.1 | 79 | 16.6 | 2.6 | 53.4 | 95.5 | 10.4 | 11.2 | 51 | 60 | 67 | 78 | 82 | 86 | 106 |
| G59 | Jihuang 13 | Hebei,China | 39.2 | 116.3 | 80.9 | 16.2 | 1.8 | 28.4 | 58.9 | 13.1 | 22.2 | 39.4 | 50.4 | 59.4 | 75.4 | 78.4 | 82.4 | 117.4 |
| G60 | L-28 | Canada | 43.4 | 79.3 | 70.3 | 12.9 | 2.6 | 34.8 | 62.4 | 8.8 | 15.5 | 32.8 | 41.8 | 48.8 | 54.8 | 60.8 | 73.8 | 90.8 |
| G61 | Beifeng 9 | Heilongjiang,China | 48.3 | 128.1 | 71.3 | 12.6 | 0.3 | 28.3 | 50.2 | 7.8 | 15.7 | 32.3 | 42.3 | 47.3 | 53.3 | 61.3 | 71.3 | 91.3 |
| G62 | Zhongzuo 00-683 | Beijing,China | 40.1 | 116.3 | 91.5 | 18.6 | 5.7 | 52.2 | 103.1 | 20.2 | 19.6 | 33.4 | 46.4 | 58.4 | 66.4 | 74.4 | 86.4 | 108.4 |
| G63 | Dongnong 50 | Heilongjiang,China | 48.3 | 128.1 | 79.4 | 17.4 | 5.4 | 79.2 | 137.1 | 12.2 | 7.3 | 33.3 | 43.3 | 49.3 | 55.3 | 64.3 | 75.3 | 90.3 |
| G64 | Dongnong 93-046 | Heilongjiang,China | 48.3 | 128.1 | 78.4 | 13.8 | 1.4 | 27.5 | 45.4 | 8.7 | 19.2 | 33.4 | 44.4 | 50.4 | 56.4 | 64.4 | 73.4 | 88.4 |
| G65 | Suinong 10 | Heilongjiang,China | 48.3 | 128.1 | 72.2 | 16.1 | 3.4 | 65 | 124.9 | 24.1 | 17.6 | 32.3 | 39.3 | 46.3 | 62.3 | 67.3 | 77.3 | 94.3 |
| G66 | Tiefeng 31 | Liaoning,China | 40.8 | 122 | 72.7 | 16.5 | 2.1 | 57.4 | 114.2 | 23.4 | 22.3 | 36.6 | 46.6 | 60.6 | 71.6 | 73.6 | 90.6 | 121.2 |
| G67 | Dongnong 49 | Heilongjiang,China | 48.3 | 128.1 | 51.6 | 11.6 | 0.2 | 29.9 | 57.2 | 8.9 | 15.6 | 33.5 | 42.5 | 48.5 | 53.5 | 59.5 | 68.5 | 89.5 |
| G68 | Huayou 446 | Hebei,China | 39.2 | 116.3 | 94.3 | 16.2 | 1.3 | 31.8 | 58.4 | 13.6 | 23.1 | 46.8 | 55.8 | 59.8 | 66.8 | 71.8 | 79.8 | 97.8 |
| G69 | Heihe 48 | Heilongjiang,China | 48.3 | 128.1 | 60.7 | 12.3 | 0.2 | 32 | 55.8 | 8 | 14.5 | 34.2 | 43.2 | 47.2 | 54.2 | 61.2 | 73.2 | 81.2 |
| G70 | Huajiang 2 | Heilongjiang,China | 48.3 | 128.1 | 47.7 | 12.5 | 0.6 | 30.4 | 63.8 | 9.5 | 14.9 | 32.4 | 41.4 | 48.4 | 55.4 | 63.4 | 70.4 | 81.4 |
| G71 | Suinong 29 | Heilongjiang,China | 48.3 | 128.1 | 72.8 | 14.3 | 0.1 | 25.4 | 48.2 | 9.2 | 19.6 | 33.4 | 41.4 | 47.4 | 55.4 | 61.4 | 70.4 | 91.4 |
| G72 | Hefeng 35 | Heilongjiang,China | 48.3 | 128.1 | 68.9 | 15.8 | 0.9 | 41.8 | 82 | 16.3 | 19.7 | 33.7 | 45.7 | 50.7 | 58.7 | 63.7 | 74.7 | 94.7 |
| G73 | Suinong 8 | Heilongjiang,China | 48.3 | 128.1 | 82.3 | 16 | 3.2 | 61.7 | 98.4 | 18 | 18.3 | 37.4 | 42.4 | 47.4 | 61.4 | 73.4 | 78.4 | 89.4 |
| G74 | Jilin 47 | Jilin,China | 43.4 | 126.3 | 75 | 15.2 | 1.4 | 55.9 | 111.7 | 23.2 | 29.1 | 31.9 | 44.1 | 56.1 | 64.1 | 72.1 | 84.1 | 105.1 |
| G75 | Heinong 48 | Heilongjiang,China | 48.3 | 128.1 | 70.3 | 14.3 | 0.9 | 39.8 | 75.4 | 18.3 | 24.3 | 34.4 | 43.4 | 48.4 | 56.4 | 62.4 | 71.4 | 98.4 |
| G76 | Nova | Italy | 41.5 | 12.3 | 101 | 16.1 | 0.9 | 36.4 | 70.7 | 17.5 | 24.7 | 35.2 | 44.2 | 52.2 | 62.2 | 68.2 | 76.2 | 101.2 |
| G77 | Beidou 14 | Heilongjiang,China | 48.3 | 128.1 | 69 | 15.3 | 1.5 | 43 | 86.9 | 14 | 16.2 | 35.4 | 43.4 | 50.4 | 57.4 | 62.4 | 71.4 | 88.4 |
| G78 | Kangxian 3 | Heilongjiang,China | 48.3 | 128.1 | 101.1 | 14.7 | 0.9 | 23.3 | 41 | 8.6 | 21 | 34.5 | 42.5 | 49.5 | 61.5 | 67.5 | 78.5 | 106.5 |
| G79 | Dongnong 42 | Heilongjiang,China | 48.3 | 128.1 | 92.2 | 14.7 | 0.9 | 29.8 | 57.3 | 11.8 | 21 | 35.3 | 46.3 | 52.3 | 65.3 | 69.3 | 78.3 | 92.3 |
| G80 | Kenjian 23 | Heilongjiang,China | 48.3 | 128.1 | 67.5 | 15.6 | 1.7 | 46.1 | 100.4 | 19 | 18.9 | 34.5 | 43.5 | 49.5 | 55.5 | 63.5 | 84.5 | 89.5 |
| G81 | Heinong 55 | Heilongjiang,China | 48.3 | 128.1 | 59 | 13.8 | 0.8 | 39.9 | 79.7 | 17.4 | 22 | 32.4 | 41.4 | 48.4 | 55.4 | 61.4 | 70.4 | 98.4 |
| G82 | Hefeng 45 | Heilongjiang,China | 48.3 | 128.1 | 76.8 | 15.8 | 1.2 | 29.7 | 55.4 | 10.4 | 18.8 | 33.5 | 41.5 | 47.5 | 55.5 | 61.5 | 70.5 | 94.5 |
| G83 | Kenfeng 15 | Heilongjiang,China | 48.3 | 128.1 | 51.5 | 13.8 | 0.5 | 53.8 | 126.9 | 21.2 | 15.2 | 34.1 | 42.1 | 49.1 | 54.1 | 60.1 | 76.1 | 91.1 |
| G84 | Chasedou | Jilin,China | 43.4 | 126.3 | 92.3 | 16.6 | 1.4 | 49.3 | 95.5 | 16.9 | 17.6 | 33.7 | 46.7 | 59.7 | 65.7 | 71.7 | 77.7 | 102.7 |
| G85 | He 05-991 | Heilongjiang,China | 48.3 | 128.1 | 53.4 | 12.5 | 0.4 | 27.8 | 48.4 | 7 | 14.7 | 33.6 | 42.6 | 45.6 | 54.6 | 60.6 | 71.6 | 85.6 |
| G86 | Xiaolimoshidou | Heilongjiang,China | 39.2 | 116.3 | 82 | 19.2 | 5.7 | 83.9 | 142.7 | 20.6 | 14.4 | 35.5 | 47.5 | 53.5 | 63.5 | 72.5 | 78.5 | 98.5 |
| G87 | Jingshanpu | Heilongjiang,China | 48.3 | 128.1 | 77.3 | 14.1 | 2.5 | 39.7 | 78.3 | 12.5 | 16 | 34.9 | 47.9 | 50.9 | 62.9 | 67.9 | 78.9 | 99.9 |
| G88 | Qinganheidou | Heilongjiang,China | 48.3 | 128.1 | 91.5 | 16.4 | 2.1 | 49.7 | 98.5 | 16.7 | 17.1 | 37 | 49 | 61 | 69 | 73 | 85 | 111 |
| G89 | Dongnong594 | Heilongjiang,China | 48.3 | 128.1 | 64.6 | 15.1 | 0.9 | 45.3 | 77.1 | 16.7 | 22 | 33.9 | 43.9 | 47.9 | 55.9 | 66.9 | 76.9 | 110.9 |
| G90 | Yapoche | Heilongjiang,China | 48.3 | 128.1 | 92.5 | 16.5 | 0.7 | 35.5 | 48.2 | 8.5 | 17.6 | 32.6 | 48.6 | 55.6 | 61.6 | 66.6 | 76.6 | 98.6 |
| G91 | Kennong 29 | Heilongjiang,China | 48.3 | 128.1 | 60.6 | 13.8 | 0.1 | 34.8 | 53.8 | 12.8 | 24.1 | 32.2 | 39.2 | 45.3 | 54.2 | 62.2 | 72.2 | 91.2 |
| G92 | Heimoshidou | Jilin,China | 43.4 | 126.3 | 117.3 | 17 | 3.6 | 81.8 | 120.7 | 17.9 | 12.8 | 55.5 | 61.5 | 67.5 | 76.5 | 83.5 | 89.5 | 112.5 |
| G93 | Ha 04-1824 | Heilongjiang,China | 48.3 | 128.1 | 64.8 | 13.2 | 1.1 | 30.9 | 58.4 | 11.3 | 19.9 | 35 | 44 | 48 | 55 | 62 | 73 | 88 |
| G94 | Fangzhengmoshidou | Heilongjiang,China | 48.3 | 128.1 | 84 | 14.4 | 3.9 | 48.3 | 62.3 | 8.4 | 13.5 | 35.3 | 45.3 | 61.3 | 69.3 | 76.3 | 81.3 | 108.3 |
| G95 | Helongyoutai | Jilin,China | 43.4 | 126.3 | 77.8 | 12.5 | 3.6 | 41.4 | 63 | 9.8 | 15.7 | 34.1 | 43.1 | 49.1 | 57.1 | 63.1 | 74.1 | 88.1 |
| G96 | Kexin 3 | Beijing,China | 40.1 | 116.3 | 83.1 | 14.3 | 2.9 | 38.1 | 59.3 | 14.6 | 24.7 | 58.4 | 66.4 | 70.4 | 76.4 | 81.4 | 85.4 | 110.4 |
| G97 | Yuanbaojin | Heilongjiang,China | 48.3 | 128.1 | 83.8 | 13.6 | 3.9 | 54.4 | 90.3 | 15.5 | 16.8 | 32.5 | 43.5 | 48.5 | 55.5 | 61.5 | 71.5 | 93.5 |
| G98 | Zhongzuo J4133 | Beijing,China | 40.1 | 116.3 | 81.3 | 15.6 | 1.4 | 39.9 | 75.3 | 15.2 | 20.3 | 48.7 | 57.7 | 67.7 | 75.7 | 83.7 | 93.7 | 119 |
| G99 | Jilin 30 | Jilin,China | 43.4 | 126.3 | 86.7 | 14.7 | 1.4 | 31.6 | 71.1 | 13.4 | 18.6 | 32.9 | 40.9 | 57.9 | 66.9 | 78.9 | 86.9 | 119 |
| G100 | Ji 100 | Jilin,China | 43.4 | 126.3 | 86 | 13.4 | 0.2 | 32.8 | 57.3 | 10.9 | 19.6 | 33.4 | 44.4 | 50.4 | 60.4 | 68.4 | 77.4 | 90.4 |
| G101 | Suinong 30 | Heilongjiang,China | 48.3 | 128.1 | 63.2 | 14.9 | 1.2 | 44.9 | 102.3 | 17.3 | 16.9 | 32.8 | 43.8 | 48.8 | 55.8 | 61.8 | 71.8 | 87.8 |
| G102 | Boige du | Germany | 52.3 | 13.2 | 79.9 | 12.6 | 3.2 | 46.8 | 83.7 | 10.2 | 12.4 | 33.3 | 38.3 | 44.3 | 62.3 | 67.3 | 75.3 | 90 |
| G103 | Beidou 16 | Heilongjiang,China | 48.3 | 128.1 | 94.7 | 17.2 | 2.5 | 47.2 | 79.7 | 16.4 | 20.5 | 36.8 | 41.8 | 58.8 | 63.8 | 70.8 | 80.8 | 114.8 |
| G104 | Kebei 1 | Heilongjiang,China | 48.3 | 128.1 | 71 | 14.6 | 0.9 | 41.9 | 69.1 | 11.3 | 16.3 | 34.5 | 42.5 | 48.5 | 61 | 65 | 76 | 91.5 |
| G105 | Mengdou 21 | Inner Mongolia,China | 44.5 | 111.7 | 56.5 | 13.3 | 0.7 | 44 | 95 | 16.1 | 17 | 32.5 | 42.5 | 46.5 | 55.5 | 60.5 | 72.5 | 91.5 |
| G106 | Heihe 38 | Heilongjiang,China | 48.3 | 128.1 | 57.3 | 13.1 | 1 | 36.2 | 57.3 | 9.9 | 17.4 | 30.7 | 39.6 | 46.7 | 55.6 | 61.6 | 73.7 | 91.8 |
| G107 | Zhongdou 35 | Beijing,China | 40.1 | 116.3 | 80.9 | 16 | 3.3 | 48.8 | 93.9 | 19.9 | 21.4 | 33.7 | 41.7 | 47.7 | 61.7 | 70.7 | 76.7 | 113.7 |
| G108 | Zhonghuang 6 | Beijing,China | 40.1 | 116.3 | 87.7 | 15.7 | 4.1 | 44.3 | 60.4 | 12 | 29.8 | 51.2 | 60.2 | 69.2 | 77.2 | 80.2 | 85.2 | 116.4 |
| G109 | Jiyu 94 | Jilin,China | 43.4 | 126.3 | 76.7 | 15.4 | 3 | 61.4 | 102.2 | 21.5 | 21.2 | 51.5 | 60.5 | 68.5 | 76.5 | 82.5 | 86.5 | 116.6 |
| G110 | Dongnong 44 | Heilongjiang,China | 48.3 | 128.1 | 53.5 | 13.6 | 1 | 36.3 | 49.3 | 7.4 | 21 | 29.5 | 41.5 | 47.5 | 53.5 | 61.5 | 66.5 | 74.5 |
| G111 | Liaonong 2 | Liaoning,China | 40.8 | 122 | 67.8 | 13 | 2.9 | 52.4 | 94.9 | 18 | 19.6 | 33.5 | 46.5 | 60.5 | 66.5 | 71.5 | 77.5 | 113.5 |
| G112 | Bei 1484 | America | 48.3 | 128.1 | 62.2 | 13.3 | 1.3 | 27.8 | 44.2 | 11.3 | 24 | 30.8 | 41.8 | 45.8 | 55.8 | 61.8 | 73.8 | 87.8 |
| G113 | Williams82 | America | 41.8 | 92.9 | 76.2 | 14.2 | 2 | 35.6 | 61.5 | 11.6 | 19.1 | 33.4 | 42.4 | 53.4 | 66.4 | 76.4 | 81.4 | 109.4 |
| G114 | Jindou 33 | Liaoning,China | 40.8 | 122 | 109.6 | 17.8 | 0.8 | 53 | 77.6 | 19.5 | 25.2 | 51.6 | 62.6 | 67.6 | 76.6 | 80.6 | 84.6 | 102.6 |
| G115 | Kenfeng 18 | Heilongjiang,China | 48.3 | 128.1 | 74.9 | 14.1 | 1 | 27.3 | 46.2 | 9.1 | 19.6 | 32.4 | 41.4 | 45.4 | 57.4 | 61.4 | 76.4 | 107.4 |
| G116 | Suinong 20 | Heilongjiang,China | 48.3 | 128.1 | 73 | 15.9 | 1.2 | 41.4 | 77.1 | 13.3 | 17.4 | 33.5 | 41.5 | 48.5 | 55.5 | 61.5 | 73.5 | 94.5 |
| G117 | Mengdou 9 | Inner Mongolia,China | 44.5 | 111.7 | 51.2 | 11.5 | 0.7 | 35.2 | 60.7 | 9.7 | 21.9 | 35.2 | 43.2 | 48.2 | 55.2 | 61.2 | 73.2 | 83.2 |
| G118 | L-9 | America | 41.8 | 92.9 | 122.2 | 26.4 | 5.3 | 60.3 | 136.8 | 19.2 | 14 | 67.5 | 75.5 | 82.5 | 90.5 | 94.5 | 106.4 | 127.6 |
| G119 | Suinong 1 | Heilongjiang,China | 48.3 | 128.1 | 77.1 | 14.7 | 1.5 | 38.6 | 76 | 13.5 | 18.1 | 33 | 43 | 49 | 57 | 62 | 74 | 90 |
| G120 | Kenfeng 14 | Heilongjiang,China | 48.3 | 128.1 | 66.1 | 13.9 | 1.5 | 29.4 | 62.1 | 11.5 | 18.7 | 35 | 45 | 51 | 59 | 65 | 78 | 106 |
| G121 | Tiejiazi | Liaoning,China | 40.8 | 122 | 85.7 | 14.5 | 0.5 | 26.2 | 48.2 | 10.3 | 22.3 | 34.2 | 43.2 | 48.2 | 63.2 | 69.2 | 79.2 | 102.2 |
| G122 | Suinong 4 | Heilongjiang,China | 48.3 | 128.1 | 64.8 | 14.4 | 1.6 | 31.9 | 50.7 | 8.8 | 17.6 | 33.3 | 43.3 | 49.3 | 59.3 | 64.3 | 71.3 | 90.3 |
| G123 | Jinshanchamoshidou | Jilin,China | 43.4 | 126.3 | 111.3 | 14.8 | 1.2 | 53.8 | 75.8 | 8.7 | 11.7 | 71.5 | 76 | 79 | 81 | 86 | 97 | 118.4 |
| G124 | Longpin 03-311 | Heilongjiang,China | 48.3 | 128.1 | 79.3 | 15.5 | 0.4 | 40.2 | 75 | 13.7 | 18.3 | 30.7 | 41.7 | 46.7 | 54.7 | 61.7 | 69 | 88.7 |
| G125 | Jilinchalihua | Jilin,China | 43.4 | 126.3 | 80.7 | 16.4 | 6.3 | 66.1 | 86.4 | 17.1 | 8.1 | 58.6 | 67.6 | 76.6 | 80.6 | 84.6 | 87.6 | 113.6 |
| G126 | Heinong 37 | Heilongjiang,China | 48.3 | 128.1 | 66.7 | 14.5 | 0.2 | 34 | 61.8 | 11.8 | 19.1 | 32.4 | 42.4 | 45.4 | 53.4 | 60.4 | 82.4 | 100.4 |
| G127 | Huajiang 4403 | Heilongjiang,China | 48.3 | 128.1 | 68 | 15 | 0.9 | 38.4 | 71.6 | 13.3 | 18.8 | 32.6 | 39.5 | 47.5 | 59.5 | 63.5 | 74.5 | 91.5 |
| G128 | Huangbaozhu | Liaoning,China | 40.8 | 122 | 102.1 | 13 | 2 | 31.8 | 68.2 | 12.7 | 18.7 | 40.9 | 52.9 | 61.9 | 67.9 | 74.9 | 81.9 | 101.9 |
| G129 | Suinong 25 | Heilongjiang,China | 48.3 | 128.1 | 82 | 17.2 | 1.2 | 44.3 | 71.8 | 12.8 | 17.7 | 33.6 | 41.6 | 48.6 | 54.6 | 62.6 | 81.6 | 95.6 |
| G130 | Sui 05-7304 | Heilongjiang,China | 48.3 | 128.1 | 55.8 | 12.7 | 0.3 | 26.5 | 48.2 | 8.2 | 17.3 | 33.4 | 41.4 | 48.4 | 54.4 | 61.4 | 81.4 | 86.4 |
| G131 | Jidou 9 | Hebei,China | 39.2 | 116.3 | 77.5 | 15.5 | 1.7 | 32.3 | 69.4 | 18.5 | 26.5 | 36.3 | 47.3 | 60.3 | 70.3 | 74.3 | 82.3 | 118.3 |
| G132 | Mengdou 14 | Inner Mongolia,China | 44.5 | 111.7 | 69.5 | 13.7 | 2 | 52 | 99.7 | 15.3 | 15.3 | 32.8 | 42.1 | 48.1 | 57.1 | 62.1 | 76.1 | 95.1 |
| G133 | Handou 5 | Hebei,China | 39.2 | 116.3 | 84.2 | 14 | 2.2 | 38.3 | 79.1 | 21.3 | 26.7 | 40.7 | 48.7 | 59.7 | 67.7 | 71.7 | 77.7 | 113.7 |
| G134 | Jidou 17 | Hebei,China | 39.2 | 116.3 | 90.1 | 17.2 | 3.1 | 31.5 | 74.2 | 15 | 20.4 | 47.4 | 57.4 | 67.4 | 76.4 | 81.4 | 85.4 | 103.4 |
| G135 | Aika 166 | Heilongjiang,China | 44.2 | 26.1 | 59.1 | 13.1 | 2.2 | 34.4 | 57.5 | 9.5 | 16.4 | 32.5 | 43.5 | 50.5 | 59.5 | 66.5 | 75.5 | 92.5 |
| G136 | Sui 04-6018 | Heilongjiang,China | 48.3 | 128.1 | 69.6 | 15.9 | 0.5 | 44.1 | 72.5 | 12.7 | 29.5 | 31.9 | 42.9 | 48.9 | 59.9 | 63.9 | 73.9 | 92.9 |
| G137 | Dongnong 48 | Heilongjiang,China | 48.3 | 128.1 | 73.5 | 15.3 | 0.5 | 34.5 | 59.2 | 11.3 | 19.1 | 33.9 | 43.9 | 45.9 | 54.9 | 60.9 | 69.9 | 89.9 |
| G138 | L-79 | America | 43.4 | 79.3 | 67.1 | 14.5 | 2.7 | 45.5 | 88.5 | 13.5 | 15.6 | 33.8 | 43.8 | 47.8 | 55.8 | 63.8 | 72.8 | 92.5 |
| G139 | Zhonghuang 35 | Beijing,China | 40.1 | 116.3 | 70 | 14 | 2 | 40.3 | 63.1 | 12.1 | 19.3 | 34.1 | 44.1 | 49.1 | 56.1 | 61.1 | 76.1 | 95.1 |
| G140 | Kennong 30 | Heilongjiang,China | 48.3 | 128.1 | 68.6 | 14.4 | 1.2 | 33.9 | 59.5 | 10.7 | 18 | 33.6 | 42.6 | 47.6 | 55.6 | 61.6 | 73.6 | 98.6 |
| G141 | Zhonghuang 30 | Beijing,China | 40.1 | 116.3 | 75.2 | 15.9 | 1.9 | 42 | 85.2 | 19.7 | 23.1 | 43.8 | 53.8 | 60.8 | 69.8 | 77.8 | 83.8 | 94.8 |
| G142 | Baichengmoshidou | Jilin,China | 43.4 | 126.3 | 103 | 13.5 | 4.9 | 51.2 | 73.1 | 8 | 11.1 | 40 | 51 | 59 | 67 | 74 | 79 | 92 |
| G143 | L-10 | America | 43.4 | 79.3 | 97.6 | 17 | 2 | 48.9 | 98.8 | 19.1 | 21.6 | 32.8 | 41.8 | 47.8 | 65.8 | 71.8 | 80.8 | 113.8 |
| G144 | Zaoshu 18 | Beijing,China | 40.1 | 116.3 | 62 | 12.1 | 3.8 | 37.3 | 66.7 | 14.5 | 21.7 | 33 | 45 | 57 | 67 | 71 | 81 | 98 |
| G145 | Zhonghuang 10 | Beijing,China | 40.1 | 116.3 | 92.7 | 13.5 | 1.6 | 34.8 | 53.4 | 9.2 | 17.4 | 47.4 | 57.4 | 67.4 | 76.4 | 81.4 | 88.4 | 117.4 |
| G146 | Dongnong L-13 | Heilongjiang,China | 43.4 | 79.3 | 93.4 | 15.8 | 0.9 | 30.3 | 48.8 | 10.8 | 22.3 | 33.2 | 43.2 | 49.3 | 60.2 | 69.2 | 79.2 | 99.2 |
| G147 | Neifeng 15 | Heilongjiang,China | 48.3 | 128.1 | 80.3 | 14.3 | 2.9 | 47.7 | 85.3 | 15.4 | 18 | 33.3 | 42.3 | 47.3 | 58.3 | 64.3 | 76.3 | 92.3 |
| G148 | Dongnong 47 | Heilongjiang,China | 48.3 | 128.1 | 70.5 | 14.4 | 1.2 | 33.3 | 76.8 | 14.3 | 18.6 | 33.5 | 41.5 | 48.5 | 54.5 | 60.5 | 74.5 | 94.5 |
| G149 | Hefeng 29 | Heilongjiang,China | 48.3 | 128.1 | 82.8 | 14.2 | 2 | 45.6 | 85.4 | 17 | 20 | 33.4 | 40.4 | 52.4 | 60.4 | 68.4 | 77.4 | 99.4 |
| G150 | Dongnong 07-909 | Heilongjiang,China | 48.3 | 128.1 | 67.6 | 14.1 | 1.9 | 40.7 | 71.3 | 12.9 | 18.2 | 35 | 44 | 50 | 58 | 63 | 75 | 98 |
| G151 | Hefeng 52 | Heilongjiang,China | 48.3 | 128.1 | 60.6 | 13.9 | 0.5 | 42.5 | 72.7 | 12.6 | 17.3 | 35.3 | 45.3 | 50.3 | 57.3 | 62.3 | 74.3 | 92.3 |
| G152 | Heihe 18 | Heilongjiang,China | 48.3 | 128.1 | 41.5 | 10.9 | 1.2 | 42.3 | 76.5 | 14.1 | 17.3 | 32.8 | 42.8 | 47.8 | 54.8 | 62.8 | 75.8 | 90.8 |
| G153 | Neifeng 11 | Heilongjiang,China | 48.3 | 128.1 | 62.5 | 13 | 0.8 | 38.3 | 69.9 | 10.4 | 21 | 33.5 | 43.5 | 48.5 | 59.5 | 68.5 | 78.5 | 94.5 |
| G154 | Heihexiaohuangdou | Heilongjiang,China | 48.3 | 128.1 | 68.1 | 14 | 2.8 | 38.6 | 88.9 | 14.3 | 16.1 | 32.1 | 40.1 | 46.1 | 57.1 | 63.1 | 72.1 | 82.1 |
| G155 | Zhongdou 27 | Beijing,China | 40.1 | 116.3 | 82.5 | 14.4 | 2.7 | 31.6 | 49 | 11.1 | 22.7 | 41.4 | 50.4 | 60.4 | 66.4 | 72.4 | 76.4 | 104.4 |
| G156 | Bei 4834 | Heilongjiang,China | 48.3 | 128.1 | 89.3 | 16 | 2 | 42.3 | 41.9 | 7.9 | 23.1 | 35.5 | 42.5 | 47.5 | 55.5 | 63.5 | 81.5 | 105.5 |
| G157 | Heilongjiang 41 | Heilongjiang,China | 48.3 | 128.1 | 92.4 | 13.6 | 0.7 | 24.2 | 38.3 | 8.3 | 21.9 | 35.5 | 49.5 | 63 | 70.5 | 77.5 | 88.5 | 112.5 |
| G158 | Zhongpin 03-5179 | Beijing,China | 40.1 | 116.3 | 78.7 | 15.6 | 2.9 | 35.6 | 66.7 | 14.4 | 21.4 | 33.4 | 42.4 | 54.4 | 60.4 | 68.4 | 73.4 | 112.4 |
| G159 | Hefeng 37 | Heilongjiang,China | 48.3 | 128.1 | 58.8 | 12 | 1.4 | 53.1 | 80.2 | 13.4 | 16.9 | 64.3 | 77.3 | 82.3 | 87.3 | 92.3 | 99.3 | 126.1 |
| G160 | Dongnong 46 | Heilongjiang,China | 48.3 | 128.1 | 67.9 | 15 | 1.5 | 42.6 | 88.4 | 17.4 | 19.6 | 34.3 | 42.3 | 48.3 | 56.3 | 67.3 | 77.3 | 88.3 |
| G161 | Dongnong 43 | Heilongjiang,China | 48.3 | 128.1 | 70.3 | 14.2 | 3.6 | 34.4 | 55.9 | 10.3 | 18.6 | 33.9 | 41.9 | 47.9 | 57.9 | 63.9 | 74.9 | 89.2 |
| G162 | Dongnong 1068 | Heilongjiang,China | 48.3 | 128.1 | 75.8 | 14.6 | 2.3 | 47.2 | 91.5 | 19.3 | 21 | 33 | 42 | 47.1 | 60 | 67 | 77 | 87 |
| G163 | Dongnong 56 | Heilongjiang,China | 48.3 | 128.1 | 72.4 | 13.5 | 0.3 | 42.2 | 76.1 | 15 | 19.4 | 35.8 | 43.8 | 47.8 | 55.8 | 61.8 | 69.8 | 101.8 |
| G164 | Jiunong 21 | Jilin,China | 43.4 | 126.3 | 74.8 | 14.6 | 0.4 | 23.6 | 45.9 | 9.4 | 21 | 34.2 | 41.2 | 43.2 | 64.2 | 72.2 | 78.2 | 110.2 |
| G165 | Chamoshidou | Jilin,China | 43.4 | 126.3 | 89.8 | 18.8 | 6 | 78 | 116.2 | 19.8 | 17 | 58.5 | 64.5 | 75.5 | 79.5 | 84.5 | 91.5 | 118.5 |
| G166 | Daliheidou | Shandong,China | 36.2 | 118.3 | 77.7 | 13.8 | 3.7 | 31 | 55 | 12.8 | 23.7 | 33.6 | 43.6 | 49.6 | 55.6 | 62.6 | 76.6 | 107.6 |
| G167 | Zhongpin 03-5373 | Beijing,China | 40.1 | 116.3 | 85.6 | 16.4 | 2.3 | 46.4 | 69.5 | 13.6 | 20 | 56.1 | 64.1 | 69.1 | 75.1 | 78.1 | 84.1 | 117.6 |
| G168 | Wuxing 4 | Hebei,China | 39.2 | 116.3 | 106.8 | 22.5 | 4.7 | 70 | 140.7 | 22.5 | 16 | 61.5 | 67.5 | 76.5 | 83.5 | 93.5 | 106.4 | 128.9 |
| G169 | Hefeng 55 | Heilongjiang,China | 48.3 | 128.1 | 71.5 | 16 | 0.7 | 29.6 | 47 | 9.3 | 19.5 | 30.5 | 41.5 | 48.5 | 56.5 | 60.5 | 74.5 | 92.5 |
| G170 | Suinong 28 | Heilongjiang,China | 48.3 | 128.1 | 71.3 | 14.3 | 1.3 | 33.5 | 67.4 | 10.8 | 16 | 32 | 41 | 50 | 59 | 66 | 75 | 91 |
| G171 | Zhongpin 03-5334 | Beijing,China | 40.1 | 116.3 | 80.8 | 17.1 | 5.3 | 80.4 | 118.6 | 22.5 | 19.1 | 51.7 | 59.7 | 69.7 | 77.7 | 82.7 | 87.7 | 106.3 |
| G172 | Fengshou 6 | Heilongjiang,China | 48.3 | 128.1 | 77.7 | 13.7 | 1.9 | 42.2 | 85.9 | 12.3 | 14.4 | 31.7 | 42.7 | 47.7 | 57.7 | 66.7 | 76.7 | 86.7 |
| G173 | Dunajika | Russia | 61.5 | 98 | 81 | 12.7 | 3.2 | 45.3 | 76.6 | 7.7 | 10.3 | 32.6 | 42.6 | 48.6 | 55.6 | 62.6 | 70.6 | 88.6 |

| **Number** | **Accession** | **Origin** | **Latitude (°N)** | **Longitude (°W)** | **2016 Changchun** | | | | | | | | | | | | | |
| --- | --- | --- | --- | --- | --- | --- | --- | --- | --- | --- | --- | --- | --- | --- | --- | --- | --- | --- |
|  |  |  |  |  | **PH**  **(cm)** | **NMN** | **EBN** | **EPN** | **GN** | **GW**  **(g)** | **HGW**  **(g)** | **R1**  **(day)** | **R2**  **(day)**  **()** | **R3**  **(day)** | **R4**  **(day)** | **R5**  **(day)** | **R6**  **(day)** | **R7**  **(day)** |
| G1 | Tiedou 50 | Liaoning,China | 40.8 | 122 | 104 | 18 | 1.6 | 49 | 96.4 | 19.6 | 20.5 | 49.3 | 53.3 | 60.3 | 68.3 | 80.3 | 94.3 | 126.3 |
| G2 | Datun xiaoheidou | Hebei,China | 39.2 | 116.3 | 122 | 18.2 | 2 | 69.1 | 72.2 | 9.6 | 12.9 | 71 | 74 | 82 | 89 | 96 | 109 | 131 |
| G3 | Ken 04-8579 | Heilongjiang,China | 48.3 | 128.1 | 64.6 | 14.5 | 0.2 | 30.9 | 80.3 | 14.8 | 18.4 | 36.2 | 46.2 | 52.2 | 56.2 | 64.2 | 71.2 | 105.2 |
| G4 | Kangxian2 | Heilongjiang,China | 48.3 | 128.1 | 90.7 | 17.5 | 0.8 | 43 | 93.8 | 17.6 | 19 | 35.3 | 46.3 | 53.3 | 62.3 | 72.3 | 83.3 | 85.3 |
| G5 | Liushitianhuancang | Liaoning,China | 40.8 | 122 | 109 | 17.2 | 0.3 | 50.6 | 66.4 | 10.7 | 16.1 | 35 | 42 | 55 | 64 | 72 | 84 | 109 |
| G6 | Tiedou 58 | Liaoning,China | 40.8 | 122 | 96.6 | 19.3 | 1.2 | 59.8 | 108 | 25.5 | 23.9 | 68 | 72 | 82 | 88 | 94 | 107 | 142 |
| G7 | Zhonghuang 20 | Beijing,China | 40.1 | 116.3 | 124 | 19.9 | 3.1 | 82.5 | 144 | 29.9 | 16.9 | 42.5 | 50.5 | 69.5 | 76.5 | 83.5 | 100.5 | 124.5 |
| G8 | Jichanghuangdou 1 | Xinjiang,China | 41.2 | 81.4 | 101 | 20 | 0.9 | 47 | 87.8 | 16.8 | 19.9 | 65 | 70 | 79 | 84 | 99 | 115 | 132 |
| G9 | Longquandadou | Heilongjiang,China | 48.3 | 128.1 | 79.2 | 13.6 | 0.9 | 21.3 | 7.5 | 2.5 | 33 | 39.3 | 47.3 | 53.3 | 58.3 | 64.3 | 74.3 | 103.3 |
| G10 | Xiaobaiqi | Liaoning,China | 40.8 | 122 | 88.6 | 16.8 | 2.6 | 51.8 | 48.1 | 9.3 | 23.9 | 57 | 61 | 68 | 74 | 84 | 98 | 129 |
| G11 | Gongye 04L-141 | Jilin,China | 43.4 | 126.3 | 82 | 16.5 | 0.2 | 25.4 | 50.3 | 8.5 | 18.3 | 37.3 | 44.3 | 62.3 | 72.3 | 80.3 | 92.3 | 111.3 |
| G12 | Tiedou 52 | Liaoning,China | 40.8 | 122 | 76.8 | 15.5 | 0.6 | 34.4 | 75.2 | 18.1 | 24.3 | 34.5 | 43.5 | 53.5 | 61.5 | 76.5 | 91.5 | 128.5 |
| G13 | L-57 | America | 43.4 | 79.3 | 102 | 17.5 | 1 | 46.7 | 72.2 | 14.3 | 19.8 | 37 | 42 | 59 | 64 | 74 | 87 | 111 |
| G14 | He 05-31 | Heilongjiang,China | 48.3 | 128.1 | 77 | 15.9 | 0.4 | 44.6 | 88.8 | 14.4 | 16.3 | 43.8 | 49.8 | 57.8 | 62.8 | 71.8 | 80.8 | 108.8 |
| G15 | L-21 | Canada | 43.4 | 79.3 | 81.1 | 16.4 | 0.9 | 37.5 | 53.8 | 13.6 | 38.7 | 33.2 | 41.2 | 49.2 | 56.2 | 64.2 | 73.2 | 98.2 |
| G16 | Hongfeng11 | Heilongjiang,China | 48.3 | 128.1 | 58.2 | 14.7 | 0.4 | 34.2 | 71.5 | 14.6 | 20.9 | 35.4 | 42.4 | 53.4 | 59.4 | 65.4 | 75.4 | 103.4 |
| G17 | Heinong 33 | Heilongjiang,China | 48.3 | 128.1 | 73.9 | 16.3 | 0.1 | 23.4 | 44 | 10 | 22.7 | 34.9 | 42.9 | 51.9 | 57.9 | 65.9 | 76.9 | 107.9 |
| G18 | Liao 98072 | Liaoning,China | 40.8 | 122 | 104 | 20.2 | 0.8 | 50.6 | 97.8 | 19.4 | 19.9 | 49.3 | 56.3 | 62.3 | 72.3 | 84.3 | 101.3 | 131.3 |
| G19 | Kenfeng 22 | Heilongjiang,China | 48.3 | 128.1 | 80.3 | 16.2 | 0.9 | 36.7 | 75.6 | 16.1 | 22.1 | 34.5 | 41.5 | 49.5 | 59.5 | 68.5 | 88.5 | 103.5 |
| G20 | Jiyu 89 | Jilin,China | 43.4 | 126.3 | 77 | 15.8 | 1 | 38 | 50.9 | 10.9 | 21.4 | 34 | 44 | 57 | 61 | 69 | 79 | 102 |
| G21 | Tiedou 51 | Liaoning,China | 40.8 | 122 | 95.1 | 17.5 | 0.5 | 36 | 57.2 | 16.5 | 40.2 | 40 | 48 | 59 | 69 | 72 | 91 | 127 |
| G22 | Tongnong 13 | Jilin,China | 43.4 | 126.3 | 92.3 | 16.3 | 1.1 | 48.4 | 52.1 | 13.8 | 31.7 | 39.3 | 47.3 | 58.3 | 69.3 | 72.3 | 82.3 | 119.3 |
| G23 | Bei 1873 | Heilongjiang,China | 48.3 | 128.1 | 63.3 | 16.1 | 0.3 | 46.1 | 84.2 | 17.8 | 21.2 | 33.4 | 39.4 | 47.4 | 52.4 | 59.4 | 65.4 | 96.4 |
| G24 | Hefeng 50 | Heilongjiang,China | 48.3 | 128.1 | 72.3 | 16.4 | 0.2 | 41 | 72.5 | 18.8 | 29.3 | 33.4 | 41.4 | 50.4 | 56.4 | 65.4 | 75.4 | 104.4 |
| G25 | Sui 02-339 | Heilongjiang,China | 48.3 | 128.1 | 70.4 | 15.7 | 0.5 | 36.2 | 53 | 13 | 29 | 39 | 46 | 55 | 62 | 72 | 81 | 111 |
| G26 | Hujiao 03-286 | Inner Mongolia,China | 44.5 | 111.7 | 67.5 | 12.9 | 0.2 | 29.5 | 38.4 | 7.9 | 20.8 | 33.3 | 41.3 | 48.3 | 51.3 | 58.3 | 63.3 | 88.3 |
| G27 | Heinong 44 | Heilongjiang,China | 48.3 | 128.1 | 73.4 | 16.1 | 0 | 56.1 | 99.1 | 20.4 | 20.6 | 34.2 | 40.2 | 51.2 | 56.2 | 66.2 | 75.2 | 100.2 |
| G28 | Hujiao 04-528 | Inner Mongolia,China | 44.5 | 111.7 | 64.9 | 13.1 | 0.1 | 30.5 | 41.4 | 7.4 | 19.6 | 34.4 | 40.4 | 47.4 | 52.4 | 57.4 | 63.4 | 88.4 |
| G29 | Fengshou 10 | Heilongjiang,China | 48.3 | 128.1 | 69.3 | 14.5 | 1 | 27.1 | 47.8 | 10.9 | 22.7 | 35.2 | 41.2 | 51.2 | 56.2 | 63.2 | 72.2 | 96.2 |
| G30 | Hersong 2 | Ukraine | 50.3 | 30.3 | 81.1 | 16.3 | 1.2 | 31.3 | 54.7 | 12.2 | 22.5 | 50 | 54 | 61 | 69 | 75 | 86 | 108 |
| G31 | Jinzhou 4-1 | Liaoning,China | 40.8 | 122 | 80.7 | 15.6 | 0.7 | 38.3 | 53 | 12.9 | 24.3 | 61 | 66 | 71 | 76 | 82 | 91 | 130 |
| G32 | Suinong 14 | Heilongjiang,China | 48.3 | 128.1 | 83.6 | 17.6 | 0.3 | 34.5 | 63.2 | 13.5 | 21.7 | 34.4 | 42.4 | 52.4 | 56.4 | 67.4 | 79.4 | 110.4 |
| G33 | Tiejiasilihuang | Jilin,China | 43.4 | 126.3 | 112 | 21.5 | 1.2 | 70.7 | 142 | 26 | 16.3 | 45.3 | 52.3 | 61.3 | 68.3 | 74.3 | 90.3 | 117.3 |
| G34 | Tiefeng 8 | Liaoning,China | 40.8 | 122 | 113 | 20.4 | 2.4 | 61.4 | 67.6 | 16.4 | 23.9 | 78.5 | 82.5 | 90.5 | 96.5 | 106.4 | 116.5 | 140.5 |
| G35 | Silihuang | Jilin,China | 43.4 | 126.3 | 86.3 | 16.9 | 0.7 | 34.4 | 62.4 | 14.1 | 22.4 | 35.3 | 42.3 | 55.3 | 60.3 | 67.3 | 78.3 | 99.3 |
| G36 | Bei 1361 | Heilongjiang,China | 48.3 | 128.1 | 74.4 | 15.4 | 0.1 | 31.9 | 55.3 | 11.8 | 21.4 | 34.4 | 41.4 | 53.4 | 58.4 | 66.4 | 75.4 | 98.4 |
| G37 | L-5 | Canada | 43.4 | 79.3 | 83.9 | 19.8 | 1 | 57.3 | 90.8 | 18.1 | 20.1 | 48 | 56 | 62 | 71 | 80 | 96 | 119 |
| G38 | Maoyandou | Gansu, China | 32.3 | 92.1 | 87.9 | 18 | 1.1 | 37 | 35 | 8.6 | 24.5 | 43 | 51 | 59 | 63 | 71 | 84 | 111 |
| G39 | Fengdihuang | Jilin,China | 43.4 | 126.3 | 94 | 17.4 | 2.2 | 36.5 | 45 | 9.6 | 20 | 56.4 | 60.4 | 70.4 | 79.4 | 89.4 | 100.4 | 130.4 |
| G40 | Tiedou 54 | Liaoning,China | 40.8 | 122 | 95.2 | 20.1 | 0.5 | 65.5 | 96.1 | 19.8 | 37.7 | 61 | 64 | 71 | 78 | 86 | 97 | 129 |
| G41 | Jiunong 20 | Jilin,China | 43.4 | 126.3 | 73.1 | 17.4 | 0.1 | 35.6 | 64.2 | 14.2 | 22.3 | 37.5 | 44.5 | 59.5 | 66.5 | 74.5 | 86.5 | 116.5 |
| G42 | Duludou | Jilin,China | 43.4 | 126.3 | 70.1 | 12.9 | 1.1 | 28.5 | 52.6 | 11.2 | 21.8 | 37.4 | 44.4 | 53.4 | 57.4 | 64.4 | 73.4 | 99.4 |
| G43 | Sui 03-3046 | Heilongjiang,China | 48.3 | 128.1 | 76.8 | 13.7 | 0 | 35.6 | 41.5 | 8.9 | 20.8 | 35 | 42 | 50 | 55 | 63 | 72 | 95 |
| G44 | Hefeng 47 | Heilongjiang,China | 48.3 | 128.1 | 104.5 | 17.5 | 0.2 | 53.4 | 84.3 | 17.9 | 21.2 | 52.4 | 57.4 | 69.4 | 75.4 | 83.4 | 94.4 | 119.4 |
| G45 | V111-4 | Jilin,China | 43.4 | 126.3 | 77.9 | 16.1 | 0 | 41.1 | 85.3 | 16 | 19.6 | 34.9 | 41.9 | 52.9 | 60.9 | 68.9 | 81.9 | 107.9 |
| G46 | Longxuan 1 | Heilongjiang,China | 48.3 | 128.1 | 69.9 | 14.5 | 0 | 24.2 | 46.2 | 10.9 | 23.9 | 35 | 42 | 53 | 56 | 62 | 72 | 105 |
| G47 | Dongnong 8004 | Heilongjiang,China | 48.3 | 128.1 | 71.8 | 15.1 | 0.1 | 33.8 | 62.5 | 15.2 | 24.7 | 35.3 | 43.3 | 51.3 | 56.3 | 66.3 | 78.3 | 102.3 |
| G48 | Sui 03-3952 | Heilongjiang,China | 48.3 | 128.1 | 63.1 | 15.6 | 0.2 | 51.9 | 79.6 | 28.8 | 41.6 | 32.3 | 42.3 | 53.3 | 62.3 | 69.3 | 81.3 | 110.3 |
| G49 | Hefeng 25 | Heilongjiang,China | 48.3 | 128.1 | 79 | 16.6 | 1.2 | 35.1 | 39.4 | 9.2 | 23.1 | 42.3 | 48.3 | 60.3 | 66.3 | 72.3 | 82.3 | 111.3 |
| G50 | Liaodou 3 | Liaoning,China | 40.8 | 122 | 118 | 18.3 | 0.1 | 52.6 | 87.3 | 18.8 | 21.7 | 45.3 | 50.3 | 59.3 | 69.3 | 79.3 | 92.3 | 126.3 |
| G51 | Hujiao 423 | Inner Mongolia,China | 44.5 | 111.7 | 73.2 | 13.7 | 0.5 | 23.3 | 45.9 | 10.3 | 22.4 | 36.2 | 43.2 | 48.2 | 52.2 | 57.2 | 68.2 | 94.2 |
| G52 | Liaoxian 1 | Liaoning,China | 40.8 | 122 | 33.5 | 9.4 | 1.4 | 16.1 | 15.2 | 5.1 | 33.2 | 44 | 49 | 58 | 62 | 67 | 81 | 113 |
| G53 | Zhongpin 95-5388 | Beijing,China | 40.1 | 116.3 | 109 | 21.9 | 0.8 | 46.8 | 68.9 | 12.6 | 19.3 | 57.5 | 59.5 | 70.5 | 75.5 | 88.5 | 109.5 | 142 |
| G54 | Heihe 45 | Heilongjiang,China | 48.3 | 128.1 | 82.2 | 17.9 | 2.6 | 67.4 | 139 | 28.8 | 21.1 | 34.3 | 39.3 | 43.3 | 51.3 | 59.3 | 66.3 | 94.3 |
| G55 | L-59 Peking | America | 41.8 | 92.9 | 117 | 19.7 | 3.2 | 68.8 | 85 | 9.9 | 11.6 | 90.1 | 93.1 | 97.4 | 106.4 | 116.4 | 131.1 | 143 |
| G56 | Sui 04-5804 | Heilongjiang,China | 48.3 | 128.1 | 73.2 | 15.5 | 0 | 28.5 | 50.7 | 11.5 | 23 | 34.4 | 42.4 | 51.4 | 57.4 | 64.4 | 73.4 | 106.4 |
| G57 | Ji 06B7 | Hebei,China | 39.2 | 116.3 | 118 | 22.4 | 0.8 | 37.8 | 55.4 | 10.8 | 19.7 | 74 | 80 | 94 | 106.4 | 116.4 | 130 | 143 |
| G58 | Marca. Joe lisa | Heilongjiang,China | 48.3 | 128.1 | 114.2 | 20.2 | 0.4 | 68.4 | 132 | 14.1 | 10.3 | 61 | 63 | 71 | 79 | 91 | 99 | 128 |
| G59 | Jihuang 13 | Hebei,China | 39.2 | 116.3 | 90.1 | 18.5 | 0.2 | 31.5 | 66.1 | 14 | 21.3 | 52 | 59 | 66 | 74 | 83 | 95 | 130 |
| G60 | L-28 | Canada | 43.4 | 79.3 | 64.1 | 13.4 | 1.6 | 43.7 | 44.9 | 9.6 | 21.7 | 34.4 | 40.4 | 48.4 | 53.4 | 59.4 | 69.4 | 98.4 |
| G61 | Beifeng 9 | Heilongjiang,China | 48.3 | 128.1 | 66.5 | 13.3 | 0 | 30 | 55.5 | 11.7 | 21 | 35.4 | 42.4 | 52.4 | 55.4 | 63.4 | 71.4 | 95.4 |
| G62 | Zhongzuo 00-683 | Beijing,China | 40.1 | 116.3 | 107.5 | 18.6 | 1.7 | 52.2 | 103 | 20.2 | 20 | 53.3 | 57.3 | 68.3 | 73.3 | 75.3 | 96.3 | 128.3 |
| G63 | Dongnong 50 | Heilongjiang,China | 48.3 | 128.1 | 78.5 | 16.3 | 1.5 | 63.5 | 126 | 12.1 | 9.7 | 34.3 | 42.3 | 54.3 | 60.3 | 66.3 | 78.3 | 99.3 |
| G64 | Dongnong 93-046 | Heilongjiang,China | 48.3 | 128.1 | 76.7 | 15.2 | 0.3 | 23.3 | 38.6 | 9.5 | 24.4 | 36.3 | 48.3 | 57.3 | 64.3 | 72.3 | 85.3 | 110.3 |
| G65 | Suinong 10 | Heilongjiang,China | 48.3 | 128.1 | 74.7 | 16 | 0.1 | 37.6 | 65.4 | 14.6 | 22.5 | 34.2 | 44.2 | 53.2 | 57.2 | 64.2 | 73.2 | 100.2 |
| G66 | Tiefeng 31 | Liaoning,China | 40.8 | 122 | 99 | 18 | 1.3 | 43.2 | 75.2 | 15.4 | 20.3 | 45 | 49 | 62 | 74 | 89 | 100 | 130 |
| G67 | Dongnong 49 | Heilongjiang,China | 48.3 | 128.1 | 63.9 | 13.3 | 0 | 24.3 | 42.1 | 8.8 | 20.9 | 33.1 | 43.1 | 51.1 | 54.1 | 59.1 | 65.1 | 87.1 |
| G68 | Huayou 446 | Hebei,China | 39.2 | 116.3 | 110 | 22.5 | 0.8 | 35.7 | 69 | 16.4 | 23.2 | 57.3 | 61.3 | 69.3 | 76.3 | 87.3 | 112.3 | 143 |
| G69 | Heihe 48 | Heilongjiang,China | 48.3 | 128.1 | 69.2 | 14.3 | 0.1 | 35.8 | 52.3 | 10.2 | 19.3 | 34.4 | 41.4 | 48.4 | 52.4 | 60.4 | 69.4 | 87.4 |
| G70 | Huajiang 2 | Heilongjiang,China | 48.3 | 128.1 | 66 | 14.2 | 0 | 37.2 | 44.4 | 9.2 | 20.3 | 33.3 | 38.3 | 44.3 | 48.3 | 57.3 | 63.3 | 81.3 |
| G71 | Suinong 29 | Heilongjiang,China | 48.3 | 128.1 | 70.7 | 14.8 | 0 | 33.1 | 97.4 | 12.6 | 22.3 | 36.3 | 44.3 | 51.3 | 57.3 | 63.3 | 70.3 | 98.3 |
| G72 | Hefeng 35 | Heilongjiang,China | 48.3 | 128.1 | 65.5 | 15.1 | 0.2 | 29.5 | 53.3 | 12.2 | 23.6 | 35.3 | 43.3 | 53.3 | 57.3 | 64.3 | 71.3 | 103.3 |
| G73 | Suinong 8 | Heilongjiang,China | 48.3 | 128.1 | 78.8 | 16.2 | 0.6 | 44 | 85.1 | 20.7 | 24.7 | 37 | 43 | 53 | 57 | 64 | 74 | 103 |
| G74 | Jilin 47 | Jilin,China | 43.4 | 126.3 | 77.3 | 17 | 0 | 53.6 | 85.9 | 22 | 32.3 | 36.2 | 46.2 | 60.2 | 66.2 | 73.2 | 82.2 | 112.2 |
| G75 | Heinong 48 | Heilongjiang,China | 48.3 | 128.1 | 83.7 | 16 | 0.2 | 31.1 | 54.6 | 16.4 | 30.6 | 36 | 42 | 52 | 56 | 66 | 73 | 110 |
| G76 | Nova | Italy | 41.5 | 12.3 | 111 | 20.3 | 0.4 | 53.3 | 87.7 | 22.7 | 26.4 | 46 | 53 | 66 | 72 | 79 | 94 | 125 |
| G77 | Beidou 14 | Heilongjiang,China | 48.3 | 128.1 | 78.1 | 15.4 | 0.3 | 33.3 | 64.1 | 15.6 | 22.5 | 34 | 42 | 50 | 53 | 60 | 69 | 94 |
| G78 | Kangxian 3 | Heilongjiang,China | 48.3 | 128.1 | 103 | 18.3 | 0.4 | 37.7 | 65.1 | 12.8 | 20.6 | 34.4 | 44.4 | 57.4 | 67.4 | 73.4 | 89.4 | 111.4 |
| G79 | Dongnong 42 | Heilongjiang,China | 48.3 | 128.1 | 81.2 | 14.8 | 0 | 21.9 | 41.1 | 11.7 | 28.7 | 33.2 | 40.2 | 51.2 | 55.2 | 64.2 | 77.2 | 104.2 |
| G80 | Kenjian 23 | Heilongjiang,China | 48.3 | 128.1 | 88.3 | 19.4 | 0 | 41.6 | 61.3 | 9.9 | 18.1 | 32.5 | 41.5 | 47.5 | 53.5 | 61.5 | 82.5 | 87.5 |
| G81 | Heinong 55 | Heilongjiang,China | 48.3 | 128.1 | 67 | 14.7 | 0 | 36.1 | 83.9 | 19.5 | 23.4 | 34.2 | 42.2 | 50.2 | 57.2 | 66.2 | 76.2 | 110.2 |
| G82 | Hefeng 45 | Heilongjiang,China | 48.3 | 128.1 | 68.3 | 16.4 | 1 | 37.3 | 76.2 | 17.8 | 23.5 | 36.3 | 46.3 | 53.3 | 57.3 | 66.3 | 79.3 | 104.3 |
| G83 | Kenfeng 15 | Heilongjiang,China | 48.3 | 128.1 | 47.7 | 12.4 | 0 | 28.1 | 59.1 | 9.2 | 15.8 | 33.2 | 41.2 | 51.2 | 55.2 | 63.2 | 74.2 | 104.2 |
| G84 | Chasedou | Jilin,China | 43.4 | 126.3 | 91.8 | 17.8 | 1.2 | 44.8 | 67 | 13.4 | 20.2 | 35.3 | 49.3 | 59.3 | 71.3 | 80.3 | 91.3 | 109.3 |
| G85 | He 05-991 | Heilongjiang,China | 48.3 | 128.1 | 84.4 | 15.7 | 0.1 | 27.3 | 43.9 | 10.1 | 22.8 | 35.2 | 41.2 | 48.2 | 52.2 | 62.2 | 69.2 | 89.2 |
| G86 | Xiaolimoshidou | Heilongjiang,China | 39.2 | 116.3 | 63.3 | 13.2 | 2 | 54.4 | 69.3 | 9.6 | 13 | 38.3 | 48.3 | 59.3 | 64.3 | 69.3 | 82.3 | 105.3 |
| G87 | Jingshanpu | Heilongjiang,China | 48.3 | 128.1 | 79.4 | 15.9 | 0.9 | 35 | 57.1 | 11.9 | 21.9 | 38.3 | 46.3 | 60.3 | 66.3 | 74.3 | 84.3 | 111.3 |
| G88 | Qinganheidou | Heilongjiang,China | 48.3 | 128.1 | 106 | 19.4 | 2.2 | 54.5 | 80.2 | 17.3 | 21.9 | 47.2 | 51 | 58 | 62 | 69 | 80 | 109 |
| G89 | Dongnong594 | Heilongjiang,China | 48.3 | 128.1 | 72.1 | 15.9 | 0.1 | 25.2 | 53.1 | 12.6 | 23.8 | 36.2 | 43.2 | 52.2 | 58.2 | 66.2 | 81.2 | 109.2 |
| G90 | Yapoche | Heilongjiang,China | 48.3 | 128.1 | 78.6 | 17.6 | 1 | 51.8 | 93.5 | 17.3 | 18.6 | 39.4 | 46.5 | 58.4 | 68.4 | 73.4 | 86.4 | 110.4 |
| G91 | Kennong 29 | Heilongjiang,China | 48.3 | 128.1 | 60.6 | 13.8 | 0.1 | 34.8 | 53.8 | 12.8 | 24.1 | 34.2 | 43.2 | 52.2 | 58.2 | 64.2 | 73.2 | 101.2 |
| G92 | Heimoshidou | Jilin,China | 43.4 | 126.3 | 129 | 18.3 | 2.6 | 58.6 | 76.2 | 10.8 | 14.4 | 60.1 | 63.1 | 70.1 | 82.1 | 88.1 | 105.1 | 126.1 |
| G93 | Ha 04-1824 | Heilongjiang,China | 48.3 | 128.1 | 76.1 | 14.1 | 0.1 | 26 | 49.3 | 10.6 | 21.6 | 36.3 | 42.3 | 53.3 | 56.3 | 64.3 | 74.3 | 109.3 |
| G94 | Fangzhengmoshidou | Heilongjiang,China | 48.3 | 128.1 | 92.2 | 17 | 1.3 | 36.2 | 49.8 | 9.9 | 18.7 | 48.4 | 56.4 | 64.4 | 71.4 | 78.4 | 88.4 | 111.4 |
| G95 | Helongyoutai | Jilin,China | 43.4 | 126.3 | 82.9 | 15.4 | 1.5 | 45.5 | 61.6 | 12.9 | 20.6 | 39 | 45 | 54 | 59 | 64 | 72 | 96 |
| G96 | Kexin 3 | Beijing,China | 40.1 | 116.3 | 109 | 21 | 0.5 | 43.4 | 71.7 | 17.1 | 24 | 72 | 74 | 84 | 92 | 96 | 112 | 133 |
| G97 | Yuanbaojin | Heilongjiang,China | 48.3 | 128.1 | 80.1 | 13.4 | 1.4 | 27.6 | 50.7 | 10.5 | 20.9 | 42.4 | 49.4 | 56.4 | 60.4 | 66.4 | 73.4 | 99.4 |
| G98 | Zhongzuo J4133 | Beijing,China | 40.1 | 116.3 | 118 | 19.2 | 0.5 | 43.2 | 72.6 | 16.1 | 22.7 | 59.4 | 62.4 | 73.4 | 89.4 | 96.4 | 114.4 | 140.4 |
| G99 | Jilin 30 | Jilin,China | 43.4 | 126.3 | 104.3 | 15.6 | 1.7 | 36.6 | 81.1 | 15.7 | 20 | 37.3 | 48.3 | 61.3 | 71.3 | 77.3 | 91.3 | 121 |
| G100 | Ji 100 | Jilin,China | 43.4 | 126.3 | 86.2 | 15.2 | 0.6 | 32.6 | 49.3 | 12.1 | 24.9 | 39.2 | 46.2 | 59.2 | 62.2 | 73.2 | 82.2 | 110.2 |
| G101 | Suinong 30 | Heilongjiang,China | 48.3 | 128.1 | 74.3 | 16.9 | 0.1 | 37.4 | 75.1 | 14.3 | 19.4 | 36.3 | 41.3 | 49.3 | 56.3 | 62.3 | 70.3 | 98.3 |
| G102 | Boige du | Germany | 52.3 | 13.2 | 77.8 | 12.6 | 1.8 | 24 | 33.7 | 6.2 | 18.5 | 44 | 50 | 57 | 61 | 66 | 77 | 103 |
| G103 | Beidou 16 | Heilongjiang,China | 48.3 | 128.1 | 94.9 | 19.7 | 0.8 | 42.4 | 66.1 | 14.2 | 21.3 | 44 | 50 | 58 | 69 | 76 | 90 | 111 |
| G104 | Kebei 1 | Heilongjiang,China | 48.3 | 128.1 | 83.1 | 15.9 | 0.5 | 35.7 | 56.2 | 13.1 | 23.5 | 34 | 42 | 51 | 57 | 64 | 72 | 96 |
| G105 | Mengdou 21 | Inner Mongolia,China | 44.5 | 111.7 | 59.2 | 14 | 0 | 34.4 | 70 | 14.1 | 20.1 | 34.5 | 44.5 | 48.5 | 57.5 | 62.5 | 74.5 | 93.5 |
| G106 | Heihe 38 | Heilongjiang,China | 48.3 | 128.1 | 64.8 | 14.4 | 0.1 | 25.2 | 29.1 | 6.5 | 22 | 33.4 | 40.4 | 49.4 | 54.4 | 60.4 | 69.4 | 94.4 |
| G107 | Zhongdou 35 | Beijing,China | 40.1 | 116.3 | 83.2 | 18.2 | 1.1 | 35 | 70.2 | 14.6 | 21.1 | 41 | 48 | 56 | 66 | 74 | 92 | 129 |
| G108 | Zhonghuang 6 | Beijing,China | 40.1 | 116.3 | 112 | 21.2 | 0.5 | 52.3 | 85.5 | 25.7 | 37.1 | 60.5 | 67.5 | 77.5 | 82.5 | 94.5 | 102.5 | 128.5 |
| G109 | Jiyu 94 | Jilin,China | 43.4 | 126.3 | 101 | 17.3 | 1.1 | 53.8 | 74.9 | 15.2 | 20.4 | 64 | 67 | 74 | 78 | 87 | 101 | 130 |
| G110 | Dongnong 44 | Heilongjiang,China | 48.3 | 128.1 | 40 | 11.4 | 0.6 | 36.4 | 32.8 | 9 | 32.2 | 32.8 | 37.8 | 41.8 | 47.8 | 55.8 | 60.8 | 80.8 |
| G111 | Liaonong 2 | Liaoning,China | 40.8 | 122 | 74.8 | 15 | 0.2 | 41.1 | 75 | 12.1 | 16.3 | 58 | 60 | 69 | 74 | 78 | 89 | 121 |
| G112 | Bei 1484 | America | 48.3 | 128.1 | 70.8 | 15.7 | 0.2 | 41 | 76.4 | 23.7 | 32.8 | 31.4 | 38.4 | 44.4 | 49.4 | 58.4 | 64.4 | 92.4 |
| G113 | Williams82 | America | 41.8 | 92.9 | 91.5 | 18.7 | 2.5 | 67.6 | 113 | 19.6 | 17.3 | 49 | 52 | 60 | 72 | 80 | 91 | 119 |
| G114 | Jindou 33 | Liaoning,China | 40.8 | 122 | 110 | 17.8 | 0.8 | 53 | 77.6 | 19.5 | 25.2 | 72.2 | 74.2 | 83.2 | 88.2 | 96.2 | 113.2 | 140.2 |
| G115 | Kenfeng 18 | Heilongjiang,China | 48.3 | 128.1 | 80.7 | 18.6 | 0.4 | 46.9 | 80.3 | 17.2 | 21.4 | 35.3 | 43.3 | 54.3 | 62.3 | 73.3 | 83.3 | 111.3 |
| G116 | Suinong 20 | Heilongjiang,China | 48.3 | 128.1 | 71 | 17.4 | 0.9 | 61.4 | 95.7 | 20.5 | 21.6 | 34.3 | 41.3 | 52.3 | 57.3 | 66.3 | 76.3 | 107.3 |
| G117 | Mengdou 9 | Inner Mongolia,China | 44.5 | 111.7 | 67.2 | 12.8 | 0 | 22.2 | 21.2 | 6.2 | 31.8 | 34 | 42 | 49 | 54 | 59 | 65 | 91 |
| G118 | L-9 | America | 41.8 | 92.9 | 122 | 26.4 | 0.3 | 40.3 | 56.8 | 9.2 | 16.7 | 73 | 78 | 94 | 106.4 | 116.4 | 127 | 143 |
| G119 | Suinong 1 | Heilongjiang,China | 48.3 | 128.1 | 102 | 19.4 | 1.4 | 67.4 | 129 | 24.7 | 19.9 | 48.2 | 53.2 | 61.2 | 70.2 | 77.2 | 94.2 | 129 |
| G120 | Kenfeng 14 | Heilongjiang,China | 48.3 | 128.1 | 70.2 | 16.2 | 0.3 | 38.8 | 77.3 | 17.3 | 23.2 | 34.4 | 44.4 | 54.4 | 58.3 | 63.4 | 76.4 | 108.4 |
| G121 | Tiejiazi | Liaoning,China | 40.8 | 122 | 103.7 | 18.1 | 0.2 | 37.4 | 65 | 15.9 | 25.1 | 41 | 48 | 67 | 75 | 80 | 93 | 126 |
| G122 | Suinong 4 | Heilongjiang,China | 48.3 | 128.1 | 67.2 | 17.4 | 1.2 | 51.3 | 87.4 | 21 | 24.3 | 37 | 43 | 52 | 57 | 66 | 78 | 109 |
| G123 | Jinshanchamoshidou | Jilin,China | 43.4 | 126.3 | 120 | 18.3 | 1.4 | 52.8 | 58.3 | 7.3 | 12.6 | 71.4 | 74.4 | 84.4 | 96.4 | 106.4 | 117.4 | 132.4 |
| G124 | Longpin 03-311 | Heilongjiang,China | 48.3 | 128.1 | 71.2 | 15.7 | 0 | 42.5 | 60.2 | 15.5 | 25.8 | 32.3 | 40.3 | 49.3 | 55.3 | 64.3 | 80.3 | 99.3 |
| G125 | Jilinchalihua | Jilin,China | 43.4 | 126.3 | 93.8 | 14.4 | 2.5 | 28 | 30.5 | 2.7 | 9.1 | 65.3 | 71.3 | 84.3 | 92.3 | 96.3 | 113.3 | 130.3 |
| G126 | Heinong 37 | Heilongjiang,China | 48.3 | 128.1 | 59.6 | 18.2 | 0.4 | 73.3 | 138 | 27 | 21 | 35.4 | 42.4 | 49.4 | 65.4 | 70.4 | 81.4 | 108 |
| G127 | Huajiang 4403 | Heilongjiang,China | 48.3 | 128.1 | 78.7 | 16 | 0.3 | 30.8 | 51.7 | 13.4 | 26.2 | 35.3 | 42.3 | 49.3 | 54.3 | 60.3 | 69.3 | 94.3 |
| G128 | Huangbaozhu | Liaoning,China | 40.8 | 122 | 124 | 19.5 | 1.6 | 51.2 | 84.3 | 17 | 20 | 49.5 | 56.5 | 62.5 | 74.5 | 80.5 | 92.5 | 125.5 |
| G129 | Suinong 25 | Heilongjiang,China | 48.3 | 128.1 | 82 | 17.3 | 0 | 34.9 | 56.9 | 15.3 | 26.9 | 35 | 43 | 54 | 62 | 72 | 85 | 108 |
| G130 | Sui 05-7304 | Heilongjiang,China | 48.3 | 128.1 | 73 | 14.2 | 0 | 27.4 | 31.6 | 7.6 | 24 | 35 | 41 | 48 | 52 | 59 | 66 | 94 |
| G131 | Jidou 9 | Hebei,China | 39.2 | 116.3 | 112 | 22 | 0.5 | 42.7 | 83.4 | 21.3 | 24.8 | 48 | 56 | 61 | 71 | 85 | 110 | 142 |
| G132 | Mengdou 14 | Inner Mongolia,China | 44.5 | 111.7 | 65.3 | 14 | 0.2 | 18.2 | 32.5 | 6.6 | 20.1 | 32.3 | 41.3 | 50.3 | 54.3 | 59.3 | 67.3 | 93.3 |
| G133 | Handou 5 | Hebei,China | 39.2 | 116.3 | 92.3 | 16.6 | 0.5 | 30.6 | 55.5 | 14.3 | 26 | 49 | 56 | 62 | 74 | 80 | 99 | 129 |
| G134 | Jidou 17 | Hebei,China | 39.2 | 116.3 | 132 | 20.3 | 0.9 | 45.6 | 77.7 | 19.8 | 25.9 | 56 | 60 | 69 | 79 | 91 | 110 | 139 |
| G135 | Aika 166 | Heilongjiang,China | 44.2 | 26.1 | 57.6 | 14.4 | 2.1 | 52 | 95.2 | 18.1 | 19.3 | 35.3 | 43.3 | 58.3 | 62.3 | 69.3 | 78.3 | 110.3 |
| G136 | Sui 04-6018 | Heilongjiang,China | 48.3 | 128.1 | 65.1 | 16.6 | 0 | 32 | 35 | 17.1 | 39.5 | 34.2 | 44.2 | 52.2 | 58.2 | 65.2 | 73.2 | 96.2 |
| G137 | Dongnong 48 | Heilongjiang,China | 48.3 | 128.1 | 68.1 | 14.3 | 0 | 19.7 | 32.1 | 7.7 | 24.1 | 36.4 | 42.4 | 51.4 | 58.4 | 63.4 | 72.4 | 104.4 |
| G138 | L-79 | America | 43.4 | 79.3 | 58.4 | 11.8 | 0.5 | 21.2 | 21.4 | 4.1 | 17.2 | 35 | 41 | 47 | 52 | 57 | 63 | 88 |
| G139 | Zhonghuang 35 | Beijing,China | 40.1 | 116.3 | 64.3 | 13.5 | 1.1 | 20.8 | 29.9 | 6.7 | 22.2 | 37.4 | 44.4 | 50.4 | 57.4 | 64.4 | 70.4 | 97.4 |
| G140 | Kennong 30 | Heilongjiang,China | 48.3 | 128.1 | 64.2 | 14.8 | 0.1 | 29.4 | 61.8 | 14.1 | 23.9 | 35 | 42 | 50 | 57 | 64 | 74 | 106 |
| G141 | Zhonghuang 30 | Beijing,China | 40.1 | 116.3 | 87.6 | 18.7 | 0.5 | 40.2 | 72.1 | 17.2 | 24.1 | 51.4 | 56.4 | 67.4 | 74.4 | 81.4 | 90.4 | 126.4 |
| G142 | Baichengmoshidou | Jilin,China | 43.4 | 126.3 | 111 | 17.5 | 1.9 | 71.7 | 128 | 17 | 13.3 | 57 | 62 | 70 | 76 | 80 | 95 | 111 |
| G143 | L-10 | America | 43.4 | 79.3 | 104 | 18.9 | 2 | 58.2 | 74.3 | 20.2 | 30.1 | 47.4 | 54.4 | 63.4 | 70.4 | 79.4 | 96.4 | 126 |
| G144 | Zaoshu 18 | Beijing,China | 40.1 | 116.3 | 60.8 | 15.2 | 2 | 42.3 | 66.9 | 18.3 | 27.2 | 50.3 | 56.3 | 61.3 | 70.3 | 78.3 | 90.3 | 125.3 |
| G145 | Zhonghuang 10 | Beijing,China | 40.1 | 116.3 | 97.1 | 17.5 | 1 | 56 | 77.7 | 15.6 | 20.4 | 62 | 67 | 79 | 84 | 92 | 111 | 134 |
| G146 | Dongnong L-13 | Heilongjiang,China | 43.4 | 79.3 | 97.2 | 15.4 | 0.1 | 37.6 | 76.9 | 16.7 | 22.8 | 34.4 | 42.4 | 53.4 | 57.4 | 65.4 | 78.4 | 105.4 |
| G147 | Neifeng 15 | Heilongjiang,China | 48.3 | 128.1 | 66.3 | 13.6 | 1 | 28.9 | 44.6 | 10.1 | 22.5 | 39.2 | 44.2 | 49.2 | 53.2 | 62.2 | 71.2 | 99.2 |
| G148 | Dongnong 47 | Heilongjiang,China | 48.3 | 128.1 | 63.2 | 14.7 | 0 | 29.4 | 70.6 | 14 | 21.5 | 36.2 | 41.2 | 48.2 | 53.2 | 66.2 | 77.2 | 104.2 |
| G149 | Hefeng 29 | Heilongjiang,China | 48.3 | 128.1 | 81.8 | 17.1 | 0.1 | 32.1 | 52.9 | 11 | 20.9 | 42 | 49 | 62 | 69 | 80 | 91 | 115 |
| G150 | Dongnong 07-909 | Heilongjiang,China | 48.3 | 128.1 | 87.2 | 17.8 | 0 | 29.2 | 51.4 | 10.8 | 21.1 | 35 | 41 | 47 | 52 | 60 | 69 | 96 |
| G151 | Hefeng 52 | Heilongjiang,China | 48.3 | 128.1 | 64.8 | 14.5 | 0.3 | 48.9 | 67.3 | 14.9 | 25.4 | 36.3 | 42.3 | 53.3 | 57.3 | 66.3 | 77.3 | 104.3 |
| G152 | Heihe 18 | Heilongjiang,China | 48.3 | 128.1 | 52.4 | 11.7 | 0.2 | 26.5 | 12.7 | 2.5 | 18 | 34.6 | 40.6 | 46.6 | 50.6 | 57.6 | 62.6 | 81.6 |
| G153 | Neifeng 11 | Heilongjiang,China | 48.3 | 128.1 | 73.6 | 15.4 | 0.1 | 27.6 | 42.9 | 10.6 | 26.2 | 35.4 | 42.4 | 53.4 | 56.4 | 63.4 | 71.4 | 102.4 |
| G154 | Heihexiaohuangdou | Heilongjiang,China | 48.3 | 128.1 | 64.4 | 15 | 0.8 | 42.2 | 58.8 | 14 | 23.5 | 33.4 | 38.4 | 47.4 | 55.4 | 61.4 | 70.4 | 84.4 |
| G155 | Zhongdou 27 | Beijing,China | 40.1 | 116.3 | 88.3 | 16.9 | 1.5 | 49.7 | 66.5 | 15.9 | 24.3 | 54.3 | 59.3 | 68.3 | 75.3 | 82.3 | 96.3 | 124.3 |
| G156 | Bei 4834 | Heilongjiang,China | 48.3 | 128.1 | 89.3 | 16 | 2 | 42.3 | 41.9 | 7.9 | 23.1 | 40.4 | 48.4 | 56.4 | 61.4 | 66.4 | 74.4 | 105.4 |
| G157 | Heilongjiang 41 | Heilongjiang,China | 48.3 | 128.1 | 116 | 18.9 | 0.1 | 37.9 | 53.1 | 11.7 | 24.3 | 45 | 52 | 62 | 71 | 77 | 91 | 121 |
| G158 | Zhongpin 03-5179 | Beijing,China | 40.1 | 116.3 | 81.7 | 16.9 | 0.8 | 30.4 | 14.4 | 0.2 | 21.4 | 42.2 | 47.2 | 55.2 | 61.2 | 68.2 | 89.2 | 130.2 |
| G159 | Hefeng 37 | Heilongjiang,China | 48.3 | 128.1 | 128 | 16.4 | 0.7 | 42.2 | 70.6 | 13.5 | 19.2 | 73 | 83 | 92 | 106.4 | 116.4 | 128 | 143 |
| G160 | Dongnong 46 | Heilongjiang,China | 48.3 | 128.1 | 61 | 14.2 | 0.5 | 23.8 | 46.8 | 11.3 | 24.4 | 37 | 45 | 51 | 57 | 64 | 76 | 103 |
| G161 | Dongnong 43 | Heilongjiang,China | 48.3 | 128.1 | 66.7 | 14.8 | 2.1 | 44.5 | 76.1 | 18 | 24.9 | 42.3 | 47.3 | 55.3 | 62.3 | 67.3 | 80.3 | 107.3 |
| G162 | Dongnong 1068 | Heilongjiang,China | 48.3 | 128.1 | 68.6 | 15.1 | 0.9 | 41.2 | 69.8 | 16.7 | 24.2 | 35.4 | 43.4 | 52.4 | 56.4 | 64.4 | 72.4 | 106.4 |
| G163 | Dongnong 56 | Heilongjiang,China | 48.3 | 128.1 | 96.4 | 17.8 | 1.1 | 56.2 | 71.5 | 15.4 | 21.5 | 40 | 44 | 55 | 61 | 69 | 79 | 110 |
| G164 | Jiunong 21 | Jilin,China | 43.4 | 126.3 | 91.3 | 19.3 | 0.8 | 57.8 | 127 | 21.7 | 17.5 | 35.5 | 43.5 | 62.5 | 67.5 | 79.5 | 91.5 | 124.5 |
| G165 | Chamoshidou | Jilin,China | 43.4 | 126.3 | 94.1 | 12.4 | 2.7 | 44.5 | 34.2 | 2.5 | 7.3 | 60 | 62 | 80 | 96.4 | 116.4 | 128 | 143 |
| G166 | Daliheidou | Shandong,China | 36.2 | 118.3 | 86.7 | 16 | 1.7 | 32.1 | 41.7 | 9.4 | 22.4 | 35.3 | 44.3 | 49.3 | 57.3 | 73.3 | 85.3 | 122.3 |
| G167 | Zhongpin 03-5373 | Beijing,China | 40.1 | 116.3 | 85.6 | 16.4 | 2.3 | 46.4 | 69.5 | 13.6 | 20 | 72.4 | 75.4 | 82.4 | 86.4 | 96.4 | 125.4 | 142 |
| G168 | Wuxing 4 | Hebei,China | 39.2 | 116.3 | 107 | 22.5 | 1.7 | 50 | 90.7 | 20.5 | 22.7 | 76 | 81 | 86 | 99 | 106.4 | 117 | 140 |
| G169 | Hefeng 55 | Heilongjiang,China | 48.3 | 128.1 | 72.3 | 16.3 | 0.6 | 37.7 | 60 | 12.8 | 21.6 | 33 | 41 | 51 | 55 | 64 | 76 | 106 |
| G170 | Suinong 28 | Heilongjiang,China | 48.3 | 128.1 | 70.4 | 16 | 0 | 32.9 | 67.8 | 13.6 | 20.3 | 35.3 | 46.3 | 54.3 | 60.3 | 66.3 | 77.3 | 106.3 |
| G171 | Zhongpin 03-5334 | Beijing,China | 40.1 | 116.3 | 80.8 | 17.1 | 3.3 | 78.4 | 129 | 24.5 | 19.1 | 65 | 69 | 80 | 87 | 94 | 110 | 139 |
| G172 | Fengshou 6 | Heilongjiang,China | 48.3 | 128.1 | 68.3 | 15.7 | 0.7 | 31.7 | 63.9 | 13.8 | 22 | 33.3 | 41.3 | 50.3 | 58.3 | 67.3 | 77.3 | 100.3 |
| G173 | Dunajika | Russia | 61.5 | 98 | 81.4 | 14.7 | 2.8 | 55.6 | 86.9 | 11.1 | 12.9 | 38.3 | 46.3 | 55.3 | 60.3 | 66.3 | 77.3 | 101.3 |

| **Number** | **Accession** | **Origin** | **Latitude (°N)** | **Longitude (°W)** | **2015 Shenyang** | | | | | | | | | | | | | |
| --- | --- | --- | --- | --- | --- | --- | --- | --- | --- | --- | --- | --- | --- | --- | --- | --- | --- | --- |
|  |  |  |  |  | **PH**  **(cm)** | **NMN** | **EBN** | **EPN** | **GN** | **GW**  **(g)** | **HGW**  **(g)** | **R1**  **(day)** | **R2**  **(day)** | **R3**  **(day)** | **R4**  **(day)** | **R5**  **(day)** | **R6**  **(day)** | **R7**  **(day)** |
| G1 | Tiedou 50 | Liaoning,China | 40.8 | 122 | 97.1 | 18.1 | 3.7 | 90.6 | 217.7 | 46 | 21.3 | 42.8 | 48.3 | 62.4 | 66.4 | 73.4 | 79.4 | 105.4 |
| G2 | Datun xiaoheidou | Hebei,China | 39.2 | 116.3 | 126 | 19.1 | 3.4 | 66.4 | 102.3 | 13.3 | 12.9 | 58.7 | 64.0 | 74.0 | 79.0 | 84.0 | 89.0 | 118.2 |
| G3 | Ken 04-8579 | Heilongjiang,China | 48.3 | 128.1 | 58.1 | 14.7 | 1 | 41.3 | 104.2 | 17.2 | 16.5 | 34.6 | 41.2 | 56.7 | 60.6 | 68.6 | 100.3 | 104.2 |
| G4 | Kangxian2 | Heilongjiang,China | 48.3 | 128.1 | 108 | 19.9 | 1.1 | 58.5 | 111.2 | 20.9 | 19 | 35.4 | 40.3 | 58.3 | 64.1 | 73.5 | 78.5 | 84.5 |
| G5 | Liushitianhuancang | Liaoning,China | 40.8 | 122 | 109 | 18.2 | 2.4 | 85.4 | 191.8 | 28.4 | 14.8 | 40.2 | 44.5 | 63.7 | 71.3 | 76.3 | 81.3 | 101.3 |
| G6 | Tiedou 58 | Liaoning,China | 40.8 | 122 | 64.8 | 15 | 4.2 | 107.3 | 213.2 | 50.8 | 23.8 | 54.0 | 58.7 | 71.0 | 77.0 | 81.0 | 87.0 | 114.0 |
| G7 | Zhonghuang 20 | Beijing,China | 40.1 | 116.3 | 114 | 18.1 | 2.6 | 53.6 | 95.9 | 17.3 | 18 | 43.6 | 49.1 | 69.3 | 73.8 | 80.8 | 88.8 | 114.8 |
| G8 | Jichanghuangdou 1 | Xinjiang,China | 41.2 | 81.4 | 101 | 17.8 | 2 | 62.3 | 115 | 20 | 17.4 | 44.3 | 57.2 | 66.2 | 72.4 | 77.4 | 82.4 | 107.4 |
| G9 | Longquandadou | Heilongjiang,China | 48.3 | 128.1 | 47.1 | 10.1 | 3 | 39.5 | 73 | 17.8 | 24.4 | 35.3 | 40.0 | 51.7 | 56.1 | 61.6 | 72.3 | 111.7 |
| G10 | Xiaobaiqi | Liaoning,China | 40.8 | 122 | 102 | 16.6 | 4.6 | 73.7 | 124.6 | 20.8 | 16.7 | 47.0 | 52.1 | 59.2 | 65.9 | 70.8 | 77.8 | 97.8 |
| G11 | Gongye 04L-141 | Jilin,China | 43.4 | 126.3 | 90.9 | 18.8 | 1.6 | 58.3 | 132.7 | 14.7 | 11 | 37.0 | 47.5 | 62.3 | 66.3 | 71.3 | 76.3 | 98.3 |
| G12 | Tiedou 52 | Liaoning,China | 40.8 | 122 | 76.5 | 15.9 | 1.8 | 48.6 | 124 | 27.1 | 21.7 | 37.3 | 42.0 | 60.4 | 66.9 | 74.9 | 81.9 | 111.9 |
| G13 | L-57 | America | 43.4 | 79.3 | 121 | 18.7 | 2.2 | 60.8 | 79.3 | 18.3 | 22.5 | 43.0 | 47.2 | 64.1 | 70.1 | 76.1 | 82.1 | 112.1 |
| G14 | He 05-31 | Heilongjiang,China | 48.3 | 128.1 | 65.1 | 16.7 | 1.8 | 77.1 | 139.7 | 26 | 18.6 | 40.1 | 49.2 | 61.9 | 66.5 | 73.5 | 78.5 | 105.5 |
| G15 | L-21 | Canada | 43.4 | 79.3 | 81.2 | 16.6 | 0.9 | 26 | 56.5 | 10.6 | 18.6 | 33.7 | 39.8 | 55.0 | 60.5 | 69.4 | 75.1 | 102.1 |
| G16 | Hongfeng11 | Heilongjiang,China | 48.3 | 128.1 | 43.1 | 13.2 | 0 | 40.2 | 71.8 | 15.1 | 21.1 | 33.5 | 38.5 | 52.4 | 59.1 | 65.6 | 75.4 | 100.4 |
| G17 | Heinong 33 | Heilongjiang,China | 48.3 | 128.1 | 98.8 | 19.8 | 0.4 | 50.5 | 123.3 | 25.6 | 20.8 | 34.7 | 41.2 | 55.6 | 58.5 | 67.9 | 72.9 | 100.9 |
| G18 | Liao 98072 | Liaoning,China | 40.8 | 122 | 86.9 | 17 | 2.7 | 65 | 157.2 | 29.5 | 18.8 | 45.0 | 50.2 | 66.7 | 70.3 | 75.3 | 81.3 | 103.3 |
| G19 | Kenfeng 22 | Heilongjiang,China | 48.3 | 128.1 | 51.8 | 15.8 | 0.4 | 71.7 | 138.4 | 25 | 18.1 | 33.4 | 40.3 | 60.8 | 64.4 | 70.1 | 94.1 | 102.1 |
| G20 | Jiyu 89 | Jilin,China | 43.4 | 126.3 | 79.1 | 18.2 | 1.8 | 61.5 | 137 | 24.9 | 18.3 | 34.8 | 40.9 | 62.1 | 67.4 | 76.4 | 80.4 | 100.4 |
| G21 | Tiedou 51 | Liaoning,China | 40.8 | 122 | 87.1 | 18.4 | 2.5 | 87.4 | 176 | 40 | 22.8 | 36.9 | 49.8 | 62.8 | 69.8 | 78.8 | 103.8 | 124.0 |
| G22 | Tongnong 13 | Jilin,China | 43.4 | 126.3 | 72.8 | 14.8 | 0.4 | 38.3 | 97.3 | 26.3 | 27.1 | 33.4 | 46.0 | 59.0 | 64.0 | 72.3 | 78.3 | 104.3 |
| G23 | Bei 1873 | Heilongjiang,China | 48.3 | 128.1 | 41.8 | 9.9 | 0.6 | 11.8 | 25.5 | 5.8 | 22.6 | 32.8 | 38.4 | 45.4 | 50.7 | 56.0 | 68.2 | 101.2 |
| G24 | Hefeng 50 | Heilongjiang,China | 48.3 | 128.1 | 70.7 | 13.3 | 0 | 38.2 | 90 | 20.4 | 22.7 | 32.6 | 39.6 | 48.2 | 53.3 | 62.2 | 71.4 | 101.4 |
| G25 | Sui 02-339 | Heilongjiang,China | 48.3 | 128.1 | 86.2 | 18.9 | 2.6 | 47.2 | 90 | 26.1 | 28 | 33.8 | 47.8 | 62.9 | 69.5 | 72.2 | 79.2 | 102.2 |
| G26 | Hujiao 03-286 | Inner Mongolia,China | 44.5 | 111.7 | 45.9 | 12.5 | 0.1 | 35.3 | 64.3 | 11.7 | 18.1 | 33.1 | 37.9 | 48.8 | 54.3 | 61.2 | 69.7 | 101.6 |
| G27 | Heinong 44 | Heilongjiang,China | 48.3 | 128.1 | 60.5 | 14.9 | 0.4 | 61.7 | 128.4 | 24.5 | 19.1 | 32.8 | 38.4 | 53.0 | 59.2 | 66.2 | 73.7 | 94.7 |
| G28 | Hujiao 04-528 | Inner Mongolia,China | 44.5 | 111.7 | 47.1 | 13.9 | 0.5 | 46 | 93 | 17.6 | 18.8 | 32.0 | 38.1 | 49.5 | 54.7 | 69.1 | 102.1 | 103.4 |
| G29 | Fengshou 10 | Heilongjiang,China | 48.3 | 128.1 | 72.7 | 17.2 | 2.2 | 66.8 | 120.6 | 24.3 | 20 | 32.9 | 44.6 | 56.8 | 60.8 | 69.7 | 89.7 | 102.9 |
| G30 | Hersong 2 | Ukraine | 50.3 | 30.3 | 95.2 | 16.9 | 4.5 | 93.2 | 196.1 | 40.1 | 20.5 | 44.2 | 48.3 | 62.0 | 67.2 | 74.3 | 80.3 | 100.3 |
| G31 | Jinzhou 4-1 | Liaoning,China | 40.8 | 122 | 69.7 | 15 | 2.2 | 74.3 | 122.8 | 31 | 25.4 | 51.7 | 58.8 | 69.7 | 75.6 | 81.6 | 87.6 | 104.6 |
| G32 | Suinong 14 | Heilongjiang,China | 48.3 | 128.1 | 56.3 | 14.6 | 0.1 | 38.6 | 76.4 | 16.1 | 21.1 | 30.5 | 39.9 | 55.6 | 60.8 | 68.0 | 74.0 | 94.0 |
| G33 | Tiejiasilihuang | Jilin,China | 43.4 | 126.3 | 106 | 19.9 | 2.4 | 66.4 | 146.7 | 26.3 | 17.9 | 33.9 | 48.9 | 65.9 | 70.8 | 75.8 | 81.8 | 110.8 |
| G34 | Tiefeng 8 | Liaoning,China | 40.8 | 122 | 77.1 | 16 | 4 | 123.7 | 203.2 | 45.9 | 22.6 | 55.8 | 61.8 | 67.8 | 73.8 | 79.8 | 104.8 | 123.0 |
| G35 | Silihuang | Jilin,China | 43.4 | 126.3 | 98.4 | 18.3 | 1.6 | 61 | 118.3 | 24.5 | 20.4 | 33.0 | 42.4 | 57.8 | 61.7 | 68.6 | 73.6 | 93.6 |
| G36 | Bei 1361 | Heilongjiang,China | 48.3 | 128.1 | 62.3 | 15.6 | 2.9 | 85.1 | 154.6 | 34.7 | 22.4 | 32.4 | 44.3 | 54.2 | 59.4 | 68.3 | 74.3 | 97.3 |
| G37 | L-5 | Canada | 43.4 | 79.3 | 87.3 | 22.6 | 3.9 | 82.2 | 156.6 | 33.9 | 21.6 | 34.1 | 48.0 | 65.5 | 67.2 | 75.2 | 81.2 | 105.2 |
| G38 | Maoyandou | Gansu, China | 32.3 | 92.1 | 85.7 | 16.5 | 3.8 | 76.8 | 150.9 | 28.2 | 18.8 | 38.3 | 47.3 | 61.5 | 64.6 | 71.9 | 76.9 | 103.9 |
| G39 | Fengdihuang | Jilin,China | 43.4 | 126.3 | 116 | 20.8 | 2.8 | 75.2 | 137.5 | 28.5 | 20.9 | 48.5 | 57.4 | 70.3 | 73.0 | 78.0 | 83.0 | 111.0 |
| G40 | Tiedou 54 | Liaoning,China | 40.8 | 122 | 52.2 | 12.8 | 3.9 | 114 | 232.7 | 40.2 | 17.2 | 48.0 | 52.5 | 61.9 | 67.0 | 72.9 | 78.9 | 102.9 |
| G41 | Jiunong 20 | Jilin,China | 43.4 | 126.3 | 81.6 | 20.4 | 1.4 | 97.2 | 221.4 | 44.7 | 20.2 | 34.7 | 49.0 | 56.5 | 63.5 | 70.5 | 78.5 | 104.5 |
| G42 | Duludou | Jilin,China | 43.4 | 126.3 | 59.8 | 15.8 | 3.5 | 79.9 | 178.1 | 32.7 | 18.4 | 38.0 | 44.9 | 54.8 | 60.4 | 68.2 | 72.3 | 98.3 |
| G43 | Sui 03-3046 | Heilongjiang,China | 48.3 | 128.1 | 48 | 12.2 | 0.7 | 46.1 | 89.5 | 15.2 | 17 | 31.1 | 37.7 | 50.2 | 53.6 | 61.3 | 70.6 | 100.6 |
| G44 | Hefeng 47 | Heilongjiang,China | 48.3 | 128.1 | 45.8 | 14.4 | 0 | 32.9 | 72.3 | 13.6 | 18.9 | 31.1 | 38.0 | 49.9 | 56.7 | 61.3 | 71.3 | 101.3 |
| G45 | V111-4 | Jilin,China | 43.4 | 126.3 | 91.5 | 18.6 | 1.7 | 66.9 | 121.6 | 30.3 | 24.9 | 42.9 | 49.9 | 67.9 | 72.9 | 77.9 | 82.9 | 107.9 |
| G46 | Longxuan 1 | Heilongjiang,China | 48.3 | 128.1 | 77.6 | 16.8 | 0.8 | 48.7 | 105.8 | 21.9 | 20.6 | 35.6 | 45.8 | 62.2 | 70.0 | 77.7 | 81.7 | 101.1 |
| G47 | Dongnong 8004 | Heilongjiang,China | 48.3 | 128.1 | 50.9 | 15.5 | 0.4 | 33.5 | 60.1 | 12.6 | 20.9 | 30.7 | 36.5 | 43.0 | 50.3 | 54.6 | 69.1 | 103.5 |
| G48 | Sui 03-3952 | Heilongjiang,China | 48.3 | 128.1 | 64.6 | 16.9 | 0.2 | 69.7 | 132.2 | 22.7 | 17.2 | 31.4 | 38.4 | 53.7 | 58.4 | 66.2 | 73.9 | 103.9 |
| G49 | Hefeng 25 | Heilongjiang,China | 48.3 | 128.1 | 64.4 | 17 | 1.1 | 48.6 | 86.3 | 18.1 | 20.9 | 33.7 | 41.4 | 59.6 | 62.6 | 68.9 | 77.6 | 94.7 |
| G50 | Liaodou 3 | Liaoning,China | 40.8 | 122 | 102 | 17.8 | 2.2 | 70.8 | 122.6 | 28.3 | 22.9 | 36.7 | 46.8 | 61.8 | 67.9 | 73.9 | 79.9 | 108.9 |
| G51 | Hujiao 423 | Inner Mongolia,China | 44.5 | 111.7 | 52.4 | 13.5 | 0.5 | 36.2 | 77.8 | 15.8 | 20.3 | 33.3 | 39.1 | 54.7 | 59.5 | 66.9 | 74.5 | 103.4 |
| G52 | Liaoxian 1 | Liaoning,China | 40.8 | 122 | 19.8 | 7 | 5 | 33.4 | 60.9 | 22.5 | 28.3 | 29.0 | 36.0 | 42.5 | 50.5 | 56.2 | 61.2 | 109.8 |
| G53 | Zhongpin 95-5388 | Beijing,China | 40.1 | 116.3 | 105 | 20.8 | 4.6 | 143.9 | 216.5 | 71.9 | 18.6 | 51.7 | 58.0 | 70.8 | 75.8 | 80.8 | 85.8 | 106.8 |
| G54 | Heihe 45 | Heilongjiang,China | 48.3 | 128.1 | 41.3 | 15 | 0.2 | 35.7 | 64.5 | 12.4 | 19.2 | 31.2 | 38.4 | 43.9 | 49.9 | 56.4 | 71.2 | 98.6 |
| G55 | L-59 Peking | America | 41.8 | 92.9 | 127 | 22.5 | 7.5 | 140.3 | 254.2 | 25.5 | 10 | 68.1 | 78.0 | 88.0 | 93.0 | 101.0 | 108.0 | 123.6 |
| G56 | Sui 04-5804 | Heilongjiang,China | 48.3 | 128.1 | 69.4 | 15.9 | 0 | 40.9 | 87.9 | 19.3 | 22 | 33.5 | 39.8 | 56.2 | 60.9 | 68.6 | 75.1 | 101.1 |
| G57 | Ji 06B7 | Hebei,China | 39.2 | 116.3 | 114 | 25.6 | 2.3 | 80.6 | 145 | 25.8 | 17.9 | 64.0 | 71.0 | 76.0 | 82.0 | 88.0 | 114.0 | 124.5 |
| G58 | Marca. Joe lisa | Heilongjiang,China | 48.3 | 128.1 | 92.8 | 19.9 | 2.9 | 91.2 | 148.9 | 17.8 | 11.9 | 52.1 | 57.4 | 69.4 | 75.4 | 82.4 | 89.4 | 115.4 |
| G59 | Jihuang 13 | Hebei,China | 39.2 | 116.3 | 111 | 21.4 | 1.9 | 75.4 | 193.9 | 38.3 | 19.7 | 43.9 | 53.1 | 69.2 | 74.7 | 80.7 | 86.7 | 111.7 |
| G60 | L-28 | Canada | 43.4 | 79.3 | 84.7 | 16.3 | 0.9 | 44 | 93.8 | 17.2 | 18.5 | 34.5 | 41.4 | 55.9 | 60.2 | 67.4 | 70.8 | 97.8 |
| G61 | Beifeng 9 | Heilongjiang,China | 48.3 | 128.1 | 51.6 | 13.6 | 0.2 | 50.1 | 110.2 | 18.6 | 17.1 | 31.7 | 38.4 | 51.2 | 55.9 | 62.2 | 72.6 | 96.3 |
| G62 | Zhongzuo 00-683 | Beijing,China | 40.1 | 116.3 | 111 | 20.3 | 2.6 | 80.2 | 148.6 | 29.5 | 19.7 | 50.8 | 56.5 | 71.4 | 76.4 | 81.4 | 87.4 | 110.4 |
| G63 | Dongnong 50 | Heilongjiang,China | 48.3 | 128.1 | 54.8 | 18 | 6.5 | 150.5 | 311.1 | 33.9 | 8 | 31.8 | 43.7 | 56.7 | 60.8 | 68.0 | 75.1 | 98.1 |
| G64 | Dongnong 93-046 | Heilongjiang,China | 48.3 | 128.1 | 116 | 24.1 | 1.8 | 70 | 136.2 | 30.9 | 23 | 32.4 | 40.4 | 56.4 | 60.4 | 69.4 | 71.4 | 100.4 |
| G65 | Suinong 10 | Heilongjiang,China | 48.3 | 128.1 | 69.4 | 16.3 | 0.3 | 38.2 | 86.4 | 16.8 | 19.3 | 32.0 | 39.5 | 57.6 | 63.1 | 65.6 | 74.7 | 100.7 |
| G66 | Tiefeng 31 | Liaoning,China | 40.8 | 122 | 90.3 | 21.9 | 3.2 | 119.7 | 254 | 49.9 | 19.6 | 46.3 | 52.7 | 72.3 | 78.3 | 82.3 | 88.3 | 105.3 |
| G67 | Dongnong 49 | Heilongjiang,China | 48.3 | 128.1 | 47 | 14.6 | 0 | 44.1 | 84.7 | 14.5 | 17.2 | 31.6 | 37.9 | 46.8 | 53.2 | 58.4 | 70.5 | 98.4 |
| G68 | Huayou 446 | Hebei,China | 39.2 | 116.3 | 106 | 25.1 | 3.2 | 101.8 | 246.1 | 55.8 | 22.5 | 52.1 | 59.4 | 70.8 | 75.0 | 83.0 | 91.0 | 116.0 |
| G69 | Heihe 48 | Heilongjiang,China | 48.3 | 128.1 | 52.6 | 13.9 | 0.1 | 39 | 71 | 11.3 | 15.9 | 32.7 | 37.6 | 51.4 | 55.5 | 61.0 | 71.4 | 96.9 |
| G70 | Huajiang 2 | Heilongjiang,China | 48.3 | 128.1 | 47.5 | 15.7 | 0.5 | 42.7 | 81.7 | 13.5 | 16.6 | 31.5 | 36.7 | 42.9 | 51.5 | 58.8 | 69.4 | 97.7 |
| G71 | Suinong 29 | Heilongjiang,China | 48.3 | 128.1 | 58.3 | 14 | 0.1 | 15.4 | 40.7 | 9 | 22.1 | 32.4 | 38.7 | 54.4 | 62.0 | 69.0 | 74.0 | 99.0 |
| G72 | Hefeng 35 | Heilongjiang,China | 48.3 | 128.1 | 51.6 | 15.3 | 0.2 | 38.2 | 78.7 | 17.8 | 22.7 | 32.4 | 39.6 | 52.7 | 58.5 | 62.2 | 70.8 | 100.8 |
| G73 | Suinong 8 | Heilongjiang,China | 48.3 | 128.1 | 71.8 | 13.9 | 0.2 | 29 | 59 | 13.4 | 22.9 | 32.9 | 39.8 | 56.5 | 61.1 | 68.5 | 73.5 | 98.5 |
| G74 | Jilin 47 | Jilin,China | 43.4 | 126.3 | 74.1 | 18.1 | 0.6 | 68.2 | 117.6 | 24.4 | 20.7 | 37.9 | 46.7 | 60.5 | 67.1 | 72.7 | 77.7 | 97.7 |
| G75 | Heinong 48 | Heilongjiang,China | 48.3 | 128.1 | 69.3 | 13.2 | 1.1 | 42.8 | 104.5 | 27 | 26 | 33.9 | 40.3 | 59.0 | 64.8 | 70.8 | 75.8 | 99.8 |
| G76 | Nova | Italy | 41.5 | 12.3 | 101 | 16.9 | 0.9 | 59.5 | 121.3 | 31.1 | 25.8 | 37.4 | 48.4 | 60.1 | 67.1 | 76.1 | 82.1 | 107.1 |
| G77 | Beidou 14 | Heilongjiang,China | 48.3 | 128.1 | 65.4 | 16.9 | 1.7 | 47.9 | 100 | 17.3 | 17.3 | 31.9 | 38.5 | 52.2 | 56.8 | 63.4 | 72.9 | 98.9 |
| G78 | Kangxian 3 | Heilongjiang,China | 48.3 | 128.1 | 96.4 | 20.7 | 1.2 | 64.6 | 129.7 | 23 | 17.8 | 32.6 | 47.2 | 63.5 | 67.9 | 74.0 | 79.0 | 99.0 |
| G79 | Dongnong 42 | Heilongjiang,China | 48.3 | 128.1 | 90.4 | 17.1 | 1.4 | 70.1 | 132.2 | 30.6 | 23.1 | 34.0 | 39.5 | 56.2 | 60.2 | 68.2 | 73.2 | 95.2 |
| G80 | Kenjian 23 | Heilongjiang,China | 48.3 | 128.1 | 58.7 | 16.6 | 0.1 | 46.6 | 106.5 | 22.7 | 21.2 | 31.2 | 37.3 | 52.0 | 57.6 | 63.0 | 71.7 | 99.2 |
| G81 | Heinong 55 | Heilongjiang,China | 48.3 | 128.1 | 63.7 | 16.6 | 1.2 | 73.8 | 163.1 | 33.6 | 21 | 34.7 | 40.1 | 58.4 | 61.0 | 65.9 | 74.0 | 100.0 |
| G82 | Hefeng 45 | Heilongjiang,China | 48.3 | 128.1 | 81.5 | 21.4 | 2.1 | 56.3 | 108.7 | 22 | 20.2 | 33.0 | 38.4 | 55.6 | 60.6 | 70.8 | 76.4 | 100.4 |
| G83 | Kenfeng 15 | Heilongjiang,China | 48.3 | 128.1 | 52.4 | 15.6 | 0.1 | 44.5 | 89.5 | 14 | 15.6 | 32.7 | 39.0 | 52.3 | 54.6 | 62.1 | 72.7 | 101.1 |
| G84 | Chasedou | Jilin,China | 43.4 | 126.3 | 67.6 | 12.9 | 0.4 | 26.9 | 60.4 | 11.6 | 19.3 | 34.0 | 43.9 | 59.7 | 66.6 | 73.8 | 79.8 | 101.8 |
| G85 | He 05-991 | Heilongjiang,China | 48.3 | 128.1 | 46.4 | 14.4 | 0.1 | 67.1 | 132.5 | 23.5 | 17.5 | 33.0 | 38.9 | 55.6 | 59.4 | 66.1 | 73.0 | 103.0 |
| G86 | Xiaolimoshidou | Heilongjiang,China | 39.2 | 116.3 | 69.1 | 21.3 | 6.3 | 157 | 304.9 | 31.9 | 7.4 | 39.4 | 47.4 | 58.1 | 61.9 | 68.9 | 77.9 | 98.9 |
| G87 | Jingshanpu | Heilongjiang,China | 48.3 | 128.1 | 55.6 | 12.1 | 1.1 | 22.4 | 56.1 | 9.8 | 17.5 | 34.0 | 46.9 | 60.2 | 63.0 | 68.6 | 77.6 | 100.6 |
| G88 | Qinganheidou | Heilongjiang,China | 48.3 | 128.1 | 123 | 19.2 | 2.7 | 55.4 | 102.9 | 16.9 | 16.6 | 41.7 | 49.7 | 68.4 | 74.4 | 80.4 | 86.4 | 106.4 |
| G89 | Dongnong594 | Heilongjiang,China | 48.3 | 128.1 | 60.3 | 18.1 | 1 | 67.4 | 132.2 | 29.3 | 22.5 | 33.6 | 42.4 | 61.5 | 64.8 | 74.7 | 77.7 | 106.9 |
| G90 | Yapoche | Heilongjiang,China | 48.3 | 128.1 | 101 | 20.4 | 1.3 | 60.5 | 110.9 | 20.4 | 18.8 | 33.3 | 42.2 | 59.9 | 63.7 | 72.8 | 78.8 | 84.8 |
| G91 | Kennong 29 | Heilongjiang,China | 48.3 | 128.1 | 57.4 | 14.3 | 0.7 | 53.5 | 110.9 | 20 | 18.1 | 32.1 | 39.5 | 53.0 | 56.5 | 60.9 | 71.2 | 102.0 |
| G92 | Heimoshidou | Jilin,China | 43.4 | 126.3 | 132 | 19.2 | 6 | 143.6 | 249.4 | 34.6 | 13.9 | 50.9 | 58.0 | 68.6 | 73.6 | 79.6 | 85.6 | 105.6 |
| G93 | Ha 04-1824 | Heilongjiang,China | 48.3 | 128.1 | 67.9 | 15.1 | 0.6 | 50.4 | 130.1 | 23.4 | 18.2 | 32.7 | 40.6 | 57.3 | 68.1 | 75.1 | 101.1 | 104.7 |
| G94 | Fangzhengmoshidou | Heilongjiang,China | 48.3 | 128.1 | 111 | 21.8 | 4.4 | 140.4 | 261.8 | 38.6 | 14.9 | 41.9 | 49.0 | 68.5 | 73.5 | 78.5 | 84.5 | 104.5 |
| G95 | Helongyoutai | Jilin,China | 43.4 | 126.3 | 96.7 | 15.4 | 3.1 | 48.9 | 84.5 | 18.9 | 22.9 | 34.3 | 40.4 | 57.8 | 61.9 | 68.2 | 74.8 | 94.8 |
| G96 | Kexin 3 | Beijing,China | 40.1 | 116.3 | 104 | 21.1 | 3 | 79 | 138.3 | 33.7 | 24.3 | 54.2 | 59.6 | 64.6 | 69.6 | 75.6 | 81.6 | 109.6 |
| G97 | Yuanbaojin | Heilongjiang,China | 48.3 | 128.1 | 98.9 | 15.2 | 2.6 | 43.3 | 83.1 | 19.6 | 23.6 | 33.3 | 40.7 | 57.6 | 62.0 | 67.2 | 75.2 | 98.2 |
| G98 | Zhongzuo J4133 | Beijing,China | 40.1 | 116.3 | 98 | 19.2 | 1.4 | 70.9 | 118.4 | 25.5 | 21.5 | 50.7 | 59.3 | 71.8 | 73.5 | 79.5 | 85.5 | 108.5 |
| G99 | Jilin 30 | Jilin,China | 43.4 | 126.3 | 77.3 | 19.4 | 3.2 | 82.8 | 210.9 | 36.2 | 17.2 | 45.5 | 51.6 | 64.8 | 73.0 | 78.0 | 84.0 | 104.0 |
| G100 | Ji 100 | Jilin,China | 43.4 | 126.3 | 77.4 | 15.5 | 0.9 | 80.7 | 179.8 | 36.1 | 20.1 | 38.0 | 46.8 | 60.3 | 64.5 | 68.5 | 74.5 | 94.5 |
| G101 | Suinong 30 | Heilongjiang,China | 48.3 | 128.1 | 56.8 | 16.3 | 0.4 | 61 | 131.6 | 25.6 | 19.6 | 33.5 | 41.9 | 51.8 | 56.4 | 60.8 | 71.1 | 98.1 |
| G102 | Boige du | Germany | 52.3 | 13.2 | 97.2 | 17.4 | 5.6 | 125.6 | 215.5 | 29.3 | 13.6 | 35.8 | 40.2 | 59.6 | 63.1 | 68.3 | 77.9 | 99.9 |
| G103 | Beidou 16 | Heilongjiang,China | 48.3 | 128.1 | 80.8 | 17.6 | 3.4 | 98.2 | 213.5 | 41.7 | 19.2 | 42.0 | 48.9 | 59.2 | 66.8 | 72.6 | 77.6 | 97.6 |
| G104 | Kebei 1 | Heilongjiang,China | 48.3 | 128.1 | 79 | 18.7 | 1.4 | 71.7 | 128.8 | 24.9 | 19.4 | 32.5 | 41.4 | 60.2 | 60.9 | 68.6 | 76.4 | 98.4 |
| G105 | Mengdou 21 | Inner Mongolia,China | 44.5 | 111.7 | 53 | 13.9 | 0.1 | 28.9 | 59.7 | 11 | 18.4 | 31.4 | 39.4 | 46.8 | 55.5 | 59.6 | 71.0 | 99.1 |
| G106 | Heihe 38 | Heilongjiang,China | 48.3 | 128.1 | 55.9 | 11.9 | 0.2 | 23.3 | 51.1 | 11.1 | 21.6 | 32.2 | 38.7 | 50.6 | 55.9 | 60.7 | 71.2 | 95.9 |
| G107 | Zhongdou 35 | Beijing,China | 40.1 | 116.3 | 114 | 23.8 | 2.9 | 89.3 | 298 | 70.1 | 23.6 | 37.1 | 40.9 | 60.7 | 69.8 | 73.5 | 79.5 | 105.5 |
| G108 | Zhonghuang 6 | Beijing,China | 40.1 | 116.3 | 111 | 21.1 | 2.9 | 62.2 | 102.6 | 18 | 17.1 | 51.6 | 55.9 | 70.4 | 76.4 | 82.4 | 88.4 | 113.4 |
| G109 | Jiyu 94 | Jilin,China | 43.4 | 126.3 | 59.8 | 12 | 1.3 | 35.1 | 117.6 | 20.8 | 17.5 | 53.0 | 58.3 | 63.1 | 66.7 | 73.2 | 78.2 | 98.2 |
| G110 | Dongnong 44 | Heilongjiang,China | 48.3 | 128.1 | 47.8 | 13.8 | 0.5 | 36.1 | 69 | 12.7 | 18.4 | 31.7 | 37.7 | 46.4 | 53.2 | 57.9 | 67.3 | 103.6 |
| G111 | Liaonong 2 | Liaoning,China | 40.8 | 122 | 42.9 | 13.1 | 2 | 57.1 | 108.3 | 20.3 | 18.7 | 32.9 | 44.9 | 61.8 | 67.1 | 73.0 | 79.0 | 103.0 |
| G112 | Bei 1484 | America | 48.3 | 128.1 | 62.5 | 17.7 | 1.5 | 59.9 | 120.9 | 22.6 | 18.7 | 32.4 | 38.8 | 48.3 | 56.5 | 64.2 | 70.7 | 92.7 |
| G113 | Williams82 | America | 41.8 | 92.9 | 84.4 | 15.3 | 2.9 | 51.6 | 117 | 20.5 | 17.7 | 42.3 | 48.2 | 61.8 | 66.3 | 71.3 | 76.3 | 103.3 |
| G114 | Jindou 33 | Liaoning,China | 40.8 | 122 | 93 | 15.6 | 2.6 | 72.1 | 150.3 | 34.7 | 23.5 | 55.4 | 62.4 | 70.6 | 75.6 | 80.6 | 86.6 | 114.6 |
| G115 | Kenfeng 18 | Heilongjiang,China | 48.3 | 128.1 | 88.5 | 18.9 | 1 | 47.3 | 101.1 | 21.7 | 21.5 | 32.7 | 39.6 | 54.7 | 62.0 | 72.9 | 78.8 | 102.8 |
| G116 | Suinong 20 | Heilongjiang,China | 48.3 | 128.1 | 61.8 | 14.8 | 0.3 | 40.8 | 86.8 | 18.2 | 20.9 | 31.2 | 37.4 | 53.2 | 57.5 | 63.1 | 71.4 | 99.4 |
| G117 | Mengdou 9 | Inner Mongolia,China | 44.5 | 111.7 | 56.4 | 12 | 0.4 | 32.8 | 56.8 | 13.5 | 23.9 | 32.6 | 37.8 | 50.9 | 55.3 | 60.6 | 69.1 | 99.9 |
| G118 | L-9 | America | 41.8 | 92.9 | 109 | 25.9 | 3.8 | 132 | 239.1 | 45.9 | 19.2 | 63.0 | 70.4 | 80.4 | 86.4 | 93.4 | 112.4 | 124.1 |
| G119 | Suinong 1 | Heilongjiang,China | 48.3 | 128.1 | 73.5 | 19.2 | 2.8 | 95.5 | 225.5 | 46.1 | 19.8 | 33.8 | 44.9 | 57.1 | 61.6 | 68.6 | 74.6 | 100.6 |
| G120 | Kenfeng 14 | Heilongjiang,China | 48.3 | 128.1 | 75.3 | 18.2 | 1.8 | 66.9 | 154 | 33.1 | 21.5 | 32.7 | 41.9 | 55.2 | 62.2 | 71.7 | 77.5 | 101.5 |
| G121 | Tiejiazi | Liaoning,China | 40.8 | 122 | 79.7 | 15.9 | 0.6 | 49.8 | 104 | 22.5 | 21.7 | 32.7 | 43.2 | 52.5 | 58.2 | 63.2 | 69.2 | 95.2 |
| G122 | Suinong 4 | Heilongjiang,China | 48.3 | 128.1 | 66.9 | 18.8 | 0.7 | 41 | 73.7 | 16 | 21.7 | 31.4 | 40.5 | 55.0 | 61.2 | 70.3 | 77.1 | 104.1 |
| G123 | Jinshanchamoshidou | Jilin,China | 43.4 | 126.3 | 125 | 19.2 | 5.5 | 133.1 | 237.1 | 31.3 | 13.3 | 55.9 | 61.2 | 71.2 | 76.2 | 81.2 | 86.2 | 106.2 |
| G124 | Longpin 03-311 | Heilongjiang,China | 48.3 | 128.1 | 77.4 | 31.9 | 39 | 57.7 | 61.6 | 66.6 | 74.1 | 95.1 | 39 | 57.7 | 61.6 | 66.6 | 74.1 | 95.1 |
| G125 | Jilinchalihua | Jilin,China | 43.4 | 126.3 | 103 | 50.2 | 54.2 | 66.6 | 79.4 | 82.4 | 116.2 | 123.2 | 54.2 | 66.6 | 79.4 | 82.4 | 116.2 | 123.2 |
| G126 | Heinong 37 | Heilongjiang,China | 48.3 | 128.1 | 58.9 | 33.5 | 42.1 | 56.9 | 62.5 | 70.2 | 75.8 | 96.8 | 42.1 | 56.9 | 62.5 | 70.2 | 75.8 | 96.8 |
| G127 | Huajiang 4403 | Heilongjiang,China | 48.3 | 128.1 | 54.3 | 32.1 | 38.2 | 49 | 55.3 | 59.8 | 70.7 | 100.7 | 38.2 | 49 | 55.3 | 59.8 | 70.7 | 100.7 |
| G128 | Huangbaozhu | Liaoning,China | 40.8 | 122 | 125 | 46.5 | 54.5 | 60.5 | 66.5 | 74.5 | 80.5 | 103.5 | 54.5 | 60.5 | 66.5 | 74.5 | 80.5 | 103.5 |
| G129 | Suinong 25 | Heilongjiang,China | 48.3 | 128.1 | 77.7 | 19 | 1.4 | 67.3 | 123 | 26.4 | 21 | 31.9 | 45.8 | 58.8 | 62.8 | 70.0 | 80.6 | 97.6 |
| G130 | Sui 05-7304 | Heilongjiang,China | 48.3 | 128.1 | 50.4 | 13.2 | 0 | 32.6 | 69.2 | 14.8 | 21.3 | 32.5 | 39.6 | 49.3 | 55.8 | 60.0 | 69.1 | 104.0 |
| G131 | Jidou 9 | Hebei,China | 39.2 | 116.3 | 98.2 | 24.5 | 4.2 | 145.9 | 306.4 | 71.6 | 21.7 | 41.6 | 52.3 | 68.8 | 73.9 | 79.9 | 89.9 | 114.9 |
| G132 | Mengdou 14 | Inner Mongolia,China | 44.5 | 111.7 | 60.8 | 17.3 | 1.1 | 46.1 | 100.7 | 18 | 17.9 | 33.0 | 39.9 | 56.3 | 62.8 | 69.0 | 77.0 | 101.0 |
| G133 | Handou 5 | Hebei,China | 39.2 | 116.3 | 110 | 22.1 | 3 | 93 | 197.6 | 50.9 | 25.8 | 44.4 | 50.0 | 68.4 | 73.4 | 79.4 | 108.4 | 123.0 |
| G134 | Jidou 17 | Hebei,China | 39.2 | 116.3 | 124 | 22.1 | 6 | 144.6 | 307.6 | 70.9 | 21 | 49.7 | 56.6 | 69.7 | 72.5 | 82.5 | 84.5 | 119.5 |
| G135 | Aika 166 | Heilongjiang,China | 44.2 | 26.1 | 62.4 | 18.1 | 3.3 | 92.7 | 179.1 | 36.3 | 20.2 | 32.9 | 48.1 | 62.9 | 69.4 | 75.5 | 80.5 | 98.5 |
| G136 | Sui 04-6018 | Heilongjiang,China | 48.3 | 128.1 | 59.4 | 18.5 | 0.3 | 45.8 | 88.4 | 17.1 | 19.3 | 32.7 | 38.1 | 53.5 | 58.2 | 63.6 | 75.6 | 100.6 |
| G137 | Dongnong 48 | Heilongjiang,China | 48.3 | 128.1 | 48.6 | 13.9 | 0.4 | 48.8 | 108.5 | 22.4 | 21.1 | 33.6 | 40.3 | 55.7 | 58.8 | 62.6 | 71.6 | 100.3 |
| G138 | L-79 | America | 43.4 | 79.3 | 57.2 | 16.1 | 1.7 | 51.5 | 87.1 | 18.2 | 20.9 | 33.1 | 38.7 | 46.5 | 54.4 | 63.2 | 72.1 | 103.3 |
| G139 | Zhonghuang 35 | Beijing,China | 40.1 | 116.3 | 74.2 | 17.8 | 2.1 | 64.6 | 114.6 | 23.3 | 20.3 | 33.8 | 42.1 | 58.9 | 63.1 | 68.5 | 75.5 | 105.5 |
| G140 | Kennong 30 | Heilongjiang,China | 48.3 | 128.1 | 61.2 | 15.6 | 1.6 | 66.3 | 137 | 22.5 | 16.4 | 31.5 | 37.7 | 52.2 | 58.5 | 61.1 | 71.7 | 99.7 |
| G141 | Zhonghuang 30 | Beijing,China | 40.1 | 116.3 | 71.4 | 16 | 2.6 | 126.9 | 298.8 | 62.7 | 21.1 | 49.5 | 55.3 | 68.8 | 75.8 | 82.8 | 85.8 | 99.8 |
| G142 | Baichengmoshidou | Jilin,China | 43.4 | 126.3 | 111 | 17.2 | 4.9 | 108.7 | 179 | 21.2 | 11.9 | 48.6 | 53.3 | 66.5 | 69.8 | 74.8 | 79.8 | 99.8 |
| G143 | L-10 | America | 43.4 | 79.3 | 82.3 | 20.2 | 1.5 | 67.7 | 124.1 | 21 | 16.9 | 34.6 | 46.9 | 56.8 | 67.7 | 77.9 | 84.9 | 110.9 |
| G144 | Zaoshu 18 | Beijing,China | 40.1 | 116.3 | 72.4 | 18.6 | 3.6 | 62.4 | 128.5 | 29.6 | 22.7 | 47.3 | 54.4 | 72.4 | 86.4 | 107.4 | 112.4 | 123.9 |
| G145 | Zhonghuang 10 | Beijing,China | 40.1 | 116.3 | 92.9 | 17.2 | 3.2 | 112.3 | 204.2 | 35.4 | 17.3 | 51.8 | 57.8 | 70.3 | 74.5 | 78.5 | 84.4 | 111.4 |
| G146 | Dongnong L-13 | Heilongjiang,China | 43.4 | 79.3 | 113 | 19.5 | 1.7 | 62.4 | 114.3 | 28.2 | 24.7 | 32.6 | 44.1 | 61.6 | 64.9 | 73.0 | 78.0 | 106.0 |
| G147 | Neifeng 15 | Heilongjiang,China | 48.3 | 128.1 | 81.6 | 16.4 | 1.6 | 49.1 | 87.3 | 16.7 | 19.2 | 32.9 | 40.3 | 59.8 | 63.9 | 71.3 | 81.2 | 102.2 |
| G148 | Dongnong 47 | Heilongjiang,China | 48.3 | 128.1 | 70.2 | 16.5 | 1.7 | 73.1 | 161.6 | 28.2 | 17.6 | 31.6 | 42.0 | 60.7 | 65.1 | 71.1 | 76.4 | 100.4 |
| G149 | Hefeng 29 | Heilongjiang,China | 48.3 | 128.1 | 87.7 | 19.2 | 2.7 | 89.1 | 196.4 | 41.2 | 20.9 | 39.0 | 47.1 | 61.9 | 64.3 | 70.2 | 76.2 | 100.2 |
| G150 | Dongnong 07-909 | Heilongjiang,China | 48.3 | 128.1 | 60.6 | 17.4 | 1.7 | 51.2 | 102.6 | 18.4 | 17.9 | 31.2 | 39.4 | 56.5 | 60.1 | 67.0 | 74.8 | 96.8 |
| G151 | Hefeng 52 | Heilongjiang,China | 48.3 | 128.1 | 59 | 15.5 | 1.2 | 79.2 | 153.7 | 25.6 | 16.6 | 30.7 | 41.1 | 58.6 | 61.4 | 66.5 | 74.5 | 100.5 |
| G152 | Heihe 18 | Heilongjiang,China | 48.3 | 128.1 | 39.7 | 11.4 | 0.3 | 36.5 | 68.4 | 13.6 | 20 | 30.2 | 36.0 | 42.7 | 48.1 | 54.1 | 61.3 | 98.2 |
| G153 | Neifeng 11 | Heilongjiang,China | 48.3 | 128.1 | 51.5 | 13 | 0.1 | 39.7 | 89.5 | 14.6 | 16.3 | 30.0 | 37.8 | 52.6 | 56.7 | 62.5 | 70.6 | 93.7 |
| G154 | Heihexiaohuangdou | Heilongjiang,China | 48.3 | 128.1 | 61.5 | 14.5 | 1.7 | 47.7 | 73.5 | 15.3 | 20.9 | 32.2 | 39.5 | 51.7 | 56.1 | 60.6 | 71.7 | 95.7 |
| G155 | Zhongdou 27 | Beijing,China | 40.1 | 116.3 | 104 | 22.1 | 4.1 | 117.2 | 242.2 | 57.5 | 23.7 | 46.1 | 57.2 | 69.6 | 73.7 | 78.7 | 83.7 | 105.7 |
| G156 | Bei 4834 | Heilongjiang,China | 48.3 | 128.1 | 42.1 | 10.2 | 4.1 | 54.5 | 103.1 | 31.4 | 28.1 | 33.7 | 38.0 | 48.1 | 54.5 | 59.8 | 69.6 | 105.7 |
| G157 | Heilongjiang 41 | Heilongjiang,China | 48.3 | 128.1 | 88.4 | 18 | 1.8 | 69.1 | 145.5 | 32.8 | 22.7 | 40.2 | 47.8 | 62.7 | 66.0 | 74.0 | 79.0 | 99.0 |
| G158 | Zhongpin 03-5179 | Beijing,China | 40.1 | 116.3 | 108 | 19.6 | 2.1 | 84.2 | 187.2 | 47.2 | 25.3 | 33.7 | 40.0 | 61.1 | 65.9 | 70.9 | 75.9 | 103.9 |
| G159 | Hefeng 37 | Heilongjiang,China | 48.3 | 128.1 | 114 | 25.4 | 3.5 | 95.4 | 177.7 | 36.9 | 20.8 | 61.3 | 68.0 | 74.0 | 80.0 | 87.0 | 110.0 | 123.1 |
| G160 | Dongnong 46 | Heilongjiang,China | 48.3 | 128.1 | 70.7 | 14.6 | 0.6 | 36.8 | 91.6 | 21.4 | 23.2 | 34.2 | 40.0 | 59.7 | 62.1 | 68.4 | 76.3 | 102.3 |
| G161 | Dongnong 43 | Heilongjiang,China | 48.3 | 128.1 | 51.9 | 17 | 1.8 | 42.1 | 66.9 | 12.9 | 19.3 | 34.6 | 41.3 | 57.2 | 61.7 | 69.8 | 78.6 | 100.6 |
| G162 | Dongnong 1068 | Heilongjiang,China | 48.3 | 128.1 | 69.1 | 16.6 | 2.6 | 92.8 | 188.6 | 39.4 | 20.3 | 32.9 | 40.2 | 55.0 | 58.7 | 66.1 | 72.1 | 96.1 |
| G163 | Dongnong 56 | Heilongjiang,China | 48.3 | 128.1 | 56.7 | 16 | 0.3 | 69.1 | 134.5 | 23.6 | 17.5 | 33.4 | 43.1 | 60.9 | 63.8 | 70.5 | 78.4 | 103.4 |
| G164 | Jiunong 21 | Jilin,China | 43.4 | 126.3 | 83.3 | 21 | 4.9 | 151.6 | 309.1 | 63.3 | 15.8 | 34.8 | 47.9 | 57.1 | 66.9 | 73.9 | 79.9 | 102.9 |
| G165 | Chamoshidou | Jilin,China | 43.4 | 126.3 | 130 | 22.6 | 7.9 | 159.6 | 305.9 | 35.5 | 7.9 | 50.0 | 59.9 | 70.8 | 84.8 | 88.8 | 99.8 | 123.5 |
| G166 | Daliheidou | Shandong,China | 36.2 | 118.3 | 104 | 16.3 | 1.9 | 48.6 | 75.9 | 15.1 | 19.3 | 34.2 | 45.1 | 63.2 | 69.8 | 75.8 | 81.8 | 108.8 |
| G167 | Zhongpin 03-5373 | Beijing,China | 40.1 | 116.3 | 70.8 | 17 | 4.7 | 146.7 | 308.8 | 68.2 | 17 | 58.5 | 62.5 | 73.5 | 83.5 | 91.5 | 110.5 | 124.5 |
| G168 | Wuxing 4 | Hebei,China | 39.2 | 116.3 | 19.8 | 2.3 | 78.2 | 125.7 | 32.2 | 25.5 | 62.6 | 67.7 | 73.7 | 79.7 | 86.7 | 106.7 | 124.4 | 19.8 |
| G169 | Hefeng 55 | Heilongjiang,China | 48.3 | 128.1 | 17.9 | 0.1 | 36 | 57.9 | 11.7 | 20.1 | 33.3 | 40.7 | 62.3 | 68.3 | 75.3 | 80.3 | 98.3 | 17.9 |
| G170 | Suinong 28 | Heilongjiang,China | 48.3 | 128.1 | 14.1 | 0.6 | 45.1 | 97.3 | 18 | 18.5 | 32.7 | 38.5 | 52.5 | 56.9 | 63.4 | 72.5 | 97.5 | 14.1 |
| G171 | Zhongpin 03-5334 | Beijing,China | 40.1 | 116.3 | 14.1 | 7.3 | 149.5 | 302.2 | 49.4 | 15 | 53.8 | 58.4 | 70.6 | 86.8 | 92.8 | 108.8 | 122.0 | 14.1 |
| G172 | Fengshou 6 | Heilongjiang,China | 48.3 | 128.1 | 15.3 | 1.3 | 32.2 | 74.5 | 12.9 | 17.3 | 31.9 | 41.8 | 58.4 | 63.2 | 68.5 | 77.5 | 100.5 | 15.3 |
| G173 | Dunajika | Russia | 61.5 | 98 | 14.5 | 3.2 | 69.5 | 137.5 | 18 | 13.1 | 31.4 | 37.5 | 58.5 | 60.5 | 66.3 | 73.3 | 97.3 | 14.5 |

| **Number** | **Accession** | **Origin** | **Latitude (°N)** | **Longitude (°W)** | **2016 Shenyang** | | | | | | | | | | | | | |
| --- | --- | --- | --- | --- | --- | --- | --- | --- | --- | --- | --- | --- | --- | --- | --- | --- | --- | --- |
|  |  |  |  |  | **PH**  **(cm)** | **NMN** | **EBN** | **EPN** | **GN** | **GW**  **(g)** | **HGW**  **(g)** | **R1**  **(day)** | **R2**  **(day)** | **R3**  **(day)** | **R4**  **(day)** | **R5**  **(day)** | **R6**  **(day)** | **R7**  **(day)** |
| G1 | Tiedou 50 | Liaoning,China | 40.8 | 122 | 114 | 23 | 1.9 | 89.7 | 186.1 | 37.9 | 20.4 | 40.4 | 54.4 | 61.4 | 65.4 | 73.4 | 84.4 | 116.4 |
| G2 | Datun xiaoheidou | Hebei,China | 39.2 | 116.3 | 128.9 | 20.2 | 4.6 | 90 | 140.4 | 13.3 | 12.9 | 54.4 | 61.6 | 71.4 | 75.4 | 83.6 | 97.1 | 127.3 |
| G3 | Ken 04-8579 | Heilongjiang,China | 48.3 | 128.1 | 63.3 | 15 | 2.1 | 55.1 | 133.6 | 23.7 | 17.8 | 27.6 | 36.7 | 45.7 | 49.4 | 56.6 | 68.8 | 96.1 |
| G4 | Kangxian2 | Heilongjiang,China | 48.3 | 128.1 | 102.8 | 20.6 | 0.7 | 64.1 | 140 | 25 | 17.9 | 29 | 38 | 48.1 | 54.2 | 62.4 | 78 | 97.8 |
| G5 | Liushitianhuancang | Liaoning,China | 40.8 | 122 | 114.3 | 21.5 | 1.8 | 74.1 | 149.1 | 23.7 | 16.3 | 41.5 | 52.5 | 63.5 | 68.5 | 73.5 | 85.5 | 111.5 |
| G6 | Tiedou 58 | Liaoning,China | 40.8 | 122 | 71.9 | 15.6 | 4 | 52.1 | 99.1 | 23.5 | 22.4 | 52.4 | 60.4 | 68.4 | 71.4 | 78.4 | 91.4 | 115.4 |
| G7 | Zhonghuang 20 | Beijing,China | 40.1 | 116.3 | 111.1 | 19.4 | 2.9 | 82 | 161.5 | 28.5 | 17.6 | 35.5 | 42.5 | 65.5 | 74.5 | 85.5 | 94.5 | 116.5 |
| G8 | Jichanghuangdou 1 | Xinjiang,China | 41.2 | 81.4 | 121.2 | 20.1 | 3.9 | 62.8 | 118.4 | 21.9 | 18.5 | 41.5 | 50.5 | 62.5 | 66.5 | 74.5 | 86.5 | 117.5 |
| G9 | Longquandadou | Heilongjiang,China | 48.3 | 128.1 | 63.4 | 11.7 | 2.4 | 34.6 | 72.9 | 28.3 | 30.8 | 32.5 | 38.5 | 44.5 | 48.5 | 55.5 | 69.5 | 105.5 |
| G10 | Xiaobaiqi | Liaoning,China | 40.8 | 122 | 110.9 | 21.6 | 5.7 | 97.6 | 194.7 | 9 | 15.6 | 42.5 | 54.5 | 62.5 | 70.5 | 78.5 | 91.5 | 117.5 |
| G11 | Gongye 04L-141 | Jilin,China | 43.4 | 126.3 | 109.6 | 23.8 | 3.3 | 114.6 | 228.1 | 47.4 | 16.5 | 38.6 | 44.6 | 52.6 | 59.6 | 76.6 | 85.6 | 103.6 |
| G12 | Tiedou 52 | Liaoning,China | 40.8 | 122 | 86.8 | 16.9 | 1 | 47 | 120.1 | 26.2 | 21.7 | 40.4 | 49.4 | 58.4 | 62.4 | 70.4 | 87.4 | 117.4 |
| G13 | L-57 | America | 43.4 | 79.3 | 121.2 | 23.9 | 6.2 | 117 | 210.9 | 44.4 | 20.7 | 39.4 | 43.4 | 56.4 | 61.4 | 68.4 | 80.4 | 114.4 |
| G14 | He 05-31 | Heilongjiang,China | 48.3 | 128.1 | 75.3 | 16 | 1.4 | 48.4 | 98.7 | 27.5 | 27.8 | 37.4 | 42.4 | 50.4 | 55.4 | 63.4 | 77.4 | 101.4 |
| G15 | L-21 | Canada | 43.4 | 79.3 | 81.6 | 19.2 | 1.2 | 52 | 106.4 | 27.2 | 25.2 | 25 | 32 | 40 | 44.2 | 54.5 | 67 | 86.5 |
| G16 | Hongfeng11 | Heilongjiang,China | 48.3 | 128.1 | 60.3 | 15.8 | 0 | 42.6 | 75.3 | 15.1 | 20 | 32.6 | 38.6 | 48.6 | 52 | 58.6 | 67.6 | 85.6 |
| G17 | Heinong 33 | Heilongjiang,China | 48.3 | 128.1 | 91.4 | 22.1 | 0.4 | 54.2 | 137.6 | 27.7 | 20.3 | 27.3 | 34.3 | 42.3 | 49.3 | 58.3 | 71.3 | 99.3 |
| G18 | Liao 98072 | Liaoning,China | 40.8 | 122 | 94.6 | 21.1 | 1.8 | 60.3 | 118.8 | 24.4 | 20.7 | 42.5 | 47.5 | 57.5 | 65.5 | 73.5 | 88.5 | 128 |
| G19 | Kenfeng 22 | Heilongjiang,China | 48.3 | 128.1 | 111.1 | 21.8 | 1.9 | 121 | 177.1 | 35.4 | 20.1 | 28.4 | 33.1 | 41.4 | 46 | 54.6 | 71.6 | 108.6 |
| G20 | Jiyu 89 | Jilin,China | 43.4 | 126.3 | 82.7 | 19.8 | 1.9 | 70.5 | 146.8 | 28.2 | 19.1 | 30.3 | 37.3 | 52.3 | 57.3 | 67.3 | 77.3 | 105.3 |
| G21 | Tiedou 51 | Liaoning,China | 40.8 | 122 | 82.3 | 16.9 | 1.4 | 47.2 | 93.5 | 19.7 | 20.9 | 35.4 | 41.4 | 52.4 | 59.4 | 71.4 | 87.4 | 114.4 |
| G22 | Tongnong 13 | Jilin,China | 43.4 | 126.3 | 100.1 | 16.7 | 0.8 | 63.4 | 129.2 | 39 | 29.1 | 31.6 | 39.4 | 50.2 | 53.6 | 60.8 | 82.8 | 100.6 |
| G23 | Bei 1873 | Heilongjiang,China | 48.3 | 128.1 | 64.5 | 15.6 | 1.3 | 59.4 | 137.4 | 31 | 22.6 | 24.3 | 33.3 | 38.3 | 41.3 | 49.3 | 71.3 | 88.3 |
| G24 | Hefeng 50 | Heilongjiang,China | 48.3 | 128.1 | 67.9 | 15.5 | 0.1 | 45.2 | 113 | 21.6 | 19.1 | 27 | 35.4 | 44.4 | 48.8 | 56.9 | 69.8 | 86.2 |
| G25 | Sui 02-339 | Heilongjiang,China | 48.3 | 128.1 | 79.8 | 19.7 | 2.3 | 39 | 78.6 | 18.2 | 23.5 | 31.2 | 38 | 48.8 | 53.4 | 64.4 | 75.4 | 93.7 |
| G26 | Hujiao 03-286 | Inner Mongolia,China | 44.5 | 111.7 | 56 | 11.3 | 0.1 | 30.2 | 69.3 | 14.8 | 21.5 | 27.4 | 33.4 | 39.4 | 43.4 | 49.4 | 61.4 | 86.4 |
| G27 | Heinong 44 | Heilongjiang,China | 48.3 | 128.1 | 65.5 | 14.1 | 0 | 57.7 | 116.7 | 23.9 | 20.5 | 30.7 | 37.8 | 44.2 | 49 | 55.6 | 67.8 | 93.1 |
| G28 | Hujiao 04-528 | Inner Mongolia,China | 44.5 | 111.7 | 49.2 | 11.2 | 0.1 | 36.1 | 84.7 | 18.2 | 21.5 | 25 | 32.1 | 37.8 | 41.1 | 49.6 | 61.4 | 83.8 |
| G29 | Fengshou 10 | Heilongjiang,China | 48.3 | 128.1 | 83 | 15.6 | 0.9 | 37.3 | 68 | 16.5 | 23.2 | 32.5 | 42.5 | 52.5 | 55.5 | 61.5 | 73.5 | 93.5 |
| G30 | Hersong 2 | Ukraine | 50.3 | 30.3 | 102.8 | 19.3 | 2.6 | 72.6 | 128.3 | 27 | 21 | 40.3 | 50.3 | 60.3 | 63.3 | 72.3 | 84.3 | 108.3 |
| G31 | Jinzhou 4-1 | Liaoning,China | 40.8 | 122 | 64.1 | 14.6 | 1.7 | 56.9 | 108.3 | 26.2 | 24.2 | 49.4 | 57.4 | 63.4 | 64.4 | 71.4 | 84.4 | 107.4 |
| G32 | Suinong 14 | Heilongjiang,China | 48.3 | 128.1 | 67.7 | 17.4 | 1.1 | 54.4 | 119 | 24.2 | 20.4 | 31.7 | 43.4 | 52.4 | 55.6 | 62.4 | 70.4 | 95.7 |
| G33 | Tiejiasilihuang | Jilin,China | 43.4 | 126.3 | 107 | 22.9 | 1.7 | 84.5 | 204.1 | 40.8 | 19.3 | 36.2 | 43.6 | 53 | 57.8 | 70.8 | 84.4 | 104.6 |
| G34 | Tiefeng 8 | Liaoning,China | 40.8 | 122 | 77.6 | 16.9 | 6.1 | 98.2 | 161.8 | 35.3 | 21.9 | 61.4 | 70.6 | 76.8 | 82.6 | 86.4 | 96.6 | 122.8 |
| G35 | Silihuang | Jilin,China | 43.4 | 126.3 | 92.1 | 20.6 | 2.7 | 72.6 | 155.4 | 32 | 20.6 | 36.6 | 46 | 53.4 | 56.6 | 64.6 | 77.7 | 100.6 |
| G36 | Bei 1361 | Heilongjiang,China | 48.3 | 128.1 | 81.1 | 20.1 | 0.4 | 51.3 | 110.3 | 24.3 | 22 | 25 | 33.8 | 45.6 | 50.6 | 61.2 | 73.8 | 92 |
| G37 | L-5 | Canada | 43.4 | 79.3 | 75.3 | 20.6 | 2.7 | 61.7 | 125.2 | 25.2 | 20.2 | 38.7 | 45.5 | 53.5 | 67.5 | 77.9 | 92.7 | 118.5 |
| G38 | Maoyandou | Gansu, China | 32.3 | 92.1 | 103.1 | 17.4 | 2.8 | 48.5 | 82.2 | 19.2 | 23.4 | 33.5 | 44.5 | 56.5 | 62.5 | 69.5 | 85.5 | 113.5 |
| G39 | Fengdihuang | Jilin,China | 43.4 | 126.3 | 115.2 | 15 | 9.8 | 101.4 | 170.8 | 35.2 | 20.8 | 44.5 | 52.5 | 61.5 | 66.5 | 74.5 | 88.5 | 119.5 |
| G40 | Tiedou 54 | Liaoning,China | 40.8 | 122 | 70.7 | 17 | 1.6 | 53.3 | 122.8 | 20.5 | 16.7 | 49.4 | 56.4 | 63.4 | 67.4 | 73.4 | 88.4 | 111.4 |
| G41 | Jiunong 20 | Jilin,China | 43.4 | 126.3 | 64.7 | 17.1 | 0.1 | 39.9 | 88.5 | 20.3 | 23 | 32.6 | 41.4 | 53.8 | 58.3 | 67.3 | 83.3 | 113.1 |
| G42 | Duludou | Jilin,China | 43.4 | 126.3 | 87.7 | 17.7 | 1.8 | 46 | 102.3 | 21.5 | 20.8 | 31.6 | 41.6 | 48.6 | 52.6 | 60.6 | 74.6 | 93.6 |
| G43 | Sui 03-3046 | Heilongjiang,China | 48.3 | 128.1 | 59.8 | 12.4 | 0 | 46.1 | 77.5 | 13.7 | 17.7 | 27 | 34.4 | 42.6 | 48.6 | 56.6 | 69.6 | 86.4 |
| G44 | Hefeng 47 | Heilongjiang,China | 48.3 | 128.1 | 85.6 | 17.4 | 0.8 | 83.4 | 179 | 37.2 | 20.8 | 42.6 | 47.6 | 56.8 | 59.4 | 67.6 | 79.5 | 99.6 |
| G45 | V111-4 | Jilin,China | 43.4 | 126.3 | 72 | 16.1 | 0.3 | 43.1 | 102.8 | 21.1 | 20.5 | 38.4 | 44.4 | 50.4 | 56.4 | 64.4 | 78.4 | 102.4 |
| G46 | Longxuan 1 | Heilongjiang,China | 48.3 | 128.1 | 71.4 | 16.1 | 0.2 | 40.7 | 95.5 | 18.7 | 19.5 | 24.5 | 32.5 | 45.5 | 49.5 | 60.5 | 72.5 | 99.5 |
| G47 | Dongnong 8004 | Heilongjiang,China | 48.3 | 128.1 | 71.7 | 8.2 | 7.6 | 76.9 | 163.3 | 12.6 | 18.8 | 29.2 | 36.4 | 42.4 | 48 | 56.4 | 69.8 | 97.7 |
| G48 | Sui 03-3952 | Heilongjiang,China | 48.3 | 128.1 | 52.5 | 12.9 | 0.3 | 69.6 | 164.1 | 24.8 | 19.6 | 36.5 | 43.6 | 53.7 | 57.5 | 63.5 | 73.4 | 107 |
| G49 | Hefeng 25 | Heilongjiang,China | 48.3 | 128.1 | 66.6 | 15.8 | 3.2 | 94.9 | 154.7 | 31.5 | 20.3 | 35.5 | 43.5 | 53 | 56.5 | 69 | 82.3 | 97.5 |
| G50 | Liaodou 3 | Liaoning,China | 40.8 | 122 | 106.4 | 20.8 | 1.3 | 56.8 | 113.1 | 23.1 | 20.3 | 37.5 | 46.5 | 56.5 | 61.5 | 71.5 | 84.5 | 126.5 |
| G51 | Hujiao 423 | Inner Mongolia,China | 44.5 | 111.7 | 64.3 | 15.2 | 0.9 | 36.5 | 86.9 | 17.4 | 20.1 | 38.5 | 43.5 | 46.5 | 49.5 | 56.5 | 64.5 | 86.5 |
| G52 | Liaoxian 1 | Liaoning,China | 40.8 | 122 | 21.2 | 7 | 5.9 | 89.4 | 140.7 | 45.1 | 30.1 | 28.3 | 36.3 | 46.3 | 49.3 | 54.3 | 70.3 | 105.3 |
| G53 | Zhongpin 95-5388 | Beijing,China | 40.1 | 116.3 | 109.4 | 26.6 | 3.3 | 85 | 168.5 | 32.9 | 19.4 | 47.3 | 60.3 | 70.3 | 76.3 | 86.3 | 101.3 | 126 |
| G54 | Heihe 45 | Heilongjiang,China | 48.3 | 128.1 | 109.9 | 17.6 | 2.3 | 85.9 | 194.9 | 37.6 | 20.2 | 25 | 32.1 | 38.2 | 41.8 | 54.6 | 71.7 | 118.2 |
| G55 | L-59 Peking | America | 41.8 | 92.9 | 121.4 | 23.7 | 8.3 | 125.7 | 231.5 | 41.9 | 14.8 | 64.3 | 70.5 | 78.3 | 81.3 | 88.6 | 99.6 | 115.3 |
| G56 | Sui 04-5804 | Heilongjiang,China | 48.3 | 128.1 | 63.6 | 14.4 | 0 | 40.2 | 90.3 | 18 | 20 | 28 | 37.6 | 46 | 49.4 | 57 | 69.4 | 85.7 |
| G57 | Ji 06B7 | Hebei,China | 39.2 | 116.3 | 111.6 | 28.9 | 2.5 | 57 | 114.5 | 27 | 23.3 | 57 | 69.4 | 80.6 | 86.6 | 96.2 | 109.8 | 128 |
| G58 | Marca. Joe lisa | Heilongjiang,China | 48.3 | 128.1 | 102.9 | 25.7 | 6.3 | 126.5 | 230.4 | 38.2 | 13.2 | 47.4 | 55.4 | 61.4 | 68.4 | 76.4 | 89.4 | 113.4 |
| G59 | Jihuang 13 | Hebei,China | 39.2 | 116.3 | 113.2 | 25.5 | 1.3 | 84.3 | 184.2 | 40 | 21.8 | 41.3 | 56.3 | 67.3 | 72.3 | 81.3 | 96.3 | 124.3 |
| G60 | L-28 | Canada | 43.4 | 79.3 | 66 | 15.4 | 3.5 | 78.2 | 127.9 | 22.4 | 17.6 | 26 | 36 | 40 | 44.3 | 52.6 | 64.5 | 93.5 |
| G61 | Beifeng 9 | Heilongjiang,China | 48.3 | 128.1 | 62.3 | 12.8 | 0 | 44.4 | 107.5 | 19.5 | 18.2 | 27 | 35.8 | 45.4 | 47.4 | 55.8 | 68.4 | 87.4 |
| G62 | Zhongzuo 00-683 | Beijing,China | 40.1 | 116.3 | 101.6 | 22 | 2 | 80.6 | 157.8 | 30.3 | 18.7 | 48.3 | 62.3 | 73.3 | 78.3 | 90.3 | 101.3 | 123.3 |
| G63 | Dongnong 50 | Heilongjiang,China | 48.3 | 128.1 | 68.1 | 16.4 | 4.1 | 90.7 | 218.6 | 34.6 | 15.6 | 29.4 | 36.1 | 49 | 53.4 | 57.8 | 75.4 | 95.6 |
| G64 | Dongnong 93-046 | Heilongjiang,China | 48.3 | 128.1 | 101.3 | 20.4 | 1.2 | 63.1 | 136.7 | 29 | 21 | 33 | 40.7 | 50 | 55 | 63 | 75.4 | 104.7 |
| G65 | Suinong 10 | Heilongjiang,China | 48.3 | 128.1 | 69.5 | 17.7 | 0.2 | 43 | 84 | 18.4 | 21.9 | 30.6 | 37.1 | 44.6 | 49.6 | 59 | 72.7 | 91 |
| G66 | Tiefeng 31 | Liaoning,China | 40.8 | 122 | 83.8 | 21.8 | 1.9 | 84.9 | 165.1 | 34.4 | 20.8 | 40.5 | 46.5 | 62.5 | 69.5 | 75.5 | 87.5 | 126.5 |
| G67 | Dongnong 49 | Heilongjiang,China | 48.3 | 128.1 | 54 | 12.2 | 0.1 | 41.4 | 81.1 | 15.9 | 19.6 | 25 | 34.6 | 41.6 | 45.6 | 52.4 | 63.8 | 82.8 |
| G68 | Huayou 446 | Hebei,China | 39.2 | 116.3 | 116.9 | 27 | 0.8 | 62.1 | 164.3 | 39 | 23.8 | 45.3 | 56.3 | 68.3 | 72.3 | 81.3 | 94.3 | 124.3 |
| G69 | Heihe 48 | Heilongjiang,China | 48.3 | 128.1 | 54.1 | 12.6 | 0 | 39.9 | 74.5 | 12.8 | 17.2 | 27 | 32.6 | 44 | 49.7 | 54.3 | 64.3 | 85.5 |
| G70 | Huajiang 2 | Heilongjiang,China | 48.3 | 128.1 | 53.2 | 13.1 | 0.7 | 37.8 | 73.4 | 14.7 | 20.1 | 25 | 31.6 | 37.2 | 40.6 | 48 | 62.4 | 85.2 |
| G71 | Suinong 29 | Heilongjiang,China | 48.3 | 128.1 | 78.2 | 18 | 0.7 | 45.3 | 110.2 | 23.7 | 21.5 | 28.4 | 36.4 | 48.4 | 51.6 | 59.6 | 76.4 | 100.6 |
| G72 | Hefeng 35 | Heilongjiang,China | 48.3 | 128.1 | 56.8 | 15 | 0.4 | 50.4 | 109.4 | 22.7 | 20.7 | 30.3 | 37.3 | 44.3 | 48.3 | 55.3 | 71.3 | 98.3 |
| G73 | Suinong 8 | Heilongjiang,China | 48.3 | 128.1 | 81.9 | 18.9 | 1.2 | 69.5 | 123.8 | 29.1 | 23.4 | 34.4 | 41.6 | 52.2 | 56.2 | 64.2 | 72.2 | 94.8 |
| G74 | Jilin 47 | Jilin,China | 43.4 | 126.3 | 74.4 | 17.7 | 1 | 62.4 | 123.1 | 32.3 | 28.3 | 35.5 | 43.5 | 49.5 | 53.5 | 63.5 | 77.5 | 104.5 |
| G75 | Heinong 48 | Heilongjiang,China | 48.3 | 128.1 | 69.2 | 16.6 | 0.7 | 57.9 | 118 | 20.6 | 17.6 | 36.4 | 40.4 | 47.4 | 53.4 | 62.4 | 75.4 | 98.4 |
| G76 | Nova | Italy | 41.5 | 12.3 | 72.7 | 17.8 | 1.8 | 80.7 | 150.8 | 41.8 | 26.3 | 29.3 | 39.3 | 53.3 | 58.3 | 68.3 | 82.3 | 109.3 |
| G77 | Beidou 14 | Heilongjiang,China | 48.3 | 128.1 | 65.6 | 17.4 | 0.6 | 57.4 | 107.3 | 21.3 | 19.1 | 25 | 32.6 | 38.6 | 42.4 | 49.6 | 72.4 | 103.6 |
| G78 | Kangxian 3 | Heilongjiang,China | 48.3 | 128.1 | 106 | 20.9 | 2.9 | 95.9 | 188.9 | 36.9 | 19.2 | 35.6 | 43.6 | 51.6 | 54.8 | 67.1 | 83.4 | 104.6 |
| G79 | Dongnong 42 | Heilongjiang,China | 48.3 | 128.1 | 102 | 20 | 1.2 | 50 | 109.4 | 26.8 | 24.5 | 29.4 | 35.4 | 48.4 | 52.4 | 61.4 | 72.4 | 97.4 |
| G80 | Kenjian 23 | Heilongjiang,China | 48.3 | 128.1 | 56.8 | 14.3 | 0.2 | 40.9 | 91.3 | 15.9 | 17.5 | 27 | 36.6 | 44.4 | 49 | 56.5 | 70.6 | 92.4 |
| G81 | Heinong 55 | Heilongjiang,China | 48.3 | 128.1 | 57.1 | 13.9 | 0 | 37.9 | 75.8 | 16.4 | 21.3 | 38.3 | 42.3 | 48.3 | 53 | 60.3 | 73.3 | 106.3 |
| G82 | Hefeng 45 | Heilongjiang,China | 48.3 | 128.1 | 80.5 | 21.9 | 5.2 | 113.6 | 226 | 49.3 | 24.3 | 28.3 | 38 | 50.6 | 54.3 | 65.5 | 86 | 105.5 |
| G83 | Kenfeng 15 | Heilongjiang,China | 48.3 | 128.1 | 51.9 | 12.8 | 0.5 | 53.9 | 127.1 | 24.9 | 18.6 | 25 | 36.6 | 45.8 | 48.8 | 57.4 | 68.6 | 88.4 |
| G84 | Chasedou | Jilin,China | 43.4 | 126.3 | 101.4 | 22.2 | 1.2 | 62 | 118.3 | 20.1 | 18 | 30.5 | 38.5 | 54.5 | 58.5 | 68.5 | 80.5 | 102.5 |
| G85 | He 05-991 | Heilongjiang,China | 48.3 | 128.1 | 101.2 | 16.7 | 0.5 | 71.3 | 124.9 | 36 | 23 | 30.6 | 38 | 52.1 | 55.6 | 67 | 85 | 107.8 |
| G86 | Xiaolimoshidou | Heilongjiang,China | 39.2 | 116.3 | 120.1 | 21 | 5.3 | 127.7 | 222 | 14.9 | 5.7 | 36.4 | 41.4 | 55.4 | 59.4 | 68.4 | 80.4 | 105.4 |
| G87 | Jingshanpu | Heilongjiang,China | 48.3 | 128.1 | 68.6 | 16.9 | 1.4 | 38.6 | 77.4 | 11 | 14.1 | 37.6 | 44.6 | 54.6 | 57.6 | 70.6 | 79.6 | 93.6 |
| G88 | Qinganheidou | Heilongjiang,China | 48.3 | 128.1 | 128.9 | 19.3 | 4.6 | 93.7 | 180 | 26.1 | 14.8 | 36.4 | 48.4 | 59.4 | 64.4 | 73.4 | 82.4 | 103.4 |
| G89 | Dongnong594 | Heilongjiang,China | 48.3 | 128.1 | 63.4 | 16.7 | 0.2 | 49.7 | 108.8 | 21.1 | 19.8 | 36.5 | 41.5 | 52.5 | 57.5 | 65.5 | 76.5 | 102.5 |
| G90 | Yapoche | Heilongjiang,China | 48.3 | 128.1 | 90 | 1.3 | 2 | 60.5 | 110.9 | 20.4 | 21.1 | 31.1 | 40.4 | 47.6 | 56.4 | 70.4 | 80.7 | 105.1 |
| G91 | Kennong 29 | Heilongjiang,China | 48.3 | 128.1 | 56.6 | 13 | 6.9 | 42.4 | 77.1 | 15.2 | 19.8 | 30.4 | 41.4 | 47.8 | 51.6 | 58.5 | 69.5 | 84.5 |
| G92 | Heimoshidou | Jilin,China | 43.4 | 126.3 | 129.8 | 23.5 | 5.4 | 111.8 | 186.1 | 27.8 | 14.7 | 46.4 | 53.4 | 63.4 | 69.4 | 76.4 | 89.4 | 112.4 |
| G93 | Ha 04-1824 | Heilongjiang,China | 48.3 | 128.1 | 64 | 12.9 | 0.1 | 43.2 | 101.4 | 17.6 | 17.4 | 27.4 | 35.8 | 46.4 | 50.3 | 57.3 | 72.2 | 102.4 |
| G94 | Fangzhengmoshidou | Heilongjiang,China | 48.3 | 128.1 | 118.8 | 22.1 | 3.2 | 50.4 | 77.8 | 10.1 | 12.9 | 41.4 | 47.4 | 63.4 | 69.4 | 76.4 | 87.4 | 117.4 |
| G95 | Helongyoutai | Jilin,China | 43.4 | 126.3 | 83 | 12.9 | 3.9 | 43.5 | 90.5 | 20.4 | 22.6 | 33.4 | 38.4 | 45.4 | 51.2 | 56.2 | 68.6 | 89.4 |
| G96 | Kexin 3 | Beijing,China | 40.1 | 116.3 | 101 | 24.7 | 1.7 | 57.8 | 111.9 | 27.3 | 24.3 | 52 | 60.7 | 70 | 76.7 | 84.1 | 95.7 | 125.4 |
| G97 | Yuanbaojin | Heilongjiang,China | 48.3 | 128.1 | 81.6 | 14.7 | 4.2 | 66.1 | 127.8 | 27.4 | 21 | 28.5 | 37.5 | 46.5 | 50.5 | 56.5 | 73.5 | 102.5 |
| G98 | Zhongzuo J4133 | Beijing,China | 40.1 | 116.3 | 93.9 | 21.6 | 2.3 | 112.4 | 204 | 39 | 18.1 | 39.4 | 55.4 | 64.4 | 67.4 | 75.4 | 91.4 | 120.4 |
| G99 | Jilin 30 | Jilin,China | 43.4 | 126.3 | 87.8 | 18.9 | 0.3 | 52.2 | 112.8 | 21.3 | 18.9 | 41.4 | 50.4 | 59.4 | 63.4 | 71.4 | 89.4 | 115.4 |
| G100 | Ji 100 | Jilin,China | 43.4 | 126.3 | 85 | 16 | 0.5 | 64.5 | 131.3 | 26.9 | 20.6 | 36.4 | 42.4 | 53.4 | 57.4 | 65.4 | 76.4 | 102.4 |
| G101 | Suinong 30 | Heilongjiang,China | 48.3 | 128.1 | 63.2 | 13.2 | 0 | 36.1 | 88.7 | 19.2 | 21.6 | 32.4 | 37.8 | 44.8 | 48.1 | 55.4 | 68.4 | 90.1 |
| G102 | Boige du | Germany | 52.3 | 13.2 | 77.8 | 12.9 | 5.1 | 85.7 | 76.3 | 12.1 | 15.5 | 35.8 | 44.6 | 53.6 | 58.8 | 66.6 | 87 | 113.4 |
| G103 | Beidou 16 | Heilongjiang,China | 48.3 | 128.1 | 124.8 | 23.6 | 3.3 | 99 | 199.5 | 39.3 | 19.9 | 41.4 | 49.4 | 53.4 | 58.4 | 67.4 | 85.4 | 113.4 |
| G104 | Kebei 1 | Heilongjiang,China | 48.3 | 128.1 | 74 | 17.9 | 1.6 | 74.6 | 166.1 | 34 | 20.5 | 31.6 | 37.6 | 45.6 | 50 | 59 | 69.1 | 87.3 |
| G105 | Mengdou 21 | Inner Mongolia,China | 44.5 | 111.7 | 56 | 14.2 | 0.5 | 49.8 | 98.6 | 21.6 | 22.2 | 37.4 | 44.4 | 51.4 | 54.4 | 61.4 | 75.4 | 100.4 |
| G106 | Heihe 38 | Heilongjiang,China | 48.3 | 128.1 | 58.8 | 13.9 | 0.2 | 47.7 | 98.7 | 40.1 | 32.1 | 23 | 28.6 | 41.1 | 43.6 | 52 | 60.4 | 82.8 |
| G107 | Zhongdou 35 | Beijing,China | 40.1 | 116.3 | 109.5 | 23.7 | 2 | 54.1 | 116.3 | 26.8 | 23 | 38.5 | 46.5 | 52.5 | 59.5 | 66.5 | 83.5 | 121.5 |
| G108 | Zhonghuang 6 | Beijing,China | 40.1 | 116.3 | 101.9 | 24 | 3.1 | 71.9 | 144.1 | 27.3 | 19 | 49.4 | 63.4 | 71.4 | 74.4 | 80.4 | 91.4 | 113.4 |
| G109 | Jiyu 94 | Jilin,China | 43.4 | 126.3 | 71.1 | 14.6 | 3.7 | 84.6 | 138.9 | 27.9 | 20.3 | 51.4 | 60.4 | 70.4 | 74.4 | 78.4 | 91.4 | 112.4 |
| G110 | Dongnong 44 | Heilongjiang,China | 48.3 | 128.1 | 53.3 | 15 | 0.6 | 40.3 | 79.8 | 14.4 | 18 | 24.3 | 32.3 | 37.3 | 40.3 | 47.3 | 68.3 | 99.3 |
| G111 | Liaonong 2 | Liaoning,China | 40.8 | 122 | 54.4 | 13.1 | 5.2 | 124.1 | 226.7 | 27.7 | 14.8 | 39.5 | 48.5 | 58.5 | 62.5 | 69.5 | 81.5 | 98.5 |
| G112 | Bei 1484 | America | 48.3 | 128.1 | 71.4 | 16.1 | 0.7 | 52.4 | 101.4 | 19.7 | 19.5 | 27 | 32.1 | 40.4 | 44.4 | 51.4 | 61.4 | 87.2 |
| G113 | Williams82 | America | 41.8 | 92.9 | 91.7 | 20.6 | 2.8 | 99.7 | 163.8 | 33.4 | 20.3 | 41.2 | 48.2 | 60.2 | 65.2 | 73.2 | 85.2 | 116.2 |
| G114 | Jindou 33 | Liaoning,China | 40.8 | 122 | 107.7 | 17.7 | 5.9 | 61 | 96.9 | 22.4 | 23.9 | 54.5 | 64.5 | 74.8 | 80.5 | 87.5 | 100 | 122.3 |
| G115 | Kenfeng 18 | Heilongjiang,China | 48.3 | 128.1 | 118.7 | 21.6 | 1.2 | 58.3 | 120.3 | 26.8 | 21.4 | 29.6 | 42 | 49 | 53.6 | 61 | 81.6 | 103.4 |
| G116 | Suinong 20 | Heilongjiang,China | 48.3 | 128.1 | 66.1 | 17.5 | 0.4 | 84.6 | 196 | 18.2 | 20.9 | 30.3 | 38.3 | 46.3 | 49.3 | 58.3 | 72.3 | 95.3 |
| G117 | Mengdou 9 | Inner Mongolia,China | 44.5 | 111.7 | 56.2 | 11.3 | 0.1 | 39.1 | 84.4 | 17.9 | 21.2 | 27 | 31.8 | 40.2 | 45.2 | 52.4 | 65.4 | 86.2 |
| G118 | L-9 | America | 41.8 | 92.9 | 109.4 | 29.1 | 3 | 101.7 | 203.7 | 29.4 | 16.5 | 57.6 | 65.3 | 75.4 | 82.3 | 97.8 | 108.3 | 125.8 |
| G119 | Suinong 1 | Heilongjiang,China | 48.3 | 128.1 | 100 | 21 | 4 | 99.3 | 202.3 | 43.7 | 21.7 | 42.4 | 50.8 | 61.8 | 68.4 | 86.8 | 101.5 | 123.2 |
| G120 | Kenfeng 14 | Heilongjiang,China | 48.3 | 128.1 | 74.9 | 19.4 | 0.2 | 44.7 | 108.6 | 22.9 | 21.1 | 34.6 | 39.6 | 45.6 | 51.6 | 59.6 | 75.6 | 102.6 |
| G121 | Tiejiazi | Liaoning,China | 40.8 | 122 | 82.1 | 18.2 | 0.7 | 73.4 | 145 | 34.1 | 23.6 | 39.4 | 47 | 56.4 | 61.4 | 68.4 | 80.4 | 104.4 |
| G122 | Suinong 4 | Heilongjiang,China | 48.3 | 128.1 | 69.6 | 17.9 | 2.6 | 71.1 | 130.5 | 28 | 21.8 | 34.6 | 41.8 | 52 | 54.6 | 60.8 | 70 | 91.9 |
| G123 | Jinshanchamoshidou | Jilin,China | 43.4 | 126.3 | 128.4 | 19 | 8.4 | 76.3 | 156.4 | 20.4 | 12.4 | 53.4 | 65 | 77.7 | 82.8 | 88.6 | 99 | 114.7 |
| G124 | Longpin 03-311 | Heilongjiang,China | 48.3 | 128.1 | 66 | 15.8 | 0.2 | 46.2 | 107.8 | 22.5 | 20.8 | 29 | 37.4 | 45 | 50.2 | 56.6 | 69.6 | 84.1 |
| G125 | Jilinchalihua | Jilin,China | 43.4 | 126.3 | 71.5 | 13.1 | 3.1 | 23 | 35 | 2.6 | 8 | 45.4 | 56.4 | 63.4 | 70.4 | 76.4 | 88.4 | 109.4 |
| G126 | Heinong 37 | Heilongjiang,China | 48.3 | 128.1 | 65.3 | 14.9 | 1 | 91.8 | 197.8 | 41.4 | 20.8 | 32 | 43 | 52 | 54 | 62 | 80 | 102.5 |
| G127 | Huajiang 4403 | Heilongjiang,China | 48.3 | 128.1 | 69.6 | 15.7 | 0.7 | 52 | 95.3 | 21.9 | 23 | 24 | 36.8 | 40.4 | 45.4 | 54.8 | 68.4 | 91.4 |
| G128 | Huangbaozhu | Liaoning,China | 40.8 | 122 | 115.3 | 17.1 | 2.4 | 46 | 95 | 19.5 | 19.6 | 43.5 | 57.5 | 68.5 | 75.5 | 82.5 | 90.5 | 113.5 |
| G129 | Suinong 25 | Heilongjiang,China | 48.3 | 128.1 | 89.9 | 19.6 | 1.8 | 74.3 | 139.8 | 31.4 | 22.4 | 30.8 | 39.8 | 49.8 | 55.4 | 61.4 | 85.8 | 97.6 |
| G130 | Sui 05-7304 | Heilongjiang,China | 48.3 | 128.1 | 56.7 | 14 | 0.2 | 39.8 | 65.1 | 16.8 | 25.7 | 24.8 | 30.8 | 36.4 | 43 | 52 | 67.4 | 90.4 |
| G131 | Jidou 9 | Hebei,China | 39.2 | 116.3 | 111.3 | 27.8 | 1.1 | 72 | 178 | 45.2 | 25.4 | 40.4 | 51.4 | 64.4 | 73.4 | 111.4 | 118.4 | 127 |
| G132 | Mengdou 14 | Inner Mongolia,China | 44.5 | 111.7 | 69.5 | 17.2 | 1.5 | 50.9 | 110.7 | 23.1 | 20.6 | 27 | 37.4 | 48 | 51.6 | 56.8 | 74.4 | 93.6 |
| G133 | Handou 5 | Hebei,China | 39.2 | 116.3 | 111.4 | 24.1 | 1.4 | 68.4 | 164.3 | 42.9 | 26.1 | 36.3 | 51.3 | 60.3 | 65.3 | 73.3 | 87.3 | 122.3 |
| G134 | Jidou 17 | Hebei,China | 39.2 | 116.3 | 115.3 | 24.4 | 4.9 | 84.4 | 158.5 | 30.2 | 19 | 46.3 | 61.3 | 71.3 | 75.3 | 84.3 | 100.3 | 126.3 |
| G135 | Aika 166 | Heilongjiang,China | 44.2 | 26.1 | 71.9 | 17.9 | 2.5 | 55.4 | 105.6 | 20.9 | 20.3 | 38.4 | 45.2 | 53.5 | 55.5 | 59.4 | 71.7 | 94.5 |
| G136 | Sui 04-6018 | Heilongjiang,China | 48.3 | 128.1 | 63.8 | 17.7 | 0 | 47.6 | 96.5 | 20.5 | 21.5 | 29.4 | 34.4 | 44.4 | 49.4 | 56.4 | 69.4 | 99.4 |
| G137 | Dongnong 48 | Heilongjiang,China | 48.3 | 128.1 | 62.2 | 13.6 | 0 | 35.9 | 84 | 20.6 | 24.7 | 34.5 | 40.5 | 47.5 | 51.5 | 58.5 | 71.5 | 97.5 |
| G138 | L-79 | America | 43.4 | 79.3 | 59 | 14.6 | 0.6 | 47.3 | 83.4 | 12.9 | 15.5 | 26.4 | 32.4 | 41.4 | 46.4 | 52.4 | 63.4 | 87.4 |
| G139 | Zhonghuang 35 | Beijing,China | 40.1 | 116.3 | 76.8 | 16.4 | 1.2 | 40 | 69.9 | 17.2 | 24.6 | 29.5 | 36.5 | 43.5 | 49.5 | 55.5 | 66.5 | 85.5 |
| G140 | Kennong 30 | Heilongjiang,China | 48.3 | 128.1 | 67.9 | 14.7 | 0.1 | 45.3 | 91.8 | 18.4 | 20 | 28 | 35.2 | 44.6 | 49.2 | 55.6 | 71.2 | 88 |
| G141 | Zhonghuang 30 | Beijing,China | 40.1 | 116.3 | 68.3 | 18.3 | 0.6 | 43.8 | 91.6 | 18.8 | 21.2 | 37.4 | 50.4 | 60.4 | 68.4 | 76.4 | 89.4 | 115.4 |
| G142 | Baichengmoshidou | Jilin,China | 43.4 | 126.3 | 106.4 | 15.2 | 6.3 | 82.2 | 110.6 | 12.3 | 11.2 | 43.3 | 51.3 | 62.3 | 68.3 | 73.3 | 82.3 | 99.3 |
| G143 | L-10 | America | 43.4 | 79.3 | 110.5 | 20.9 | 3.1 | 85.4 | 183.2 | 35.4 | 19.3 | 41.4 | 52.4 | 62.4 | 72.4 | 81.4 | 94.4 | 117.4 |
| G144 | Zaoshu 18 | Beijing,China | 40.1 | 116.3 | 77.4 | 21.1 | 2.8 | 58.4 | 112.1 | 29.2 | 25.7 | 44.3 | 49.3 | 59.3 | 64.3 | 73.3 | 89.3 | 128.3 |
| G145 | Zhonghuang 10 | Beijing,China | 40.1 | 116.3 | 87 | 16.5 | 3.5 | 89.6 | 176.9 | 30.2 | 17.1 | 50.4 | 58.4 | 69.4 | 73.4 | 80.4 | 89.4 | 113.4 |
| G146 | Dongnong L-13 | Heilongjiang,China | 43.4 | 79.3 | 104.5 | 18 | 0.6 | 51 | 94.1 | 19.4 | 17.8 | 31.3 | 40.3 | 51.3 | 56.3 | 63.3 | 77.3 | 106.3 |
| G147 | Neifeng 15 | Heilongjiang,China | 48.3 | 128.1 | 88.8 | 19.8 | 2 | 51.2 | 87.9 | 19.5 | 21.9 | 33.4 | 40.4 | 47.4 | 54.4 | 64.4 | 76.6 | 92.1 |
| G148 | Dongnong 47 | Heilongjiang,China | 48.3 | 128.1 | 66.2 | 21.6 | 1.1 | 51.6 | 118.6 | 25.1 | 21.3 | 26 | 37.4 | 49 | 53.8 | 58.1 | 69.7 | 91.6 |
| G149 | Hefeng 29 | Heilongjiang,China | 48.3 | 128.1 | 91.4 | 24.8 | 2 | 66.3 | 133.1 | 28.1 | 21.3 | 40.5 | 45.5 | 54.5 | 58.5 | 66.5 | 77.5 | 107.5 |
| G150 | Dongnong 07-909 | Heilongjiang,China | 48.3 | 128.1 | 67.9 | 19.1 | 0.9 | 67.2 | 138.6 | 46.5 | 31.8 | 25 | 32.7 | 42.8 | 47.4 | 53.4 | 83.1 | 93.5 |
| G151 | Hefeng 52 | Heilongjiang,China | 48.3 | 128.1 | 65.2 | 13.9 | 1.2 | 74.6 | 164.9 | 35 | 21.2 | 23 | 36.4 | 47.8 | 53.6 | 59 | 73.8 | 88.6 |
| G152 | Heihe 18 | Heilongjiang,China | 48.3 | 128.1 | 49.9 | 10.5 | 0.3 | 41 | 88.2 | 16.9 | 19.2 | 22 | 29.7 | 37.6 | 41.2 | 47 | 64 | 84.6 |
| G153 | Neifeng 11 | Heilongjiang,China | 48.3 | 128.1 | 45.7 | 19.2 | 0.2 | 57 | 131.6 | 21.6 | 16.3 | 36.3 | 42.3 | 49.3 | 53.3 | 59.3 | 69.3 | 93.3 |
| G154 | Heihexiaohuangdou | Heilongjiang,China | 48.3 | 128.1 | 63 | 15 | 1.6 | 59 | 107.7 | 22.4 | 20.6 | 26 | 31.4 | 36.4 | 41.4 | 53 | 65.8 | 83.6 |
| G155 | Zhongdou 27 | Beijing,China | 40.1 | 116.3 | 96.9 | 24.1 | 3 | 108 | 210.2 | 48.1 | 22.7 | 45.3 | 56.3 | 65.3 | 70.3 | 78.3 | 91.3 | 115.3 |
| G156 | Bei 4834 | Heilongjiang,China | 48.3 | 128.1 | 58.3 | 11.4 | 3.5 | 60.1 | 123.4 | 33.5 | 27 | 26.8 | 35 | 43.4 | 47.8 | 52.4 | 64.2 | 98.6 |
| G157 | Heilongjiang 41 | Heilongjiang,China | 48.3 | 128.1 | 82.5 | 16.5 | 1.7 | 63 | 133.8 | 31.6 | 23.7 | 38.5 | 45.5 | 52.5 | 62.5 | 69.5 | 81.5 | 101.5 |
| G158 | Zhongpin 03-5179 | Beijing,China | 40.1 | 116.3 | 108.3 | 23.4 | 2.2 | 69.6 | 151.8 | 30.1 | 19.8 | 37.4 | 44.4 | 57.4 | 61.4 | 69.4 | 84.4 | 116.4 |
| G159 | Hefeng 37 | Heilongjiang,China | 48.3 | 128.1 | 114.9 | 28 | 3.9 | 84.5 | 173.1 | 40.3 | 23.9 | 55.2 | 61.7 | 71.7 | 78.2 | 88.4 | 101.4 | 126 |
| G160 | Dongnong 46 | Heilongjiang,China | 48.3 | 128.1 | 72 | 18.7 | 1.2 | 48 | 111.3 | 28.4 | 25.6 | 32.6 | 44 | 49.6 | 52.8 | 62.8 | 69.6 | 95.1 |
| G161 | Dongnong 43 | Heilongjiang,China | 48.3 | 128.1 | 68.7 | 17.9 | 3.5 | 43.7 | 78.7 | 16.8 | 21.8 | 30.4 | 37.4 | 46.4 | 51.4 | 61.4 | 68.4 | 87.4 |
| G162 | Dongnong 1068 | Heilongjiang,China | 48.3 | 128.1 | 66.6 | 16.9 | 1.8 | 73.8 | 159.7 | 37.6 | 23.9 | 32 | 38.6 | 46.4 | 50.4 | 59.4 | 71.6 | 94.4 |
| G163 | Dongnong 56 | Heilongjiang,China | 48.3 | 128.1 | 66.9 | 14.9 | 0.8 | 67.1 | 121.5 | 25 | 20.6 | 34.4 | 39.4 | 45.4 | 51.6 | 58.4 | 71.7 | 98.6 |
| G164 | Jiunong 21 | Jilin,China | 43.4 | 126.3 | 93.3 | 21.9 | 1 | 74.9 | 157.8 | 21.1 | 13.5 | 38.4 | 43.4 | 56.4 | 61.4 | 69.4 | 79.4 | 105.4 |
| G165 | Chamoshidou | Jilin,China | 43.4 | 126.3 | 123.8 | 15.7 | 7.3 | 91.2 | 134.1 | 11 | 8.3 | 57.4 | 64.6 | 78.1 | 81.4 | 92.4 | 103.1 | 123.6 |
| G166 | Daliheidou | Shandong,China | 36.2 | 118.3 | 88.1 | 15.8 | 5 | 53.7 | 97.9 | 18.4 | 18.2 | 31.8 | 39 | 47 | 51 | 68 | 86.4 | 117.4 |
| G167 | Zhongpin 03-5373 | Beijing,China | 40.1 | 116.3 | 75.6 | 16.8 | 5.8 | 87.7 | 162.3 | 31.9 | 19.6 | 53.2 | 65.8 | 73.4 | 79.4 | 87.4 | 100.2 | 124.4 |
| G168 | Wuxing 4 | Hebei,China | 39.2 | 116.3 | 75.9 | 14.7 | 9.3 | 46.9 | 129 | 33.4 | 25.9 | 54.4 | 65.2 | 77.8 | 81.6 | 87.5 | 94.6 | 128.1 |
| G169 | Hefeng 55 | Heilongjiang,China | 48.3 | 128.1 | 65.7 | 18.6 | 0.3 | 41.7 | 77.3 | 18.3 | 23.8 | 25 | 33.4 | 42.2 | 45.6 | 53.6 | 67.7 | 91.1 |
| G170 | Suinong 28 | Heilongjiang,China | 48.3 | 128.1 | 64.9 | 15.1 | 0.5 | 46.3 | 88.8 | 18.6 | 20.9 | 32.6 | 42.6 | 48.6 | 53.4 | 59.8 | 70.2 | 105 |
| G171 | Zhongpin 03-5334 | Beijing,China | 40.1 | 116.3 | 63.1 | 15.3 | 4.2 | 62.3 | 104.9 | 20.8 | 20.2 | 54.6 | 63.8 | 72.3 | 78.3 | 89.6 | 100.6 | 124.6 |
| G172 | Fengshou 6 | Heilongjiang,China | 48.3 | 128.1 | 72.1 | 18.2 | 1.3 | 45.9 | 100.3 | 19.5 | 19.1 | 25 | 34.6 | 42 | 49 | 57.4 | 69.6 | 84.4 |
| G173 | Dunajika | Russia | 61.5 | 98 | 102.5 | 16.2 | 2.7 | 58.6 | 108.6 | 14.9 | 13.5 | 28 | 38.4 | 44.6 | 49.6 | 56.1 | 72.7 | 93.6 |

R1: beginning bloom, R2: full bloom, R3: beginning pod, R4: full pod, R5: beginning seed, R6: full seed, R7: beginning maturity, PH: plant height, NMN: number of main stem nodes, EBN: effective branch number per plant, EPN: effective pod number per plant, GN: grain number per plant, GW: grain weight per plant, HGW: hundred-grain weight.

**Supplementary Table 3.** The growth periods of nineteen strand soybeans in maturity groups (MGs) from North America at three locations.

| **Number** | **Accession** | **Origin** | **MGs** | **2015 Harbin** | | | | | | | **2016 Harbin** | | | | | | |
| --- | --- | --- | --- | --- | --- | --- | --- | --- | --- | --- | --- | --- | --- | --- | --- | --- | --- |
|  |  |  |  | **R1**  **(day)** | **R2**  **(day)** | **R3**  **(day)** | **R4**  **(day)** | **R5**  **(day)** | **R6**  **(day)** | **R7**  **(day)** | **R1**  **(day)** | **R2**  **(day)** | **R3**  **(day)** | **R4**  **(day)** | **R5**  **(day)** | **R6**  **(day)** | **R7**  **(day)** |
| G174 | Maple Presto | Canada | MG000 | 27.5 | 38.5 | 44.7 | 55.0 | 61.0 | 67.7 | 94.0 | 36.3 | 41.7 | 45.7 | 55.3 | 57.4 | 77.5 | 92.7 |
| G175 | OAC Vision | Canada | MG000 | 29.0 | 37.7 | 48.3 | 59.0 | 63.6 | 67.9 | 95.9 | 36.6 | 43.2 | 47.5 | 51.3 | 59.3 | 72.2 | 92.7 |
| G176 | Canatto | Canada | MG000 | 28.0 | 38.9 | 47.7 | 56.8 | 61.4 | 67.0 | 95.0 | 36.8 | 42.9 | 48.4 | 56.3 | 65.6 | 81.0 | 95.6 |
| G177 | Maple Ridge | Canada | MG00 | 27.7 | 36.7 | 45.0 | 53.7 | 61.2 | 66.3 | 94.3 | 36.6 | 43.5 | 47.6 | 55.7 | 60.5 | 74.3 | 94.5 |
| G178 | Glacier | America | MG00 | 29.5 | 37.5 | 49.5 | 57.5 | 64.0 | 72.5 | 98.5 | 37.5 | 43.6 | 54.4 | 60 | 67.6 | 77.5 | 99.0 |
| G179 | MN0201 | America | MG00 | 37.2 | 49.8 | 59.8 | 65.6 | 70.6 | 77.6 | 102.6 | 44.9 | 55.3 | 58.4 | 60.5 | 70.7 | 78.8 | 104 |
| G180 | Traill | America | MG0 | 29.6 | 37.0 | 51.5 | 57.4 | 65.5 | 73.6 | 99.6 | 36.8 | 42.6 | 48.2 | 59.1 | 66.3 | 76.2 | 98.1 |
| G181 | MN0901 | America | MG0 | 42.9 | 51.6 | 69.9 | 77.6 | 82.6 | 89.6 | 119.6 | 54.0 | 57.9 | 70.9 | 80.7 | 85.5 | 102.5 | 124.2 |
| G182 | Surge | America | MG0 | 51.4 | 64.0 | 72.6 | 79.0 | 86.6 | 93.6 | 123.6 | 47.9 | 51.0 | 60.5 | 76.7 | 81.2 | 95.0 | 120.1 |
| G183 | NE1900 | America | MGI | 39.6 | 59.3 | 63.9 | 74.8 | 79.8 | 88.3 | 116.8 | 52.9 | 54.9 | 63.5 | 73.7 | 84.0 | 94.3 | 121.6 |
| G184 | Titan | America | MGI | 37.7 | 53.9 | 60.9 | 75.9 | 79.0 | 88.8 | 116.0 | 46.9 | 56.1 | 60.6 | 75.7 | 81.1 | 96.3 | 118.5 |
| G185 | Holt | America | MGII | 40.8 | 53.7 | 64.2 | 72.8 | 80.1 | 86.8 | 118.3 | 51.9 | 53.5 | 62.5 | 72.3 | 83.3 | 94.7 | 120.6 |
| G186 | OAC Talbot | America | MGII | 41.0 | 51.9 | 66.6 | 69.0 | 81.3 | 86.0 | 118.8 | 54.0 | 63.9 | 71.2 | 77.3 | 87.8 | 99.5 | 124.5 |
| G187 | Flint | America | MGII | 42.2 | 54.4 | 67.5 | 75.1 | 81.8 | 88.1 | 119.1 | 54.0 | 58.9 | 71.0 | 80.9 | 88.0 | 103.3 | 126.3 |
| G188 | Athow | America | MGIII | 50.1 | 62.7 | 72.0 | 81.5 | 86.6 | 94.5 | 122.1 | 54.9 | 63.6 | 73.3 | 83.6 | 89.0 | 108.8 | 129.5 |
| G189 | Zane | America | MGIII | 52.3 | 66.3 | 75.7 | 83.2 | 87.5 | 95.4 | 124.1 | 55.0 | 66.2 | 75.6 | 87.4 | 94.8 | 109.1 | 129.0 |
| G190 | NS93-4118 | America | MGIV | 54.1 | 62.3 | 77.1 | 80.6 | 88.4 | 93.6 | NA | 63.0 | 70.8 | 81.0 | 87.7 | 102.8 | 111.3 | NA |
| G191 | Flyer | America | MGIV | 57.2 | 71.5 | 81.5 | 88.5 | 95.5 | 103.5 | NA | 62.8 | 70.4 | 79.4 | 91.5 | 97.6 | 119.6 | NA |
| G192 | TN4-94 | America | MGIV | 57.5 | 68.7 | 82.2 | 89.2 | 96.2 | 104.2 | NA | 70.9 | 78.9 | 90.7 | 97.2 | 108.0 | 124.1 | NA |

| **Number** | **Accession** | **Origin** | **MGs** | **2015 Changchun** | | | | | | | **2016 Changchun** | | | | | | |
| --- | --- | --- | --- | --- | --- | --- | --- | --- | --- | --- | --- | --- | --- | --- | --- | --- | --- |
|  |  |  |  | **R1**  **(day)** | **R2**  **(day)** | **R3**  **(day)** | **R4**  **(day)** | **R5**  **(day)** | **R6**  **(day)** | **R7**  **(day)** | **R1**  **(day)** | **R2**  **(day)** | **R3**  **(day)** | **R4**  **(day)** | **R5**  **(day)** | **R6**  **(day)** | **R7**  **(day)** |
| G174 | Maple Presto | Canada | MG000 | 30.4 | 41.4 | 45.3 | 53.4 | 55.4 | 65.4 | 71.4 | 32 | 42.2 | 46.2 | 55 | 56 | 63.2 | 74.2 |
| G175 | OAC Vision | Canada | MG000 | 30.4 | 41.4 | 45.3 | 53.4 | 56.4 | 66.4 | 71.4 | 33 | 39.5 | 46.4 | 50.2 | 56.2 | 62.2 | 74.2 |
| G176 | Canatto | Canada | MG000 | 32.2 | 41.4 | 47.2 | 54.4 | 56.4 | 65.4 | 71.4 | 33.2 | 42.8 | 48 | 57.9 | 58.2 | 68 | 77 |
| G177 | Maple Ridge | Canada | MG00 | 33.3 | 43.3 | 46.4 | 49.3 | 58.2 | 67.3 | 73.3 | 33.3 | 40 | 48.3 | 51 | 60.1 | 63 | 78 |
| G178 | Glacier | America | MG00 | 32.3 | 43.2 | 47.4 | 55.2 | 57.2 | 67.2 | 73.2 | 34 | 43.3 | 50 | 55 | 62 | 66.3 | 87.3 |
| G179 | MN0201 | America | MG00 | 33.4 | 42.4 | 49.4 | 54.4 | 60.4 | 67.4 | 73.4 | 35.3 | 42 | 54 | 56 | 63 | 67 | 96 |
| G180 | Traill | America | MG0 | 33.2 | 41.2 | 48.4 | 52.2 | 57.3 | 66.2 | 73.2 | 35.2 | 40.3 | 50.3 | 54.3 | 62.3 | 67.3 | 91.3 |
| G181 | MN0901 | America | MG0 | 33.4 | 46.7 | 49.4 | 70.7 | 79.3 | 93.7 | 108 | 44 | 46 | 59.3 | 69 | 79.3 | 92 | 123 |
| G182 | Surge | America | MG0 | 33.7 | 45.5 | 50.2 | 63.5 | 70.3 | 91.5 | 108 | 39 | 45.2 | 58.2 | 69.2 | 76.2 | 87.2 | 115.2 |
| G183 | NE1900 | America | MGI | 34.3 | 44.3 | 50.2 | 64.3 | 70.3 | 91.3 | 109 | 37.3 | 53.3 | 58 | 65.3 | 72.2 | 86.3 | 110.3 |
| G184 | Titan | America | MGI | 34.4 | 43.4 | 53.3 | 63.4 | 70.4 | 88.4 | 109.1 | 39 | 45 | 58 | 69 | 74 | 87 | 114 |
| G185 | Holt | America | MGII | 34.5 | 43.3 | 56.3 | 64.3 | 71.2 | 87.3 | 119 | 39.2 | 50.3 | 58.2 | 65.2 | 76.3 | 83.2 | 116.2 |
| G186 | OAC Talbot | America | MGII | 35.2 | 41.2 | 57.2 | 59.2 | 71.3 | 86.2 | 119 | 40.3 | 45.3 | 58.3 | 69.3 | 79 | 92.3 | 117.3 |
| G187 | Flint | America | MGII | 35.3 | 47.3 | 58.5 | 67.3 | 71.3 | 93.3 | 119 | 45.2 | 48.3 | 60.3 | 70.3 | 79.3 | 92.3 | 129.3 |
| G188 | Athow | America | MGIII | 36.3 | 44.3 | 59.3 | 66.3 | 72.5 | 92.3 | 120 | 49.3 | 56.4 | 65 | 76.4 | 82 | 101.4 | 129.4 |
| G189 | Zane | America | MGIII | 36.3 | 46.3 | 59.7 | 66.3 | 74.7 | 95.3 | 121 | 50.4 | 61 | 69 | 86 | 91 | 107 | 138 |
| G190 | NS93-4118 | America | MGIV | 37.3 | 48.2 | 60.3 | 73.2 | 83.2 | 97.2 | 115.2 | 50 | 53.7 | 69 | 72 | 87.4 | 91 | 131 |
| G191 | Flyer | America | MGIV | 38.2 | 46.3 | 60.3 | 73.3 | 84.3 | 95.3 | 113.3 | 57.5 | 67.4 | 71.5 | 86 | 95.5 | 119 | 142 |
| G192 | TN4-94 | America | MGIV | 47.3 | 60.3 | 75.3 | 82.3 | 88.3 | 99.3 | 117.3 | 53.4 | 61.5 | 70.4 | 85.5 | 95 | 108.5 | 141 |

| **Number** | **Accession** | **Origin** | **MGs** | **2015 Shenyang** | | | | | | | **2016 Shenyang** | | | | | | |
| --- | --- | --- | --- | --- | --- | --- | --- | --- | --- | --- | --- | --- | --- | --- | --- | --- | --- |
|  |  |  |  | **R1**  **(day)** | **R2**  **(day)** | **R3**  **(day)** | **R4**  **(day)** | **R5**  **(day)** | **R6**  **(day)** | **R7**  **(day)** | **R1**  **(day)** | **R2**  **(day)** | **R3**  **(day)** | **R4**  **(day)** | **R5**  **(day)** | **R6**  **(day)** | **R7**  **(day)** |
| G174 | Maple Presto | Canada | MG000 | 28 | 37.4 | 42.8 | 68.4 | 74.7 | 80.4 | 96.4 | 31.1 | 36.3 | 45.2 | 49.4 | 57.4 | 65.7 | 86.4 |
| G175 | OAC Vision | Canada | MG000 | 28 | 33.5 | 45.4 | 50.8 | 57 | 66.1 | 96.8 | 25.2 | 31.6 | 38.4 | 41.7 | 53 | 63.6 | 85.2 |
| G176 | Canatto | Canada | MG000 | 31.5 | 39.5 | 57.4 | 61.5 | 73.5 | 86.5 | 109.5 | 28 | 37.4 | 42.2 | 49.4 | 55.2 | 66.4 | 85.6 |
| G177 | Maple Ridge | Canada | MG00 | 29.4 | 38.8 | 51.5 | 56.6 | 65.4 | 69.6 | 101.8 | 30.5 | 31.8 | 44.6 | 52.1 | 57 | 65.1 | 86.3 |
| G178 | Glacier | America | MG00 | 29.4 | 41.8 | 48.4 | 60.2 | 63.7 | 71.4 | 101.4 | 28.1 | 33 | 42.2 | 46.2 | 55.5 | 65.2 | 85.8 |
| G179 | MN0201 | America | MG00 | 30.4 | 38.7 | 53 | 54.7 | 66.8 | 72.7 | 104.7 | 25.2 | 29.7 | 38 | 49.8 | 52.2 | 69.7 | 84.6 |
| G180 | Traill | America | MG0 | 30.5 | 47.3 | 54 | 57.8 | 72.6 | 82.8 | 105.8 | 25.3 | 35.2 | 39.7 | 47 | 54.1 | 66.2 | 85.5 |
| G181 | MN0901 | America | MG0 | 31.8 | 41.8 | 58.4 | 66.8 | 74.4 | 85.8 | 111.6 | 34.4 | 40.6 | 61.1 | 68.7 | 78.2 | 88.3 | 116.9 |
| G182 | Surge | America | MG0 | 33.8 | 39.4 | 58.5 | 69.4 | 74.5 | 90.4 | 112.4 | 33 | 41.6 | 57.1 | 66.2 | 75.3 | 90.2 | 112.8 |
| G183 | NE1900 | America | MGI | 37.4 | 45 | 60.6 | 64.4 | 80.4 | 92.6 | 115.8 | 32 | 53.7 | 56.5 | 64 | 75.2 | 82.2 | 112.7 |
| G184 | Titan | America | MGI | 42.2 | 54.5 | 64.5 | 70.5 | 81.7 | 93.5 | 118 | 31.9 | 40.7 | 55.6 | 67.2 | 70.8 | 84 | 109.3 |
| G185 | Holt | America | MGII | 35 | 42.7 | 60.2 | 66.9 | 74.8 | 84.9 | 114.9 | 33.4 | 38.3 | 59.4 | 65.5 | 76.6 | 85.5 | 114.1 |
| G186 | OAC Talbot | America | MGII | 44.5 | 54.2 | 69.1 | 78.7 | 83.2 | 91.7 | 121.7 | 33.1 | 38.4 | 59.3 | 66.4 | 75.5 | 86.8 | 112.9 |
| G187 | Flint | America | MGII | 40.4 | 53.2 | 64.4 | 75.2 | 80.9 | 92.2 | 117.2 | 36.2 | 40.6 | 61.7 | 66.3 | 79.6 | 95.9 | 120.6 |
| G188 | Athow | America | MGIII | 45.1 | 52.2 | 69.6 | 76.1 | 86.1 | 98.1 | 122.4 | 40.6 | 49.1 | 62.3 | 64.9 | 80.4 | 91.1 | 125.4 |
| G189 | Zane | America | MGIII | 47 | 51.8 | 71.2 | 81.8 | 88.4 | 105.8 | 123.3 | 41.6 | 51.6 | 64.6 | 70.9 | 82.5 | 93.1 | 126.1 |
| G190 | NS93-4118 | America | MGIV | 54.6 | 63.6 | 73.6 | 83.6 | 91.8 | 105.6 | 124.5 | 43.4 | 46 | 66 | 72 | 83 | 96.9 | 127.4 |
| G191 | Flyer | America | MGIV | 49.6 | 55 | 73.4 | 80.4 | 88.9 | 107.4 | 124.4 | 44.9 | 54.6 | 68.5 | 74.8 | 84.3 | 100.5 | 127.6 |
| G192 | TN4-94 | America | MGIV | 47.3 | 61.4 | 71.2 | 80.6 | 88.4 | 107.2 | 124 | 49.2 | 56.9 | 69.6 | 73.2 | 85.6 | 102 | 128 |

PH: plant height, NMN: number of nodes of main stem, EBN: number of branch number per plant, EPN: number of pods per plant, GN: grain number per plant, GW: grain yield per plant, HGW: 100-grain weight, R1: beginning bloom, R2: full bloom, R3: beginning pod, R4: full pod, R5: beginning seed, R6: full seed, R7: beginning maturity.

**Supplementary Table 4.** The descriptive statistics of phenotypic values of 173 soybean varieties at three locations in 2015 and 2016.

| **Traits** | **Year** | **Location** | **Mean ± SD** | **Max** | **Min** | **CV(%)** |
| --- | --- | --- | --- | --- | --- | --- |
|  |  |  |  |  |  |  |
| Plant  height (cm) | 2015 | Harbin | 84.4±15.1 | 119.6 | 37.2 | 17.9 |
|  | 2015 | Changchun | 83.8±17.9 | 116.8 | 35.3 | 21.4 |
|  | 2015 | Shenyang | 78.1±15.7 | 112.2 | 25.8 | 20.1 |
|  | 2016 | Harbin | 86.0±22.6 | 144.7 | 37.2 | 21.3 |
|  | 2016 | Changchun | 84.2±18.9 | 132.0 | 33.5 | 30.5 |
|  | 2016 | Shenyang | 83.0±22.3 | 129.8 | 21.2 | 28.5 |
| Number of nodes of main stem | 2015 | Harbin | 16.9±2.3 | 26.1 | 10.9 | 13.4 |
|  | 2015 | Changchun | 16.6±2.1 | 25.7 | 10.7 | 12.7 |
|  | 2015 | Shenyang | 14.9±2.2 | 24.4 | 8.6 | 14.6 |
|  | 2016 | Harbin | 17.8±2.5 | 26.4 | 9.4 | 15.1 |
|  | 2016 | Changchun | 17.5±3.3 | 25.9 | 7.0 | 19.1 |
|  | 2016 | Shenyang | 17.1±4.3 | 25.1 | 6.3 | 23.8 |
| Number of branches per plant | 2015 | Harbin | 2.1±1.8 | 8.4 | 0.0 | 68.1 |
|  | 2015 | Changchun | 2.6±1.7 | 8.2 | 0.0 | 44.2 |
|  | 2015 | Shenyang | 2.0±1.5 | 6.8 | 0.1 | 54.5 |
|  | 2016 | Harbin | 0.8±0.8 | 3.3 | 0.0 | 77.6 |
|  | 2016 | Changchun | 2.0±1.7 | 7.9 | 0.0 | 64.8 |
|  | 2016 | Shenyang | 2.2±2.1 | 9.8 | 0.0 | 75.7 |
| Number of pods per plant | 2015 | Harbin | 46.3±14.6 | 93.9 | 10.7 | 31.6 |
|  | 2015 | Changchun | 56.9±16.2 | 105.5 | 25.2 | 28.5 |
|  | 2015 | Shenyang | 42.6±13.4 | 83.9 | 21.1 | 31.4 |
|  | 2016 | Harbin | 41.0±13.1 | 82.5 | 16.1 | 32.0 |
|  | 2016 | Changchun | 68.3±32.5 | 159.6 | 11.8 | 47.7 |
|  | 2016 | Shenyang | 64.8±22.7 | 127.7 | 23.0 | 35.0 |
| Number of grain per plant | 2015 | Harbin | 90.0±28.7 | 170.7 | 19.3 | 31.9 |
|  | 2015 | Changchun | 120.1±33.0 | 212.9 | 53.8 | 27.5 |
|  | 2015 | Shenyang | 76.5±24.7 | 143.9 | 36.0 | 32.2 |
|  | 2016 | Harbin | 66.9±25.8 | 143.5 | 7.5 | 38.5 |
|  | 2016 | Changchun | 136.7±65.6 | 311.1 | 25.5 | 48.0 |
|  | 2016 | Shenyang | 128.8±41.9 | 231.5 | 35.0 | 32.5 |
| Grain yield per plant (g) | 2015 | Harbin | 16.9±5.8 | 34.4 | 0.7 | 34.0 |
|  | 2015 | Changchun | 20.0±6.6 | 44.4 | 4.7 | 33.2 |
|  | 2015 | Shenyang | 14.0±4.4 | 25.2 | 6.4 | 31.6 |
|  | 2016 | Harbin | 14.1±5.3 | 29.9 | 0.2 | 37.7 |
|  | 2016 | Changchun | 26.7±13.4 | 71.9 | 5.8 | 50.2 |
|  | 2016 | Shenyang | 25.8±9.1 | 49.3 | 2.6 | 35.3 |
| Hundred-grain weight (g) | 2015 | Harbin | 19.2±4.5 | 35.7 | 2.8 | 23.6 |
|  | 2015 | Changchun | 18.1±4.4 | 38.4 | 3.2 | 24.4 |
|  | 2015 | Shenyang | 19.0±4.2 | 31.0 | 7.3 | 22.0 |
|  | 2016 | Harbin | 22.3±5.4 | 41.6 | 7.3 | 24.0 |
|  | 2016 | Changchun | 19.6±3.7 | 28.3 | 7.4 | 18.8 |
|  | 2016 | Shenyang | 20.4±3.9 | 32.1 | 5.7 | 19.3 |
| R1(day) | 2015 | Harbin | 42.6±13.2 | 78.6 | 29.5 | 31.0 |
|  | 2015 | Changchun | 37.7±8.8 | 76.9 | 29.0 | 22.1 |
|  | 2015 | Shenyang | 36.3±8.2 | 68.1 | 27.0 | 23.5 |
|  | 2016 | Harbin | 43.2±12.1 | 90.1 | 31.4 | 28.1 |
|  | 2016 | Changchun | 38.3±8.5 | 68.1 | 29.0 | 22.3 |
|  | 2016 | Shenyang | 35.8±9.0 | 64.3 | 22.0 | 25.1 |
| R2(day) | 2015 | Harbin | 54.4±11.4 | 82.4 | 38.3 | 20.9 |
|  | 2015 | Changchun | 47.0±8.6 | 77.5 | 36.6 | 18.6 |
|  | 2015 | Shenyang | 45.0±8.1 | 71.0 | 34.5 | 18.3 |
|  | 2016 | Harbin | 49.8±10.9 | 93.1 | 37.8 | 22.0 |
|  | 2016 | Changchun | 45.9±8.3 | 78.0 | 36.0 | 18.1 |
|  | 2016 | Shenyang | 44.1±9.7 | 70.6 | 28.6 | 22.0 |
| R3(day) | 2015 | Harbin | 63.1±10.9 | 90.4 | 44.0 | 17.3 |
|  | 2015 | Changchun | 54.3±9.2 | 84.9 | 43.2 | 17.5 |
|  | 2015 | Shenyang | 53.3±7.5 | 83.0 | 42.5 | 17.0 |
|  | 2016 | Harbin | 59.7±11.3 | 100.1 | 42.8 | 19.2 |
|  | 2016 | Changchun | 58.1±7.9 | 88.0 | 41.5 | 13.3 |
|  | 2016 | Shenyang | 53.3±10.4 | 80.6 | 36.4 | 19.6 |
| R4(day) | 2015 | Harbin | 70.8±10.7 | 97.4 | 53.7 | 15.1 |
|  | 2015 | Changchun | 62.9±8.7 | 92.5 | 50.5 | 14.0 |
|  | 2015 | Shenyang | 60.5±7.9 | 91.0 | 48.1 | 13.8 |
|  | 2016 | Harbin | 65.7±12.7 | 106.1 | 48.1 | 19.3 |
|  | 2016 | Changchun | 65.0±8.3 | 93.0 | 47.8 | 12.7 |
|  | 2016 | Shenyang | 58.1±10.9 | 86.6 | 40.3 | 18.7 |
| R5(day) | 2015 | Harbin | 77.6±10.8 | 105.6 | 60.1 | 13.9 |
|  | 2015 | Changchun | 69.2±8.3 | 101.0 | 59.5 | 12.6 |
|  | 2015 | Shenyang | 68.0±8.2 | 97.5 | 54.1 | 12.0 |
|  | 2016 | Harbin | 73.4±12.9 | 111.1 | 55.8 | 17.5 |
|  | 2016 | Changchun | 71.6±8.6 | 107.4 | 54.1 | 12.0 |
|  | 2016 | Shenyang | 66.4±11.7 | 101.4 | 47.0 | 17.7 |
| R6(day) | 2015 | Harbin | 85.0±10.3 | 116.2 | 66.5 | 12.2 |
|  | 2015 | Changchun | 78.5±7.4 | 111.2 | 64.0 | 12.8 |
|  | 2015 | Shenyang | 76.1±10.5 | 109.0 | 61.2 | 13.4 |
|  | 2016 | Harbin | 85.3±15.7 | 131.1 | 60.8 | 18.4 |
|  | 2016 | Changchun | 80.1±10.5 | 116.2 | 61.2 | 13.1 |
|  | 2016 | Shenyang | 79.7±11.3 | 118.4 | 60.4 | 14.2 |
| R7(day) | 2015 | Harbin | 113.7±8.7 | 130.0 | 90.7 | 17.7 |
|  | 2015 | Changchun | 100.4±11.5 | 128.9 | 84.5 | 13.2 |
|  | 2015 | Shenyang | 98.1±8.0 | 124.5 | 74.5 | 14.4 |
|  | 2016 | Harbin | 112±15.8 | 143.0 | 84.8 | 16.1 |
|  | 2016 | Changchun | 103.8±7.8 | 124.5 | 80.5 | 17.6 |
|  | 2016 | Shenyang | 101.0±13.0 | 123.3 | 82.8 | 14.5 |

R1: beginning bloom, R2: full bloom, R3: beginning pod, R4: full pod, R5: beginning seed, R6: full seed, R7: beginning maturity.

**Supplementary Table 5.** The partial correlation analysis of main agronomic traits.

| **r(y, x)** | **Partial correlation coefficient** | **t-test** | ***P*-value** |
| --- | --- | --- | --- |
| r(Grain weight per plant, Number of pods per plant) | 0.0684 | 3.1206 | 0.0018 |
| r(Grain weight per plant, Grain number per plant) | 0.8631 | 77.7902 | 0.0001 |
| r(Grain weight per plant, Hundred-grain weight) | 0.8786 | 83.7572 | 0.0001 |

The correlation coefficient was 0.97.

**Supplementary Table 6.** The combined analysis of variance (ANOVA) of fourteen agronomic traits of 173 soybean varieties at three locations in 2015 and 2016.

| **Traits** | **Source of variation** | **Df** | **SS** | **MS** | **F value** | **Percentage of treatment SS/%** | ***h^2^*(%)** |
| --- | --- | --- | --- | --- | --- | --- | --- |
| Plant high  (cm) | Locality | 2 | 15.9 | 8.0 | 16.4** | 0.8 | 86.1 |
|  | Year | 1 | 34.3 | 34.3 | 200.4** | 1.7 |  |
|  | Genotype | 172 | 1224.4 | 7.1 | 14.6** | 60.7 |  |
|  | Locality-by-Year | 2 | 37.3 | 18.6 | 108.8** | 1.8 |  |
|  | Locality-by-Genotype | 344 | 316.3 | 0.9 | 1.9** | 15.7 |  |
|  | Year-by-Genotype | 172 | 98.0 | 0.6 | 3.3** | 4.9 |  |
|  | Locality-by-Year-by-Genotype | 344 | 167.2 | 0.5 | 2.8** | 8.3 |  |
|  | Residuals | 1038 | 177.8 | 0.2 |  |  |  |
|  | Total | 2075 | 2071.3 |  |  |  |  |
| Number  of nodes of main  stem | Locality | 2 | 131.7 | 65.8 | 112.9** | 6.4 | 75.2 |
|  | Year | 1 | 44.6 | 44.6 | 156.4** | 2.2 |  |
|  | Genotype | 172 | 797.3 | 4.6 | 7.9** | 38.5 |  |
|  | Locality-by-Year | 2 | 52.5 | 26.3 | 92.1** | 2.5 |  |
|  | Locality-by-Genotype | 344 | 411.3 | 1.2 | 2.0** | 19.9 |  |
|  | Year-by-Genotype | 172 | 136.6 | 0.8 | 2.8** | 6.6 |  |
|  | Locality-by-Year-by-Genotype | 344 | 200.6 | 0.6 | 2.0** | 9.7 |  |
|  | Residuals | 1038 | 296.0 | 0.3 |  |  |  |
|  | Total | 2075 | 2070.7 |  |  |  |  |
| Number of  branches per plant | Locality | 2 | 120.3 | 60.2 | 85.0** | 4.3 | 85.8 |
|  | Year | 1 | 7.7 | 7.7 | 22.5** | 0.3 |  |
|  | Genotype | 172 | 1462.7 | 8.5 | 12.0** | 52.0 |  |
|  | Locality-by-Year | 2 | 178.3 | 89.1 | 260.5** | 6.3 |  |
|  | Locality-by-Genotype | 344 | 295.7 | 0.9 | 3.2** | 10.5 |  |
|  | Year-by-Genotype | 172 | 148.5 | 0.9 | 4.5** | 5.3 |  |
|  | Locality-by-Year-by-Genotype | 344 | 243.4 | 0.7 | 4.1** | 8.7 |  |
|  | Residuals | 1038 | 355.1 | 0.3 |  |  |  |
|  | Total | 2075 | 2811.8 |  |  |  |  |
| Number of  pods per plant | Locality | 2 | 101.3 | 190.7 | 229.5** | 4.9 | 66.0 |
|  | Year | 1 | 13.0 | 13.0 | 40.7** | 0.6 |  |
|  | Genotype | 172 | 808.9 | 3.1 | 3.7** | 39.1 |  |
|  | Locality-by-Year | 2 | 29.5 | 14.7 | 46.3** | 1.4 |  |
|  | Locality-by-Genotype | 344 | 325.8 | 0.9 | 4.1** | 15.7 |  |
|  | Year-by-Genotype | 172 | 176.6 | 1.0 | 6.2** | 8.5 |  |
|  | Locality-by-Year-by-Genotype | 344 | 285.8 | 0.8 | 5.6** | 13.8 |  |
|  | Residuals | 1038 | 330.4 | 0.3 |  |  |  |
|  | Total | 2075 | 2071.3 |  |  |  |  |
| Grain number per plant | Locality | 2 | 261.9 | 280.9 | 354.0** | 12.6 | 71.3 |
|  | Year | 1 | 1.2 | 1.2 | 4.0** | 0.1 |  |
|  | Genotype | 172 | 812.4 | 2.3 | 2.9** | 39.2 |  |
|  | Locality-by-Year | 2 | 35.8 | 17.9 | 59.5** | 1.7 |  |
|  | Locality-by-Genotype | 344 | 330.8 | 1 | 5.2** | 16.0 |  |
|  | Year-by-Genotype | 172 | 143.8 | 1 | 7.2** | 6.9 |  |
|  | Locality-by-Year-by-Genotype | 344 | 173 | 0.8 | 6.6** | 8.3 |  |
|  | Residuals | 1038 | 312.5 | 0.3 |  |  |  |
|  | Total | 2075 | 2071.4 |  |  |  |  |
| Grain yield per plant (g) | Locality | 2 | 302.1 | 276.1 | 360.9** | 14.6 | 70.7 |
|  | Year | 1 | 10.9 | 10.9 | 38.1** | 0.5 |  |
|  | Genotype | 172 | 747.2 | 2.7 | 3.5** | 36.1 |  |
|  | Locality-by-Year | 2 | 5.4 | 2.7 | 9.5** | 0.3 |  |
|  | Locality-by-Genotype | 344 | 312.2 | 0.9 | 5.2** | 15.1 |  |
|  | Year-by-Genotype | 172 | 154.3 | 1 | 7.6** | 7.4 |  |
|  | Locality-by-Year-by-Genotype | 344 | 243.2 | 0.8 | 6.7** | 11.7 |  |
|  | Residuals | 1038 | 296.2 | 0.3 |  |  |  |
|  | Total | 2075 | 2071.4 |  |  |  |  |
| Hundred-grain weight (g) | Locality | 2 | 56.2 | 28.1 | 45.7** | 2.7 | 82.1 |
|  | Year | 1 | 30.2 | 30.2 | 108.5** | 1.5 |  |
|  | Genotype | 172 | 984.0 | 5.7 | 9.3** | 47.5 |  |
|  | Locality-by-Year | 2 | 94.7 | 47.3 | 169.8** | 4.6 |  |
|  | Locality-by-Genotype | 344 | 284.5 | 0.8 | 5.3** | 13.7 |  |
|  | Year-by-Genotype | 172 | 120.5 | 0.7 | 6.5** | 5.8 |  |
|  | Locality-by-Year-by-Genotype | 344 | 211.4 | 0.6 | 6.2** | 10.2 |  |
|  | Residuals | 1038 | 289.3 | 0.3 |  |  |  |
|  | Total | 2075 | 2070.8 |  |  |  |  |
| R1(day) | Locality | 2 | 218.2 | 109.1 | 247.2** | 10.6 | 93.3 |
|  | Year | 1 | 57.8 | 57.8 | 9124.5** | 2.8 |  |
|  | Genotype | 172 | 1290.4 | 7.5 | 17.0** | 62.7 |  |
|  | Locality-by-Year | 2 | 157.3 | 78.7 | 12418.6** | 7.6 |  |
|  | Locality-by-Genotype | 344 | 134.0 | 0.4 | 10.9** | 6.5 |  |
|  | Year-by-Genotype | 172 | 43.2 | 0.3 | 39.6** | 2.1 |  |
|  | Locality-by-Year-by-Genotype | 344 | 151.8 | 0.4 | 69.7** | 7.4 |  |
|  | Residuals | 1038 | 6.6 | 0.01 |  |  |  |
|  | Total | 2075 | 2059.2 |  |  |  |  |
| R2(day) | Locality | 2 | 390.3 | 195.1 | 651.8** | 18.9 | 93.9 |
|  | Year | 1 | 5.1 | 5.1 | 1419.8** | 0.2 |  |
|  | Genotype | 172 | 1337.3 | 7.8 | 26.0** | 64.8 |  |
|  | Locality-by-Year | 2 | 40.5 | 20.2 | 5647.3** | 2.0 |  |
|  | Locality-by-Genotype | 344 | 132.0 | 0.4 | 11.3** | 6.4 |  |
|  | Year-by-Genotype | 172 | 51.4 | 0.3 | 83.3** | 2.5 |  |
|  | Locality-by-Year-by-Genotype | 344 | 103.0 | 0.3 | 83.6** | 5.0 |  |
|  | Residuals | 1038 | 3.7 | 0.004 |  |  |  |
|  | Total | 2075 | 2063.3 |  |  |  |  |
| R3(day) | Locality | 2 | 201.2 | 100.6 | 340.6** | 9.7 | 94.3 |
|  | Year | 1 | 0.2 | 0.2 | 24.4** | 0.01 |  |
|  | Genotype | 172 | 1431.1 | 8.3 | 28.2** | 69.3 |  |
|  | Locality-by-Year | 2 | 149.0 | 74.5 | 7287.9** | 7.2 |  |
|  | Locality-by-Genotype | 344 | 103.0 | 0.3 | 11.0** | 5.0 |  |
|  | Year-by-Genotype | 172 | 68.3 | 0.4 | 38.8** | 3.3 |  |
|  | Locality-by-Year-by-Genotype | 344 | 101.6 | 0.3 | 28.9** | 4.9 |  |
|  | Residuals | 1038 | 10.6 | 0.01 |  |  |  |
|  | Total | 2075 | 2064.9 |  |  |  |  |
| R4(day) | Locality | 2 | 329.8 | 164.9 | 652.4** | 16 | 95.1 |
|  | Year | 1 | 3.2 | 3.2 | 1190.5** | 0.2 |  |
|  | Genotype | 172 | 1378.1 | 8.0 | 31.7** | 66.7 |  |
|  | Locality-by-Year | 2 | 124.0 | 62 | 22772.4** | 6 |  |
|  | Locality-by-Genotype | 344 | 82.3 | 0.2 | 10.9** | 4 |  |
|  | Year-by-Genotype | 172 | 57.4 | 0.3 | 122.5** | 2.8 |  |
|  | Locality-by-Year-by-Genotype | 344 | 87.0 | 0.3 | 92.8** | 4.2 |  |
|  | Residuals | 1038 | 2.8 | 0.003 |  |  |  |
|  | Total | 2075 | 2064.6 |  |  |  |  |
| R5(day) | Locality | 2 | 288.1 | 144.1 | 513.7** | 14 | 94.6 |
|  | Year | 1 | 0.03 | 0.03 | 11.3** | 0.001 |  |
|  | Genotype | 172 | 1418.8 | 8.2 | 29.4** | 68.8 |  |
|  | Locality-by-Year | 2 | 95.7 | 47.8 | 15534.3** | 4.6 |  |
|  | Locality-by-Genotype | 344 | 93.5 | 0.3 | 11.0** | 4.5 |  |
|  | Year-by-Genotype | 172 | 66.9 | 0.4 | 126.3** | 3.2 |  |
|  | Locality-by-Year-by-Genotype | 344 | 96.5 | 0.3 | 91.0** | 4.7 |  |
|  | Residuals | 1038 | 3.2 | 0.003 |  |  |  |
|  | Total | 2075 | 2062.7 |  |  |  |  |
| R6(day) | Locality | 2 | 244.5 | 122.2 | 235.1** | 11.8 | 91.4 |
|  | Year | 1 | 52.7 | 52.7 | 17425.0** | 2.6 |  |
|  | Genotype | 172 | 1296.8 | 7.5 | 14.5** | 62.8 |  |
|  | Locality-by-Year | 2 | 62.3 | 31.2 | 10304.7** | 3.0 |  |
|  | Locality-by-Genotype | 344 | 131.9 | 0.4 | 10.7** | 6.4 |  |
|  | Year-by-Genotype | 172 | 95.3 | 0.6 | 183.2** | 4.6 |  |
|  | Locality-by-Year-by-Genotype | 344 | 178.9 | 0.5 | 172.0** | 8.7 |  |
|  | Residuals | 1038 | 3.1 | 0.003 |  |  |  |
|  | Total | 2075 | 2065.5 |  |  |  |  |
| R7(day) | Locality | 2 | 254.1 | 127.0 | 306.4** | 12.1 | 90.5 |
|  | Year | 1 | 42.8 | 42.8 | 9283.6** | 2.0 |  |
|  | Genotype | 172 | 1282.3 | 7.5 | 18.0** | 61.0 |  |
|  | Locality-by-Year | 2 | 92.3 | 46.1 | 10009.7** | 4.4 |  |
|  | Locality-by-Genotype | 344 | 190.5 | 0.6 | 16.3** | 9.1 |  |
|  | Year-by-Genotype | 172 | 94.1 | 0.5 | 118.7** | 4.5 |  |
|  | Locality-by-Year-by-Genotype | 344 | 142.6 | 0.4 | 90.0** | 6.8 |  |
|  | Residuals | 1038 | 4.8 | 0.005 |  |  |  |
|  | Total | 2075 | 2103.5 |  |  |  |  |

** *P*<0.01, * *P*<0.05. R1: beginning bloom, R2: full bloom, R3: beginning pod, R4: full pod, R5: beginning seed, R6: full seed, R7: beginning maturity.

Df: degree of freedom, SS: sum square, MS: mean square, *h^2^*: broad-sense heritability.

**Supplementary Table 7.** The analysis on the variance of additive main effect and multiplicative interactions (AMMI) for the fourteen agronomic traits of 173 soybean varieties across environments.

| **Traits** | **Source of variation** | **Df** | **SS** | **MS** | **F value** | **Percentage of treatment SS/%** |
| --- | --- | --- | --- | --- | --- | --- |
| Plant  high  (cm) | Environment | 2 | 3210.0 | 1605.1 | 697.2*** | 0.8 |
|  | Repetition (Environment) | 3 | 170.8 | 3.2 | 1.1 | 0.0 |
|  | Genotype | 172 | 284330.0 | 1653.1 | 276.0*** | 73.3 |
|  | Genotype-by-Environment | 344 | 72982.0 | 212.2 | 28.9*** | 18.8 |
|  | Residuals | 516 | 27064.5 | 2.9 |  |  |
| Number of nodes of main stem | Environment | 2 | 537.0 | 268.5 | 310.5*** | 6.6 |
|  | Repetition (Environment) | 3 | 19.9 | 0.4 | 4.8 | 0.2 |
|  | Genotype | 172 | 4545.4 | 26.4 | 150.8*** | 55.7 |
|  | Genotype-by-Environment | 344 | 2343.7 | 6.8 | 30.8*** | 28.7 |
|  | Residuals | 516 | 711.3 | 0.08 |  |  |
| Number of branches per plant | Environment | 2 | 164.5 | 82.3 | 160.8*** | 5.8 |
|  | Repetition (Environment) | 3 | 11.1 | 0.2 | 5.4 | 0.4 |
|  | Genotype | 172 | 1900.9 | 11.1 | 152.9*** | 67.0 |
|  | Genotype-by-Environment | 344 | 402.9 | 1.2 | 21.8*** | 14.2 |
|  | Residuals | 516 | 358.9 | 0.04 |  |  |
| Number of pods per plant | Environment | 2 | 56216.0 | 53608.0 | 2063.1*** | 13.0 |
|  | Repetition (Environment) | 3 | 365.7 | 6.8 | 0.9 | 0.1 |
|  | Genotype | 172 | 231960.0 | 1052.0 | 90.7*** | 53.8 |
|  | Genotype-by-Environment | 344 | 107466.0 | 312.0 | 30.1*** | 24.9 |
|  | Residuals | 516 | 34777.2 | 7.0 |  |  |
| Grain number per plant | Environment | 2 | 245648 | 322824 | 2277.2*** | 13.4 |
|  | Repetition (Environment) | 3 | 1813.4 | 33.6 | 0.8 | 0.1 |
|  | Genotype | 172 | 983282 | 3391 | 52.0*** | 53.5 |
|  | Genotype-by-Environment | 344 | 456765 | 1328 | 25.2*** | 24.8 |
|  | Residuals | 516 | 151602.2 | 37.9 |  |  |
| Grain yield per plant (g) | Environment | 2 | 5361 | 13180.7 | 69.9*** | 7.8 |
|  | Repetition (Environment) | 3 | 3261.8 | 60.4 | 3.1 | 4.2 |
|  | Genotype | 172 | 34606 | 137.2 | 10.9*** | 50.5 |
|  | Genotype-by-Environment | 344 | 19343 | 56.2 | 9.9*** | 24.7 |
|  | Residuals | 516 | 5892.8 | 19.5 |  |  |
| Hundred-grain weight (g) | Environment | 2 | 737.2 | 368.6 | 15.2*** | 3.4 |
|  | Repetition (Environment) | 3 | 1759.5 | 32.6 | 2.5 | 8.1 |
|  | Genotype | 172 | 12157.2 | 70.7 | 10.9*** | 56.2 |
|  | Genotype-by-Environment | 344 | 5218.7 | 15.2 | 9.1*** | 24.1 |
|  | Residuals | 516 | 1762.0 | 12.8 |  |  |
| R1(day) | Environment | 2 | 15257.0 | 7628.5 | 7523.5*** | 12.2 |
|  | Repetition (Environment) | 3 | 17.5 | 0.3 | 3.6 | 0.0 |
|  | Genotype | 172 | 102892.0 | 598.2 | 36552.7*** | 82.1 |
|  | Genotype-by-Environment | 344 | 7042.0 | 20.5 | 999.0*** | 5.6 |
|  | Residuals | 516 | 75.9 | 0.005 |  |  |
| R2(day) | Environment | 2 | 20244.0 | 10122.2 | 6055.9*** | 17.1 |
|  | Repetition (Environment) | 3 | 19.5 | 0.4 | 4.1 | 0.0 |
|  | Genotype | 172 | 92496.0 | 537.8 | 34468.2*** | 78.2 |
|  | Genotype-by-Environment | 344 | 5422.0 | 15.8 | 886.2*** | 4.6 |
|  | Residuals | 516 | 72.7 | 0.005 |  |  |
| R3(day) | Environment | 2 | 11383.0 | 5691.4 | 2058.3*** | 9.5 |
|  | Repetition (Environment) | 3 | 46.6 | 0.9 | 1.3 | 0.0 |
|  | Genotype | 172 | 95487.0 | 555.2 | 426.5*** | 80.0 |
|  | Genotype-by-Environment | 344 | 6273.0 | 18.2 | 13.6*** | 5.3 |
|  | Residuals | 516 | 6133.3 | 0.7 |  |  |
| R4(day) | Environment | 2 | 21106.0 | 10553.0 | 5601.8*** | 17.0 |
|  | Repetition (Environment) | 3 | 26.0 | 0.5 | 4.4 | 0.0 |
|  | Genotype | 172 | 97449.0 | 566.6 | 34639.2*** | 78.6 |
|  | Genotype-by-Environment | 344 | 5365.0 | 15.6 | 979.0*** | 4.3 |
|  | Residuals | 516 | 76.4 | 0.005 |  |  |
| R5(day) | Environment | 2 | 18166.0 | 9083.2 | 4550.9*** | 14.8 |
|  | Repetition (Environment) | 3 | 27.2 | 0.5 | 2.5 | 0.0 |
|  | Genotype | 172 | 98653.0 | 573.6 | 16318.7*** | 80.3 |
|  | Genotype-by-Environment | 344 | 5922.0 | 17.2 | 539.9*** | 4.8 |
|  | Residuals | 516 | 163.6 | 0.005 |  |  |
| R6(day) | Environment | 2 | 16891.0 | 8445.6 | 6171.4*** | 13.3 |
|  | Repetition (Environment) | 3 | 25.1 | 0.5 | 2.6 | 0.0 |
|  | Genotype | 172 | 101958.0 | 592.8 | 38374.1*** | 80.3 |
|  | Genotype-by-Environment | 344 | 8035.0 | 23.4 | 2162.3*** | 6.3 |
|  | Residuals | 516 | 72.4 | 0.005 |  |  |
| R7(day) | Environment | 2 | 27540.0 | 13770.2 | 7025.7*** | 17.2 |
|  | Repetition (Environment) | 3 | 27.1 | 0.5 | 3.3 | 0.0 |
|  | Genotype | 172 | 119168.0 | 692.8 | 18218.8*** | 74.5 |
|  | Genotype-by-Environment | 344 | 12946.0 | 37.6 | 1117.6*** | 8.1 |
|  | Residuals | 516 | 180.8 | 0.005 |  |  |

*** 0<*P*<0.001, ** 0.001<*P*<0.01, * 0.01<*P*<0.05

R1: beginning bloom, R2: full bloom, R3: beginning pod, R4: full pod, R5: beginning seed, R6: full seed, R7: beginning maturity.

Df : degree of freedom, SS : sum square, MS : mean square.

**Supplementary Table 8.** The maturity group (MGs) classification thresholds based on the growth periods of nineteen soybeans in strand MGs from North America.

| **Maturity group** | **Mean (day)** | **Range (day)** |
| --- | --- | --- |
| 000 | 85.8 | <85.8 |
| 00 | 89.8 | 85.9-87.8 |
| 0 | 104.7 | 87.9-97.3 |
| I | 114.2 | 97.4-109.5 |
| II | 119.3 | 109.6-116.7 |
| III | 125.9 | 116.8-122.6 |
| IV | 129.9 | >122.6 |

**Supplementary Table 9.** The maturity groups (MGs) classification of 173 soybean genotypes.

| **Number** | **Accession** | **Origin** | **MGs** |
| --- | --- | --- | --- |
| G1 | Tiedou 50 | Liaoning,China | MGI |
| G2 | Datun xiaoheidou | Hebei,China | MGIII |
| G3 | Ken 04-8579 | Heilongjiang,China | MG0 |
| G4 | Kangxian2 | Heilongjiang,China | MG00 |
| G5 | Liushitianhuancang | Liaoning,China | MGI |
| G6 | Tiedou 58 | Liaoning,China | MGII |
| G7 | Zhonghuang 20 | Beijing,China | MGII |
| G8 | Jichanghuangdou 1 | Xinjiang,China | MGII |
| G9 | Longquandadou | Heilongjiang,China | MG0 |
| G10 | Xiaobaiqi | Liaoning,China | MGII |
| G11 | Gongye 04L-141 | Jilin,China | MG0 |
| G12 | Tiedou 52 | Liaoning,China | MGII |
| G13 | L-57 | America | MGI |
| G14 | He 05-31 | Heilongjiang,China | MG0 |
| G15 | L-21 | Canada | MG0 |
| G16 | Hongfeng11 | Heilongjiang,China | MG0 |
| G17 | Heinong 33 | Heilongjiang,China | MG0 |
| G18 | Liao 98072 | Liaoning,China | MGII |
| G19 | Kenfeng 22 | Heilongjiang,China | MG0 |
| G20 | Jiyu 89 | Jilin,China | MG0 |
| G21 | Tiedou 51 | Liaoning,China | MGII |
| G22 | Tongnong 13 | Jilin,China | MGII |
| G23 | Bei 1873 | Heilongjiang,China | MG0 |
| G24 | Hefeng 50 | Heilongjiang,China | MG0 |
| G25 | Sui 02-339 | Heilongjiang,China | MG0 |
| G26 | Hujiao 03-286 | Inner Mongolia,China | MG00 |
| G27 | Heinong 44 | Heilongjiang,China | MG0 |
| G28 | Hujiao 04-528 | Inner Mongolia,China | MG00 |
| G29 | Fengshou 10 | Heilongjiang,China | MG0 |
| G30 | Hersong 2 | Ukraine | MG0 |
| G31 | Jinzhou 4-1 | Liaoning,China | MGII |
| G32 | Suinong 14 | Heilongjiang,China | MG0 |
| G33 | Tiejiasilihuang | Jilin,China | MGI |
| G34 | Tiefeng 8 | Liaoning,China | MGIII |
| G35 | Silihuang | Jilin,China | MG0 |
| G36 | Bei 1361 | Heilongjiang,China | MG0 |
| G37 | L-5 | Canada | MGII |
| G38 | Maoyandou | Gansu, China | MGI |
| G39 | Fengdihuang | Jilin,China | MGII |
| G40 | Tiedou 54 | Liaoning,China | MGII |
| G41 | Jiunong 20 | Jilin,China | MGI |
| G42 | Duludou | Jilin,China | MG0 |
| G43 | Sui 03-3046 | Heilongjiang,China | MG0 |
| G44 | Hefeng 47 | Heilongjiang,China | MG0 |
| G45 | V111-4 | Jilin,China | MGI |
| G46 | Longxuan 1 | Heilongjiang,China | MG0 |
| G47 | Dongnong 8004 | Heilongjiang,China | MG0 |
| G48 | Sui 03-3952 | Heilongjiang,China | MG0 |
| G49 | Hefeng 25 | Heilongjiang,China | MG0 |
| G50 | Liaodou 3 | Liaoning,China | MGI |
| G51 | Hujiao 423 | Inner Mongolia,China | MG0 |
| G52 | Liaoxian 1 | Liaoning,China | MGI |
| G53 | Zhongpin 95-5388 | Beijing,China | MGIII |
| G54 | Heihe 45 | Heilongjiang,China | MG00 |
| G55 | L-59 Peking | America | MGIII |
| G56 | Sui 04-5804 | Heilongjiang,China | MG0 |
| G57 | Ji 06B7 | Hebei,China | MGIV |
| G58 | Marca. Joe lisa | Heilongjiang,China | MGI |
| G59 | Jihuang 13 | Hebei,China | MGIII |
| G60 | L-28 | Canada | MG0 |
| G61 | Beifeng 9 | Heilongjiang,China | MG0 |
| G62 | Zhongzuo 00-683 | Beijing,China | MGII |
| G63 | Dongnong 50 | Heilongjiang,China | MG0 |
| G64 | Dongnong 93-046 | Heilongjiang,China | MG0 |
| G65 | Suinong 10 | Heilongjiang,China | MG0 |
| G66 | Tiefeng 31 | Liaoning,China | MGIII |
| G67 | Dongnong 49 | Heilongjiang,China | MG00 |
| G68 | Huayou 446 | Hebei,China | MGIII |
| G69 | Heihe 48 | Heilongjiang,China | MG00 |
| G70 | Huajiang 2 | Heilongjiang,China | MG000 |
| G71 | Suinong 29 | Heilongjiang,China | MG0 |
| G72 | Hefeng 35 | Heilongjiang,China | MG0 |
| G73 | Suinong 8 | Heilongjiang,China | MG0 |
| G74 | Jilin 47 | Jilin,China | MGI |
| G75 | Heinong 48 | Heilongjiang,China | MG0 |
| G76 | Nova | Italy | MGI |
| G77 | Beidou 14 | Heilongjiang,China | MG00 |
| G78 | Kangxian 3 | Heilongjiang,China | MG0 |
| G79 | Dongnong 42 | Heilongjiang,China | MG0 |
| G80 | Kenjian 23 | Heilongjiang,China | MG0 |
| G81 | Heinong 55 | Heilongjiang,China | MG0 |
| G82 | Hefeng 45 | Heilongjiang,China | MG0 |
| G83 | Kenfeng 15 | Heilongjiang,China | MG00 |
| G84 | Chasedou | Jilin,China | MGI |
| G85 | He 05-991 | Heilongjiang,China | MG00 |
| G86 | Xiaolimoshidou | Heilongjiang,China | MG0 |
| G87 | Jingshanpu | Heilongjiang,China | MGI |
| G88 | Qinganheidou | Heilongjiang,China | MGI |
| G89 | Dongnong594 | Heilongjiang,China | MGI |
| G90 | Yapoche | Heilongjiang,China | MGI |
| G91 | Kennong 29 | Heilongjiang,China | MG0 |
| G92 | Heimoshidou | Jilin,China | MGII |
| G93 | Ha 04-1824 | Heilongjiang,China | MG0 |
| G94 | Fangzhengmoshidou | Heilongjiang,China | MGI |
| G95 | Helongyoutai | Jilin,China | MG0 |
| G96 | Kexin 3 | Beijing,China | MGII |
| G97 | Yuanbaojin | Heilongjiang,China | MG0 |
| G98 | Zhongzuo J4133 | Beijing,China | MGIII |
| G99 | Jilin 30 | Jilin,China | MGII |
| G100 | Ji 100 | Jilin,China | MGI |
| G101 | Suinong 30 | Heilongjiang,China | MG0 |
| G102 | Boige du | Germany | MG0 |
| G103 | Beidou 16 | Heilongjiang,China | MGI |
| G104 | Kebei 1 | Heilongjiang,China | MG0 |
| G105 | Mengdou 21 | Inner Mongolia,China | MG00 |
| G106 | Heihe 38 | Heilongjiang,China | MG00 |
| G107 | Zhongdou 35 | Beijing,China | MGIII |
| G108 | Zhonghuang 6 | Beijing,China | MGII |
| G109 | Jiyu 94 | Jilin,China | MGII |
| G110 | Dongnong 44 | Heilongjiang,China | MG00 |
| G111 | Liaonong 2 | Liaoning,China | MGII |
| G112 | Bei 1484 | America | MG00 |
| G113 | Williams82 | America | MGII |
| G114 | Jindou 33 | Liaoning,China | MGIII |
| G115 | Kenfeng 18 | Heilongjiang,China | MGI |
| G116 | Suinong 20 | Heilongjiang,China | MG0 |
| G117 | Mengdou 9 | Inner Mongolia,China | MG00 |
| G118 | L-9 | America | MGIV |
| G119 | Suinong 1 | Heilongjiang,China | MGI |
| G120 | Kenfeng 14 | Heilongjiang,China | MGI |
| G121 | Tiejiazi | Liaoning,China | MG0 |
| G122 | Suinong 4 | Heilongjiang,China | MG0 |
| G123 | Jinshanchamoshidou | Jilin,China | MGII |
| G124 | Longpin 03-311 | Heilongjiang,China | MG0 |
| G125 | Jilinchalihua | Jilin,China | MGII |
| G126 | Heinong 37 | Heilongjiang,China | MGI |
| G127 | Huajiang 4403 | Heilongjiang,China | MG0 |
| G128 | Huangbaozhu | Liaoning,China | MGII |
| G129 | Suinong 25 | Heilongjiang,China | MG0 |
| G130 | Sui 05-7304 | Heilongjiang,China | MG0 |
| G131 | Jidou 9 | Hebei,China | MGIII |
| G132 | Mengdou 14 | Inner Mongolia,China | MG0 |
| G133 | Handou 5 | Hebei,China | MGIII |
| G134 | Jidou 17 | Hebei,China | MGIII |
| G135 | Aika 166 | Heilongjiang,China | MGI |
| G136 | Sui 04-6018 | Heilongjiang,China | MG0 |
| G137 | Dongnong 48 | Heilongjiang,China | MG0 |
| G138 | L-79 | America | MG00 |
| G139 | Zhonghuang 35 | Beijing,China | MGII |
| G140 | Kennong 30 | Heilongjiang,China | MG0 |
| G141 | Zhonghuang 30 | Beijing,China | MGII |
| G142 | Baichengmoshidou | Jilin,China | MGI |
| G143 | L-10 | America | MGII |
| G144 | Zaoshu 18 | Beijing,China | MGII |
| G145 | Zhonghuang 10 | Beijing,China | MGII |
| G146 | Dongnong L-13 | Heilongjiang,China | MGI |
| G147 | Neifeng 15 | Heilongjiang,China | MG0 |
| G148 | Dongnong 47 | Heilongjiang,China | MG0 |
| G149 | Hefeng 29 | Heilongjiang,China | MGI |
| G150 | Dongnong 07-909 | Heilongjiang,China | MG0 |
| G151 | Hefeng 52 | Heilongjiang,China | MG0 |
| G152 | Heihe 18 | Heilongjiang,China | MG000 |
| G153 | Neifeng 11 | Heilongjiang,China | MG0 |
| G154 | Heihexiaohuangdou | Heilongjiang,China | MG00 |
| G155 | Zhongdou 27 | Beijing,China | MGII |
| G156 | Bei 4834 | Heilongjiang,China | MGI |
| G157 | Heilongjiang 41 | Heilongjiang,China | MGI |
| G158 | Zhongpin 03-5179 | Beijing,China | MGII |
| G159 | Hefeng 37 | Heilongjiang,China | MGI |
| G160 | Dongnong 46 | Heilongjiang,China | MG0 |
| G161 | Dongnong 43 | Heilongjiang,China | MG0 |
| G162 | Dongnong 1068 | Heilongjiang,China | MG0 |
| G163 | Dongnong 56 | Heilongjiang,China | MGI |
| G164 | Jiunong 21 | Jilin,China | MGII |
| G165 | Chamoshidou | Jilin,China | MGII |
| G166 | Daliheidou | Shandong,China | MGI |
| G167 | Zhongpin 03-5373 | Beijing,China | MGIII |
| G168 | Wuxing 4 | Hebei,China | MGIV |
| G169 | Hefeng 55 | Heilongjiang,China | MG0 |
| G170 | Suinong 28 | Heilongjiang,China | MG0 |
| G171 | Zhongpin 03-5334 | Beijing,China | MGIII |
| G172 | Fengshou 6 | Heilongjiang,China | MG0 |
| G173 | Dunajika | Russia | MG0 |

**Supplementary Table 10.** The soybean genotypes with stable above- or below-average traits.

| **Traits** | | **Genotype** | | **Phenotypic value** |
| --- | --- | --- | --- | --- |
|  |  | **Number** | **Variety name** |  |
| Plant height (cm) | Higher | G7 | Zhonghuang 20 | 107.5 |
|  |  | G8 | Jichanghuangdou 1 | 105.9 |
|  |  | G10 | Xiaobaiqi | 104.4 |
|  |  | G11 | Gongye 04L-141 | 96.8 |
|  |  | G18 | Liao 98072 | 95.3 |
|  |  | G21 | Tiedou 51 | 89.3 |
|  |  | G30 | Hersong 2 | 95.9 |
|  |  | G34 | Tiefeng 8 | 94.7 |
|  |  | G45 | V111-4 | 87.4 |
|  |  | G64 | Dongnong 93-046 | 95.7 |
|  |  | G66 | Tiefeng 31 | 88.9 |
|  |  | G79 | Dongnong 42 | 95.1 |
|  |  | G90 | Yapoche | 93.3 |
|  |  | G92 | Heimoshidou | 123.9 |
|  |  | G97 | Yuanbaojin | 90.0 |
|  |  | G100 | Ji 100 | 86.7 |
|  |  | G114 | Jindou 33 | 106.1 |
|  |  | G118 | L-9 | 112.7 |
|  |  | G123 | Jinshanchamoshidou | 119.7 |
|  |  | G129 | Suinong 25 | 86.8 |
|  |  | G131 | Jidou 9 | 101.5 |
|  |  | G133 | Handou 5 | 101.8 |
|  |  | G145 | Zhonghuang 10 | 94.6 |
|  |  | G149 | Hefeng 29 | 88.9 |
|  |  | G155 | Zhongdou 27 | 93.8 |
|  |  | G157 | Heilongjiang 41 | 97.2 |
|  |  | G164 | Jiunong 21 | 90.1 |
|  | Lower | G24 | Hefeng 50 | 70.1 |
|  |  | G29 | Fengshou 10 | 79.7 |
|  |  | G36 | Bei 1361 | 77.0 |
|  |  | G47 | Dongnong 8004 | 63.5 |
|  |  | G52 | Liaoxian 1 | 29.8 |
|  |  | G60 | L-28 | 73.1 |
|  |  | G63 | Dongnong 50 | 75.2 |
|  |  | G67 | Dongnong 49 | 53.4 |
|  |  | G70 | Huajiang 2 | 54.3 |
|  |  | G77 | Beidou 14 | 73.0 |
|  |  | G91 | Kennong 29 | 62.1 |
|  |  | G117 | Mengdou 9 | 58.2 |
|  |  | G122 | Suinong 4 | 69.1 |
|  |  | G127 | Huajiang 4403 | 68.9 |
|  |  | G130 | Sui 05-7304 | 58.6 |
|  |  | G138 | L-79 | 61.0 |
|  |  | G148 | Dongnong 47 | 72.4 |
|  |  | G150 | Dongnong 07-909 | 75.4 |
|  |  | G161 | Dongnong 43 | 70.0 |
|  |  | G162 | Dongnong 1068 | 75.2 |
|  |  | G163 | Dongnong 56 | 73.6 |
|  |  | G169 | Hefeng 55 | 76.4 |
|  |  | G171 | Zhongpin 03-5334 | 71.6 |
|  |  | G172 | Fengshou 6 | 76.6 |
| Number of nodes of main stem | Higher | G6 | Tiedou 58 | 17.7 |
|  |  | G8 | Jichanghuangdou 1 | 18.2 |
|  |  | G10 | Xiaobaiqi | 18.4 |
|  |  | G13 | L-57 | 18.6 |
|  |  | G15 | L-21 | 17.4 |
|  |  | G21 | Tiedou 51 | 16.9 |
|  |  | G33 | Tiejiasilihuang | 19.4 |
|  |  | G40 | Tiedou 54 | 16.8 |
|  |  | G59 | Jihuang 13 | 20.0 |
|  |  | G63 | Dongnong 50 | 17.3 |
|  |  | G76 | Nova | 18.2 |
|  |  | G78 | Kangxian 3 | 18.4 |
|  |  | G82 | Hefeng 45 | 18.4 |
|  |  | G94 | Fangzhengmoshidou | 18.6 |
|  |  | G103 | Beidou 16 | 19.4 |
|  |  | G119 | Suinong 1 | 18.6 |
|  |  | G136 | Sui 04-6018 | 17.3 |
|  |  | G146 | Dongnong L-13 | 17.2 |
|  |  | G149 | Hefeng 29 | 18.3 |
|  | Lower | G12 | Tiedou 52 | 16.0 |
|  |  | G42 | Duludou | 14.9 |
|  |  | G43 | Sui 03-3046 | 13.0 |
|  |  | G46 | Longxuan 1 | 15.6 |
|  |  | G70 | Huajiang 2 | 14.1 |
|  |  | G72 | Hefeng 35 | 15.6 |
|  |  | G81 | Heinong 55 | 15.0 |
|  |  | G91 | Kennong 29 | 14.1 |
|  |  | G97 | Yuanbaojin | 14.3 |
|  |  | G100 | Ji 100 | 15.2 |
|  |  | G110 | Dongnong 44 | 13.7 |
|  |  | G112 | Bei 1484 | 15.5 |
|  |  | G130 | Sui 05-7304 | 13.8 |
|  |  | G132 | Mengdou 14 | 15.4 |
|  |  | G140 | Kennong 30 | 15.0 |
|  |  | G142 | Baichengmoshidou | 15.9 |
|  |  | G145 | Zhonghuang 10 | 16.2 |
|  |  | G147 | Neifeng 15 | 15.8 |
|  |  | G152 | Heihe 18 | 11.5 |
|  |  | G160 | Dongnong 46 | 15.8 |
|  |  | G161 | Dongnong 43 | 16.0 |
|  |  | G172 | Fengshou 6 | 15.9 |
| Number of branches per plant | Higher | G1 | Tiedou 50 | 2.8 |
|  |  | G18 | Liao 98072 | 2.1 |
|  |  | G21 | Tiedou 51 | 2.2 |
|  |  | G25 | Sui 02-339 | 2.4 |
|  |  | G33 | Tiejiasilihuang | 2.1 |
|  |  | G42 | Duludou | 2.0 |
|  |  | G47 | Dongnong 8004 | 2.7 |
|  |  | G53 | Zhongpin 95-5388 | 3.4 |
|  |  | G60 | L-28 | 2.9 |
|  |  | G86 | Xiaolimoshidou | 5.3 |
|  |  | G88 | Qinganheidou | 3.1 |
|  |  | G92 | Heimoshidou | 4.5 |
|  |  | G95 | Helongyoutai | 3.5 |
|  |  | G97 | Yuanbaojin | 3.5 |
|  |  | G107 | Zhongdou 35 | 2.9 |
|  |  | G122 | Suinong 4 | 1.9 |
|  |  | G139 | Zhonghuang 35 | 2.0 |
|  |  | G142 | Baichengmoshidou | 4.9 |
|  |  | G143 | L-10 | 2.7 |
|  |  | G158 | Zhongpin 03-5179 | 2.4 |
|  |  | G171 | Zhongpin 03-5334 | 5.4 |
|  | Lower | G26 | Hujiao 03-286 | 0.8 |
|  |  | G28 | Hujiao 04-528 | 0.9 |
|  |  | G61 | Beifeng 9 | 0.1 |
|  |  | G70 | Huajiang 2 | 0.4 |
|  |  | G72 | Hefeng 35 | 0.6 |
|  |  | G74 | Jilin 47 | 0.9 |
|  |  | G81 | Heinong 55 | 0.5 |
|  |  | G83 | Kenfeng 15 | 0.3 |
|  |  | G93 | Ha 04-1824 | 0.7 |
|  |  | G104 | Kebei 1 | 1.2 |
|  |  | G105 | Mengdou 21 | 0.5 |
|  |  | G112 | Bei 1484 | 1.1 |
|  |  | G115 | Kenfeng 18 | 1.1 |
|  |  | G117 | Mengdou 9 | 0.4 |
|  |  | G121 | Tiejiazi | 0.4 |
|  |  | G132 | Mengdou 14 | 1.4 |
|  |  | G136 | Sui 04-6018 | 0.3 |
|  |  | G140 | Kennong 30 | 1.0 |
|  |  | G146 | Dongnong L-13 | 0.8 |
| Number of pods per plant | Higher | G2 | Datun xiaoheidou | 81.9 |
|  |  | G8 | Jichanghuangdou 1 | 53.1 |
|  |  | G57 | Ji 06B7 | 62.1 |
|  |  | G62 | Zhongzuo 00-683 | 66.4 |
|  |  | G66 | Tiefeng 31 | 72.5 |
|  |  | G74 | Jilin 47 | 63.8 |
|  |  | G103 | Beidou 16 | 66.2 |
|  |  | G113 | Williams82 | 64.9 |
|  |  | G116 | Suinong 20 | 59.1 |
|  |  | G118 | L-9 | 75.3 |
|  |  | G122 | Suinong 4 | 51.7 |
|  |  | G131 | Jidou 9 | 63.8 |
|  |  | G135 | Aika 166 | 57.4 |
|  |  | G141 | Zhonghuang 30 | 59.5 |
|  |  | G156 | Bei 4834 | 51.2 |
|  |  | G173 | Dunajika | 59.1 |
|  | Lower | G51 | Hujiao 423 | 38.9 |
|  |  | G67 | Dongnong 49 | 34.1 |
|  |  | G71 | Suinong 29 | 33.0 |
|  |  | G72 | Hefeng 35 | 41.9 |
|  |  | G75 | Heinong 48 | 44.1 |
|  |  | G77 | Beidou 14 | 47.3 |
|  |  | G105 | Mengdou 21 | 43.0 |
|  |  | G115 | Kenfeng 18 | 45.4 |
|  |  | G120 | Kenfeng 14 | 44.1 |
|  |  | G132 | Mengdou 14 | 42.9 |
|  |  | G150 | Dongnong 07-909 | 46.3 |
|  |  | G161 | Dongnong 43 | 44.5 |
|  |  | G166 | Daliheidou | 39.8 |
|  |  | G172 | Fengshou 6 | 41.4 |
| Grain number per plant | Higher | G1 | Tiedou 50 | 130.3 |
|  |  | G40 | Tiedou 54 | 139.7 |
|  |  | G74 | Jilin 47 | 120.5 |
|  |  | G82 | Hefeng 45 | 112.0 |
|  |  | G99 | Jilin 30 | 117.2 |
|  |  | G113 | Williams82 | 118.7 |
|  |  | G114 | Jindou 33 | 103.9 |
|  |  | G134 | Jidou 17 | 141.0 |
|  |  | G141 | Zhonghuang 30 | 130.3 |
|  |  | G149 | Hefeng 29 | 114.3 |
|  |  | G167 | Zhongpin 03-5373 | 138.9 |
|  | Lower | G26 | Hujiao 03-286 | 71.9 |
|  |  | G29 | Fengshou 10 | 82.8 |
|  |  | G35 | Silihuang | 90.2 |
|  |  | G67 | Dongnong 49 | 68.1 |
|  |  | G75 | Heinong 48 | 90.2 |
|  |  | G85 | He 05-991 | 81.6 |
|  |  | G110 | Dongnong 44 | 57.7 |
|  |  | G124 | Longpin 03-311 | 89.2 |
|  |  | G132 | Mengdou 14 | 91.0 |
|  |  | G144 | Zaoshu 18 | 95.6 |
|  |  | G150 | Dongnong 07-909 | 91.7 |
|  |  | G166 | Daliheidou | 70.1 |
|  |  | G170 | Suinong 28 | 87.8 |
| Grain yield per plant (g) | Higher | G34 | Tiefeng 8 | 26.9 |
|  |  | G62 | Zhongzuo 00-683 | 26.5 |
|  |  | G66 | Tiefeng 31 | 29.1 |
|  |  | G89 | Dongnong594 | 19.7 |
|  |  | G111 | Liaonong 2 | 19.8 |
|  |  | G113 | Williams82 | 21.2 |
|  |  | G116 | Suinong 20 | 22.1 |
|  |  | G119 | Suinong 1 | 29.6 |
|  |  | G122 | Suinong 4 | 18.8 |
|  |  | G124 | Longpin 03-311 | 18.7 |
|  |  | G136 | Sui 04-6018 | 18.8 |
|  |  | G144 | Zaoshu 18 | 23.0 |
|  | Lower | G14 | He 05-31 | 16.7 |
|  |  | G28 | Hujiao 04-528 | 13.2 |
|  |  | G29 | Fengshou 10 | 16.6 |
|  |  | G42 | Duludou | 17.0 |
|  |  | G46 | Longxuan 1 | 16.0 |
|  |  | G51 | Hujiao 423 | 16.2 |
|  |  | G56 | Sui 04-5804 | 15.7 |
|  |  | G67 | Dongnong 49 | 11.7 |
|  |  | G71 | Suinong 29 | 14.4 |
|  |  | G90 | Yapoche | 16.9 |
|  |  | G95 | Helongyoutai | 15.9 |
|  |  | G110 | Dongnong 44 | 10.5 |
|  |  | G138 | L-79 | 11.4 |
|  |  | G140 | Kennong 30 | 16.9 |
|  |  | G147 | Neifeng 15 | 16.1 |
|  |  | G152 | Heihe 18 | 11.7 |
|  |  | G170 | Suinong 28 | 16.6 |
|  |  | G173 | Dunajika | 12.8 |
| Hundred-grain weight (g) | Higher | G9 | Longquandadou | 28.5 |
|  |  | G23 | Bei 1873 | 20.4 |
|  |  | G38 | Maoyandou | 22.2 |
|  |  | G45 | V111-4 | 20.4 |
|  |  | G59 | Jihuang 13 | 20.8 |
|  |  | G73 | Suinong 8 | 21.3 |
|  |  | G79 | Dongnong 42 | 24.0 |
|  |  | G106 | Heihe 38 | 21.7 |
|  |  | G114 | Jindou 33 | 24.0 |
|  |  | G120 | Kenfeng 14 | 20.5 |
|  |  | G121 | Tiejiazi | 22.4 |
|  |  | G130 | Sui 05-7304 | 21.3 |
|  |  | G156 | Bei 4834 | 24.3 |
|  |  | G169 | Hefeng 55 | 20.4 |
|  | Lower | G2 | Datun xiaoheidou | 11.9 |
|  |  | G5 | Liushitianhuancang | 15.0 |
|  |  | G8 | Jichanghuangdou 1 | 17.7 |
|  |  | G27 | Heinong 44 | 18.4 |
|  |  | G33 | Tiejiasilihuang | 16.9 |
|  |  | G42 | Duludou | 18.8 |
|  |  | G43 | Sui 03-3046 | 17.4 |
|  |  | G55 | L-59 Peking | 10.7 |
|  |  | G61 | Beifeng 9 | 17.5 |
|  |  | G63 | Dongnong 50 | 9.1 |
|  |  | G69 | Heihe 48 | 16.6 |
|  |  | G77 | Beidou 14 | 18.4 |
|  |  | G80 | Kenjian 23 | 18.1 |
|  |  | G92 | Heimoshidou | 13.8 |
|  |  | G99 | Jilin 30 | 18.3 |
|  |  | G113 | Williams82 | 18.0 |
|  |  | G123 | Jinshanchamoshidou | 12.5 |
|  |  | G125 | Jilinchalihua | 7.9 |
|  |  | G142 | Baichengmoshidou | 11.0 |
|  |  | G145 | Zhonghuang 10 | 17.7 |
|  |  | G173 | Dunajika | 11.8 |
| R1 (day) | Higher | G1 | Tiedou 50 | 46.4 |
|  |  | G2 | Datun xiaoheidou | 63.0 |
|  |  | G6 | Tiedou 58 | 60.3 |
|  |  | G7 | Zhonghuang 20 | 41.0 |
|  |  | G10 | Xiaobaiqi | 53.5 |
|  |  | G14 | He 05-31 | 40.2 |
|  |  | G18 | Liao 98072 | 46.2 |
|  |  | G30 | Hersong 2 | 47.7 |
|  |  | G31 | Jinzhou 4-1 | 55.5 |
|  |  | G34 | Tiefeng 8 | 67.6 |
|  |  | G39 | Fengdihuang | 51.4 |
|  |  | G40 | Tiedou 54 | 54.6 |
|  |  | G50 | Liaodou 3 | 42.2 |
|  |  | G53 | Zhongpin 95-5388 | 55.2 |
|  |  | G55 | L-59 Peking | 76.8 |
|  |  | G57 | Ji 06B7 | 69.1 |
|  |  | G58 | Marca. Joe lisa | 57.6 |
|  |  | G59 | Jihuang 13 | 49.0 |
|  |  | G66 | Tiefeng 31 | 44.0 |
|  |  | G68 | Huayou 446 | 52.9 |
|  |  | G86 | Xiaolimoshidou | 43.0 |
|  |  | G88 | Qinganheidou | 44.8 |
|  |  | G94 | Fangzhengmoshidou | 45.2 |
|  |  | G100 | Ji 100 | 41.6 |
|  |  | G103 | Beidou 16 | 42.8 |
|  |  | G108 | Zhonghuang 6 | 57.7 |
|  |  | G109 | Jiyu 94 | 59.0 |
|  |  | G113 | Williams82 | 44.4 |
|  |  | G114 | Jindou 33 | 61.9 |
|  |  | G118 | L-9 | 68.6 |
|  |  | G119 | Suinong 1 | 40.2 |
|  |  | G128 | Huangbaozhu | 49.4 |
|  |  | G131 | Jidou 9 | 45.7 |
|  |  | G134 | Jidou 17 | 53.6 |
|  |  | G141 | Zhonghuang 30 | 48.7 |
|  |  | G142 | Baichengmoshidou | 50.8 |
|  |  | G143 | L-10 | 43.8 |
|  |  | G145 | Zhonghuang 10 | 56.6 |
|  |  | G149 | Hefeng 29 | 42.1 |
|  |  | G155 | Zhongdou 27 | 50.8 |
|  |  | G157 | Heilongjiang 41 | 42.6 |
|  |  | G159 | Hefeng 37 | 67.5 |
|  |  | G165 | Chamoshidou | 58.8 |
|  |  | G167 | Zhongpin 03-5373 | 63.6 |
|  |  | G168 | Wuxing 4 | 66.8 |
|  |  | G171 | Zhongpin 03-5334 | 60.2 |
|  | Lower | G3 | Ken 04-8579 | 35.6 |
|  |  | G17 | Heinong 33 | 33.9 |
|  |  | G51 | Hujiao 423 | 35.4 |
|  |  | G61 | Beifeng 9 | 32.4 |
|  |  | G63 | Dongnong 50 | 33.8 |
|  |  | G70 | Huajiang 2 | 30.9 |
|  |  | G72 | Hefeng 35 | 35.1 |
|  |  | G79 | Dongnong 42 | 33.6 |
|  |  | G80 | Kenjian 23 | 33.5 |
|  |  | G83 | Kenfeng 15 | 32.4 |
|  |  | G85 | He 05-991 | 36.2 |
|  |  | G101 | Suinong 30 | 35.9 |
|  |  | G104 | Kebei 1 | 34.4 |
|  |  | G106 | Heihe 38 | 31.3 |
|  |  | G116 | Suinong 20 | 33.8 |
|  |  | G120 | Kenfeng 14 | 34.8 |
|  |  | G129 | Suinong 25 | 35.3 |
| R2 (day) | Higher | G2 | Datun xiaoheidou | 68.7 |
|  |  | G6 | Tiedou 58 | 66.1 |
|  |  | G7 | Zhonghuang 20 | 50.6 |
|  |  | G8 | Jichanghuangdou 1 | 64.0 |
|  |  | G10 | Xiaobaiqi | 60.5 |
|  |  | G11 | Gongye 04L-141 | 53.1 |
|  |  | G22 | Tongnong 13 | 49.4 |
|  |  | G30 | Hersong 2 | 53.6 |
|  |  | G31 | Jinzhou 4-1 | 63.0 |
|  |  | G33 | Tiejiasilihuang | 51.0 |
|  |  | G38 | Maoyandou | 50.5 |
|  |  | G39 | Fengdihuang | 60.2 |
|  |  | G40 | Tiedou 54 | 60.6 |
|  |  | G50 | Liaodou 3 | 50.8 |
|  |  | G53 | Zhongpin 95-5388 | 63.5 |
|  |  | G55 | L-59 Peking | 82.0 |
|  |  | G57 | Ji 06B7 | 77.3 |
|  |  | G58 | Marca. Joe lisa | 63.3 |
|  |  | G59 | Jihuang 13 | 60.6 |
|  |  | G66 | Tiefeng 31 | 50.8 |
|  |  | G68 | Huayou 446 | 61.2 |
|  |  | G74 | Jilin 47 | 49.5 |
|  |  | G86 | Xiaolimoshidou | 51.0 |
|  |  | G87 | Jingshanpu | 50.5 |
|  |  | G88 | Qinganheidou | 53.0 |
|  |  | G92 | Heimoshidou | 63.5 |
|  |  | G94 | Fangzhengmoshidou | 52.2 |
|  |  | G96 | Kexin 3 | 69.3 |
|  |  | G98 | Zhongzuo J4133 | 63.8 |
|  |  | G103 | Beidou 16 | 50.2 |
|  |  | G108 | Zhonghuang 6 | 65.4 |
|  |  | G109 | Jiyu 94 | 65.0 |
|  |  | G111 | Liaonong 2 | 55.3 |
|  |  | G113 | Williams82 | 50.7 |
|  |  | G114 | Jindou 33 | 68.9 |
|  |  | G118 | L-9 | 75.6 |
|  |  | G119 | Suinong 1 | 50.3 |
|  |  | G123 | Jinshanchamoshidou | 72.1 |
|  |  | G128 | Huangbaozhu | 58.7 |
|  |  | G131 | Jidou 9 | 56.8 |
|  |  | G133 | Handou 5 | 56.1 |
|  |  | G134 | Jidou 17 | 62.4 |
|  |  | G141 | Zhonghuang 30 | 57.3 |
|  |  | G142 | Baichengmoshidou | 58.3 |
|  |  | G143 | L-10 | 51.9 |
|  |  | G144 | Zaoshu 18 | 55.2 |
|  |  | G145 | Zhonghuang 10 | 63.8 |
|  |  | G149 | Hefeng 29 | 48.5 |
|  |  | G155 | Zhongdou 27 | 59.6 |
|  |  | G167 | Zhongpin 03-5373 | 70.2 |
|  |  | G168 | Wuxing 4 | 73.1 |
|  |  | G171 | Zhongpin 03-5334 | 66.0 |
|  | Lower | G19 | Kenfeng 22 | 41.9 |
|  |  | G26 | Hujiao 03-286 | 39.6 |
|  |  | G28 | Hujiao 04-528 | 39.1 |
|  |  | G32 | Suinong 14 | 44.9 |
|  |  | G36 | Bei 1361 | 43.6 |
|  |  | G43 | Sui 03-3046 | 40.9 |
|  |  | G65 | Suinong 10 | 41.2 |
|  |  | G80 | Kenjian 23 | 43.0 |
|  |  | G83 | Kenfeng 15 | 40.7 |
|  |  | G112 | Bei 1484 | 38.9 |
|  |  | G122 | Suinong 4 | 44.4 |
|  |  | G130 | Sui 05-7304 | 41.2 |
|  |  | G137 | Dongnong 48 | 45.3 |
|  |  | G140 | Kennong 30 | 42.0 |
|  |  | G147 | Neifeng 15 | 44.6 |
|  |  | G153 | Neifeng 11 | 44.0 |
|  |  | G160 | Dongnong 46 | 44.9 |
|  |  | G162 | Dongnong 1068 | 41.3 |
|  |  | G172 | Fengshou 6 | 41.4 |
| R3 (day) | Higher | G1 | Tiedou 50 | 65.2 |
|  |  | G2 | Datun xiaoheidou | 76.0 |
|  |  | G7 | Zhonghuang 20 | 68.4 |
|  |  | G18 | Liao 98072 | 63.9 |
|  |  | G30 | Hersong 2 | 64.5 |
|  |  | G31 | Jinzhou 4-1 | 70.3 |
|  |  | G33 | Tiejiasilihuang | 62.2 |
|  |  | G53 | Zhongpin 95-5388 | 73.5 |
|  |  | G55 | L-59 Peking | 88.5 |
|  |  | G57 | Ji 06B7 | 85.3 |
|  |  | G68 | Huayou 446 | 70.3 |
|  |  | G87 | Jingshanpu | 59.6 |
|  |  | G88 | Qinganheidou | 63.6 |
|  |  | G94 | Fangzhengmoshidou | 66.7 |
|  |  | G98 | Zhongzuo J4133 | 73.8 |
|  |  | G99 | Jilin 30 | 65.5 |
|  |  | G100 | Ji 100 | 60.3 |
|  |  | G108 | Zhonghuang 6 | 75.0 |
|  |  | G113 | Williams82 | 63.0 |
|  |  | G114 | Jindou 33 | 76.5 |
|  |  | G118 | L-9 | 85.3 |
|  |  | G119 | Suinong 1 | 60.0 |
|  |  | G123 | Jinshanchamoshidou | 79.9 |
|  |  | G128 | Huangbaozhu | 66.6 |
|  |  | G133 | Handou 5 | 66.6 |
|  |  | G134 | Jidou 17 | 72.3 |
|  |  | G135 | Aika 166 | 57.8 |
|  |  | G141 | Zhonghuang 30 | 68.4 |
|  |  | G143 | L-10 | 61.3 |
|  |  | G145 | Zhonghuang 10 | 73.7 |
|  |  | G149 | Hefeng 29 | 59.5 |
|  |  | G155 | Zhongdou 27 | 69.2 |
|  |  | G158 | Zhongpin 03-5179 | 57.6 |
|  |  | G164 | Jiunong 21 | 59.5 |
|  |  | G165 | Chamoshidou | 77.7 |
|  |  | G167 | Zhongpin 03-5373 | 77.4 |
|  |  | G168 | Wuxing 4 | 81.7 |
|  |  | G171 | Zhongpin 03-5334 | 76.0 |
|  | Lower | G15 | L-21 | 49.0 |
|  |  | G26 | Hujiao 03-286 | 47.5 |
|  |  | G27 | Heinong 44 | 50.7 |
|  |  | G28 | Hujiao 04-528 | 46.7 |
|  |  | G46 | Longxuan 1 | 53.4 |
|  |  | G51 | Hujiao 423 | 50.5 |
|  |  | G60 | L-28 | 49.2 |
|  |  | G65 | Suinong 10 | 51.7 |
|  |  | G75 | Heinong 48 | 53.9 |
|  |  | G79 | Dongnong 42 | 53.4 |
|  |  | G80 | Kenjian 23 | 50.9 |
|  |  | G93 | Ha 04-1824 | 52.2 |
|  |  | G101 | Suinong 30 | 50.2 |
|  |  | G106 | Heihe 38 | 48.8 |
|  |  | G115 | Kenfeng 18 | 53.4 |
|  |  | G117 | Mengdou 9 | 49.3 |
|  |  | G122 | Suinong 4 | 53.7 |
|  |  | G127 | Huajiang 4403 | 48.2 |
|  |  | G138 | L-79 | 46.9 |
|  |  | G139 | Zhonghuang 35 | 51.2 |
|  |  | G140 | Kennong 30 | 49.8 |
|  |  | G162 | Dongnong 1068 | 52.1 |
|  |  | G169 | Hefeng 55 | 52.2 |
|  |  | G172 | Fengshou 6 | 50.4 |
| R4 (day) | Higher | G2 | Datun xiaoheidou | 81.9 |
|  |  | G10 | Xiaobaiqi | 75.3 |
|  |  | G18 | Liao 98072 | 72.7 |
|  |  | G21 | Tiedou 51 | 66.7 |
|  |  | G30 | Hersong 2 | 70.8 |
|  |  | G33 | Tiejiasilihuang | 69.4 |
|  |  | G34 | Tiefeng 8 | 86.4 |
|  |  | G37 | L-5 | 71.5 |
|  |  | G41 | Jiunong 20 | 68.9 |
|  |  | G50 | Liaodou 3 | 68.5 |
|  |  | G55 | L-59 Peking | 93.7 |
|  |  | G59 | Jihuang 13 | 78.4 |
|  |  | G66 | Tiefeng 31 | 75.5 |
|  |  | G76 | Nova | 68.9 |
|  |  | G84 | Chasedou | 70.4 |
|  |  | G99 | Jilin 30 | 73.4 |
|  |  | G103 | Beidou 16 | 67.9 |
|  |  | G108 | Zhonghuang 6 | 80.5 |
|  |  | G109 | Jiyu 94 | 77.6 |
|  |  | G111 | Liaonong 2 | 72.1 |
|  |  | G113 | Williams82 | 71.7 |
|  |  | G123 | Jinshanchamoshidou | 85.9 |
|  |  | G128 | Huangbaozhu | 74.2 |
|  |  | G133 | Handou 5 | 74.7 |
|  |  | G134 | Jidou 17 | 79.3 |
|  |  | G141 | Zhonghuang 30 | 76.4 |
|  |  | G142 | Baichengmoshidou | 73.8 |
|  |  | G145 | Zhonghuang 10 | 79.8 |
|  |  | G164 | Jiunong 21 | 69.7 |
|  |  | G165 | Chamoshidou | 87.8 |
|  |  | G168 | Wuxing 4 | 88.6 |
|  | Lower | G15 | L-21 | 56.1 |
|  |  | G16 | Hongfeng11 | 59.9 |
|  |  | G36 | Bei 1361 | 59.3 |
|  |  | G42 | Duludou | 60.2 |
|  |  | G43 | Sui 03-3046 | 55.5 |
|  |  | G54 | Heihe 45 | 51.3 |
|  |  | G67 | Dongnong 49 | 54.1 |
|  |  | G70 | Huajiang 2 | 51.1 |
|  |  | G73 | Suinong 8 | 60.9 |
|  |  | G91 | Kennong 29 | 58.6 |
|  |  | G95 | Helongyoutai | 59.3 |
|  |  | G97 | Yuanbaojin | 59.6 |
|  |  | G101 | Suinong 30 | 56.7 |
|  |  | G105 | Mengdou 21 | 56.4 |
|  |  | G115 | Kenfeng 18 | 61.0 |
|  |  | G116 | Suinong 20 | 58.4 |
|  |  | G117 | Mengdou 9 | 54.4 |
|  |  | G120 | Kenfeng 14 | 60.5 |
|  |  | G122 | Suinong 4 | 60.5 |
|  |  | G137 | Dongnong 48 | 59.1 |
|  |  | G139 | Zhonghuang 35 | 58.4 |
|  |  | G140 | Kennong 30 | 57.8 |
|  |  | G172 | Fengshou 6 | 59.3 |
| R5 (day) | Higher | G1 | Tiedou 50 | 79.4 |
|  |  | G2 | Datun xiaoheidou | 88.6 |
|  |  | G6 | Tiedou 58 | 86.5 |
|  |  | G11 | Gongye 04L-141 | 80.0 |
|  |  | G12 | Tiedou 52 | 77.2 |
|  |  | G13 | L-57 | 76.0 |
|  |  | G18 | Liao 98072 | 80.0 |
|  |  | G30 | Hersong 2 | 77.0 |
|  |  | G31 | Jinzhou 4-1 | 80.6 |
|  |  | G33 | Tiejiasilihuang | 76.8 |
|  |  | G37 | L-5 | 79.9 |
|  |  | G50 | Liaodou 3 | 76.9 |
|  |  | G53 | Zhongpin 95-5388 | 89.0 |
|  |  | G55 | L-59 Peking | 99.2 |
|  |  | G58 | Marca. Joe lisa | 85.8 |
|  |  | G59 | Jihuang 13 | 85.1 |
|  |  | G66 | Tiefeng 31 | 82.5 |
|  |  | G68 | Huayou 446 | 84.5 |
|  |  | G76 | Nova | 76.1 |
|  |  | G86 | Xiaolimoshidou | 74.1 |
|  |  | G94 | Fangzhengmoshidou | 80.7 |
|  |  | G99 | Jilin 30 | 80.7 |
|  |  | G103 | Beidou 16 | 75.6 |
|  |  | G107 | Zhongdou 35 | 74.9 |
|  |  | G108 | Zhonghuang 6 | 86.6 |
|  |  | G114 | Jindou 33 | 88.3 |
|  |  | G118 | L-9 | 101.4 |
|  |  | G123 | Jinshanchamoshidou | 92.5 |
|  |  | G128 | Huangbaozhu | 80.8 |
|  |  | G133 | Handou 5 | 81.2 |
|  |  | G134 | Jidou 17 | 87.6 |
|  |  | G141 | Zhonghuang 30 | 83.2 |
|  |  | G142 | Baichengmoshidou | 79.1 |
|  |  | G145 | Zhonghuang 10 | 85.6 |
|  |  | G155 | Zhongdou 27 | 81.4 |
|  |  | G157 | Heilongjiang 41 | 77.0 |
|  |  | G165 | Chamoshidou | 95.1 |
|  | Lower | G3 | Ken 04-8579 | 65.6 |
|  |  | G15 | L-21 | 64.6 |
|  |  | G17 | Heinong 33 | 67.5 |
|  |  | G19 | Kenfeng 22 | 66.3 |
|  |  | G26 | Hujiao 03-286 | 59.4 |
|  |  | G36 | Bei 1361 | 67.7 |
|  |  | G42 | Duludou | 66.6 |
|  |  | G43 | Sui 03-3046 | 62.8 |
|  |  | G56 | Sui 04-5804 | 66.9 |
|  |  | G61 | Beifeng 9 | 63.4 |
|  |  | G65 | Suinong 10 | 66.4 |
|  |  | G67 | Dongnong 49 | 59.9 |
|  |  | G69 | Heihe 48 | 62.0 |
|  |  | G70 | Huajiang 2 | 58.3 |
|  |  | G71 | Suinong 29 | 67.0 |
|  |  | G77 | Beidou 14 | 60.6 |
|  |  | G79 | Dongnong 42 | 68.1 |
|  |  | G80 | Kenjian 23 | 63.8 |
|  |  | G81 | Heinong 55 | 67.2 |
|  |  | G83 | Kenfeng 15 | 64.9 |
|  |  | G95 | Helongyoutai | 65.2 |
|  |  | G97 | Yuanbaojin | 66.0 |
|  |  | G101 | Suinong 30 | 63.2 |
|  |  | G106 | Heihe 38 | 61.3 |
|  |  | G110 | Dongnong 44 | 58.0 |
|  |  | G116 | Suinong 20 | 65.3 |
|  |  | G120 | Kenfeng 14 | 68.1 |
|  |  | G122 | Suinong 4 | 68.1 |
|  |  | G124 | Longpin 03-311 | 66.1 |
|  |  | G127 | Huajiang 4403 | 61.8 |
|  |  | G130 | Sui 05-7304 | 60.4 |
|  |  | G137 | Dongnong 48 | 65.2 |
|  |  | G138 | L-79 | 61.0 |
|  |  | G151 | Hefeng 52 | 66.6 |
|  |  | G160 | Dongnong 46 | 67.4 |
|  |  | G161 | Dongnong 43 | 68.3 |
|  |  | G162 | Dongnong 1068 | 66.0 |
|  |  | G163 | Dongnong 56 | 68.0 |
|  |  | G172 | Fengshou 6 | 67.1 |
|  |  | G173 | Dunajika | 65.4 |
| R6 (day) | Higher | G2 | Datun xiaoheidou | 97.9 |
|  |  | G6 | Tiedou 58 | 96.1 |
|  |  | G12 | Tiedou 52 | 87.8 |
|  |  | G13 | L-57 | 86.6 |
|  |  | G18 | Liao 98072 | 90.8 |
|  |  | G22 | Tongnong 13 | 85.0 |
|  |  | G30 | Hersong 2 | 86.0 |
|  |  | G31 | Jinzhou 4-1 | 89.0 |
|  |  | G33 | Tiejiasilihuang | 88.0 |
|  |  | G34 | Tiefeng 8 | 105.1 |
|  |  | G37 | L-5 | 91.0 |
|  |  | G38 | Maoyandou | 83.8 |
|  |  | G41 | Jiunong 20 | 85.7 |
|  |  | G50 | Liaodou 3 | 86.4 |
|  |  | G58 | Marca. Joe lisa | 94.0 |
|  |  | G59 | Jihuang 13 | 94.4 |
|  |  | G66 | Tiefeng 31 | 93.8 |
|  |  | G68 | Huayou 446 | 97.1 |
|  |  | G76 | Nova | 86.8 |
|  |  | G78 | Kangxian 3 | 84.2 |
|  |  | G90 | Yapoche | 84.0 |
|  |  | G94 | Fangzhengmoshidou | 88.6 |
|  |  | G99 | Jilin 30 | 91.5 |
|  |  | G103 | Beidou 16 | 86.7 |
|  |  | G108 | Zhonghuang 6 | 95.0 |
|  |  | G114 | Jindou 33 | 98.2 |
|  |  | G118 | L-9 | 114.8 |
|  |  | G128 | Huangbaozhu | 89.1 |
|  |  | G134 | Jidou 17 | 98.1 |
|  |  | G141 | Zhonghuang 30 | 92.1 |
|  |  | G155 | Zhongdou 27 | 91.5 |
|  |  | G158 | Zhongpin 03-5179 | 85.4 |
|  |  | G159 | Hefeng 37 | 111.7 |
|  |  | G165 | Chamoshidou | 105.6 |
|  | Lower | G15 | L-21 | 75.3 |
|  |  | G23 | Bei 1873 | 73.0 |
|  |  | G24 | Hefeng 50 | 74.9 |
|  |  | G26 | Hujiao 03-286 | 67.8 |
|  |  | G36 | Bei 1361 | 78.1 |
|  |  | G42 | Duludou | 76.6 |
|  |  | G43 | Sui 03-3046 | 73.5 |
|  |  | G48 | Sui 03-3952 | 78.7 |
|  |  | G51 | Hujiao 423 | 72.9 |
|  |  | G56 | Sui 04-5804 | 75.5 |
|  |  | G61 | Beifeng 9 | 73.2 |
|  |  | G63 | Dongnong 50 | 78.0 |
|  |  | G65 | Suinong 10 | 77.1 |
|  |  | G67 | Dongnong 49 | 69.9 |
|  |  | G70 | Huajiang 2 | 69.0 |
|  |  | G72 | Hefeng 35 | 74.8 |
|  |  | G73 | Suinong 8 | 77.4 |
|  |  | G95 | Helongyoutai | 75.3 |
|  |  | G101 | Suinong 30 | 73.5 |
|  |  | G104 | Kebei 1 | 76.4 |
|  |  | G117 | Mengdou 9 | 69.8 |
|  |  | G122 | Suinong 4 | 77.2 |
|  |  | G127 | Huajiang 4403 | 73.5 |
|  |  | G136 | Sui 04-6018 | 76.4 |
|  |  | G137 | Dongnong 48 | 75.4 |
|  |  | G138 | L-79 | 71.5 |
|  |  | G140 | Kennong 30 | 74.9 |
|  |  | G148 | Dongnong 47 | 76.0 |
|  |  | G151 | Hefeng 52 | 77.5 |
|  |  | G154 | Heihexiaohuangdou | 71.3 |
|  |  | G160 | Dongnong 46 | 76.9 |
|  |  | G162 | Dongnong 1068 | 75.6 |
|  |  | G163 | Dongnong 56 | 78.7 |
|  |  | G169 | Hefeng 55 | 77.0 |
|  |  | G172 | Fengshou 6 | 77.4 |
|  |  | G173 | Dunajika | 76.7 |
| R7 (day) | Higher | G1 | Tiedou 50 | 117.2 |
|  |  | G5 | Liushitianhuancang | 112.0 |
|  |  | G6 | Tiedou 58 | 122.1 |
|  |  | G12 | Tiedou 52 | 119.8 |
|  |  | G18 | Liao 98072 | 120.3 |
|  |  | G33 | Tiejiasilihuang | 113.2 |
|  |  | G34 | Tiefeng 8 | 126.3 |
|  |  | G39 | Fengdihuang | 120.4 |
|  |  | G59 | Jihuang 13 | 121.7 |
|  |  | G62 | Zhongzuo 00-683 | 120.8 |
|  |  | G68 | Huayou 446 | 121.0 |
|  |  | G76 | Nova | 114.6 |
|  |  | G88 | Qinganheidou | 111.7 |
|  |  | G89 | Dongnong594 | 111.2 |
|  |  | G96 | Kexin 3 | 122.1 |
|  |  | G103 | Beidou 16 | 113.2 |
|  |  | G114 | Jindou 33 | 120.9 |
|  |  | G118 | L-9 | 128.9 |
|  |  | G128 | Huangbaozhu | 115.1 |
|  |  | G131 | Jidou 9 | 125.8 |
|  |  | G133 | Handou 5 | 122.6 |
|  |  | G155 | Zhongdou 27 | 115.7 |
|  |  | G159 | Hefeng 37 | 128.6 |
|  |  | G165 | Chamoshidou | 127.1 |
|  | Lower | G15 | L-21 | 98.0 |
|  |  | G23 | Bei 1873 | 98.1 |
|  |  | G35 | Silihuang | 101.0 |
|  |  | G42 | Duludou | 99.3 |
|  |  | G43 | Sui 03-3046 | 96.5 |
|  |  | G51 | Hujiao 423 | 98.3 |
|  |  | G60 | L-28 | 98.8 |
|  |  | G63 | Dongnong 50 | 100.2 |
|  |  | G65 | Suinong 10 | 100.4 |
|  |  | G67 | Dongnong 49 | 91.4 |
|  |  | G69 | Heihe 48 | 91.7 |
|  |  | G72 | Hefeng 35 | 102.5 |
|  |  | G73 | Suinong 8 | 100.3 |
|  |  | G101 | Suinong 30 | 98.0 |
|  |  | G116 | Suinong 20 | 101.9 |
|  |  | G137 | Dongnong 48 | 101.8 |
|  |  | G139 | Zhonghuang 35 | 98.3 |
|  |  | G148 | Dongnong 47 | 101.2 |
|  |  | G152 | Heihe 18 | 92.4 |
|  |  | G162 | Dongnong 1068 | 99.9 |
|  |  | G173 | Dunajika | 98.5 |

The mean of plant height, number of nodes of main stem, number of branches per plant, number of pods per plant, grain number per plant, grain yield per plant, hundred-grain weight, R1, R2, R3, R4, R5, R6, R7 of 173 genotypes across three environments were 83.0 cm, 16.6, 1.9, 53.3, 103.2, 19.6 g, 19.8 g, 41.3 d, 49.6 d, 58.9 d, 66.0 d, 73.0 d, 83.7 d, 107.9 d.

R1: beginning bloom, R2: full bloom, R3: beginning pod, R4: full pod, R5: beginning seed, R6: full seed, R7: beginning maturity.

**Supplementary Table 11.** The most stable genotypes with the highest or the lowest phenotype for fourteen agronomic traits across environments.

| **Traits** | | **Genotype** | | **Phenotypic value** |
| --- | --- | --- | --- | --- |
|  |  | **Number** | **Variety name** |  |
| Plant height (cm) | Highest | G92 | Heimoshidou | 123.9 |
|  | Lowest | G52 | Liaoxian 1 | 29.8 |
| Number of nodes main stem | Highest | G59 | Jihuang 13 | 19.9 |
|  | Lowest | G152 | Heihe 18 | 11.5 |
| Number of branches per plant | Highest | G171 | Zhongpin 03-5334 | 5.4 |
|  | Lowest | G61 | Beifeng 9 | 0.1 |
| Number of pods per plant | Highest | G2 | Datunxiaoheidou | 81.9 |
|  | Lowest | G71 | Suinong 29 | 32.9 |
| Grain number per plant | Highest | G134 | Jidou 17 | 141.0 |
|  | Lowest | G110 | Dongnong 44 | 57.7 |
| Grain yield per plant (g) | Highest | G119 | Suinong 1 | 29.6 |
|  | Lowest | G110 | Dongnong 44 | 10.5 |
| Hundred-grain weight (g) | Highest | G9 | Longquandadou | 28.5 |
|  | Lowest | G125 | Jilinchalihua | 7.9 |
| R1 (day) | Highest | G55 | L-59 Peking | 76.8 |
|  | Lowest | G70 | Huajiang 2 | 30.9 |
| R2 (day) | Highest | G55 | L-59 Peking | 82.0 |
|  | Lowest | G112 | Bei 1484 | 38.9 |
| R3 (day) | Highest | G55 | L-59 Peking | 88.5 |
|  | Lowest | G28 | Hujiao 04-528 | 46.7 |
| R4 (day) | Highest | G55 | L-59 Peking | 93.7 |
|  | Lowest | G70 | Huajiang 2 | 51.1 |
| R5 (day) | Highest | G118 | L-9 | 101.4 |
|  | Lowest | G110 | Dongnong 44 | 58.0 |
| R6 (day) | Highest | G118 | L-9 | 114.8 |
|  | Lowest | G26 | Hujiao 03-286 | 67.8 |
| R7 (day) | Highest | G118 | L-9 | 128.9 |
|  | Lowest | G67 | Dongnong 49 | 91.4 |

R1: beginning bloom, R2: full bloom, R3: beginning pod, R4: full pod, R5: beginning seed, R6: full seed, R7: beginning maturity.
